# The thiol-sulfoxonium ylide photo-click reaction for bioconjugation†

**DOI:** 10.1039/d2sc05650j

**Published:** 2022-12-03

**Authors:** Chuan Wan, Zhanfeng Hou, Dongyan Yang, Ziyuan Zhou, Hongkun Xu, Yuena Wang, Chuan Dai, Mingchan Liang, Jun Meng, Jiean Chen, Feng Yin, Rui Wang, Zigang Li

**Affiliations:** a State Key Laboratory of Chemical Oncogenomics, School of Chemical Biology and Biotechnology, Peking University Shenzhen Graduate School Shenzhen 518055 P. R. China lizg.sz@pku.edu.cn lizg@szbl.ac.cn; b Pingshan Translational Medicine Center, Shenzhen Bay Laboratory Shenzhen 518118 P. R. China wangrui@szbl.ac.cn yinfeng@szbl.ac.cn; c College of Chemistry and Chemical Engineering, Zhongkai University of Agriculture and Engineering Guangzhou 510225 P. R. China; d National Cancer Center/National Clinical Research Center for Cancer/Cancer Hospital & Shenzhen Hospital, Chinese Academy of Medical Sciences and Peking Union Medical College Shenzhen 518116 P. R. China

## Abstract

Visible-light-mediated methods were heavily studied as a useful tool for cysteine-selective bio-conjugation; however, many current methods suffer from bio-incompatible reaction conditions and slow kinetics. To address these challenges, herein, we report a transition metal-free thiol-sulfoxonium ylide photo-click reaction that enables bioconjugation under bio-compatible conditions. The reaction is highly cysteine-selective and generally finished within minutes with naturally occurring riboflavin derivatives as organic photocatalysts. The catalysts and substrates are readily accessible and bench stable and have satisfactory water solubility. As a proof-of-concept study, the reaction was smoothly applied in chemo-proteomic analysis, which provides efficient tools to explore the druggable content of the human proteome.

## Abstract 2

Thiol-sulfoxonium ylide photo-click chemistry: an ultrafast and highly cysteine-specific method for protein functionalization and proteome profiling.

## Graphical abstract

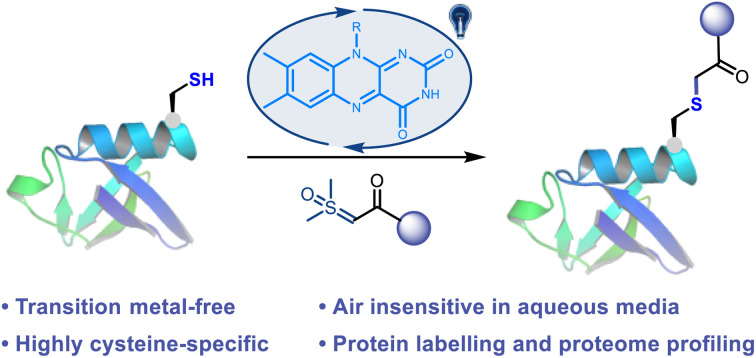

## Introduction

**Fig. 1:**
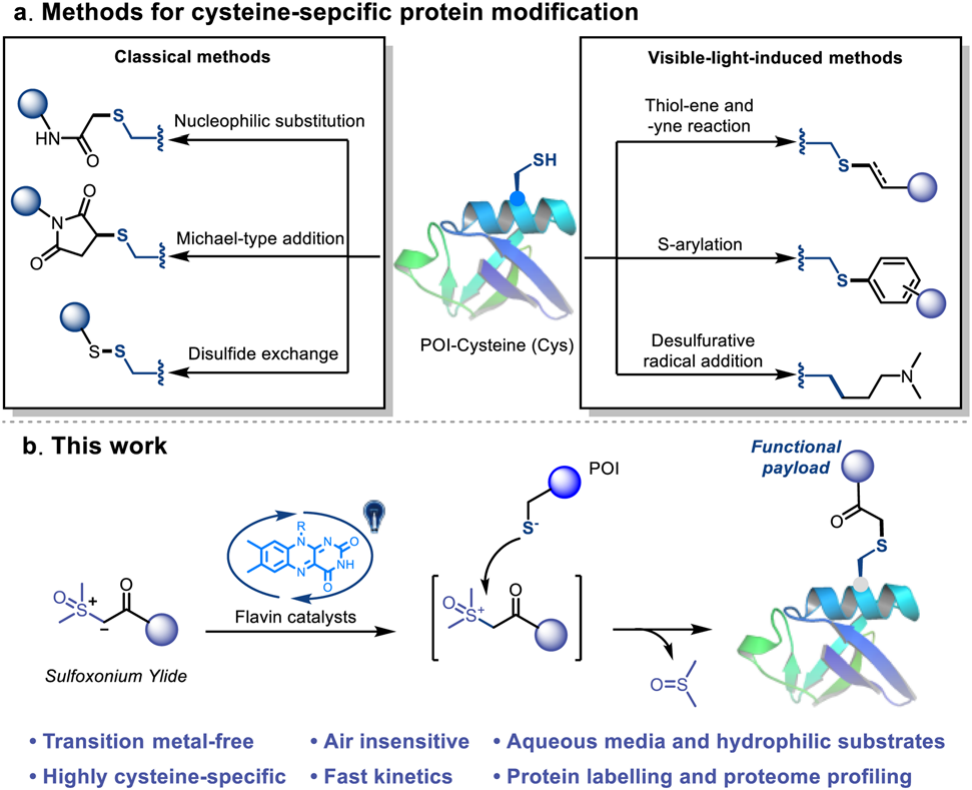
Cysteine-selective bioconjugations. (a) Reported methods for cysteine-selective bioconjugation. (b) This work: visible-light-induced thiol-sulfoxonium ylide click reaction.

Site-selective chemical modifications of proteins are of great importance for contemporary chemical biology, biotechnology and pharmaceutical development.^1–5^ Controllable and precise protein chemical modification enables the probing of the interactions of proteins and small molecules, including drug candidates, metabolites or protein post-translational modifications (PTMs). Chemo-proteomic analysis could further be used to study the proteome-scale dynamic interactions and modifications.^6^ Diverse bioconjugation technologies have been developed to achieve chemo- and site-selective functionalization of natural amino acids (AAs) for the purpose of selective protein modification.^7–20^ The low abundance and unique reactivity of the thiol side chain of cysteine (Cys) make it an ideal candidate for bioconjugation chemistry.^21–33^ Michael-type addition, nucleophilic substitution and disulfide exchange reaction represent the classical approaches for Cys-selective bioconjugation (Fig. 1a).^34–43^ The advantages of fast reaction kinetics and robustness enable the widespread applications of these methods. However, each method presents particular advantages and disadvantages. A wide variety of nucleophilic molecules and AAs in a biological context notably interrupt the chemo-selectivity of these methods.^44^ For example, more than 1000 reactive cysteine sites in the human proteome have been identified using an electrophilic iodoacetamide (IA) probe,^45^ but the covalent ligand/inhibitor for Cys was mainly constructed by using relatively low reactivity warheads (such as chloroacetamide and acrylamide) due to the potential off-target effects.^46–48^

Alternatively, several visible-light-induced methods, including thiol–ene^49–51^ and thiol-yne reactions,^52^*S*-arylation^53,54^ and desulfurative functionalization,^55,56^ have been well established as efficient Cys-based bioconjugation methods (Fig. 1a). The multidimensional controls and novel reaction pathway of these methods provide versatile tools for bioorthogonal applications.^57,58^ Despite the advances, the thiyl-radical based reactions suffered from relatively slow kinetics and non-biocompatible conditions, such as the use of transition metal catalysts and/or stoichiometric amounts of the oxidant. Another challenge is the side reactions caused by the highly oxidizing conditions, which may severely disrupt the structure of the protein or cause unwanted cross-linking. Consequently, the requirement of a visible-light-induced bioconjugation that possess the advantages of both the photocatalytic and classical methods, *i.e.* spatiotemporal control, fast reaction kinetics and high chemo-selectivity under mild and biocompatible conditions, is still unmet.

Sulfoxonium ylide is one of the most stable and industrially safe ylides that contains a nucleophilic carbon attached to sulfoxide.^59–61^ Significant efforts were devoted to the study of X–H insertion of sulfoxonium ylides (Fig. S1†).^62–70^ Brønsted acid or transition metal catalysts are generally needed for these methods, and they mainly go through either a nucleophilic addition or an electrophilic metal carbenoid pathway.^59,60^ Previously, sulfoxonium ylide was utilized to design cathepsin X-selective activity-based probes (ABPs) by Edgington-Mitchell *et al.*;^71^ the thiol-sulfoxonium ylide reaction is relatively slow and requires specific conditions and substrates.^64^ Motivated by the hydrogen atom transfer (HAT) pathway of photocatalysis,^72^ we envisioned that the sulfoxonium ylide species may act as a sacrificial hydrogen acceptor to provide the highly reactive sulfoxonium species, and then undergo a fast nucleophilic substitution with thiol groups.^64^

Herein, we report a novel visible-light-induced thiol-sulfoxonium ylide click reaction that enables Cys-selective bioconjugation under physiological conditions (Fig. 1b). Readily accessible, bench stable and water-soluble sulfoxonium ylides were prepared and utilized in this study. By exploring the conditions in aqueous media, derivatives of riboflavin (vitamin B2) behaved as the most efficient photosensitizers. The practicality of the reaction was further investigated with a series of Cys-containing peptides and proteins. In addition, a chemo-proteomic application was performed to further validate its biocompatibility and possibility as an efficient tool for exploring the druggable content of the human proteome.

## Results and discussion

### Reaction condition optimization and substrate scope

We initiated the investigation of the thiol-sulfoxonium ylide reaction by applying luminescence quenching screening with a series of photosensitizers and sulfoxonium ylide 1a. The derivatives of flavin gave rise to higher luminescence quenching (26% to 43% quenching fraction) than that of other metal and organic photocatalysts when sulfoxonium ylide 1a was present, indicating an efficient energy transfer or electron transfer event from flavins to sulfoxonium ylide.^73^ We then screened photocatalytic reactions between 1a and Ac-Cys-OH 2 in aqueous solution under visible-light irradiation (*λ*_max_ = 450 nm), and the S–H insertion product 2a was detected by ^1^H NMR. The yield of the product 2a with flavins (63% to 86% yield, Table S1, Fig. S2 and S3†) correlated well with their high quenching fractions.

To further optimize the reaction conditions, we defined the thiol-sulfoxonium ylide reaction with a riboflavin tetraacetate (RFTA) photocatalyst under 450 nm light in water as the standard conditions (86% yield, entry 1, Table 1), and the observed second-order reaction constant *k*_2_ was estimated to be 0.172 M^−1^ s^−1^ (Fig. S4†). The light irradiation and photosensitizer (RFTA) are both essential for this reaction (entry 2–7). To examine the influences of different solvents, phosphate buffer (PB, pH 7.4 in D_2_O), protic solvent methanol-d_4_ as well as aprotic solvent DMSO-d_6_ and acetonitrile-d_3_ were used as the reaction solvent (entry 8–11). Interestingly, the reactions in aqueous and protic solvents gave significantly higher yields (87% and 74%) than that in aprotic solvents (18% and 26%), and the disulfide by-product was detected as the main product in aprotic solvents (see detailed data in the ESI†), hinting at the importance of a hydrogen source. Notably, the α-carbonyl methylene of product 2a was fully deuterated in D_2_O (Fig. S4†). Furthermore, the addition of a radical trapper (2,2,6,6-tetramethylpiperidine-1-oxyl, TEMPO) fully quenched the reaction (entry 12), indicating a radical involving pathway in this reaction. Additional reaction conditions, including oxygen, temperature and scale variants, were investigated to assess the sensitivity of the current protocol (Table 1).^74,75^ The average yields for typical protic and aprotic solvents were assessed, respectively. As a result, except for aprotic solvents, the transformation was shown to be insensitive, suggesting the robustness of this thiol-sulfoxonium ylide reaction.

**Table 1:** Visible-light-induced S–H insertion of sulfoxonium ylide

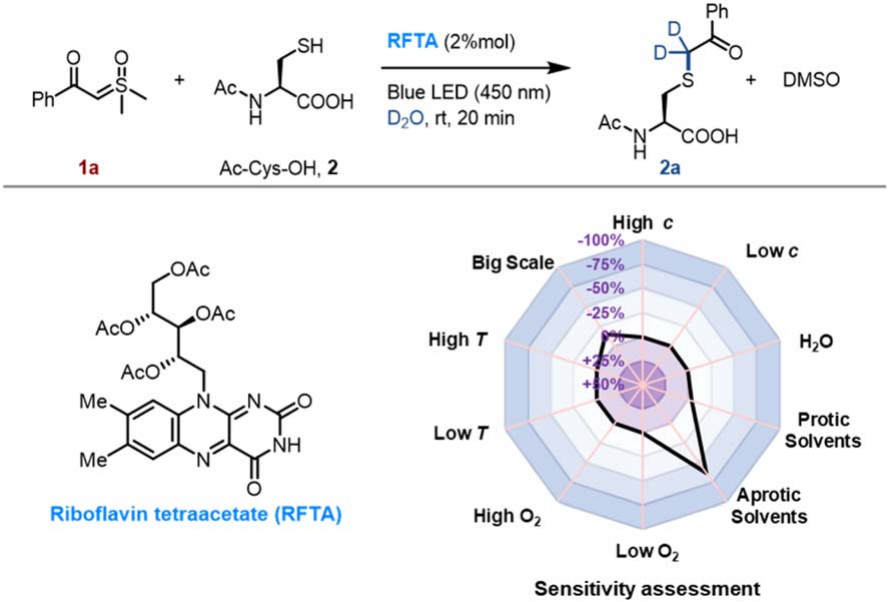		
Entry	Deviations from the standard conditions^a^	Yield^b^ (%)
1	None	86
2	Dark (12 h)	0
3	Dark (50 °C, 12 h)	0
4	White light	58
5	UV light (365 nm)	0
6	Absence of RFTA	0
7	Flavin mononucleotide (FMN) instead of RFTA	81
8	PB buffer (pH 7.4) instead of D_2_O	87
9	Methanol-d_4_ instead of D_2_O	74
10	DMSO-d_6_ instead of D_2_O	18
11	Acetonitrile-d_4_ instead of D_2_O	26
12^c^	Addition of TEMPO	0

a Standard conditions: 1a (50 mM), 2 (25 mM) and photocatalysts (2% mol) under light irradiation (450 nm) for 20 min in D_2_O at rt.

b Yields were determined by ^1^H NMR with dimethyl sulfone (MSM) as the internal standard.

c Methanol-d_4_ was used as the solvent.

**Fig. 2:**
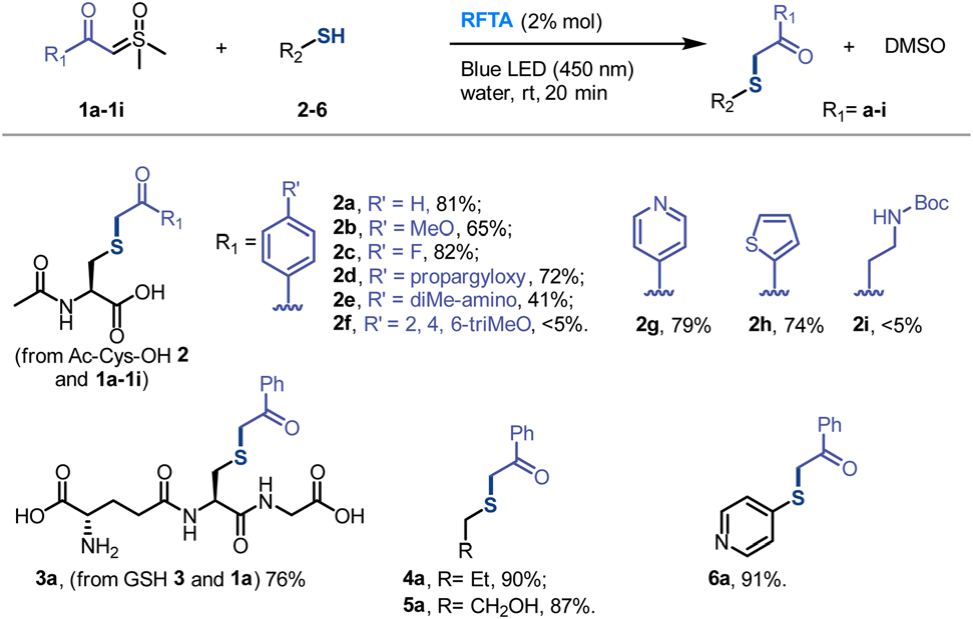
Scope of the S–H insertion of sulfoxonium ylides. Conditions: 1 (1 mmol, 2 equiv.), thiol 2–6 (0.5 mmol, 1 equiv.) and RFTA (2% mol) under light irradiation (450 nm) for 20 min in water (8 mL) at rt. Isolated yields were reported.

Then different sulfoxonium ylides and thiol substrates were tested to gain more insights into this photoreaction. For the scope of sulfoxonium ylides, 1b, 1d, 1e, 1f and 1i with electron-donating groups, 1c with an electron-withdrawing group and 1g and 1h with a hetero-aromatic ring were tested (Fig. 2). The isolated yields of products (2c) from electron-withdrawing sulfoxonium ylides were higher than those of electron-donating compounds (2b, 2d, 2e, 2f and 2i), and the hetero-aromatic ring containing substrates gave moderated yields, such as 2g (79%) and 2h (74%). For the scope of thiol substrates, glutathione (GSH) 3, propanethiol 4, mercaptoethanol 5 and 4-mercaptopyridine 6 were subjected to the reaction with 1a, and satisfactory yields (76 to 91%) were achieved (see more substrates in Fig. S5†). As a result, the thiol-sulfoxonium ylide reaction was carried out smoothly with satisfactory yield and substrate tolerance.

### Mechanistic investigations

To investigate the mechanism, the kinetic experiments, photosensitization of substrates (1a and 2) and RFTA as well as control reactions were studied in detail. The kinetic studies showed that the rate of product 2a generation is only related to the concentration of 1a, but not to the concentration of 2 under the fixed photocatalytic conditions (light source and photocatalyst) (see Fig. S6 and S4e† for additional data and discussion), suggesting that the rate-determine step does not involve the thiol substrates. In this regard, we assumed that the photocatalytic pathway is mainly associated with sulfoxonium ylide, and the photosensitization study may provide additional evidence.

**Fig. 3:**
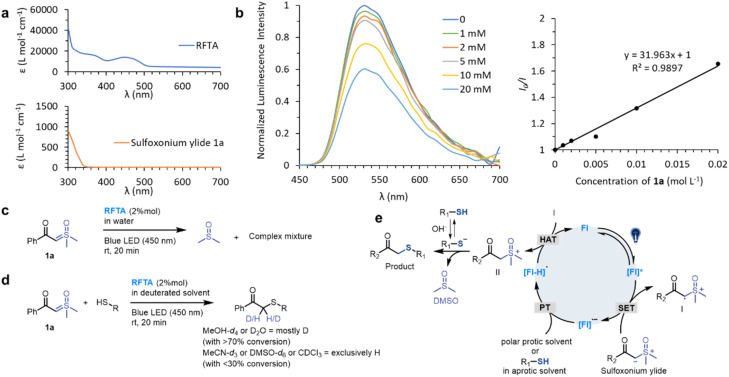
Mechanistic studies. (a) UV-vis absorption spectra of RFTA and 1a. (b) Stern–Volmer luminescence quenching study between the photocatalyst RFTA and sulfoxonium ylide 1a. (c) Control reaction: photolysis of 1a. (d) Hydrogen sources in deuterated solvents. (e) Proposed mechanism.

The UV-vis absorption spectra showed that the visible light (400–500 nm) was exclusively absorbed by RFTA (Fig. 3a and S7†). Stern–Volmer luminescence quenching studies were then performed between RFTA and substrate 1a and 2, respectively (Fig. 3b and S8†). Apparently, favorable luminescence quenching between RFTA and 1a was observed, but no significant interactions between RFTA and 2 were observed. Thus, the energy transfer or electron transfer event between RFTA and 1a was proved. In addition, a control reaction was carried out in the absence of a thiol substrate, and the sulfoxonium ylide was decomposed to DMSO, indicating the highly reactive nature of the potential intermediate (Fig. 3c and S9a†).

To examine the proton transfer event, the hydrogen sources were investigated in deuterated protic and aprotic solvents. As summarized in Fig. 3d (see detailed data in Fig. S9†), the reactions were performed smoothly in protic solvents and most of the carbonyl-α-carbon was deuterated. In contrast, the desired reactions were inefficient in aprotic solvents and the α-carbon was exclusively hydrogenated. Consequently, we concluded that the hydrogen source is mainly from the solvent in the protic solvents, and thiol may be the hydrogen source in the aprotic solvents.

Based on the mechanistic experiments, a photocatalytic activation of sulfoxonium ylide was proposed (Fig. 3e). The flavin (Fl) photocatalyst is light-promoted to the singlet-excited state followed by intersystem crossing to result in the triplet-excited state [Fl]*.^76^ The triplet-excited flavins were reported as a strong single electron oxidant (*E*^red^_1/2_ = 2.2 V *versus* Fc/Fc^+^ electrode for RFTA) and they should undergo facile single electron transfer (SET) with sulfoxonium ylide (*E*^red^_1/2_ = 1.3 V *versus* Fc/Fc^+^ electrode for 1a) to furnish radical cation I along with the radical anion [Fl]˙^−^ (see the ESI† for experimental details). The flavin species can act as a base (p*K*_a_ [RFTA-H]˙ = 8.3),^77,78^ favoring proton transfer (PT) from the protic solvent or thiol group. This unique property of flavin derivatives can partially explain their outstanding catalytic efficiency. The radical cation I could abstract a hydrogen atom from the redox state [Fl-H]˙ to turn over the ground-state photocatalyst Fl^79^ and generate the highly reactive sulfoxonium species II. The nucleophilic substitution between the sulfoxonium II and ionized thiol is a fast step^64^ which furnishes the target product and DMSO.

### Peptide and protein modification

Visible-light-induced protein modification is a powerful tool for the spatiotemporal control of bioconjugation.^58^ Thus, we further focused on the photoreaction of peptides and proteins. The initial examination of the reaction between a model peptide 7 (1 mM) and sulfoxonium ylide 1a (10 mM) under standard conditions for small molecules led to oxidation and decomposition of the peptide. Hence, we considered the possibility of adding suitable additives to quench the unwanted side reactions. Yoon *et al.* used aromatic amine as a redox mediator for improving the photocatalyzed thiol–ene reaction.^51^ In addition, thiourea was used as an additive in Gaunt *et al.*'s report to avoid non-specific oxidation and labeling in a protein methionine-selective alkylation.^80^ Thus, we tried a thiourea (10 mM) additive and it resulted in a 76% conversion of peptide 7 (Fig. S10†). Notably, the rest of peptide 7 (24%) was oxidized to form a disulfide dimer and the addition of thiourea efficiently inhibited the decomposition of peptide (Fig. 5b). We further evaluated the effects of other (thio)urea derivatives, and found that they all showed protective effects on peptide, but their protecting effects were significantly weaker than that of thiourea (Table S2†).

With the thiourea additive, the conditions for peptide modification were further optimized. Photoredox conditions are essential in the presence of thiourea (Fig. S10†). RFTA was still the most efficient photocatalyst in the reaction of peptide, and the kinetic investigation demonstrated that the starting peptide was fully converted within 1 min. In addition, degassing and nitrogen protection of the model peptide reaction could further decrease the formation of by-products.

**Fig. 4:**
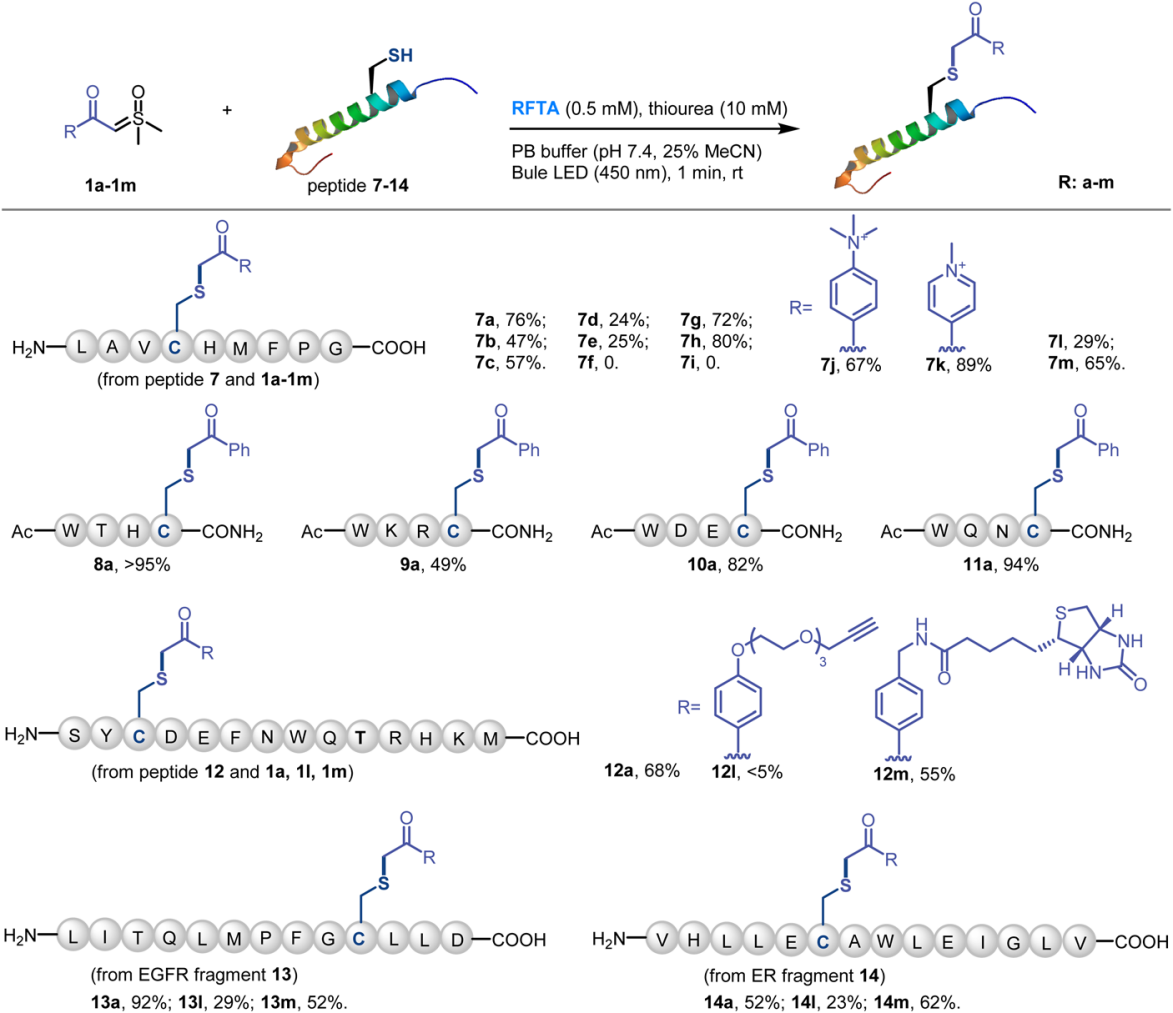
Scope of the Cys-selective peptide modification. Conditions: sulfoxonium ylide 1 (10 mM), peptide 7–14 (1 mM), RFTA (0.5 mM) and thiourea (10 mM) under light irradiation (450 nm) for 1 min at rt in PBS buffer (pH 7.4, 25% MeCN was added to dissolve peptides) as the solvent. Yield was determined by LC-MS.

Then various sulfoxonium ylides and peptides were subjected to the visible-light-induced thiol-sulfoxonium ylide reaction under optimized conditions (Fig. 4). First, the sulfoxonium ylide 1a–1i and four additional substrates 1j–1m were reacted with model peptide 7. Except for the highly electron-donating substrates, most of the reactions gave satisfactory yields (57–89%). Moreover, four short peptides 8–11, containing various nucleophilic residues, were designed and prepared for the investigation of AA tolerance. Under the standard conditions with sulfoxonium ylide 1a, moderate to high yields were obtained for all of the four peptides (49% to >95%). Furthermore, we tested a model peptide 12, which contains all of the 14 reactive AA residues, for the chemo-selective study of the reaction. 68% of Cys-adducted product 12a was obtained, highlighting the excellent chemo-selectivity of the thiol-sulfoxonium ylide reaction. And the biologically relevant substrates (bioorthogonal handle 1l and biotin 1m) reacted with peptide 12 in a different manner. Only a trace amount of 12l was detected, and moderate yield (55%) of 12m was observed. To further examine the practicality of the reaction, two protein fragments 13 and 14 were derived from two important cancer targets epidermal growth factor receptor (EGFR) and estrogen receptor (ER), respectively. Similarly, the reactions of 1a and 1m with 13 and 14 gave higher yields (52–92%) than that of 1l (23–29%). All of the peptide products were characterized by MS/MS analysis on the cysteine site, and the adducted positions were further confirmed by the MS/MS searching for all the potential functionalization on nucleophilic residues in product 12a, 13a and 14a, which demonstrated that the cysteine site is the only possible reaction position (see detailed data in the ESI†). To further evaluate the chemo-selectivity, we have performed reactions on peptides that contain nucleophilic residues but without free cysteine (Fig. S11†), and no product was observed under the standard conditions for the thiol-sulfoxonium ylide reaction. All in all, the current thiol-sulfoxonium ylide protocol presents a versatile platform for Cys-specific bioconjugation under physiological conditions.

**Fig. 5:**
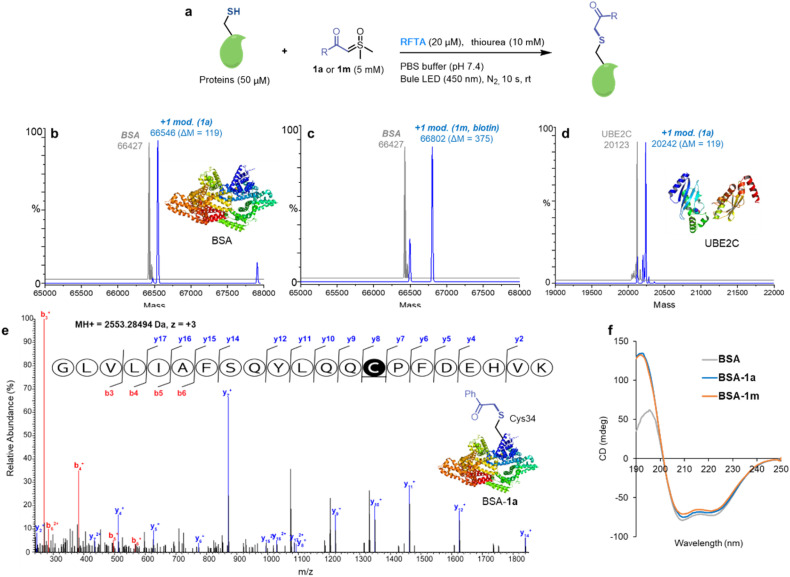
Cysteine-specific modification of proteins. Standard conditions: sulfoxonium ylide 1a or 1m (5 mM), specific proteins (50 μM), RFTA (20 μM) and thiourea (10 mM) under light irradiation (450 nm) for 10 s at rt in PBS buffer (pH 7.4) under nitrogen protection. (a) The reaction conditions of proteins. (b) ESI-TOF MS analysis of the BSA-1a adduct (MW = 66 546 Da). (c) ESI-TOF MS analysis of the BSA-1m adduct (MW = 66 802 Da). (d) ESI-TOF MS analysis of the ubiquitin-conjugating enzyme 2C-1a (UBE2C-1a) adduct (MW = 20 242 Da). (e) MS/MS analysis of BSA-1a on Cys34. (f) The circular dichroism (CD) analysis of BSA adducts.

Having optimized the reaction conditions with peptides, the visible-light-induce thiol-sulfoxonium ylide reaction for chemical modification of proteins was then investigated (Fig. 5). In order to eliminate the interference of possible side reactions, nitrogen protection was performed for protein modification (Fig. 5a). Bovine serum albumin (BSA) was used as a model protein due to its unique free Cys34 residue. ESI-TOF MS analysis of reactions between BSA and 1a/1m afforded >90% modification (Fig. 5b and c). In addition, the reaction between 1a and ubiquitin-conjugating enzyme 2C (UBE2C) gave >70% modification (Fig. 5d). Significantly, an exceedingly fast (10 s reaction time) reaction was found. Furthermore, the Cys34 selectivity of the thiol-sulfoxonium ylide reaction was confirmed by LC-MS/MS analysis on the BSA-1a adduct (Fig. 5e), and circular dichroism (CD) analysis was also performed to check the potential conformational change (Fig. 5f). Similar CD spectra were observed for the control BSA and two adducts, highlighting that there was no significant change in their secondary structural content under the photoreaction conditions. Thus, the thiol-sulfoxonium ylide photo-click reaction was capable of covalently modifying proteins with controllable and ultrafast kinetics and outstanding cysteine selectivity under mild and biocompatible conditions.

With the efficient labeling of proteins, we then applied the thiol-sulfoxonium ylide reaction in chemo-proteomic applications. As a relatively low-reactive electrophile, the biological application of a sulfoxonium ylide warhead was highly reliant on the design of ligand-based probes. For example, the practicability of sulfoxonium ylide electrophiles as one ABP to detect the cathepsin X activity was evidenced.^71^ But, it's difficult to directly apply the sulfoxonium ylide warhead to globally profile the reactive and ligandable cysteinome. In fact, due to the impact of the off-target effect, researchers usually use different warheads in the study of chemoproteomics and covalent ligand/inhibitors, respectively.^46–48^ Thus, we proposed that the visible-light-activated condition may enable the direct application of sulfoxonium ylide as a probe to profile the functional proteome, and thus provide more visions to explore the druggable contents by using this hydrophilic, stable and cysteine-selective probe.

**Fig. 6:**
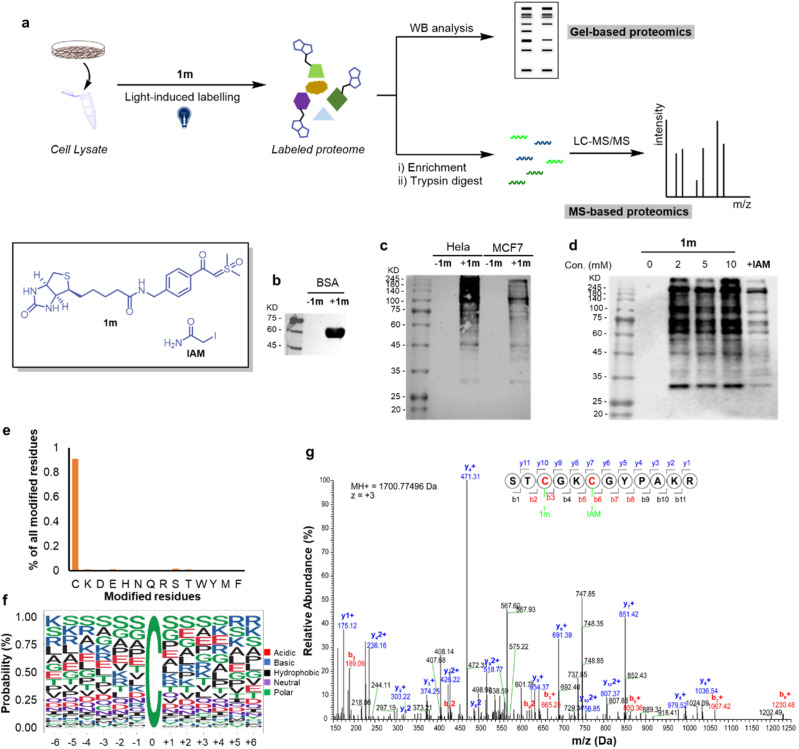
The chemo-proteomic applications of the thiol-sulfoxonium ylide photo-click reaction. (a) The workflow of gel- and MS-based proteomics. (b) The western-blot (WB) analysis of BSA-1m. (c) The WB analysis of the reaction between 1m and cells (HeLa and MCF-7 cell lysates). (d) The WB analysis of the reaction between 1m and HeLa cell lysate at different concentrations (2–10 mM) and competitive profiling with the cysteine reactive IAM. (e) Characterizing amino-acid selectivity in proteomes. Percentage of unique peptides labeled on each nucleophilic amino acid by using 1m (5 mM) in HeLa (*n* = 2) proteomes. (f) Consensus motifs identified by 1m. (g) Annotated MS2 of 1m-labeled RPL37 peptide identified in the proteomic studies.

Along these lines, we conducted protein profiling by both gel-based and MS-based protocols (Fig. 6a). Initially, we checked the western-blot (WB) analysis of the biotinylated BSA-1m adduct, and obvious bands emerged (Fig. 6b), demonstrating that the biological function of biotin was preserved after the reaction. Next, we switched the protein to human cells (HeLa and MCF7 cell lysates) and found that 1m exhibited relatively higher labeling effects in HeLa cells (Fig. 6c). We then proceeded to investigate the labeling efficiency and cysteine selectivity of 1m in a competition assay with the known highly reactive and cysteine-selective reagent, iodoacetamide (IAM). 1m exhibited strong immunofluorescence intensity at a concentration of 2, 5, and 10 mM. Pretreatment of excess IAM successfully decreased the intensity of the bands, indicating that the labeling of 1m predominantly occurs at cysteine residues with a high cysteine selectivity (Fig. 6d). Furthermore, we employed the MS-based proteomics technique, and 3246 modified cysteine sites from HeLa cells were identified by the light-induced labelling of 1m (see more details in ESI Table S3†). The percentage of unique peptide modification for each nucleophilic amino acid was plotted for 1m. As shown in Fig. 6e, 1m primarily labeled cysteine residues with a significantly high cysteine reactivity (>90%). Interestingly, the alignment of local sequences flanking the modified cysteines in HeLa showed that 1m prefers to label cysteine residues that surrounded by serine residues (Fig. 6f). As an example, by analyzing the b, y ion mode, annotated MS/MS of the 1m-labeled RPL37 peptide was sufficient to confirm the Cys-site selectivity of 1m labelling in the chemoproteomic studies (Fig. 6g). Taken together, as a proof-of-concept study, the visible-light-induced conditions have enabled the direct application of the thiol-sulfoxonium ylide reaction for protein profiling in the proteome. This result not only validated the biocompatibility of the photo-click reaction, but also provided a possibility to develop a covalent ligand/inhibitor for cysteine by using the same reactive chemotypes of chemo-proteomics analysis under appropriate conditions.

## Conclusions

In summary, we report here a flavin derivative-catalyzed thiol-sulfoxonium ylide photo-click reaction that enables Cys-specific bioconjugation. The reaction is bio-compatible, metal-free and has extraordinarily fast kinetics. Most of the sulfoxonium ylides have satisfactory aqueous solubility and bench stability, and the photocatalysts are the derivatives of biocompatible flavins. The chemo-selectivity, functional group tolerance and scope of the reaction are then examined by exploiting the reaction of various substrates, peptides and proteins under biocompatible conditions. This metal-free and highly efficient thiol-sulfoxonium ylide photo-click reaction furnishes a possibility that possess the currently dominant advantages of both the photocatalytic and classical methods. Furthermore, the chemo-proteomic applications were also performed smoothly with more than 3000 identified cysteine sites and >90% cysteine selectivity, which may provide more visions to explore the druggable content in the human proteome.

## Data availability

Primary data for experiments described herein, as well as, NMR, HPLC, mass and MS/MS spectrometry data and the data for proteomic analysis and bioinformatics analysis are provided in the ESI.†

## Author contributions

The research was conceived by FY, RW and ZL. The experiments were designed by CW, ZH, DY, FY, RW and ZL. The chemical experiments were conducted by CW, ZH, ZZ, HX, CD, JM and JC. The biochemical assays and chemo-proteomic experiments were performed by DY, YW, ML, FY and RW. The manuscript was written and proofread by all authors.

## Conflicts of interest

There are no conflicts to declare.

## Supplementary Material

SC-014-D2SC05650J-s001

SC-014-D2SC05650J-s002
