# Supplementary material for: The thiol-sulfoxonium ylide photo-click reaction for bioconjugation
Source: Chem Sci. 2022 Dec 3;14(3):604–12. doi: 10.1039/d2sc05650j (PMC9847666; doi:10.1039/d2sc05650j)
Supplement: SC-014-D2SC05650J-s002 [file SC-014-D2SC05650J-s002.pdf]

Electronic Supplementary Material (ESI) for Chemical Science.
This journal is © The Royal Society of Chemistry 2022

Thiol-sulfoxonium ylide photo-click reaction for bioconjugation

Chuan Wan,[†a] Zhanfeng Hou,[†a] Dongyan Yang,[†c] Ziyuan Zhou,[d] Hongkun Xu,[a] Yuena Wang,[a] Chuan Dai,[a] Mingchan Liang,[b] Jun Meng,[d] Jiean Chen,[b] Feng Yin,[*b] Rui Wang,[*b] and Zigang Li[*a, b]

[a.] *State Key Laboratory of Chemical Oncogenomics, School of Chemical Biology and Biotechnology, Peking University Shenzhen Graduate School, Shenzhen, 518055, P. R. China.*

[b.] *Pingshan translational medicine center, Shenzhen Bay Laboratory, Shenzhen, 518118, P. R. China.*

[c.] *College of Chemistry and Chemical Engineering, Zhongkai University of Agriculture and Engineering, Guangzhou, 510225, P. R. China.*

[d.] *National Cancer Center/National Clinical Research Center for Cancer/Cancer Hospital & Shenzhen Hospital, Chinese Academy of Medical Sciences and Peking Union Medical College, Shenzhen, 518116, P. R. China*

† These authors contributed equally to this work

* Address correspondence to yinfeng@szbl.ac.cn, wangrui@szbl.ac.cn, lizg.sz@pku.edu.cn, lizg@szbl.ac.cn.

Table of Contents

1. Supplementary figures and tables 3
2. Supplementary material and information for organic chemistry 12
    2.1. General information 12
    2.2. Synthesis of sulfoxonium ylide 12
    2.3. General procedure A for the thiol-sulfoxonium ylide reaction 18
3. Supplementary material and information for peptide modification 21
    3.1. General information 21
    3.2. General procedure B for the reaction of peptide 22
    3.3. Characterization data for the reaction of peptide 7 23
    3.4. Characterization data for the reaction of peptide 8 37
    3.5. Characterization data for the reaction of peptide 9 39
    3.6. Characterization data for the reaction of peptide 10 41
    3.7. Characterization data for the reaction of peptide 11 43
    3.8. Characterization data for the reaction of peptide 12 45
    3.9. Characterization data for the reaction of peptide 13 51
    3.10. Characterization data for the reaction of peptide 14 57
    3.11. Characterization data for the reaction of peptide I-III 62
4. Mechanistic experiments 64
    4.1. Luminescence screening 64
    4.2. Stern-Volmer luminescence quenching studies 64
    4.3. UV/vis absorption studies 64
    4.4. Cyclic Voltammogram 65
5. Supplementary material and information for protein modification 67
    5.1. General information 67
    5.2. General procedure C for the reaction of protein and proteome 67
    5.3. LC-MS analysis 67
    5.4. LC-MS/MS analysis 67
6. Supplementary material and information for chemoproteomics analysis 69
    6.1. Western blot 69
    6.2. MS-based proteomics 69
7. Reference 70
8. NMR spectra 71

1. Supplementary figures and tables

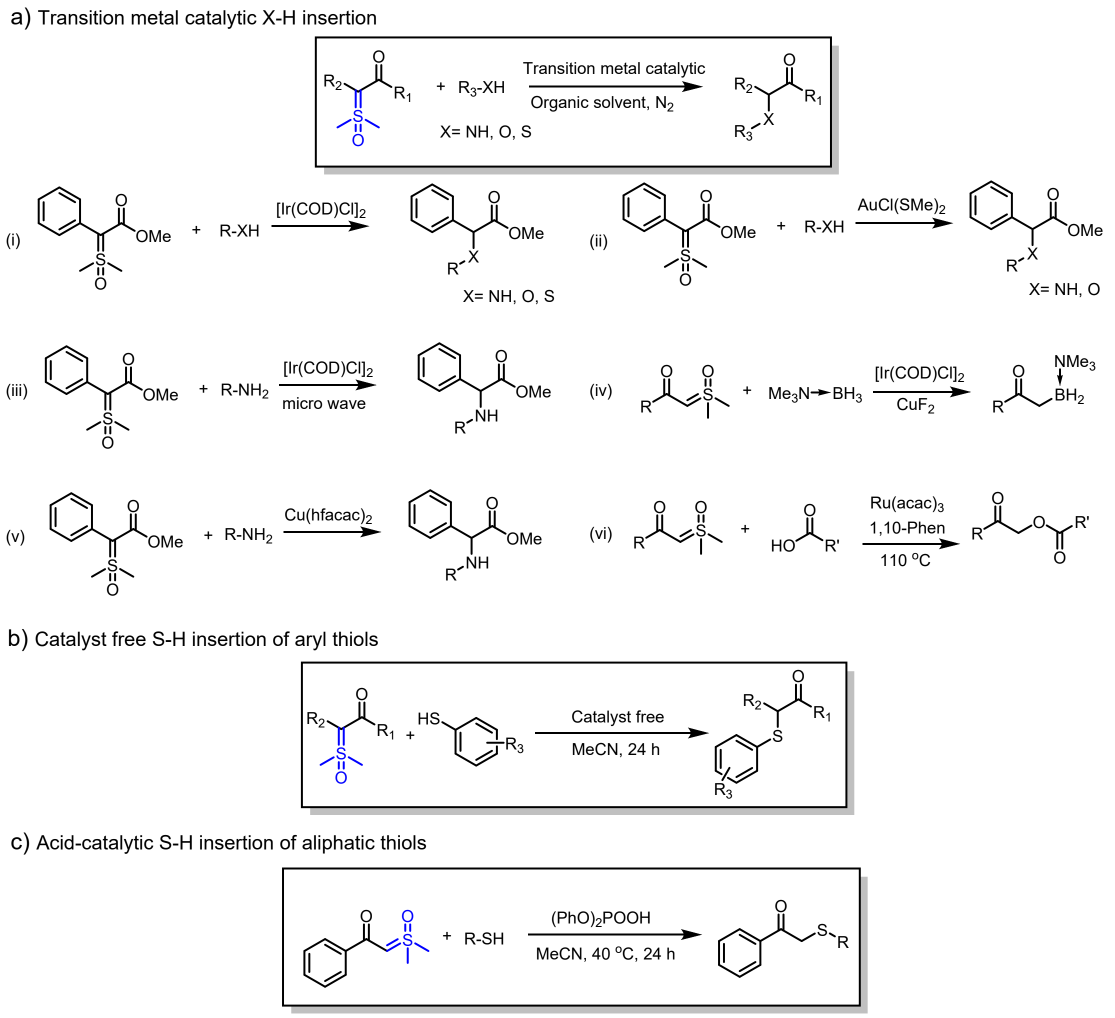

Figure S1. Recent reports of X-H insertion of sulfoxonium ylides. (**a**) Transition metal catalytic X-H insertion.[1-6] (**b**) Catalyst free S-H insertion of aryl thiols.[7] (**c**) Acid-catalytic S-H insertion of aliphatic thiols.[7]

Table S1. Screening of photocatalysts for thiol-sulfoxonium ylide reaction.

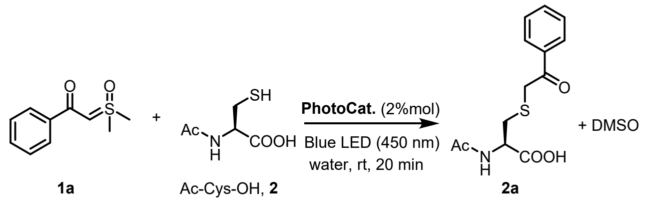

Entry	PhotoCat.	Quenching fraction *F*	Yield (%)
1	-	-	N.D.
2	$Ru(bpz)_3(PF_6)_2$	<5	<10
3	Rose Bengal (RB)	9	37
4	$Ir(dtbbpy)(ppy)_2(PF_6)$	13	38
5	$MesAcrClO_4$	13	48
6	Riboflavin	26	63
7	Flavin mononucleotide (FMN)	33	81
8	Riboflavin tetrabutyrate (RFTB)	39	83
9	Riboflavin tetraacetate (RFTA)	43	86
10	Alloxazine-Bu	32	76[a]

Conditions: **1a** (50 mM), **2** (25 mM) and photocat. (2% mol) under light irradiation (450 nm, 18 W) for 20 min in D_2O at rt with dimethyl sulfone as internal standard. [a] The 400 nm, 18 W light irradiation was used.

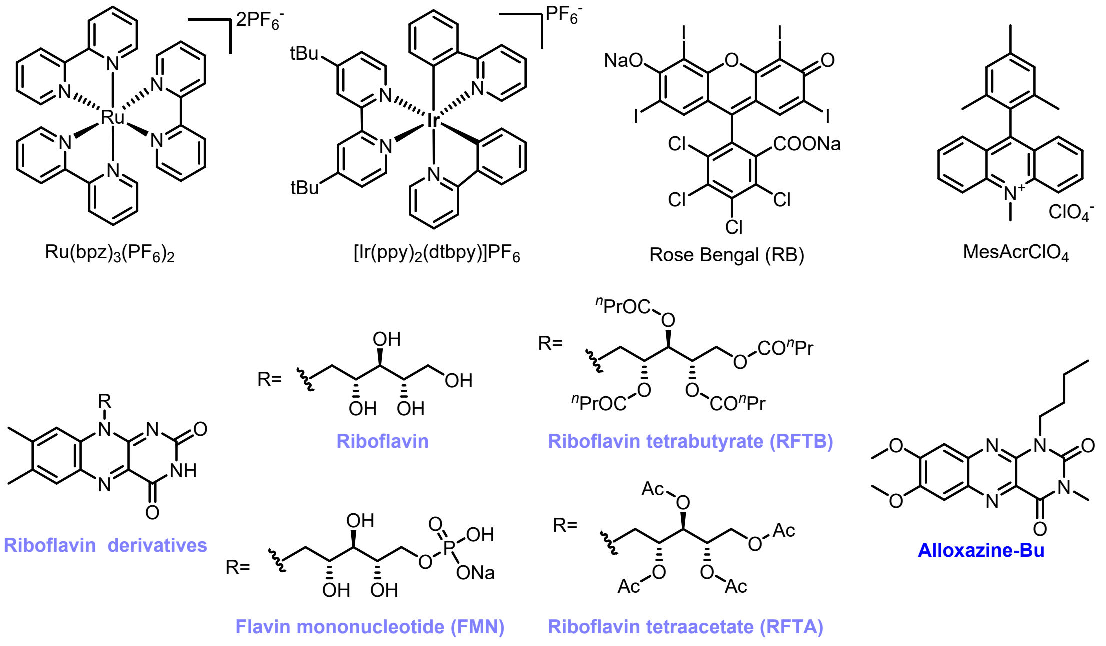

Figure S2. The chemical structure of the photocatalysts used in this work.

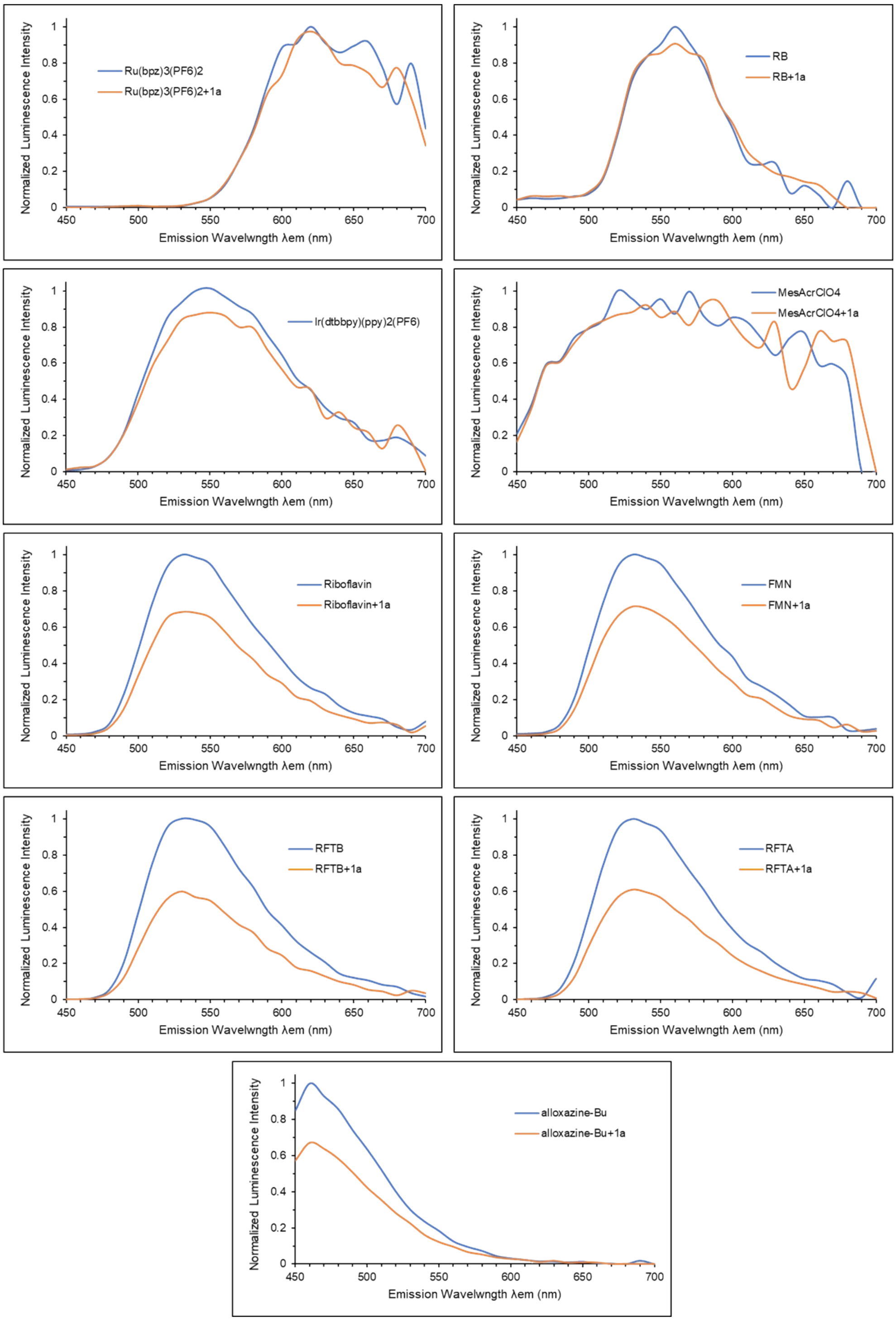

Figure S3. Luminescence spectra of the screening of the photocatalysts with sulfoxonium ylide **1a**.

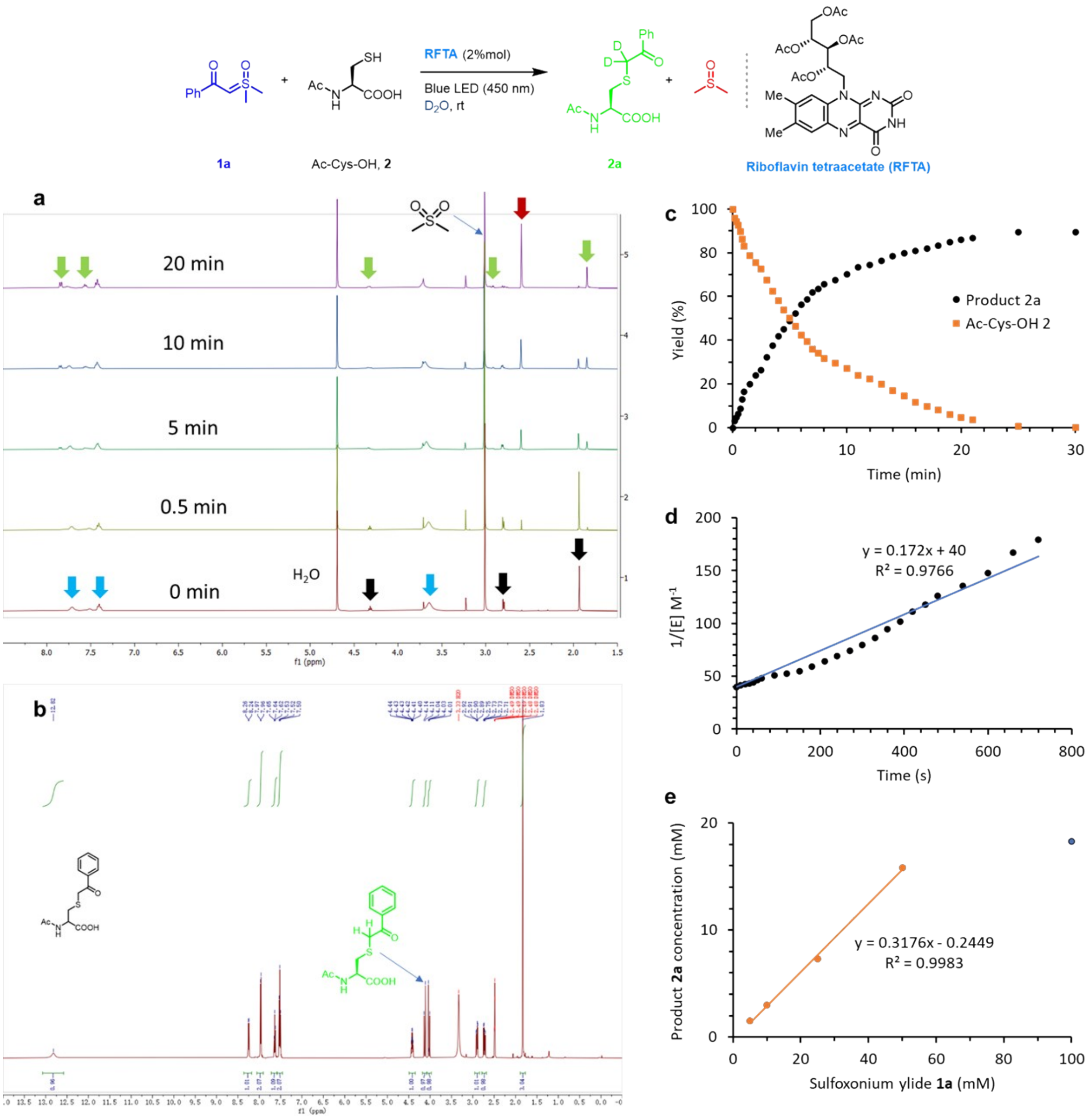

Figure S4. Kinetic studies of the reaction between **1a** and **2**. (**a**) Kinetic study of the S-H insertion of sulfoxonium ylide by ^{1}H NMR analysis. Blue, black, green and red arrows point to signals of sulfoxonium ylide **1a**, Ac-Cys-OH **2**, the product **2a** and leaving DMSO. Conditions: **1a** (50 mM), **2** (25 mM) and RFTA (2% mol) under light irradiation (450 nm, 18 W) in D_2O at rt with dimethyl sulfone as internal standard. (**b**) ^{1}H NMR (DMSO-d_6) assignment of α-carbonyl methylene of the purified product **2a** from H_2O. (**c**) The yield versus time plot of the standard reaction. (**d**) Estimation of the second-order reaction constant (k_{obs} = 0.172 M^{-1} s^{-1}) using the linearly-fitted region of the 1/[**2**] versus time plot. (**e**) The correlation of product **2a** concentration versus starting concentration of sulfoxonium ylide **1a** plot. The data was from **Figure S6b**. Conditions: **2** (25 mM) and RFTA (2% mol) with sulfoxonium ylide **1a** (5, 10, 25, 50 and 100 mM) under light irradiation (450 nm, 18 W) in D_2O at rt for 5 min with dimethyl sulfone as internal standard.

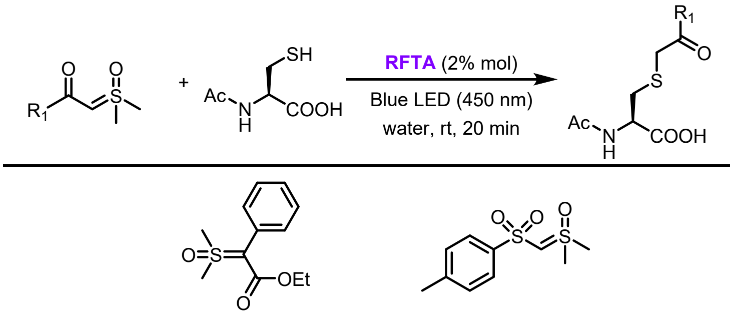

S1, Trace **S2**, N.D.

Figure S5. Low reactive and inactive sulfoxonium ylides for the visible-light-induced thiol-sulfoxonium ylide reaction. The trace amount of products were detected by LC-MS.

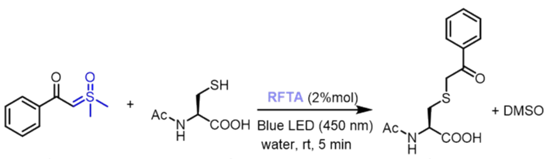

1a Ac-Cys-OH, **2** **2a**

(a) 50 mM sulfoxonium ylide **1a**			
Entry	Cys concentration (mM)	5 min yield (%) for Cys	product concentration (mM)
1	50	31	15.5
2	25	62	15.6
3	10	89	8.9
4	5	90	4.5
5	2.5	92	2.3

(b) 25 mM Cys **2**				
Entry	Ylide concentration (mM)	5 min yield (%) for Cys	product concentration (mM)	5 min yield (%) for Ylide
1	100	73	18.3	18
2	50	63	15.8	32
3	25	29	7.3	29
4	10	12	3.0	30
5	5	6	1.5	30

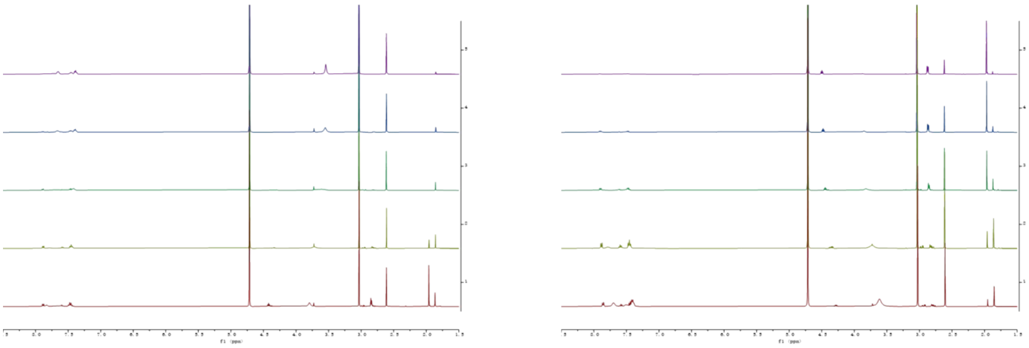

Figure S6. The kinetic studies with various concentrations of Cys or sulfoxonium ylide.

a) The 5 min reaction efficiency of gradient change of Cys concentrations by fixing the concentration of sulfoxonium ylide **1a** (50 mM). For the low concentration of **2**, all of the reactions (entry 3-5) exhibited almost quantitative conversions. For the high concentration of **2**, entry 1 and 2 shown that the same amount of product **2a** (15.5 and 15.6 mM) was observed for 50 mM and 25 mM **2**, suggesting that the reaction rate remains the same as the Cys **2** concentration increases.

b) The 5 min reaction efficiency of gradient change of sulfoxonium ylide concentrations by fixing the concentration of Cys **2** (25 mM). Not as fast as the reactions with excessive **1a**, the excess of **2** led to around 30% conversions of **1a** (entry 4 and 5). Moreover, the conversions of equivalent and slightly excess **1a** (entry 2 and 3) were also around 30%, and the rate of product **2a** generation was linearly related to the concentration of starting concentration of **1a** (5-50 mM, also see **Figure S4e**), indicating that the activation rates of sulfoxonium ylide **1a** were almost the same under the fixed photocatalytic conditions.

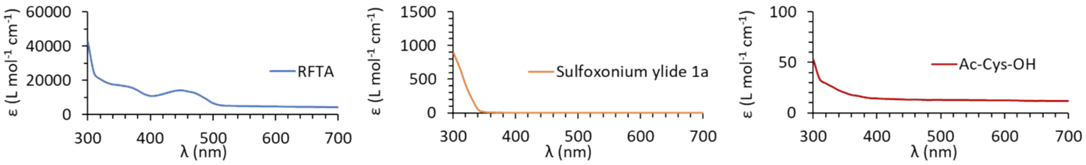

Figure S7. UV/vis absorption spectra of the starting materials: RFTA, sulfoxonium ylide **1a** and Ac-Cys-OH **2**.

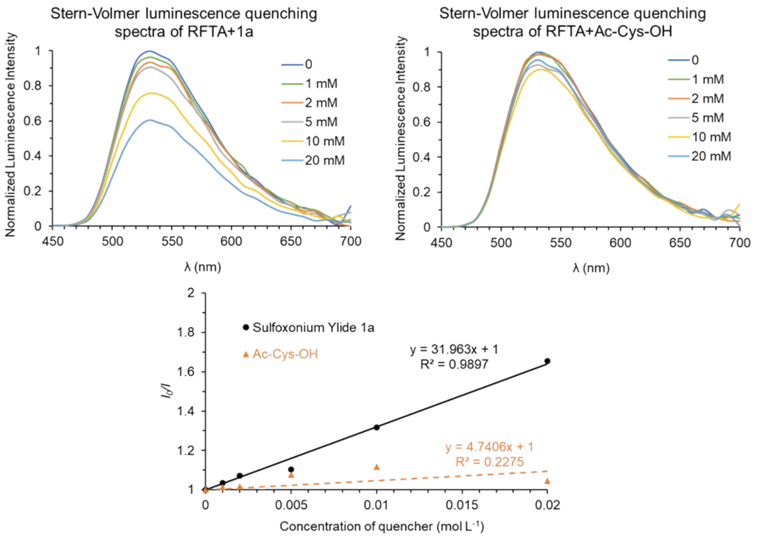

Figure S8. Stern-Volmer luminescence quenching analysis for RFTA with sulfoxonium ylide **1a** and Ac-Cys-OH **2**.

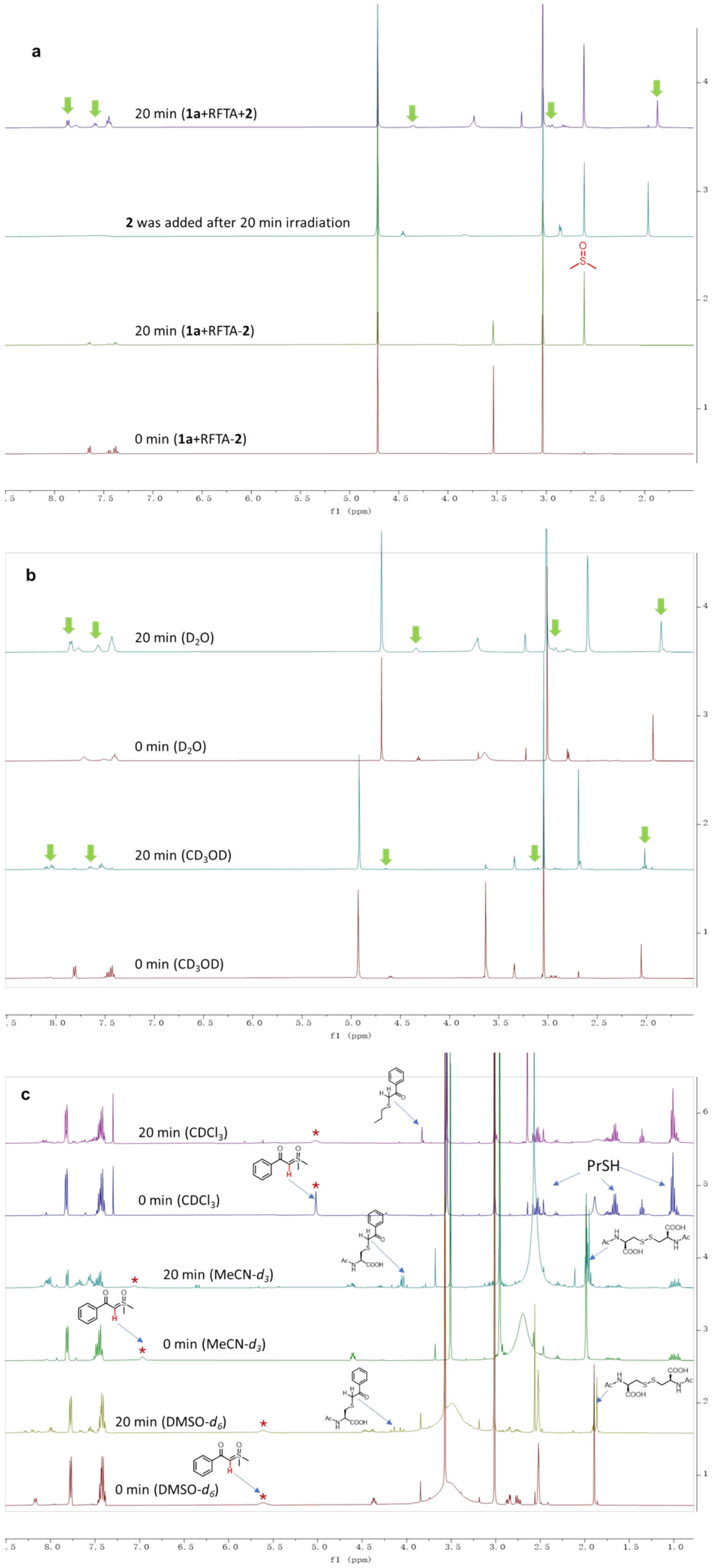

Figure S9. Mechanistic studies of the reaction between sulfoxonium ylide **1a** and Ac-Cys-OH **2** by *in situ* ^{1}H NMR. A standard reaction was defined as: **1a** (50 mM), **2** (25 mM) and RFTA (2%mol) under light irradiation (450 nm) for 20 min in D_2O (or other mentioned solvent) at rt. Green arrows point to

signals of the product **2a**. (**a**) Control experiments. Bottom to top traces: **1a** (50 mM) and RFTA (2%mol) under light irradiation (450 nm) at 0 min and 20 min in D_2O at rt in the absence of **2**; **2** (25 mM) was added after 20 min irradiation; and the trace of standard reaction at 20 min. (**b**) The reactions in polar protic solvent. Bottom to top traces: a standard reaction was conducted in CD_3OD at 0 min and 20 min; and the trace of standard reaction at 0 min and 20 min. (**c**) The reactions in nonpolar and polar aprotic solvent. Bottom to top traces: a standard reaction was conducted in DMSO-d_6 at 0 min and 20 min; a standard reaction was conducted in MeCN-d_3 at 0 min and 20 min; and **1a** (50 mM), *n*-PrSH (25 mM) and RFTA (2%mol) under light irradiation (450 nm) at 0 min and 20 min in D_2O at rt. The red asterisk marks the peak of the active hydrogen atom of sulfoxonium ylide **1a**.

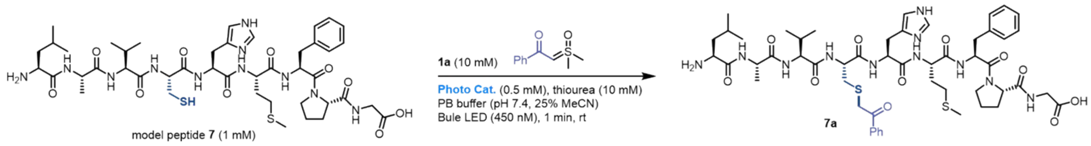

a

Entry	PhotoCat.	Quenching fraction *F*	Conversion (%)
1	-	-	N.D.
2	Ru(bpz)$_3$(PF$_6$)$_2$	<5	N.D.
3	Rose Bengal	9	28
4	MesAcrClO$_4$	13	35
5	Riboflavin	26	42
6	FMN	33	59
7	RFTB	39	62
8	RFTA	43	76
9	RFTA	43	85[a]

Increasing Quenching fraction *F*

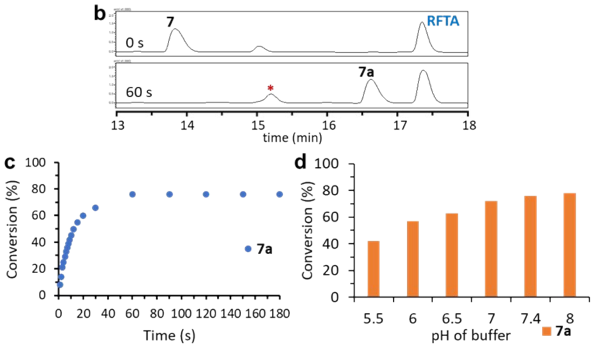

Figure S10. Reaction with peptides. (**a**) Optimization of the reaction between sulfoxonium ylide **1a** and model peptide **7**. Conditions: **1a** (10 mM), **7** (1 mM), photocat. (0.5 mM) and thiourea (10 mM) under light irradiation (450 nm) for 1 min at rt using PB buffer (pH 7.4 with 25% MeCN) as solvent. Conversion was determined by LC-MS. [a] The conversion in parenthesis refers to the reactions under nitrogen protection. (**b**) HPLC traces the reaction. The red asterisk marks the peak of oxidized by-product (disulfide). (**c**) Estimation of the conversion versus time plot. (**d**) Estimation of the conversion versus pH plot.

Table S2. Optimization of additives for peptide cysteine modification.

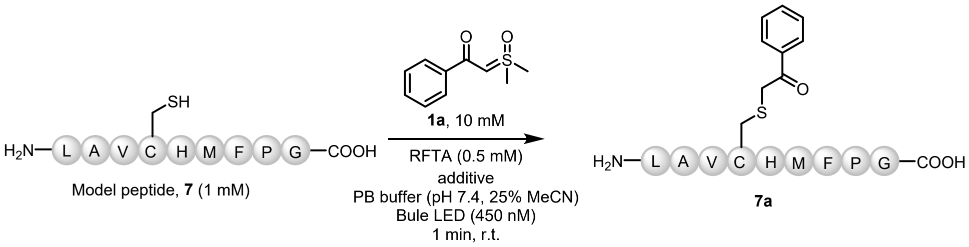

Entry	Additive	Additive loading (mM)	Conversion (%)
1		20	77
2		10	76
3	Thiourea	5	66
4		1	48
5		0.5	45
6	Phenylthiourea	10	55
7	Benzylthiourea	10	17
8	Urea	10	36
9	Phenylthiourea	10	51

Conditions: **1a** (10 mM), **7** (1 mM), RFTA (0.5 mM) and additive (10 mM) under light irradiation (450 nm, 40 W) for 1 min at rt using PB buffer (pH 7.4, 25% MeCN) as solvent.

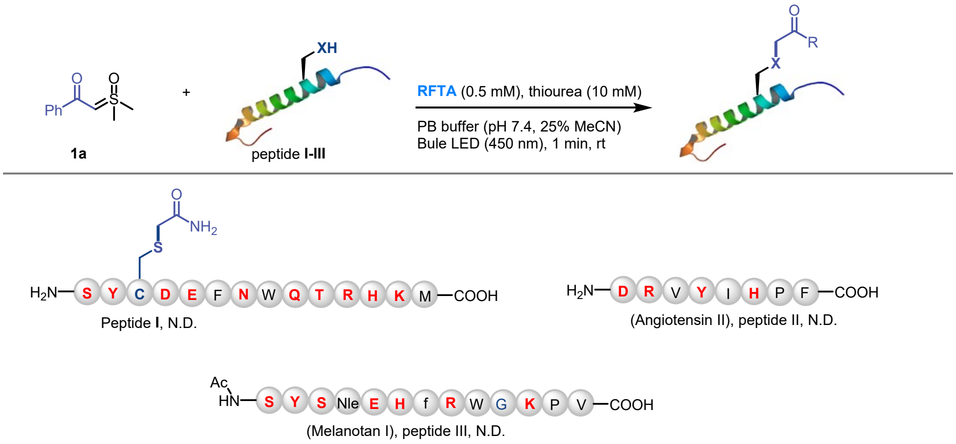

Figure S11. Scope of the peptide reaction without free cysteine residue. Conditions: sulfoxonium ylide 1 (10 mM), peptide **I-III** (1 mM), RFTA (0.5 mM) and thiourea (10 mM) under light irradiation (450 nm) for 1 min at rt in PBS buffer (pH 7.4, 25% MeCN was added to dissolve peptides) as solvent.

2. Supplementary material and information for organic chemistry

2.1. General information

All chemical reagents are commercially available from *Energy Chemical* without purification. The reactions were monitored by TLC (silica gel-G). **Nuclear Magnetic Resonance (NMR)** spectra were recorded on Bruker 400 MHz or 500 MHz spectrometer using trimethylsilane (TMS) as internal standard under ambient temperature (20 °C). **High-Resolution Mass Spectrometry (HRMS)** were measured on a Q_Exactive_Focus. **Mass Spectrometry** (**MS**) to screen the molecular weight of HPLC fractions were carried out on SHIMAZU LC-MS 8030 in positive ion mode.

General method for preparative LC

Preparative LC were performed on a Shimadzu LC-6AD system equipped with Shimadzu Shim-pack GIST C18 column (20 × 250 mm, 5 μm; room temperature). Water (containing 0.1% TFA, A phase) and pure CH_3CN (B phase) were used as solvents in linear gradient mixtures at a flow rate of 8 mL·min^{-1}.

2.2. Synthesis of sulfoxonium ylide

2.2.1. Preparation of 4-nitrophenol ester

4-Nitrophenol esters **S3-S7** were prepared as intermediates for the synthesis of sulfoxonium ylides. The preparation of 4-nitrophenol ester was followed the reported procedure.[8]

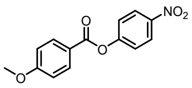

S3, white powder, yield 82%. ^{1}H NMR (400 MHz, Chloroform-*d*) δ 8.34 – 8.26 (m, 2H), 8.19 – 8.10 (m, 2H), 7.44 – 7.36 (m, 2H), 7.04 – 6.96 (m, 2H), 3.91 (s, 3H). ^{13}C NMR (101 MHz, Chloroform-*d*) δ 164.57, 164.06, 156.09, 145.40, 132.68, 125.36, 122.81, 120.85, 114.23, 55.75.

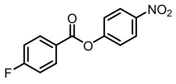

S4, white powder, yield 87%. ^{1}H NMR (400 MHz, Chloroform-*d*) δ 8.34 – 8.26 (m, 2H), 8.26 – 8.17 (m, 2H), 7.45 – 7.37 (m, 2H), 7.26 – 7.15 (m, 2H). ^{13}C NMR (101 MHz, Chloroform-*d*) δ 167.91, 165.36, 163.40, 155.71, 145.60, 133.22, 133.13, 125.42, 124.96, 124.93, 122.75, 116.34, 116.12. ^{19}F NMR (376 MHz, Chloroform-*d*) δ -102.96.

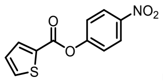

S5, white powder, yield 75%. ^{1}H NMR (400 MHz, Chloroform-*d*) δ 8.40 – 8.31 (m, 2H), 8.06 (dd, *J* = 3.8, 1.3 Hz, 1H), 7.77 (dd, *J* = 5.0, 1.3 Hz, 1H), 7.51 – 7.43 (m, 2H), 7.25 (dd, *J* = 5.0, 3.8 Hz, 1H). ^{13}C NMR (101 MHz, Chloroform-*d*) δ 159.63, 155.36, 145.49, 135.60, 134.62, 131.74, 128.41, 125.35, 122.61.

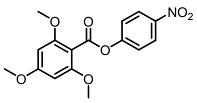

S6, white powder, yield 90%. ^{1}H NMR (400 MHz, Chloroform-*d*) δ 8.36 – 8.27 (m, 2H), 7.48 – 7.40 (m, 2H), 6.19 (s, 2H), 3.90 (s, 6H), 3.89 (s, 3H). ^{13}C NMR (101 MHz, Chloroform-*d*) δ 163.72, 159.57, 156.21, 145.28, 126.12, 125.22, 122.78, 115.76, 90.73, 56.18, 55.60.

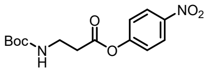

S7, white powder, yield 83%. ^{1}H NMR (500 MHz, Chloroform-*d*) δ 8.27 – 8.21 (m, 2H), 7.31 – 7.25 (m, 2H), 5.15 (t, J = 6.4 Hz, 1H), 3.51 (q, J = 6.2 Hz, 2H), 2.82 (t, J = 6.1 Hz, 2H), 1.43 (s, 9H).

2.2.2. Preparation of intermediates for the synthesis of 1l and 1m

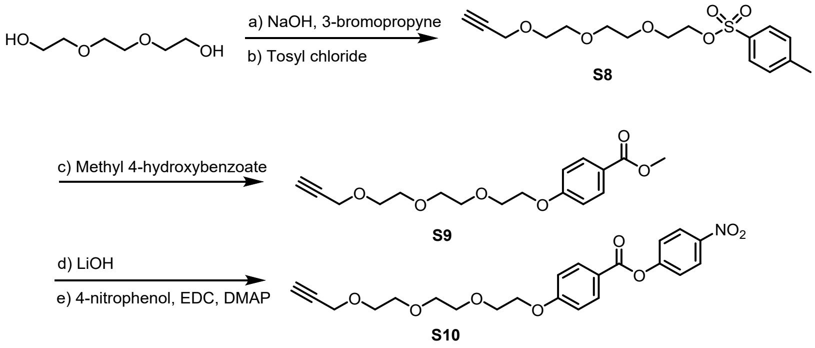

To a round-bottomed flask was added 2,2'-(ethane-1,2-diylbis(oxy))bis(ethan-1-ol) (3.0 g, 20 mmol) dissolved in 100 mL THF, and cooled in an ice bath. NaOH (1.6 g, 40 mmol) was added in one portion, and 3-bromoprop-1-yne (2.4 g, 20 mmol) was added dropwise after 1h. The solution was allowed to warm to room temperature and stirred for 16 h. After reaction completion, the solvent was removed, and then 50 mL H_2O was added. The aqueous solution was neutralized with 1 M dilute HCl, and extracted with CH_2Cl_2 (3×30 mL). The organic phase was washed with saturated NaCl solution (2×20 mL) and dried over anhydrous Na_2SO_4. The crud product was purified by flash column chromatography using eluent solution Hexane/EtOAc (1:1). The purified intermediate was used directly for next step. The intermediate 2-(2-(2-(prop-2-yn-1-yloxy)ethoxy)ethoxy)ethan-1-ol was dissolved with Tosyl chloride (TsCl, 3.8 g, 20 mmol) and Et_3N (2.0g, 20 mmol) in 100 mL CH_2Cl_2. The solution was allowed to stir for 2 h. The organic phase was washed with saturated NaCl solution (2×20 mL) and dried over anhydrous Na_2SO_4. The crud product was purified by flash column chromatography using eluent solution Hexane/EtOAc (5:1). **S8**, yellow oil, yield 75% for two steps. ^{1}H NMR (400 MHz, Chloroform-*d*) δ 7.78 – 7.70 (m, 2H), 7.34 – 7.27 (m, 2H), 4.13 (d, J = 2.4 Hz, 2H), 4.12 – 4.09 (m, 2H), 3.67 – 3.60 (m, 4H), 3.60 – 3.55 (m, 2H), 3.53 (s, 4H), 2.39 (s, 3H), 1.98 (s, 1H). ^{13}C NMR (101 MHz, Chloroform-*d*) δ 144.80, 132.93, 129.81, 127.92, 79.61, 74.60, 70.65, 70.48, 70.36, 69.26, 69.02, 68.61, 58.32, 21.59.

The intermediate **S8** (6.6g, 10 mmol), methyl 4-hydroxybenzoate (1.4 g, 10 mmol) and addition Et_3N (1.0 g, 10 mmol) were added in a round-bottomed flask with 100 mL CH_2Cl_2 as solvent, and the reaction was stirred and continued for 16 h. After reaction completion, the solvent was removed, and then 50 mL H_2O was added. The crud mixture was extracted with CH_2Cl_2 (3×20 mL). The organic phase was washed with saturated NaCl solution (2×10 mL) and dried over anhydrous Na_2SO_4. The crud product was purified by flash column chromatography using eluent solution Hexane/EtOAc (5:1). **S9**, yellow oil, yield 84%. ^{1}H NMR (500 MHz, Chloroform-*d*) δ 7.99 – 7.93 (m, 2H), 6.94 – 6.88 (m, 2H), 4.20 – 4.14

(m, 4H), 3.86 (d, J = 4.3 Hz, 5H), 3.75 – 3.69 (m, 2H), 3.69 – 3.63 (m, 6H), 2.42 (t, J = 2.4 Hz, 1H).

To a round-bottomed flask was added intermediate **S9** (1.6 g, 5 mmol) dissolved in 50 mL methanol, and cooled in an ice bath. An aqueous solution of LiOH (50 mL, 1 M) was added dropwise. The solution was allowed to warm to room temperature and stirred for 1 h. After reaction completion, the organic solvent was removed, and the aqueous solution was neutralized with 1 M dilute HCl, and extracted with CH_2Cl_2 (3×30 mL). The organic phase was washed with saturated NaCl solution (2×20 mL) and dried over anhydrous Na_2SO_4. The solvent was removed under reduced pressure, and the crude intermediate was used directly for next step. Under an ice bath, to a round-bottomed flask was added the crude intermediate, 4-nitrophenol (0.83 g, 5 mmol), 1-(3-dimethylaminopropyl)-3-ethylcarbodiimide hydrochloride (EDC, 1.9 g, 10 mmol) and 4-dimethylaminopyridine (DMAP, 0.06 g, 10% mol) dissolved in 50 mL CH_2Cl_2. The solution was allowed to warm to room temperature and stir for 16 h. The organic phase was washed with saturated NaCl solution (2×20 mL) and dried over anhydrous Na_2SO_4. The crud product was purified by flash column chromatography using eluent solution Hexane/EtOAc (5:1). **S10**, yellow oil, yield 71% for two steps. ^{1}H NMR (400 MHz, Chloroform-d) δ 8.32 – 8.23 (m, 2H), 8.15 – 8.07 (m, 2H), 7.46 – 7.37 (m, 2H), 7.06 – 6.98 (m, 2H), 4.27 – 4.21 (m, 2H), 4.19 (d, J = 2.4 Hz, 2H), 3.94 – 3.87 (m, 2H), 3.78 – 3.66 (m, 8H), 2.52 (t, J = 2.4 Hz, 1H).

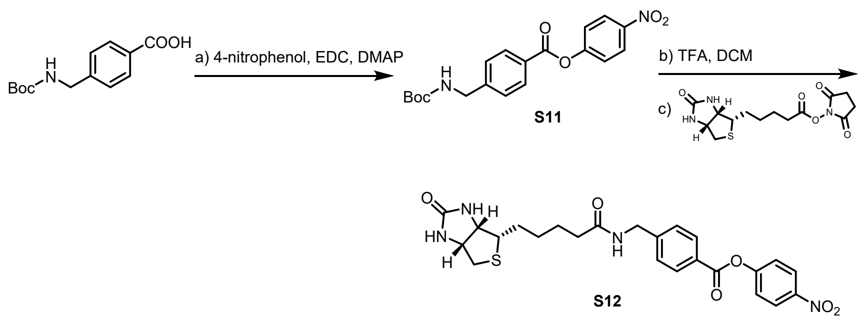

Under an ice bath, to a round-bottomed flask was added the 4-(((tert-butoxycarbonyl)amino)methyl)benzoic acid (2.5 g, 10 mmol), 4-nitrophenol (1.7 g, 10 mmol), EDC (3.8 g, 20 mmol) and 4-dimethylaminopyridine (DMAP, 0.12 g, 10% mol) dissolved in 100 mL CH_2Cl_2. The solution was allowed to warm to room temperature and stir for 16 h. The organic phase was washed with saturated NaCl solution (2×20 mL) and dried over anhydrous Na_2SO_4. The crud product was purified by flash column chromatography using eluent solution Hexane/EtOAc (2:1). **S11**, white powder, yield 86%. ^{1}H NMR (400 MHz, Chloroform-d) δ 8.32 – 8.24 (m, 2H), 8.16 – 8.09 (m, 2H), 7.45 – 7.35 (m, 4H), 5.18 (t, J = 6.3 Hz, 1H), 4.40 (d, J = 6.2 Hz, 2H), 1.45 (s, 9H). ^{13}C NMR (101 MHz, Chloroform-d) δ 164.15, 155.86, 146.21, 145.50, 130.78, 127.56, 126.16, 125.38, 122.77, 115.68, 80.05, 44.42, 28.51.

To a round-bottomed flask was added intermediate **S11** (1.9 g, 5 mmol) dissolved in 100 mL of 1/1 mixture of trifluoroacetate (TFA) and CH_2Cl_2. The solution was allowed to stir for 6 h. After reaction completion, the organic solvent was removed under reduced pressure, and neutralized with dilute $NaHCO_3$, and extracted with CH_2Cl_2 (3×30 mL). The organic phase was washed with saturated NaCl solution (2×20 mL) and dried over anhydrous Na_2SO_4. The solvent was removed under reduced pressure, and the crude intermediate was used directly for next step. To a round-bottomed flask was added the crude intermediate, Biotin-NHS ester (1.7 g, 5 mmol) dissolved in 100 mL CH_2Cl_2. The solution was allowed to warm to room temperature and stir for 16 h. The organic phase was washed with saturated NaCl solution (2×20 mL) and dried over anhydrous Na_2SO_4. The crud product was purified by flash column chromatography using eluent solution CH_2Cl_2/MeOH (20:1). **S12**, yellow solid, yield 57% for

two steps. ^{1}H NMR (400 MHz, Methanol-*d*$_4$) δ 8.00 – 7.83 (m, 4H), 7.59 – 7.51 (m, 4H), 4.51 (dd, *J* = 7.9, 4.8 Hz, 1H), 4.45 (s, 1H), 4.29 (dd, *J* = 7.9, 4.4 Hz, 1H), 4.20 (s, 2H), 3.38 (s, 2H), 3.21 (dt, *J* = 9.9, 5.3 Hz, 1H), 2.96 (dd, *J* = 12.8, 5.0 Hz, 1H), 2.73 (d, *J* = 12.7 Hz, 1H), 2.32 (t, *J* = 7.3 Hz, 2H), 1.78 – 1.56 (m, 4H), 1.45 (p, *J* = 7.8 Hz, 2H).

2.2.3. Synthesis of sulfoxonium ylide

The synthesis of sulfoxonium ylide was followed the similar procedure of previous reports.[7, 9] Generally, the acyl chloride or 4-nitrophenol ester was used as substrates. The synthesis of **1a** was described as an example.

To a round-bottomed flask was added trimethylsulfoxonium iodide (6.6 g, 30 mmol) and potassium t-butoxide (*t*-BuOK, 6.7 g, 60 mmol) dissolved in 200 mL dry tetrahydrofuran (THF) under nitrogen protection, and heated in an oil bath. The solution was allowed to reflux and stirred for 3 h. After reaction completion, the mixture was cooled in an ice bath. To this cooled solution, a solution of benzoyl chloride (1.4 g, 10 mmol) in 20 mL dry THF was added dropwise. After the addition, the solution was allowed to warm to room temperature and stirred for additional 6 h. After the reaction completion, the organic phase was washed with saturated NaCl solution (2×50 mL) and dried over anhydrous Na_2SO_4. The crud product was purified by flash column chromatography using eluent solution CH_2Cl_2/MeOH (20:1).

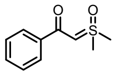

1a, white powder, yield 85%. The synthesis of sulfoxonium ylide was followed the similar procedure of previous reports.[7] The acyl chloride was used as substrates. ^{1}H NMR (500 MHz, Chloroform-*d*) δ 7.77 – 7.68 (m, 2H), 7.40 – 7.27 (m, 3H), 5.00 (s, 1H), 3.42 (s, 6H). ^{13}C NMR (126 MHz, Chloroform-*d*) δ 182.44, 138.98, 130.77, 128.21, 126.59, 69.30, 42.17. HRMS m/z (ESI-TOF): $[M+H]^+$ calcd for $C_{10}H_{13}O_2S$ 197.0631, found 197.0632.

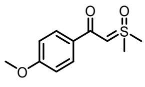

1b, white powder, yield 89%. The synthesis of sulfoxonium ylide was followed the similar procedure of previous reports.[9] The 4-nitrophenol ester **S5** was used as substrates. ^{1}H NMR (400 MHz, DMSO-*d*$_6$) δ 7.78 – 7.70 (m, 2H), 6.98 – 6.90 (m, 2H), 5.54 (s, 1H), 3.80 (s, 3H), 3.55 (s, 6H). ^{13}C NMR (101 MHz, DMSO-*d*$_6$) δ 180.42, 161.44, 132.50, 128.60, 113.67, 71.66, 55.71, 41.16. HRMS m/z (ESI-TOF): $[M+H]^+$ calcd for $C_{11}H_{15}O_3S$ 227.0736, found 227.0732.

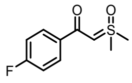

1c, white powder, yield 81%. The synthesis of sulfoxonium ylide was followed the similar procedure of previous reports.[9] The 4-nitrophenol ester **S6** was used as substrates. ^{1}H NMR (400 MHz, DMSO-*d*$_6$) δ 7.88 – 7.80 (m, 4H), 7.22 (t, *J* = 8.9 Hz, 4H), 5.63 (s, 2H), 3.58 (s, 11H), 2.52 (p, *J* = 1.9 Hz, 1H). ^{13}C NMR (101 MHz, DMSO-*d*$_6$) δ 179.47, 165.15, 162.69, 136.44, 136.41, 129.32, 129.23, 115.41, 115.20, 72.72, 41.03. ^{19}F NMR (376 MHz, DMSO-*d*$_6$) δ -111.12. HRMS m/z (ESI-TOF): $[M+H]^+$ calcd for $C_{10}H_{12}FO_2S$ 215.0537, found 215.0533.

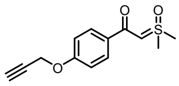

1d, yellow solid, yield 56%. The synthesis of sulfoxonium ylide was followed the similar procedure of previous reports.[7] The acyl chloride was used as substrates. ^{1}H NMR (400 MHz,

Chloroform-*d*) δ 7.80 – 7.71 (m, 2H), 6.99 – 6.91 (m, 2H), 4.91 (s, 1H), 4.71 (d, *J* = 2.4 Hz, 2H), 3.48 (s, 6H), 2.52 (t, *J* = 2.4 Hz, 1H). ^{13}C NMR (101 MHz, Chloroform-*d*) δ 181.79, 159.75, 132.58, 128.44, 114.44, 78.34, 75.95, 67.67, 55.94, 42.72. HRMS m/z (ESI-TOF): $[M+H]^+$ calcd for $C_{13}H_{15}O_3S$ 251.0736, found 251.0738.

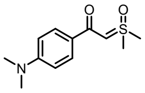

1e, yellow powder, yield 66%. The synthesis of sulfoxonium ylide was followed the similar procedure of previous reports.[7] The acyl chloride was used as substrates. ^{1}H NMR (400 MHz, Chloroform-*d*) δ 7.74 (d, *J* = 8.6 Hz, 2H), 6.72 – 6.64 (m, 2H), 4.93 (s, 1H), 3.53 (s, 6H), 3.04 (s, 6H). ^{13}C NMR (101 MHz, Chloroform-*d*) δ 182.52, 152.31, 128.20, 126.43, 111.02, 66.39, 42.86, 40.26. HRMS m/z (ESI-TOF): $[M+H]^+$ calcd for $C_{12}H_{18}NO_2S$ 240.1053, found 240.1052.

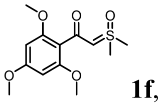

1f, white solid, yield 83%. The synthesis of sulfoxonium ylide was followed the similar procedure of previous reports.[9] The 4-nitrophenol ester **S8** was used as substrates. ^{1}H NMR (400 MHz, Chloroform-*d*) δ 6.07 (s, 2H), 4.52 (s, 1H), 3.78 (s, 3H), 3.75 (s, 6H), 3.48 (s, 6H). ^{13}C NMR (101 MHz, $CDCl_3$) δ 182.30, 161.12, 157.99, 114.47, 90.73, 73.11, 56.01, 55.39, 42.69. HRMS m/z (ESI-TOF): $[M+H]^+$ calcd for $C_{13}H_{19}O_5S$ 287.0948, found 287.0949.

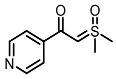

1g, yellow solid, yield 71%. The synthesis of sulfoxonium ylide was followed the similar procedure of previous reports.[7] The acyl chloride was used as substrates. ^{1}H NMR (400 MHz, Chloroform-*d*) δ 8.69 – 8.63 (m, 2H), 7.62 – 7.56 (m, 2H), 5.03 (s, 1H), 3.52 (s, 6H). ^{13}C NMR (101 MHz, $CDCl_3$) δ 179.74, 150.39, 145.86, 120.63, 70.21, 42.36. HRMS m/z (ESI-TOF): $[M+H]^+$ calcd for $C_9H_{12}NO_2S$ 198.0583, found 198.0584.

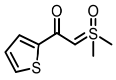

1h, yellow solid, yield 84%. The synthesis of sulfoxonium ylide was followed the similar procedure of previous reports.[9] The 4-nitrophenol ester **S7** was used as substrates. ^{1}H NMR (400 MHz, DMSO-d_6) δ 7.63 (dd, *J* = 5.0, 1.2 Hz, 1H), 7.48 (dd, *J* = 3.7, 1.2 Hz, 1H), 7.09 (dd, *J* = 5.0, 3.7 Hz, 1H), 5.54 (s, 1H), 3.57 (s, 6H). ^{13}C NMR (101 MHz, DMSO) δ 174.72, 147.57, 129.74, 128.24, 127.09, 71.85, 41.15. HRMS m/z (ESI-TOF): $[M+H]^+$ calcd for $C_8H_{11}O_2S_2$ 203.0195, found 203.0196.

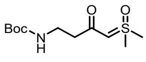

1i, yellow powder, yield 72%. The synthesis of sulfoxonium ylide was followed the similar procedure of previous reports.[9] The 4-nitrophenol ester **S9** was used as substrates. ^{1}H NMR (500 MHz, Chloroform-*d*) δ 5.24 (s, 1H), 4.43 (s, 1H), 3.37 (s, 6H), 3.32 (q, *J* = 6.1 Hz, 2H), 2.34 (t, *J* = 6.2 Hz, 2H), 1.39 (s, 9H). ^{13}C NMR (126 MHz, Chloroform-*d*) δ 189.17, 156.07, 79.01, 70.41, 42.22, 40.02, 37.33, 28.55. HRMS m/z (ESI-TOF): $[M+H]^+$ calcd for $C_{11}H_{22}NO_4S$ 264.1264, found 264.1266.

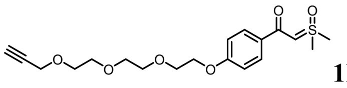

1l, yellow solid, yield 58%. The synthesis of sulfoxonium ylide was followed the similar procedure of previous reports.[9] The 4-nitrophenol ester **S12** was used as substrates. ^{1}H NMR (400 MHz, Chloroform-*d*) δ 7.75 – 7.67 (m, 2H), 6.91 – 6.82 (m, 2H), 4.90 (s, 1H), 4.16 (d, *J* = 2.4 Hz, 2H), 4.14 – 4.11 (m, 2H), 3.87 – 3.80 (m, 2H), 3.74 – 3.68 (m, 2H), 3.68 – 3.61 (m, 6H), 3.46 (s, 6H), 2.41 (t, *J* = 2.4 Hz, 1H). ^{13}C NMR (101 MHz, Chloroform-*d*) δ 181.93, 161.06, 131.82, 128.41, 114.11, 79.79, 74.75, 70.95, 70.76, 70.56, 69.77, 69.22, 67.70, 67.58, 58.52, 42.65. HRMS m/z (ESI-

TOF): $[M+H]^+$ calcd for $C_{19}H_{27}O_6S$ 383.1523, found 383.1517.

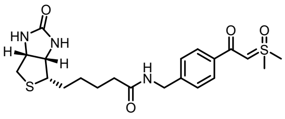

1m, yellow solid, yield 71%. The synthesis of sulfoxonium ylide was followed the similar procedure of previous reports.[9] The 4-nitrophenol ester **S14** was used as substrates. ^{1}H NMR (400 MHz, DMSO-d_6) δ 8.37 (t, J = 6.0 Hz, 1H), 7.74 – 7.66 (m, 2H), 7.28 – 7.21 (m, 2H), 6.41 (d, J = 26.1 Hz, 2H), 5.57 (s, 1H), 4.31 (d, J = 2.7 Hz, 1H), 4.13 (d, J = 5.4 Hz, 1H), 3.54 (s, 6H), 3.17 (d, J = 5.0 Hz, 2H), 3.09 (ddd, J = 8.6, 6.1, 4.3 Hz, 1H), 2.83 (dd, J = 12.4, 5.0 Hz, 1H), 2.58 (d, J = 12.4 Hz, 1H), 2.15 (t, J = 7.4 Hz, 2H), 1.67 – 1.45 (m, 4H), 1.39 – 1.27 (m, 2H). ^{13}C NMR (101 MHz, DMSO-d_6) δ 180.69, 172.74, 163.34, 142.58, 138.58, 127.27, 126.96, 72.62, 61.65, 59.80, 56.04, 49.20, 42.32, 41.17, 35.75, 28.83, 28.63, 25.91. HRMS m/z (ESI-TOF): $[M+H]^+$ calcd for $C_{21}H_{30}N_3O_4S_2$ 452.1672, found 452.1681.

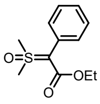

S1, white solid, yield 65%. The synthesis of sulfoxonium ylide was followed the similar procedure of previous reports.[7] ^{1}H NMR (400 MHz, Chloroform-d) δ 7.32 (d, J = 4.6 Hz, 5H), 4.11 (q, J = 7.2 Hz, 2H), 3.42 (s, 6H), 1.19 (t, J = 7.1 Hz, 3H). ^{13}C NMR (101 MHz, $CDCl_3$) δ 166.35, 133.61, 132.53, 128.44, 127.07, 70.38, 59.00, 43.26, 14.81. HRMS m/z (ESI-TOF): $[M+H]^+$ calcd for $C_{12}H_{17}O_3S$ 241.0893, found 241.0893.

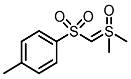

S2, colorless solid, yield 89%. The synthesis of sulfoxonium ylide was followed the similar procedure of previous reports.[10] The Tosyl fluoride was used as substrates. ^{1}H NMR (400 MHz, DMSO-d_6) δ 7.77 – 7.69 (m, 2H), 7.32 (d, J = 8.0 Hz, 2H), 4.52 (s, 1H), 3.50 (s, 6H), 2.37 (s, 3H). ^{13}C NMR (101 MHz, DMSO-d_6) δ 145.83, 141.46, 129.59, 125.76, 62.11, 43.15, 21.38. HRMS m/z (ESI-TOF): $[M+H]^+$ calcd for $C_{10}H_{15}O_3S_2$ 247.0457, found 247.0460.

2.2.4. Synthesis of sulfoxonium ylide 1j and 1k

The methylation of **1g** and **1h** was followed a same procedure. The synthesis of **1j** was described as an example. To a round-bottomed flask was charged with **1g** (0.24 g, 1 mmol). The flask was sealed and refiled with nitrogen for 3 times. CH_2Cl_2 was added by a syringe, and the flask was cooled in an ice bath. Methyl trifluoromethanesulfonate (MeOTf, 0.25 g, 1.5 mmol) was added dropwise. The reaction solution was allowed to stir for 1 h at room temperature, and white was precipitated. The product was filtered and washed with ether and CH_2Cl_2. The obtained powder was dried under vacuum, and the pure pyridinium salt **1j** was provided.

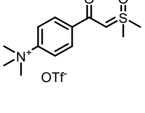

1j, white solid, yield 62%. ^{1}H NMR (400 MHz, DMSO-d_6) δ 8.05 – 7.94 (m, 4H), 5.78 (s, 1H), 3.63 (s, 9H), 3.60 (s, 6H). ^{13}C NMR (101 MHz, DMSO-d_6) δ 178.32, 148.76, 141.19, 128.27, 120.83, 73.92, 56.89, 40.98. ^{19}F NMR (376 MHz, DMSO-d_6) δ -77.73. HRMS m/z (ESI-TOF): $[M]^+$ calcd for $C_{13}H_{20}NO_2S$ 254.1209, found 254.1208.

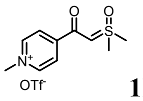

1k, yellow oil, yield 76%. ^{1}H NMR (400 MHz, DMSO-d_6) δ 9.04 (d, *J* = 6.5 Hz, 2H), 8.37 – 8.30 (m, 2H), 6.14 (s, 1H), 4.36 (s, 3H), 3.66 (s, 6H). ^{13}C NMR (101 MHz, DMSO-d_6) δ 173.23, 152.99, 146.63, 124.52, 77.89, 48.12, 40.62. ^{19}F NMR (376 MHz, DMSO-d_6) δ -77.74. HRMS m/z (ESI-TOF): $[M]^+$ calcd for $C_{10}H_{14}NO_2S$ 212.0740, found 212.0741.

2.3. General procedure A for the thiol-sulfoxonium ylide reaction

To a glass flask was added sulfhydryl compound **2** (0.5 mmol), sulfoxonium ylide **1** (1 mmol), RFTA (2% mol) and 8 mL water. The flask was then sealed and equipped with magnetic bar. The reaction was stirred and irradiated with 450 nm LED light (18 W) for 60 min. The resulting solution was then purified directly *via* preparative HPLC after filtration. Desired distillates were identified by MS and lyophilized to obtain target products.

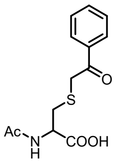

2a, white powder, yield 81%. ^{1}H NMR (500 MHz, DMSO-d_6) δ 12.82 (s, 1H), 8.25 (d, *J* = 8.2 Hz, 1H), 7.97 (d, *J* = 7.4 Hz, 2H), 7.64 (t, *J* = 7.3 Hz, 1H), 7.52 (t, *J* = 7.7 Hz, 2H), 4.42 (td, *J* = 8.6, 5.0 Hz, 1H), 4.13 (d, *J* = 15.0 Hz, 1H), 4.02 (d, *J* = 15.1 Hz, 1H), 2.91 (dd, *J* = 13.6, 4.9 Hz, 1H), 2.73 (dd, *J* = 13.6, 8.8 Hz, 1H), 1.83 (s, 3H). ^{13}C NMR (126 MHz, DMSO-d_6) δ 194.93, 172.27, 169.48, 135.37, 133.59, 128.89, 128.72, 51.62, 37.60, 33.07, 22.50. HRMS m/z (ESI-TOF): $[M-H]^-$ calcd for $C_{13}H_{14}NO_4S$ 280.0649, found 280.0643.

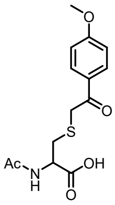

2b, white powder, yield 65%. ^{1}H NMR (400 MHz, DMSO-d_6) δ 8.28 (d, *J* = 8.1 Hz, 1H), 8.00 – 7.92 (m, 2H), 7.09 – 7.00 (m, 2H), 4.43 (td, *J* = 8.6, 4.9 Hz, 1H), 4.07 (d, *J* = 14.8 Hz, 1H), 3.96 (d, *J* = 14.8 Hz, 1H), 3.84 (s, 3H), 2.92 (dd, *J* = 13.6, 4.9 Hz, 1H), 2.74 (dd, *J* = 13.5, 8.9 Hz, 1H), 1.85 (s, 3H). ^{13}C NMR (101 MHz, DMSO-d_6) δ 193.44, 172.25, 169.46, 163.41, 131.06, 128.06, 114.02, 55.66, 51.51, 37.18, 32.96, 22.42. HRMS m/z (ESI-TOF): $[M+H]^+$ calcd for $C_{14}H_{18}NO_5S$ 312.0900, found 312.0897.

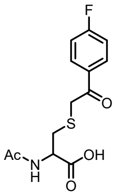

2c, white powder, yield 82%. ^{1}H NMR (400 MHz, DMSO-d_6) δ 8.20 (d, *J* = 8.1 Hz, 1H), 8.04 – 7.95 (m, 2H), 7.35 – 7.24 (m, 2H), 4.36 (td, *J* = 8.6, 4.9 Hz, 1H), 4.06 (d, *J* = 14.9 Hz, 1H), 3.95 (d, *J* = 15.0 Hz, 1H), 2.84 (dd, *J* = 13.6, 4.9 Hz, 1H), 2.66 (dd, *J* = 13.6, 8.9 Hz, 1H), 1.77 (s, 3H). ^{13}C NMR (101 MHz, DMSO-d_6) δ 193.49, 172.20, 169.45, 166.46, 163.95, 131.95, 131.92, 131.76, 131.66, 115.96, 115.74, 51.45, 37.37, 32.90, 22.40. ^{19}F NMR (376 MHz, DMSO-d_6) δ -74.10, -105.50, -105.52, -105.54. HRMS m/z (ESI-TOF): $[M+H]^+$ calcd for $C_{13}H_{15}FNO_4S$ 300.0700, found 300.0702.

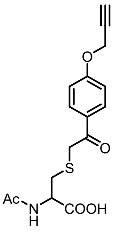
2d, white powder, yield 72%. ^{1}H NMR (400 MHz, DMSO-d_6) δ 8.28 (d, J = 8.1 Hz, 1H), 8.00 – 7.93 (m, 2H), 7.12 – 7.06 (m, 2H), 4.91 (d, J = 2.4 Hz, 2H), 4.43 (td, J = 8.5, 4.9 Hz, 1H), 4.07 (d, J = 14.8 Hz, 1H), 3.96 (d, J = 14.9 Hz, 1H), 3.63 – 3.62 (m, 1H), 2.96 – 2.86 (m, 1H), 2.78 – 2.68 (m, 1H), 1.85 (s, 3H). ^{13}C NMR (101 MHz, DMSO-d_6) δ 193.69, 172.48, 169.77, 161.48, 131.20, 128.88, 115.08, 79.08, 79.00, 56.02, 51.74, 37.43, 33.19, 22.65. HRMS m/z (ESI-TOF): $[M+H]^+$ calcd for $C_{16}H_{18}NO_5S$ 336.0900, found 336.0899.

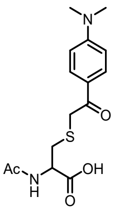
2e, white powder, yield 41%. ^{1}H NMR (400 MHz, Methanol-d_4) δ 7.95 – 7.87 (m, 2H), 6.82 – 6.74 (m, 2H), 4.68 (dd, J = 8.6, 4.6 Hz, 1H), 4.00 – 3.86 (m, 2H), 3.11 (s, 7H), 2.92 (dd, J = 13.9, 8.6 Hz, 1H), 2.03 (s, 3H). ^{13}C NMR (101 MHz, MeOD) δ 194.17, 172.36, 172.06, 154.09, 130.95, 122.81, 110.67, 51.94, 38.89, 36.54, 33.33, 21.05. HRMS m/z (ESI-TOF): $[M+H]^+$ calcd for $C_{15}H_{21}N_2O_4S$ 325.1217, found 325.1217.

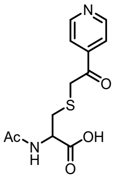
2g, colorless oil, yield 79%. ^{1}H NMR (400 MHz, Methanol-d_4) δ 8.76 (s, 2H), 7.90 (s, 2H), 4.61 (dd, J = 8.4, 4.6 Hz, 1H), 4.10 – 3.90 (m, 2H), 3.02 (d, J = 9.4 Hz, 1H), 2.84 – 2.78 (m, 1H), 1.95 (s, 3H). ^{13}C NMR (101 MHz, MeOD) δ 193.69, 172.02, 171.65, 149.94, 142.40, 122.42, 51.70, 32.98, 31.41, 21.05. HRMS m/z (ESI-TOF): $[M+H]^+$ calcd for $C_{12}H_{15}N_2O_4S$ 283.0747, found 283.0747.

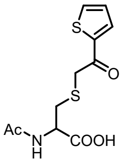
2h, yellow powder, yield 74%. ^{1}H NMR (400 MHz, DMSO-d_6) δ 8.31 (d, J = 8.1 Hz, 1H), 8.07 – 7.99 (m, 2H), 7.27 (dd, J = 4.9, 3.8 Hz, 1H), 4.46 (td, J = 8.6, 4.9 Hz, 1H), 4.04 (d, J = 14.8 Hz, 1H), 3.97 (d, J = 14.8 Hz, 1H), 2.99 (dd, J = 13.6, 5.0 Hz, 1H), 2.80 (dd, J = 13.5, 8.9 Hz, 1H), 1.87 (s, 3H). ^{13}C NMR (101 MHz, DMSO-d_6) δ 188.43, 172.23, 169.56, 142.36, 135.67, 134.44, 128.94, 51.57, 37.41, 33.22, 22.44. HRMS m/z (ESI-TOF): $[M+H]^+$ calcd for $C_{11}H_{14}NO_4S_2$ 288.0359, found 288.0357.

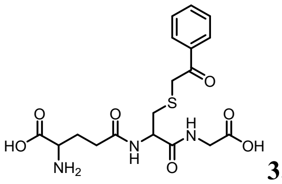
3a, white powder, yield 76%. ^{1}H NMR (500 MHz, DMSO-d_6) δ 8.46 (d, J = 8.2 Hz, 1H), 7.97 (dd, J = 12.7, 6.4 Hz, 3H), 7.62 (t, J = 7.3 Hz, 1H), 7.50 (t, J = 7.6 Hz, 2H), 4.49 (q, J = 8.7 Hz, 1H), 4.11 (d, J = 15.1 Hz, 1H), 4.03 (d, J = 15.1 Hz, 1H), 3.50 (ddd, J = 40.2, 17.1, 4.9 Hz, 2H),

3.40 (t, *J* = 6.1 Hz, 1H), 2.92 (dd, *J* = 13.2, 4.3 Hz, 1H), 2.68 (dd, *J* = 13.2, 9.8 Hz, 1H), 2.54 (s, 2H), 2.33 (hept, *J* = 7.9 Hz, 2H), 1.96 (q, *J* = 6.8 Hz, 2H). ^{13}C NMR (126 MHz, DMSO-d_6) δ 195.21, 172.48, 172.42, 171.72, 170.04, 159.36, 159.11, 158.86, 158.61, 135.48, 133.66, 128.96, 128.77, 120.83, 118.46, 116.08, 113.71, 53.62, 52.37, 40.43, 37.82, 33.84, 31.70, 27.07. HRMS m/z (ESI-TOF): $[M-H]^-$ calcd for $C_{18}H_{22}N_3O_7S$ 424.1184, found 424.1187.

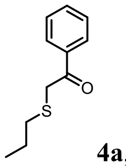

4a, yellow oil, yield 90%. The crud product was purified by flash column chromatography using eluent solution Hexane/EtOAc (15:1). ^{1}H NMR (400 MHz, Chloroform-*d*) δ 8.01 – 7.93 (m, 2H), 7.60 – 7.51 (m, 1H), 7.50 – 7.41 (m, 2H), 3.77 (s, 2H), 2.53 (td, *J* = 7.3, 0.8 Hz, 2H), 1.66 – 1.56 (m, 2H), 0.96 (td, *J* = 7.3, 0.8 Hz, 3H). ^{13}C NMR (101 MHz, Chloroform-*d*) δ 194.67, 135.28, 133.36, 128.85, 128.71, 37.07, 34.35, 22.34, 13.42. The NMR spectra are consistant with the reported literature.[7]

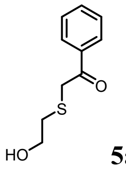

5a, yellow oil, yield 87%. The crud product was purified by flash column chromatography using eluent solution Hexane/EtOAc (5:1). ^{1}H NMR (400 MHz, Chloroform-*d*) δ 8.01 – 7.93 (m, 2H), 7.64 – 7.54 (m, 1H), 7.52 – 7.43 (m, 2H), 3.89 (s, 2H), 3.77 (t, *J* = 5.9 Hz, 2H), 2.77 (t, *J* = 5.9 Hz, 2H). ^{13}C NMR (101 MHz, Chloroform-*d*) δ 195.27, 135.10, 133.72, 128.83, 60.72, 37.15, 35.37. The NMR spectra are consistant with the reported literature.[11]

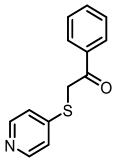

6a, yellow oil, yield 91%. The crud product was purified by flash column chromatography using eluent solution Hexane/EtOAc (5:1). ^{1}H NMR (400 MHz, Chloroform-*d*) δ 8.35 (dt, *J* = 4.6, 1.6 Hz, 2H), 7.96 (dt, *J* = 8.5, 1.3 Hz, 2H), 7.64 – 7.54 (m, 1H), 7.47 (td, *J* = 7.8, 1.6 Hz, 2H), 7.13 (dt, *J* = 4.6, 1.5 Hz, 2H), 4.40 (s, 2H). ^{13}C NMR (101 MHz, Chloroform-*d*) δ 192.93, 149.40, 147.56, 134.99, 134.09, 128.97, 128.68, 120.94, 38.04. HRMS m/z (ESI-TOF): $[M-H]^-$ calcd for $C_{13}H_{12}NOS$ 230.0634, found 230.0637.

3. Supplementary material and information for peptide modification

3.1. General information

All chemical reagents are commercially available from *Energy Chemical* without purification. Peptide **7**, **12**, **13**, **14**, Angiotensin II and Melanotan I were purchased from Wuxi Asiapeptide Biotechnology Co. Ltd.. The procedures for the preparation of peptide **8-11** were illustrated in the following section. **High-Resolution Mass Spectrometry (HRMS)** and **MS/MS** analysis were measured on a Q_Exactive_Focus. **Mass Spectrometry** (**MS**) to screen the molecular weight of HPLC fractions were carried out on SHIMAZU LC-MS 8030 in positive ion mode.

General procedure for SPPS

Peptide **8-11** were synthesized on Rink Amide MBHA resin by Fmoc solid-phase peptide synthesis (SPPS). Rink-amide resin was pre-swelled with DCM for 30min, filtered, the Fmoc (9-fluorenylmethyloxycarbonyl) group was removed with 50% (vol/vol) morpholine for 30min*2; the resin was sequentially washed with DCM and DMF for three times. Fmoc-protected amino acids (2.0 equiv.) and HATU (2.0 equiv.) were dissolved in DMF, followed by DIPEA (3.0 equiv.). The mixture was pre-activated for 1min and added to the resin for 1 h with N_2 bubbling, repeated once. The resin was washed sequentially with DCM, DMF for three times, then dried under a stream of nitrogen for next step. For cleavage of resin, the final resin was treated with TFA/TIS/water (95:2.5:2.5) at room temperature for 3 h and concentrated under a stream of nitrogen. The crude peptides were precipitated and washed with cold hexane/diethyl ether (1:2, v/v) at 4°C, redissolved in 50% acetonitrile in water. Crude peptides were purified by preparative HPLC.

General method for estimating the conversions and LC/MS analysis

LC-MS conversions were estimated by UV absorption at 220 nm of the peak corresponding to the sulfoxonium ylide adducted product versus the sum of all peptide peaks (also checked by MS): % conversion = A_p/A_{all}. A_p is the peak area of sulfoxonium ylide-adducted products; A_{all} is the peak area of all peptide peaks. The remaining substrates, disulfide by-products, oxidized by-products (MS+16) and other peptidic by-products were calculated. Note that, some of the peaks possess MS signals around the peptide substrates, but we couldn't clearly assign the by-reactions. Thus, we termed these peaks as other peptidic by-products. All of the conversions were reported as an average of three trials. Analytical LC-MS were performed on a Shimadzu LC-MS 8030 system equipped with Kromasil 100-5-C18 column (4.6 × 250 mm, 5 μm; room temperature) or Inertsil ODS-SP C18 column (4.6 × 250 mm, 5 μm; room temperature). Water (containing 0.1% TFA, A phase) and pure CH_3CN (B phase) were used as solvents in linear gradient mixtures at a flow rate of 1 $mL\cdot min^{-1}$.

General method for preparative LC

Preparative LC were performed on a Shimadzu LC-6AD system equipped with Shimadzu Shim-pack GIST C18 column (20 × 250 mm, 5 μm; room temperature). Water (containing 0.1% TFA, A phase) and pure CH_3CN (B phase) were used as solvents in linear gradient mixtures at a flow rate of 8 $mL\cdot min^{-1}$.

General method for MS/MS analysis

The fragmentation of modally modified peptides was investigated in positive electrospray ionization mode, loaded onto a Thermo Q Exactive Focus Orbitrap LC-MS/MS system. The protonated

molecule was generated by spraying a 0.5 ng/µl solution in 20:80 water:methanol + 0.1% formic acid (FA) with a flow rate of 0.28 mL/min. Parameters are as follows in Full MS/ data dependent-MS2 TopN mode: mass analyzer over m/z range of 145−2175 with a mass resolution of 70,000 (at m/z=200) in a data-dependent mode. MS/MS spectra were obtained using collision energy values at 25% normalized activation energy with a HCD (High Energy Collision Dissociation) mode.

3.2. General procedure B for the reaction of peptide

A 20 mM MeCN stock solution of RFTA was made up of 5.7 mg RFTA (0.01 mmol) and 0.5 mL MeCN, a 1 M water stock solution of thiourea was made up of 76 mg thiourea (1 mmol) and 1 mL deionized water, and a 100 mM water stock solution of sulfoxonium ylide was made up of specific sulfoxonium ylide (0.1 mmol) and 1 mL deionized water. These stock solutions were stored at room temperature away from light. To a 2 mL vial was added 200 uL specific peptide stock solution (1 mM, PB buffer, pH 7.4, 25% MeCN), 20 µL specific sulfoxonium ylide (100 mM), 2 µL thiourea (1 M) and 5 µL RFTA (20 mM) stock solution. The vial was then caped and equipped with magnetic bar. The reaction was stirred and irradiated with 450 nm LED light (40 W) for 1 min, as shown in the following figure. The resulting solution was then analyzed directly *via* HPLC after filtration. Desired distillates were identified by MS and lyophilized to obtain target products.

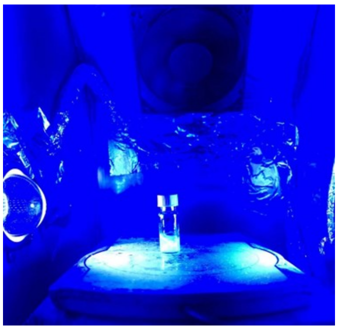

3.3. Characterization data for the reaction of peptide 7

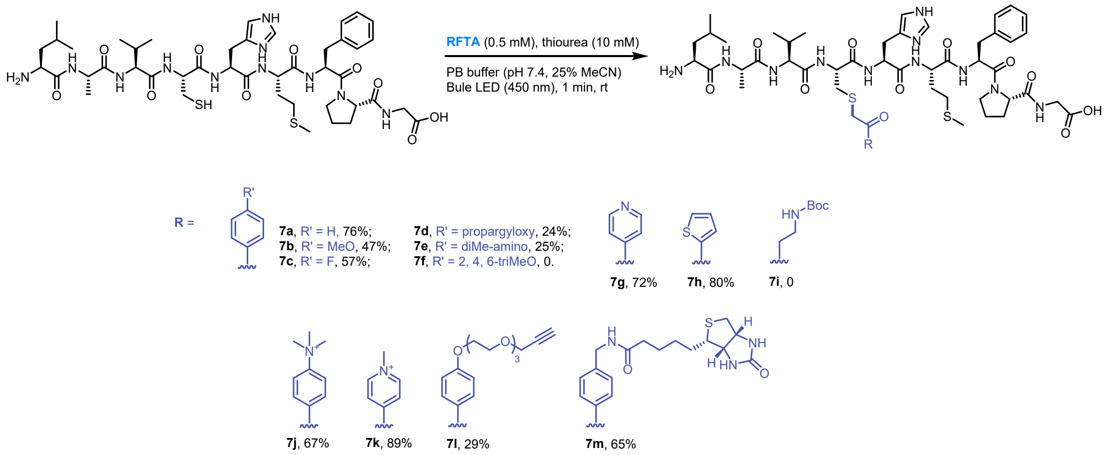

The reaction was followed **General procedure B** using peptide **7** (1 mM), sequence: NH_2-LAVCHMFPG-OH, and sulfoxonium ylide **1a-1m**. A stock solution of peptide **7** (1 mM) was made up by 5 mL PB buffer (pH 7.4, 25% MeCN) and 5 mg peptide **7**.

3.3.1. Characterization of 7a

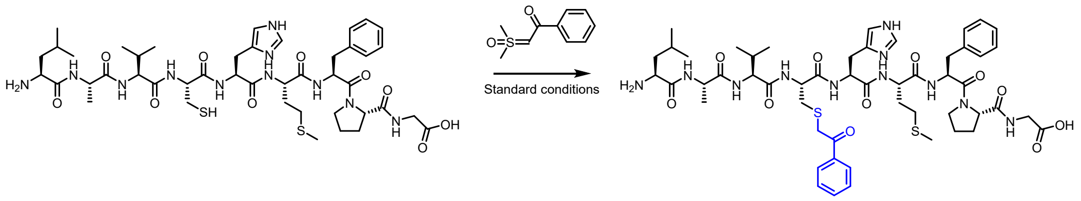

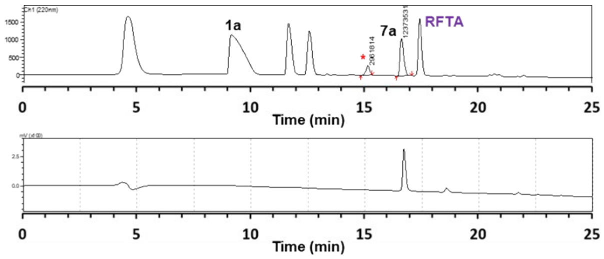

Peptide peak	Relative area
Substrate **7**	0
Target product **7a**	76%
By-product (disulfide) *	24%

LC trace of the reaction of peptide **7** and **1a** and purified product **7a**. (10-80% B phase over 25 min, 1 mL·min^{-1} flow rate, 0.1% TFA, λ = 220 nm, Inertsil ODS-SP 4.6 × 250 mm, 5 µm column). The table refer to the normalized integration of the peptide peaks.

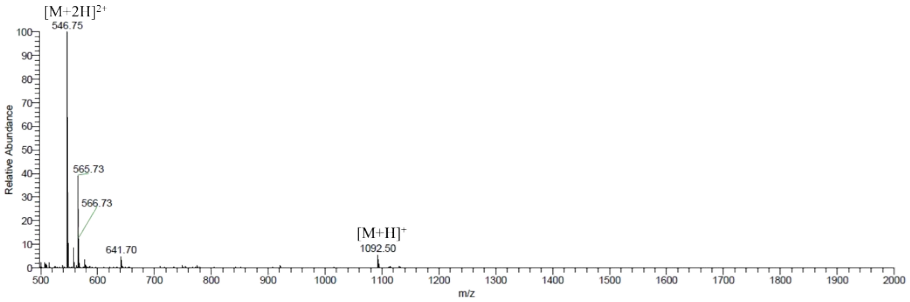

ESI Mass spectrum of purified product **7a**. Calculated Mass $[M+H]^+$: 1092.50; $[M+2H]^{2+}$: 546.75;

Mass Found (ESI+) $[M+H]^+$: 1092.50; $[M+2H]^{2+}$: 546.75.

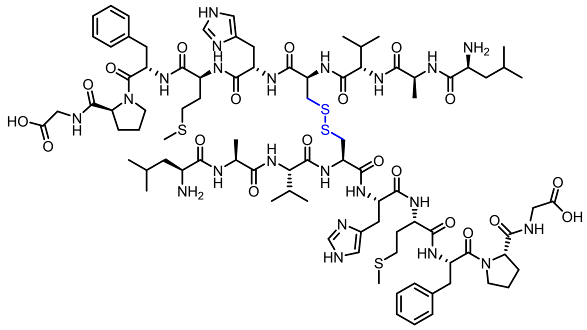

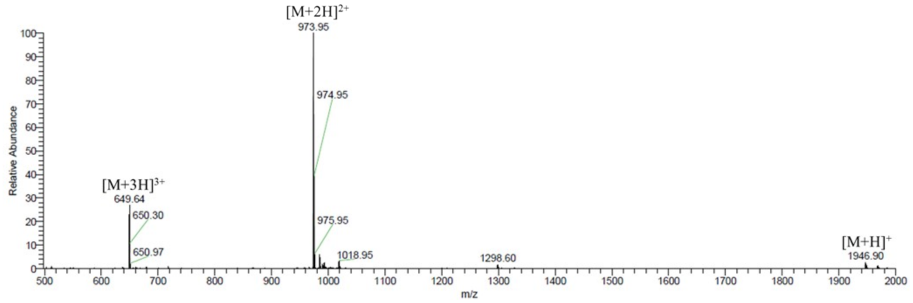

ESI Mass spectrum of oxidized by-product of **7** (disulfide). Calculated Mass $[M+H]^+$: 1945.90; $[M+2H]^{2+}$: 973.95; $[M+3H]^{3+}$: 649.64; Mass Found (ESI+) $[M+H]^+$: 1946.90; $[M+2H]^{2+}$: 973.95; $[M+3H]^{3+}$: 649.64.

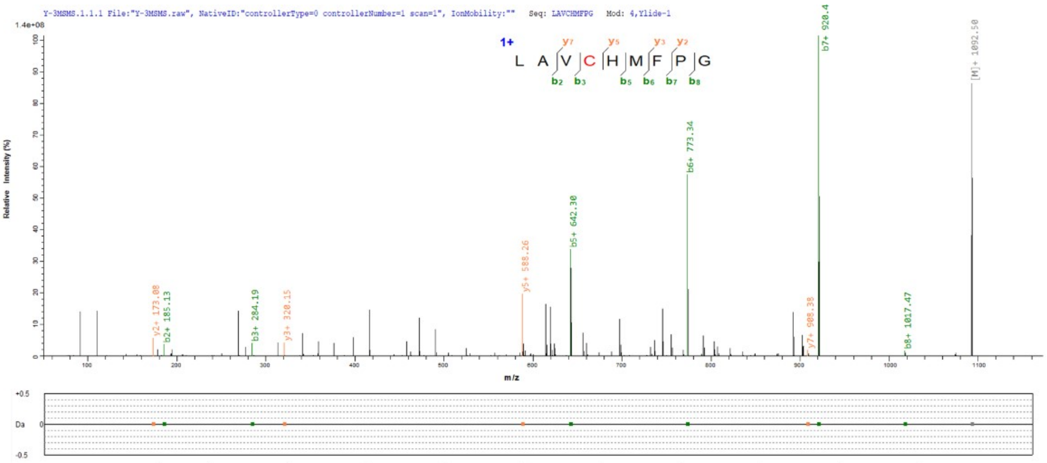

#	L(1/*)	A(2/8)	V(3/7)	C(4/6)	H(5/5)	M(6/4)	F(7/3)	P(8/2)	G(*/1)
b+	114.0913	185.1284	284.1969	505.2479	642.3068	773.3473	920.4157	1017.4685	*
b++	57.5493	93.0679	142.6021	253.1276	321.6571	387.1773	460.7115	509.2379	*
y+	*	979.4165	908.3793	809.3109	588.2599	451.2010	320.1605	173.0921	76.0393
y++	*	490.2119	454.6933	405.1591	294.6336	226.1041	160.5839	87.0497	38.5233

De novo ms/ms analysis of purified product **7a**.

3.3.2. Characterization of 7b

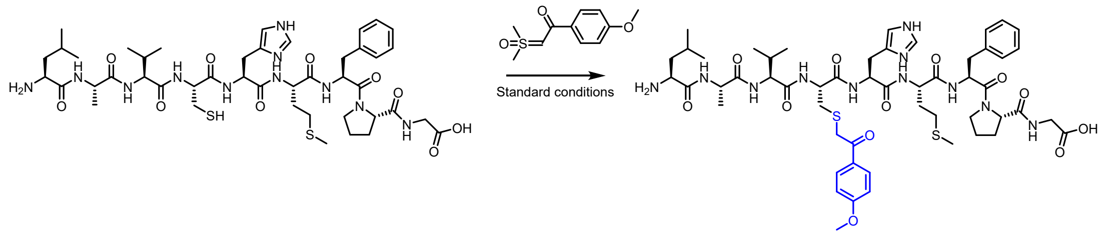

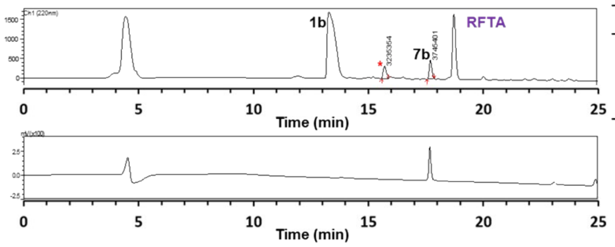

Peptide peak	Relative area
Substrate **7**	0
Target product **7b**	47%
By-product (disulfide) *	53%

LC trace of the reaction of peptide **7** and **1b** and purified product **7b**. (10-80% B phase over 25 min, 1 mL·min^{-1} flow rate, 0.1% TFA, λ = 220 nm, Kromasil 100-5-C18 4.6 × 250 mm, 5 µm column). The table refer to the normalized integration of the peptide peaks.

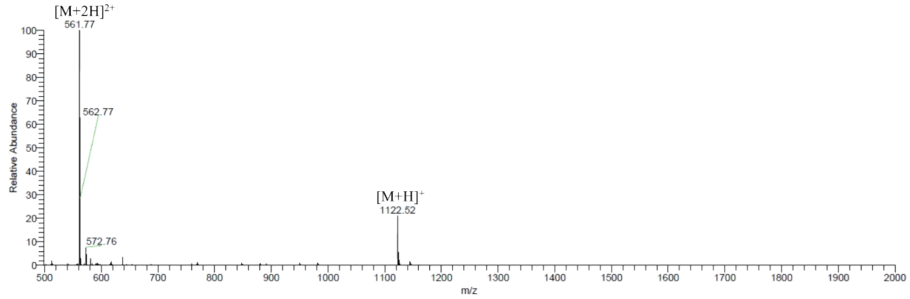

ESI Mass spectrum of purified product **7b**. Calculated Mass [M+H]$^{+}$: 1122.52; [M+2H]$^{2+}$: 561.77; Mass Found (ESI+) [M+H]$^{+}$: 1122.52; [M+2H]$^{2+}$: 561.77.

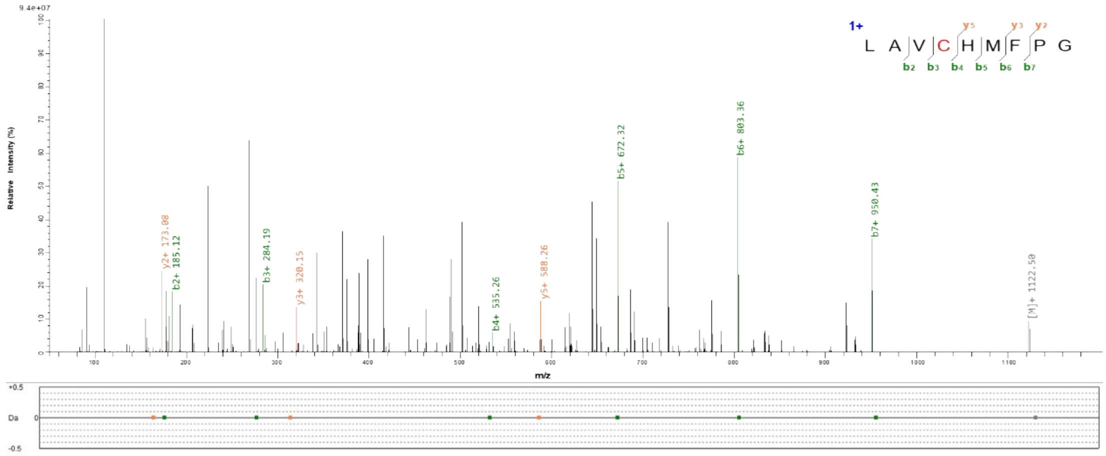

#	L(1/*)	A(2/8)	V(3/7)	C(4/6)	H(5/5)	M(6/4)	F(7/3)	P(8/2)	G(*/1)
b+	114.0913	185.1284	284.1969	535.2585	672.3174	803.3579	950.4263	1047.4790	*
b++	57.5493	93.0679	142.6021	268.1329	336.6623	402.1826	475.7168	524.2432	*
y+	*	1009.4270	938.3899	839.3215	588.2599	451.2010	320.1605	173.0921	76.0393
y++	*	505.2171	469.6986	420.1644	294.6336	226.1041	160.5839	87.0497	38.5233

De novo ms/ms analysis of purified product **7b**.

3.3.3. Characterization of 7c

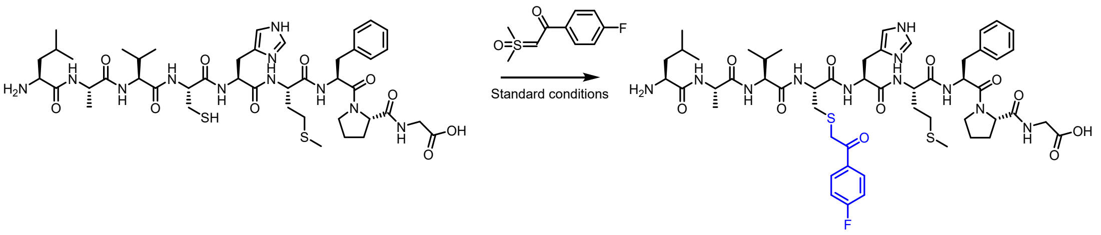

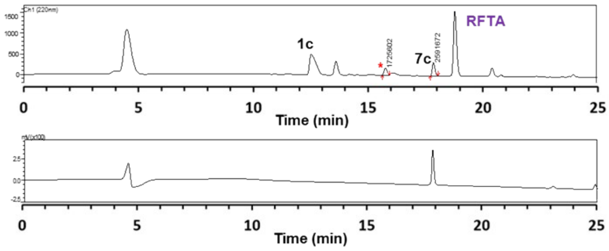

Peptide peak	Relative area
Substrate **7**	0
Target product **7c**	57%
By-product (disulfide) *	43%

LC trace of the reaction of peptide **7** and **1c** and purified product **7c**. (10-80% B phase over 25 min, 1 mL·min^{-1} flow rate, 0.1% TFA, λ = 220 nm, Kromasil 100-5-C18 4.6 × 250 mm, 5 µm column). The table refer to the normalized integration of the peptide peaks.

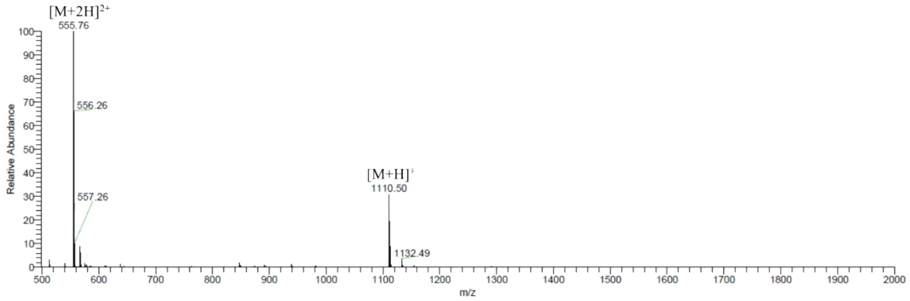

ESI Mass spectrum of purified product **7c**. Calculated Mass $[M+H]^+$: 1110.50; $[M+2H]^{2+}$: 555.76; Mass Found (ESI+) $[M+H]^+$: 1110.50; $[M+2H]^{2+}$: 555.76.

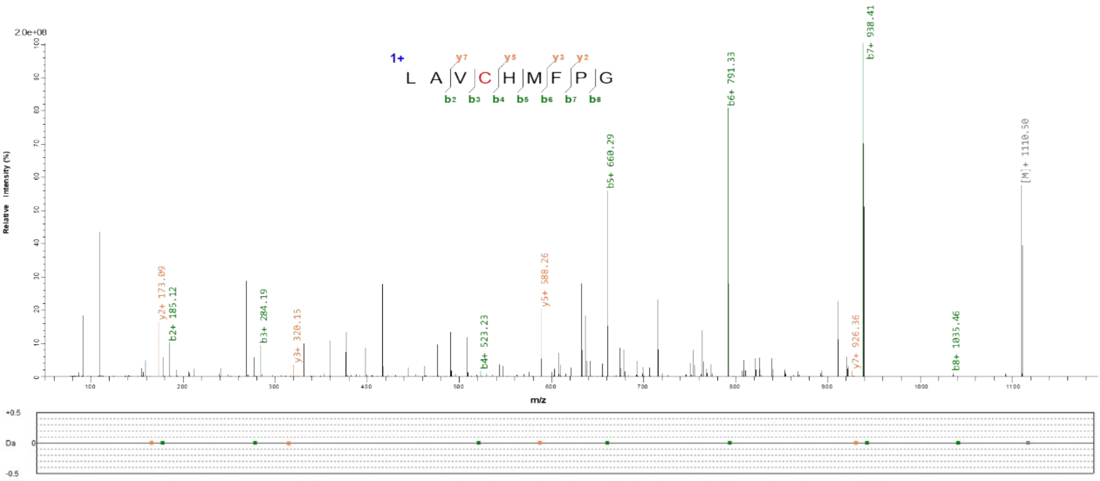

#	L(1/*)	A(2/8)	V(3/7)	C(4/6)	H(5/5)	M(6/4)	F(7/3)	P(8/2)	G(*/1)
b+	114.0913	185.1284	284.1969	523.2385	660.2974	791.3379	938.4063	1035.4590	*
b++	57.5493	93.0679	142.6021	262.1229	330.6523	396.1726	469.7068	518.2332	*
y+	*	997.4070	926.3699	827.3015	588.2599	451.2010	320.1605	173.0921	76.0393
y++	*	499.2071	463.6886	414.1544	294.6336	226.1041	160.5839	87.0497	38.5233

De novo ms/ms analysis of purified product **7c**.

3.3.4. Characterization of 7d

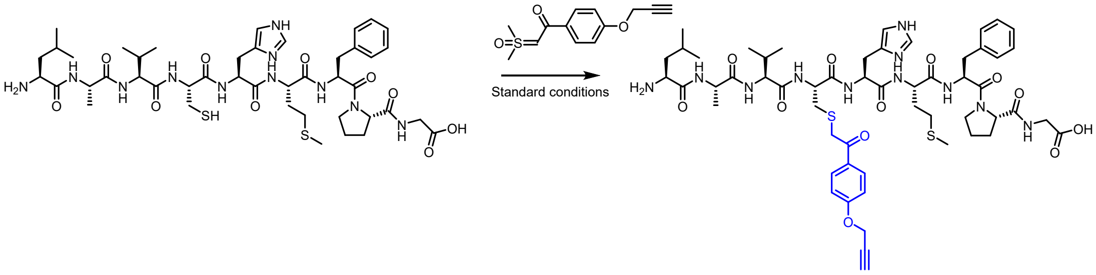

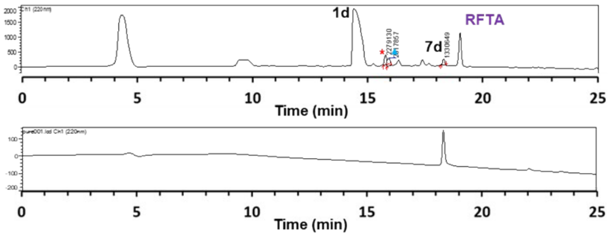

Peptide peak	Relative area
Substrate 7	0
Target product **7d**	24%
By-product (disulfide) *	40%
By-product (**7** MS+17) ▲	36%

LC trace of the reaction of peptide **7** and **1d** and purified product **7d**. (10-80% B phase over 25 min, 1 mL·min^{-1} flow rate, 0.1% TFA, λ = 220 nm, Kromasil 100-5-C18 4.6 × 250 mm, 5 µm column). The table refer to the normalized integration of the peptide peaks.

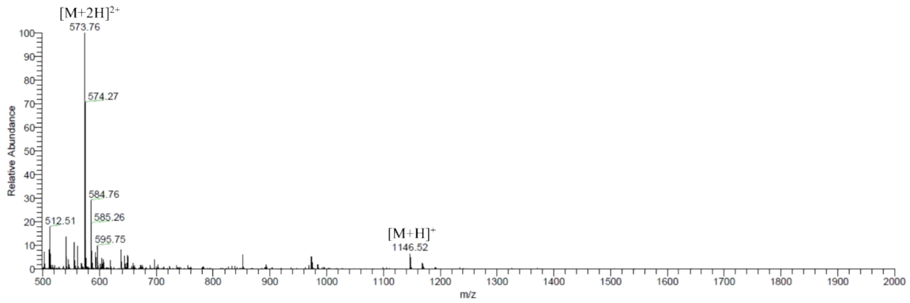

ESI Mass spectrum of purified product **7d**. Calculated Mass $[M+H]^+$: 1146.52; $[M+2H]^{2+}$: 573.76; Mass Found (ESI+) $[M+H]^+$: 1146.52; $[M+2H]^{2+}$: 573.76.

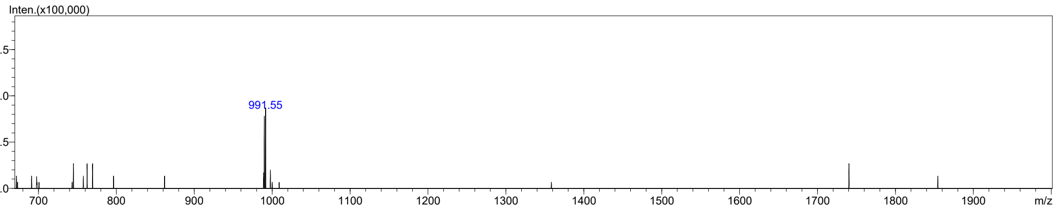

ESI Mass spectrum of by-product (**7** MS+17). Calculated Mass $[M+H]^+$: 991.45; Mass Found (ESI+) $[M+H]^+$: 991.55.

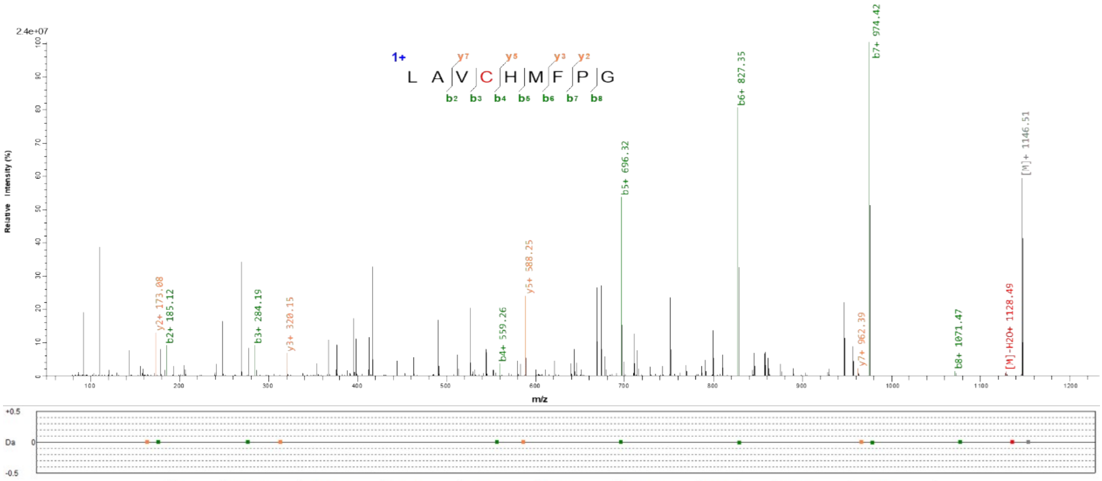

#	L(1/*)	A(2/8)	V(3/7)	C(4/6)	H(5/5)	M(6/4)	F(7/3)	P(8/2)	G(*/1)
b+	114.0913	185.1284	284.1969	559.2585	696.3174	827.3579	974.4263	1071.4790	*
b++	57.5493	93.0679	142.6021	280.1329	348.6623	414.1826	487.7168	536.2432	*
y+	*	1033.4270	962.3899	863.3215	588.2599	451.2010	320.1605	173.0921	76.0393
y++	*	517.2171	481.6986	432.1644	294.6336	226.1041	160.5839	87.0497	38.5233

De novo ms/ms analysis of purified product **7d**.

3.3.5. Characterization of 7e

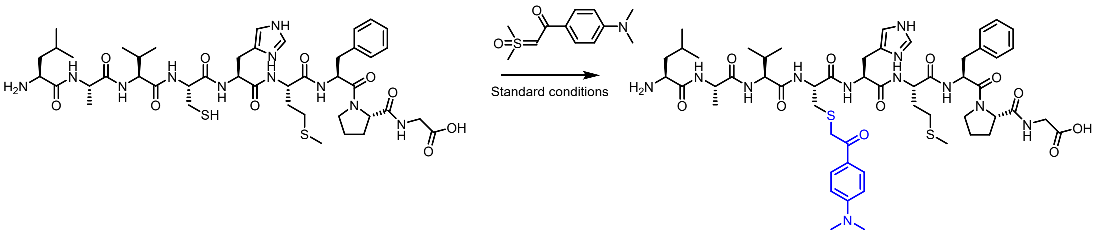

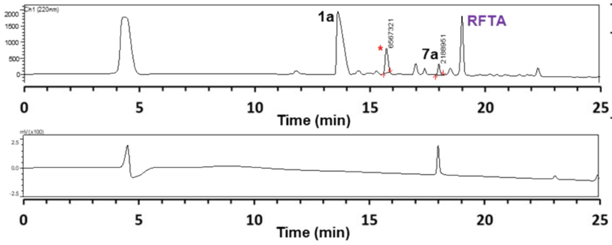

Peptide peak	Relative area
Substrate 7	0
Target product 7e	25%
By-product (disulfide) *	75%

LC trace of the reaction of peptide **7** and **1e** and purified product **7e**. (10-80% B phase over 25 min, 1 mL·min^{-1} flow rate, 0.1% TFA, λ = 220 nm, Kromasil 100-5-C18 4.6 × 250 mm, 5 µm column). The table refer to the normalized integration of the peptide peaks.

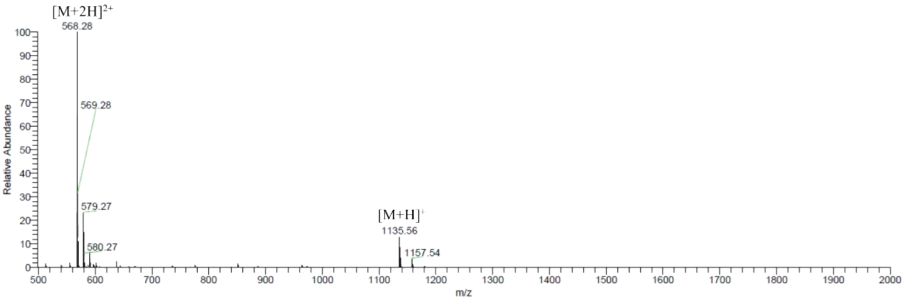

ESI Mass spectrum of purified product **7e**. Calculated Mass $[M+H]^+$: 1135.56; $[M+2H]^{2+}$: 568.28; Mass Found (ESI+) $[M+H]^+$: 1135.56; $[M+2H]^{2+}$: 568.28.

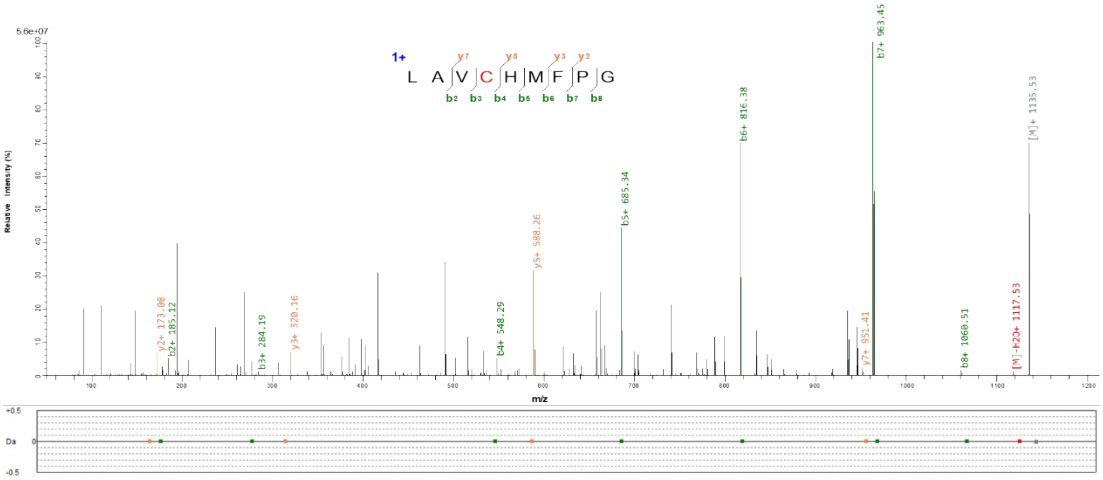

#	L(1/*)	A(2/8)	V(3/7)	C(4/6)	H(5/5)	M(6/4)	F(7/3)	P(8/2)	G(*/1)
b+	114.0913	185.1284	284.1969	548.2902	685.3491	816.3896	963.4580	1060.5107	*
b++	57.5493	93.0679	142.6021	274.6487	343.1782	408.6984	482.2326	530.7590	*
y+	*	1022.4587	951.4216	852.3532	588.2599	451.2010	320.1605	173.0921	76.0393
y++	*	511.7330	476.2144	426.6802	294.6336	226.1041	160.5839	87.0497	38.5233

De novo ms/ms analysis of purified product **7e**.

3.3.6. Characterization of 7f

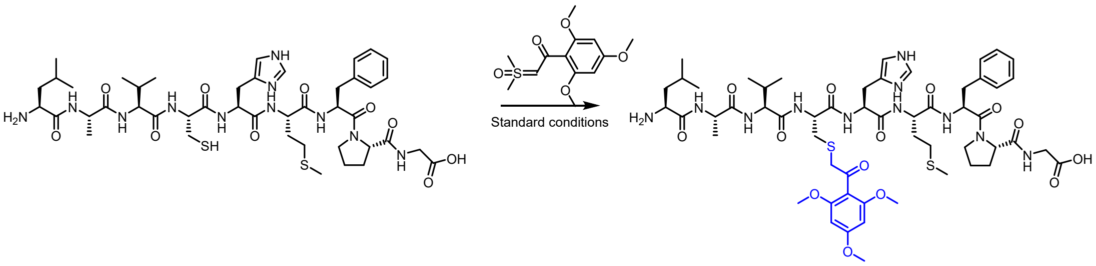

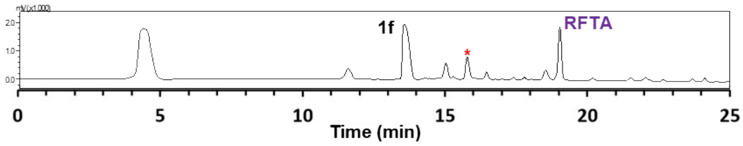

LC trace of the reaction of peptide **7** and **1f** and purified product **7f**. (10-80% B phase over 25 min, 1 mL·min^{-1} flow rate, 0.1% TFA, λ = 220 nm, Kromasil 100-5-C18 4.6 × 250 mm, 5 µm column).

3.3.7. Characterization of 7g

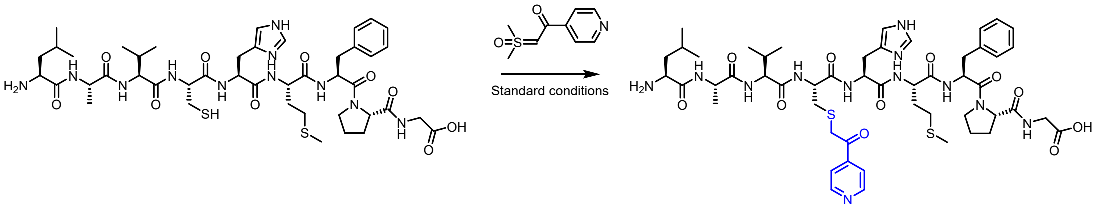

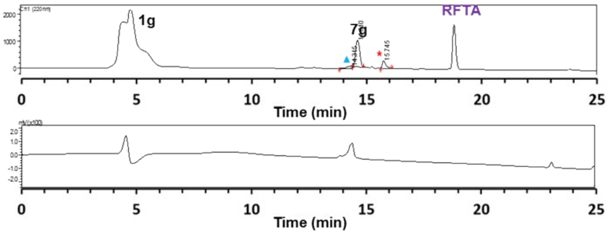

Peptide peak	Relative area
Substrate **7**	0
Target product **7g**	72%
By-product (disulfide) *	18%
By-product (**7g** MS+19) ▲	10%

LC trace of the reaction of peptide **7** and **1g** and purified product **7g**. (10-80% B phase over 25 min, 1 mL·min^{-1} flow rate, 0.1% TFA, λ = 220 nm, Kromasil 100-5-C18 4.6 × 250 mm, 5 μm column). The table refer to the normalized integration of the peptide peaks.

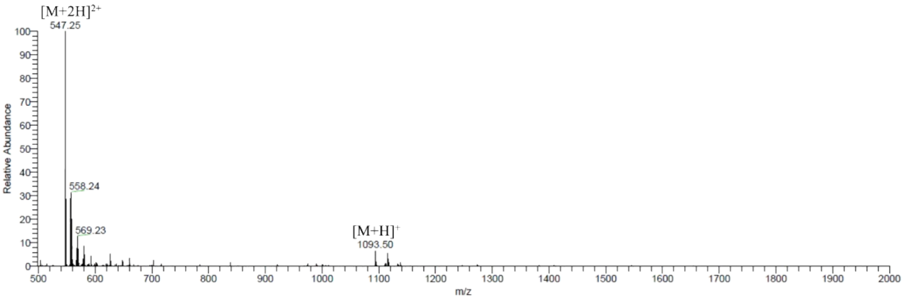

ESI Mass spectrum of purified product **7g**. Calculated Mass [M+H]$^+$: 1093.50; [M+2H]$^{2+}$: 547.25; Mass Found (ESI+) [M+H]$^+$: 1093.50; [M+2H]$^{2+}$: 547.25.

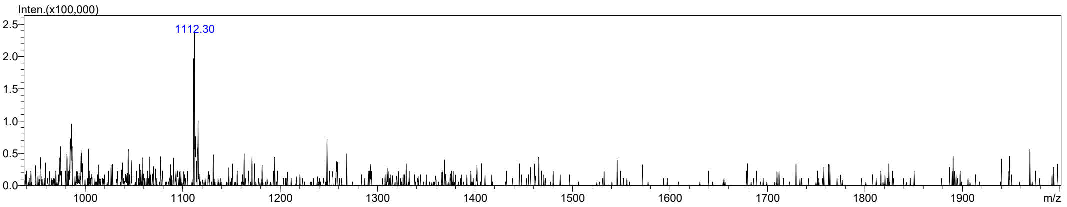

ESI Mass spectrum of by-product (**7g** MS+19). Calculated Mass [M+H]$^+$: 1112.50; Mass Found (ESI+) [M+H]$^+$: 1112.30.

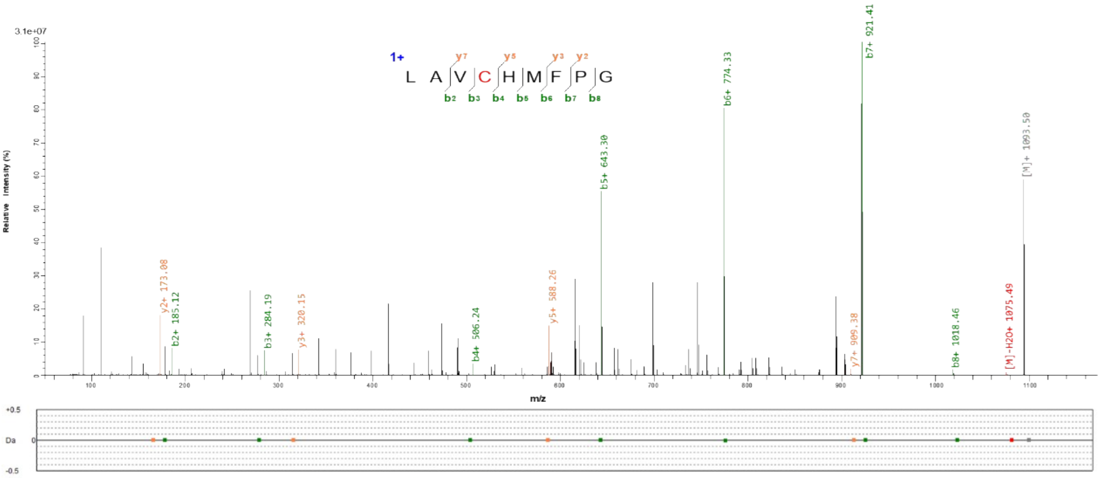

#	L(1/*)	A(2/8)	V(3/7)	C(4/6)	H(5/5)	M(6/4)	F(7/3)	P(8/2)	G(*/1)
b+	114.0913	185.1284	284.1969	506.2432	643.3021	774.3426	921.4110	1018.4637	*
b++	57.5493	93.0679	142.6021	253.6252	322.1547	387.6749	461.2091	509.7355	*
y+	*	980.4117	909.3746	810.3062	588.2599	451.2010	320.1605	173.0921	76.0393
y++	*	490.7095	455.1909	405.6567	294.6336	226.1041	160.5839	87.0497	38.5233

De novo ms/ms analysis of purified product **7g**.

3.3.8. Characterization of 7h

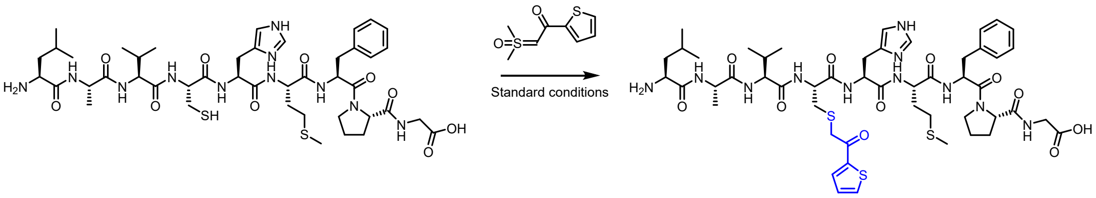

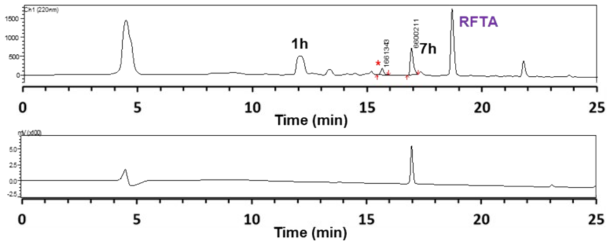

Peptide peak	Relative area
Substrate 7	0
Target product **7h**	80%
By-product (disulfide) *	20%

LC trace of the reaction of peptide **7** and **1h** and purified product **7h**. (10-80% B phase over 25 min, 1 mL·min^{-1} flow rate, 0.1% TFA, λ = 220 nm, Kromasil 100-5-C18 4.6 × 250 mm, 5 µm column). The table refer to the normalized integration of the peptide peaks.

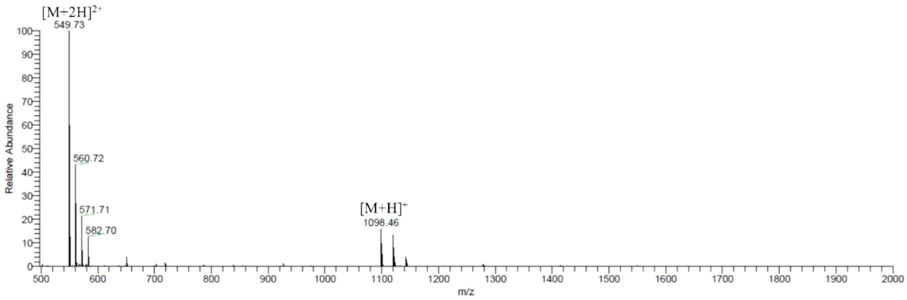

ESI Mass spectrum of purified product **7h**. Calculated Mass $[M+H]^+$: 1098.46; $[M+2H]^{2+}$: 549.73; Mass Found (ESI+) $[M+H]^+$: 1098.46; $[M+2H]^{2+}$: 549.73.

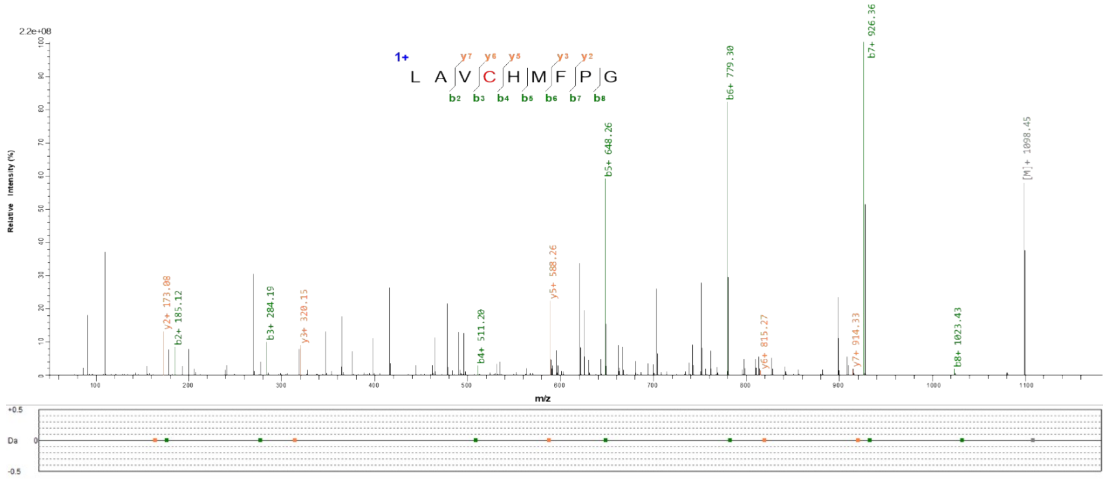

#	L(1/*)	A(2/8)	V(3/7)	C(4/6)	H(5/5)	M(6/4)	F(7/3)	P(8/2)	G(*/1)
b+	114.0913	185.1284	284.1969	511.2044	648.2633	779.3038	926.3722	1023.4249	*
b++	57.5493	93.0679	142.6021	256.1058	324.6353	390.1555	463.6897	512.2161	*
y+	*	985.3729	914.3358	815.2674	588.2599	451.2010	320.1605	173.0921	76.0393
y++	*	493.1901	457.6715	408.1373	294.6336	226.1041	160.5839	87.0497	38.5233

De novo ms/ms analysis of purified product **7h**.

3.3.9. Characterization of 7i

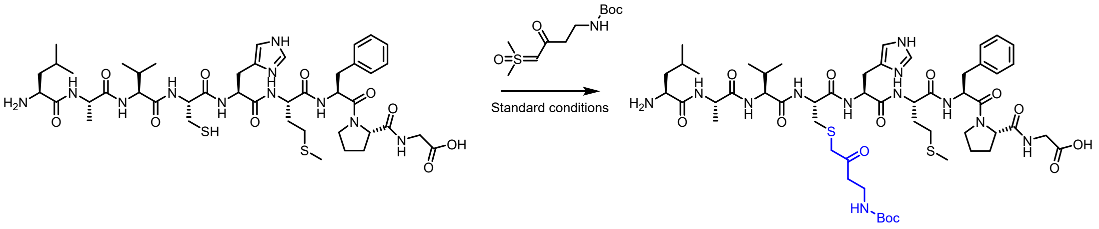

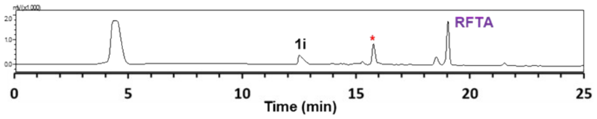

LC trace of the reaction of peptide **7** and **1i** and purified product **7i**. (10-80% B phase over 25 min, 1 mL·min^{-1} flow rate, 0.1% TFA, λ = 220 nm, Kromasil 100-5-C18 4.6 × 250 mm, 5 μm column).

3.3.10. Characterization of 7j

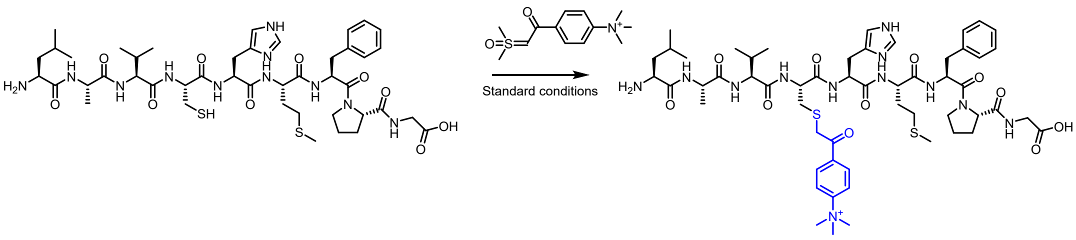

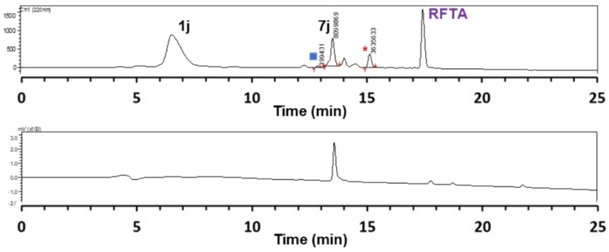

Peptide peak	Relative area
Substrate **7**	0
Target product **7j**	67%
By-product (disulfide) *	30%
Oxidized **7j** (MS+16) ■	3%

LC trace of the reaction of peptide **7** and **1j** and purified product **7j**. (10-80% B phase over 25 min, 1 mL·min^{-1} flow rate, 0.1% TFA, λ = 220 nm, Inertsil ODS-SP 4.6 × 250 mm, 5 μm column). The table refer to the normalized integration of the peptide peaks.

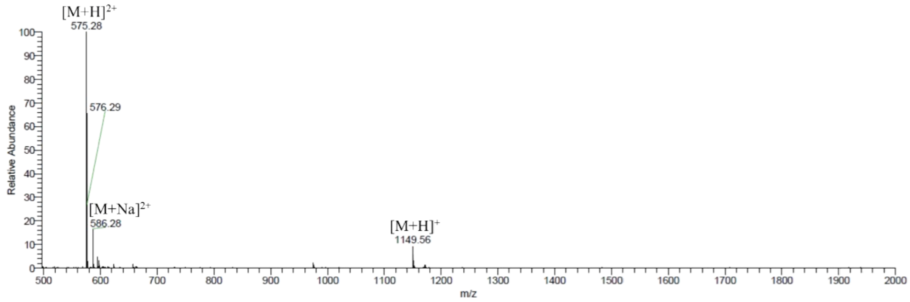

ESI Mass spectrum of purified product **7j**. Calculated Mass [M]$^{+}$: 1149.56; [M+H]$^{2+}$: 575.28; Mass Found (ESI+) [M]$^{+}$: 1149.56; [M+Na]$^{2+}$: 586.28; [M+H]$^{2+}$: 575.28.

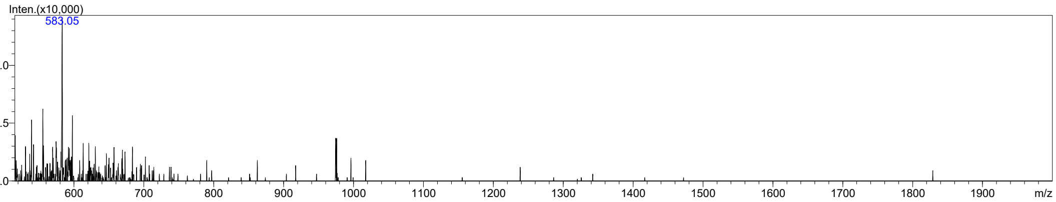

ESI Mass spectrum of Oxidized **7j** (MS+16). Calculated Mass [M]$^{+}$: 1165.56; [M+H]$^{2+}$: 583.28; Mass Found (ESI+) [M+H]$^{2+}$: 583.05.

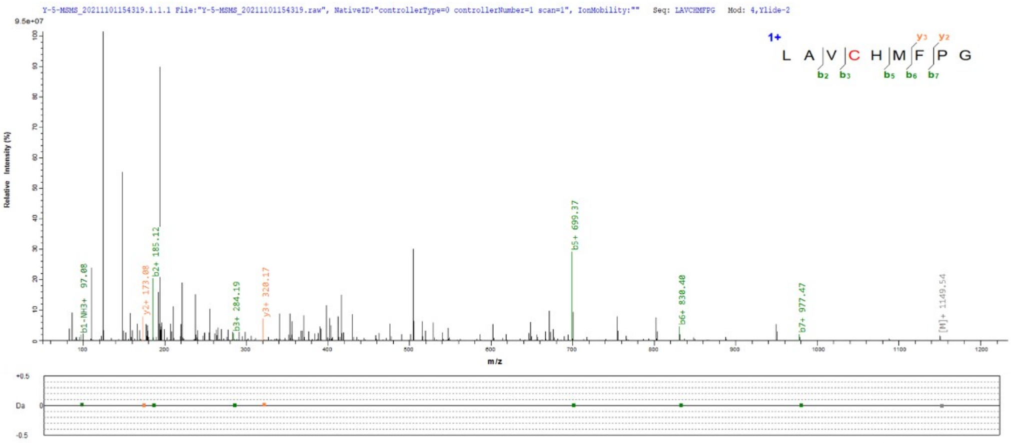

#	L(1/*)	A(2/8)	V(3/7)	C(4/6)	H(5/5)	M(6/4)	F(7/3)	P(8/2)	G(*/1)
b+	114.0913	185.1284	284.1969	562.3058	699.3647	830.4052	977.4736	1074.5263	*
b++	57.5493	93.0679	142.6021	281.6565	350.1860	415.7062	489.2404	537.7668	*
y+	*	1036.4743	965.4372	866.3688	588.2599	451.2010	320.1605	173.0921	76.0393
y++	*	518.7408	483.2222	433.6880	294.6336	226.1041	160.5839	87.0497	38.5233

De novo ms/ms analysis of purified product **7j**.

3.3.11. Characterization of 7k

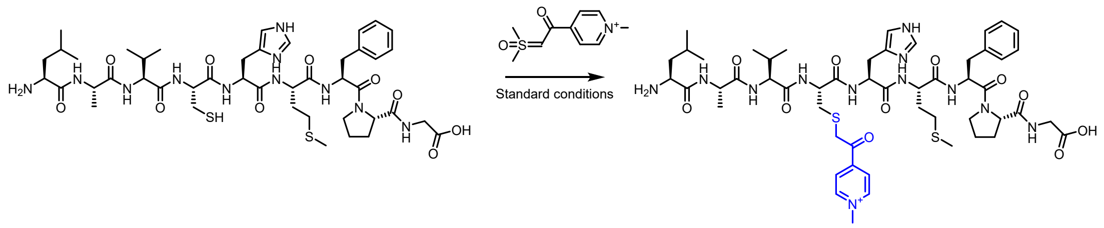

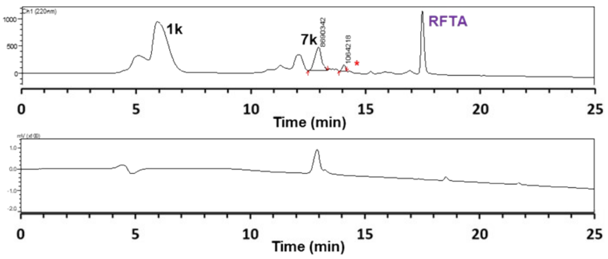

Peptide peak	Relative area
Substrate **7**	0
Target product **7k**	89%
By-product (disulfide) *	11%

LC trace of the reaction of peptide **7** and **1k** and purified product **7k**. (10-80% B phase over 25 min, 1 mL·min^{-1} flow rate, 0.1% TFA, λ = 220 nm, Inertsil ODS-SP 4.6 × 250 mm, 5 µm column). The table refer to the normalized integration of the peptide peaks.

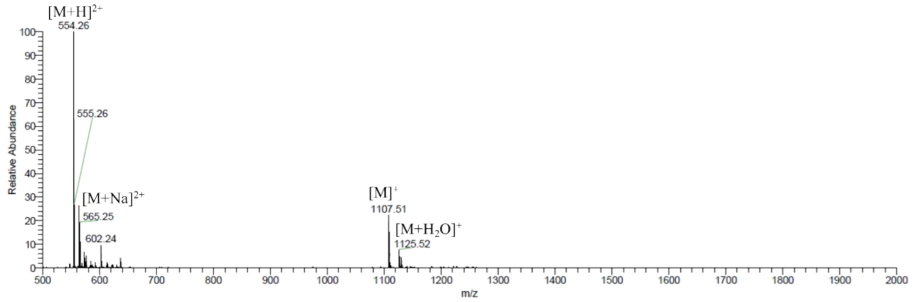

ESI Mass spectrum of purified product **7k**. Calculated Mass [M]$^{+}$: 1107.51; [M+H]$^{2+}$: 554.26; Mass Found (ESI+) [M]$^{+}$: 1107.51; [M+Na]$^{2+}$: 565.25; [M+H]$^{2+}$: 554.26.

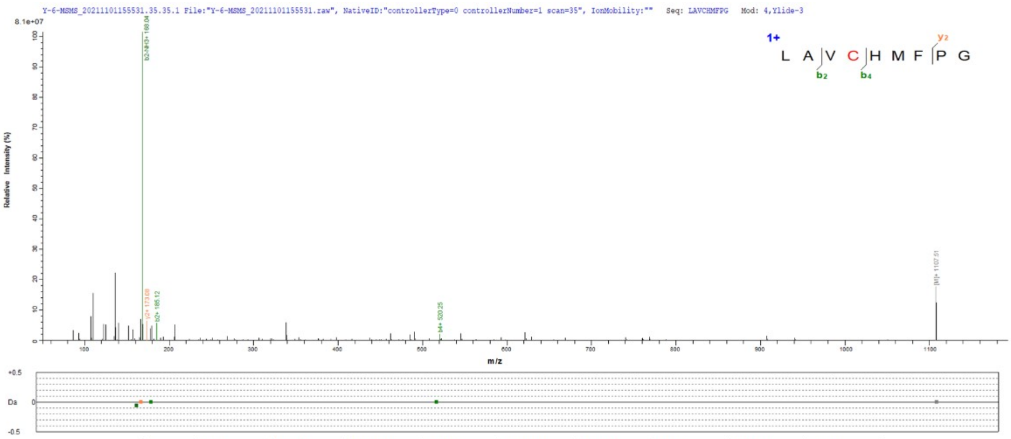

#	L(1/*)	A(2/8)	V(3/7)	C(4/6)	H(5/5)	M(6/4)	F(7/3)	P(8/2)	G(*/1)
b+	114.0913	185.1284	284.1969	520.2588	657.3177	788.3582	935.4266	1032.4794	*
b++	57.5493	93.0679	142.6021	260.6330	329.1625	394.6828	468.2170	516.7433	*
y+	*	994.4274	923.3902	824.3218	588.2599	451.2010	320.1605	173.0921	76.0393
y++	*	497.7173	462.1988	412.6646	294.6336	226.1041	160.5839	87.0497	38.5233

De novo ms/ms analysis of purified product **7k**

3.3.12. Characterization of 7l

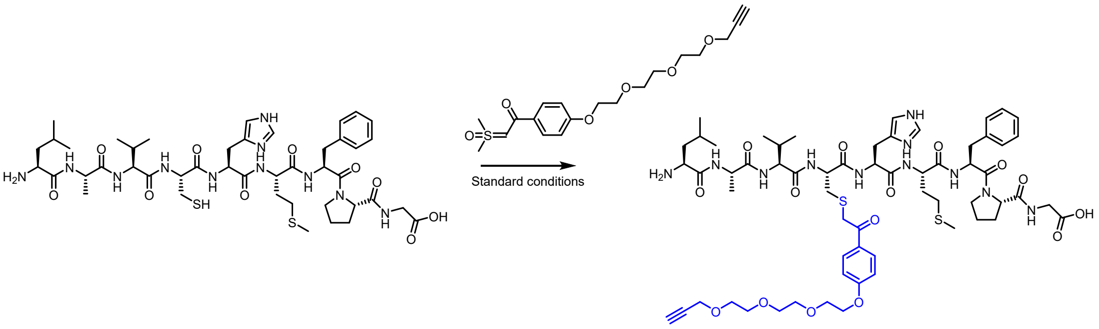

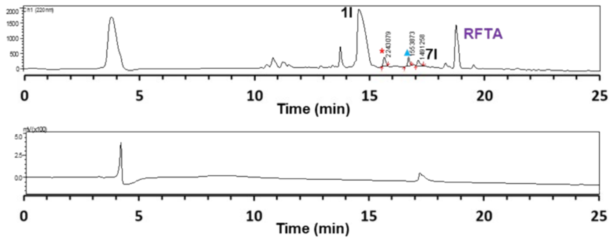

Peptide peak	Relative area
Substrate 7	0
Target product 7l	29%
By-product (disulfide) *	42%
By-product (7 MS+7) ▲	29%

LC trace of the reaction of peptide **7** and **1l** and purified product **7l**. (10-80% B phase over 25 min, 1 mL·min^{-1} flow rate, 0.1% TFA, λ = 220 nm, Kromasil 100-5-C18 4.6 × 250 mm, 5 µm column). The table refer to the normalized integration of the peptide peaks.

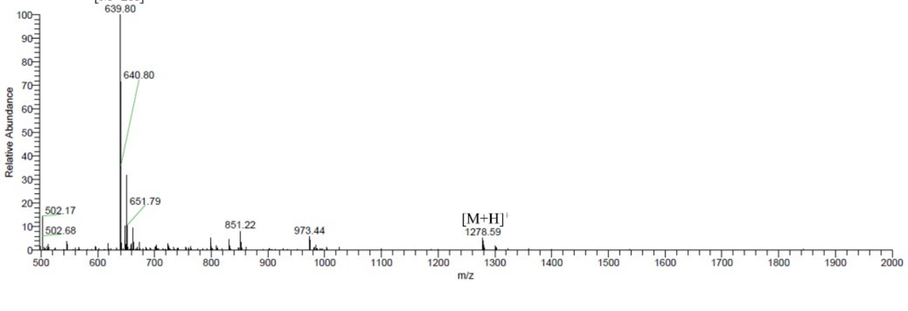

ESI Mass spectrum of purified product **7l**. Calculated Mass [M+H]$^+$: 1278.59; [M+2H]$^{2+}$: 639.80; Mass Found (ESI+) [M+H]$^+$: 1278.59; [M+2H]$^{2+}$: 639.80.

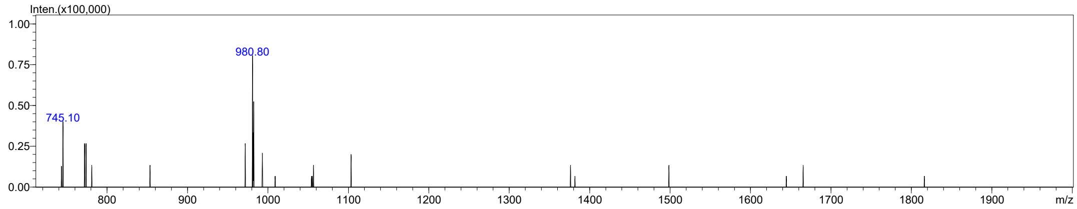

ESI Mass spectrum of By-product (**7** MS+7). Calculated Mass [M+H]$^+$: 980.45; Mass Found (ESI+) [M+H]$^+$: 980.80.

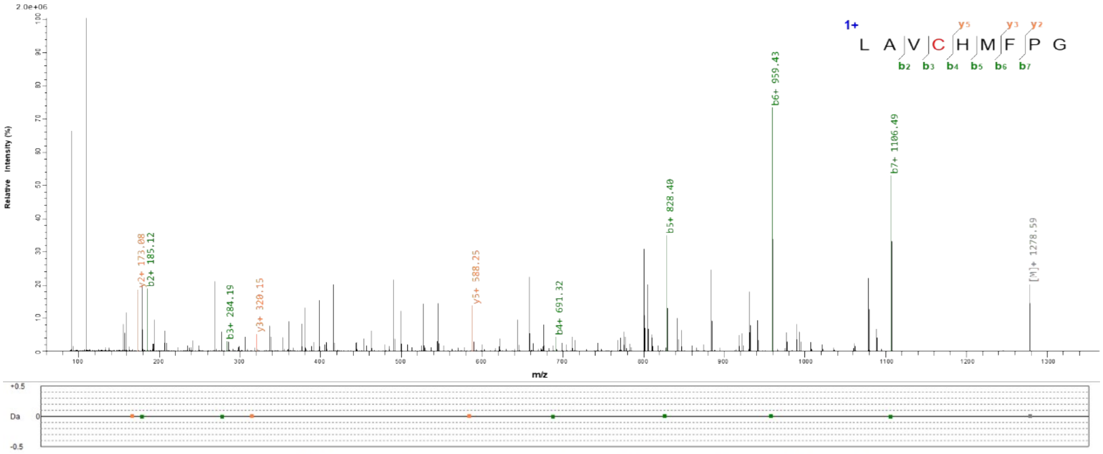

#	L(1/*)	A(2/8)	V(3/7)	C(4/6)	H(5/5)	M(6/4)	F(7/3)	P(8/2)	G(*/1)
b+	114.0913	185.1284	284.1969	691.3371	828.3960	959.4365	1106.5049	1203.5577	*
b++	57.5493	93.0679	142.6021	346.1722	414.7017	480.2219	553.7561	602.2825	*
y+	*	1165.5057	1094.4686	995.4001	588.2599	451.2010	320.1605	173.0921	76.0393
y++	*	583.2565	547.7379	498.2037	294.6336	226.1041	160.5839	87.0497	38.5233

De novo ms/ms analysis of purified product **7l**.

3.3.13. Characterization of 7m

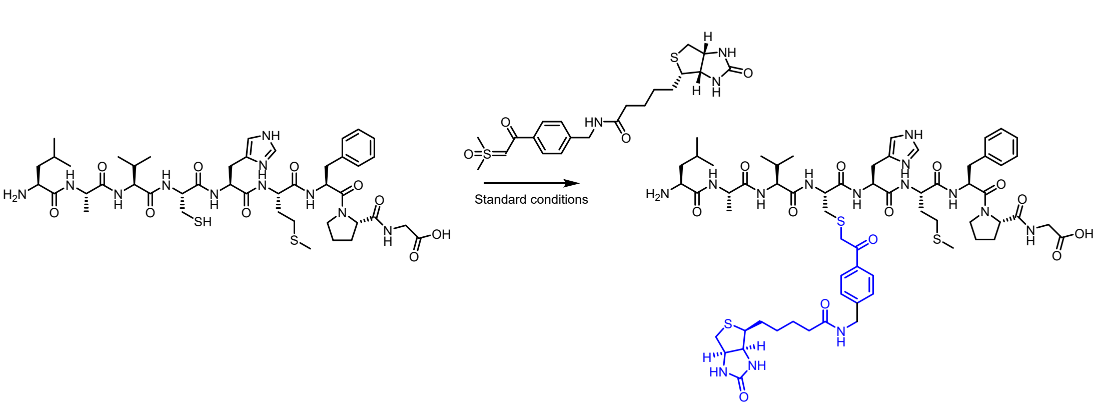

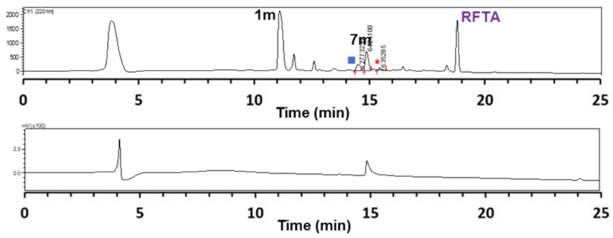

Peptide peak	Relative area
Substrate **7**	0
Target product **7m**	65%
By-product (disulfide) *	7%
Oxidized **7m** (MS+16) ■	28%

LC trace of the reaction of peptide **7** and **1m** and purified product **7m**. (10-80% B phase over 25 min, 1 mL·min^{-1} flow rate, 0.1% TFA, λ = 220 nm, Kromasil 100-5-C18 4.6 × 250 mm, 5 μm column). The table refer to the normalized integration of the peptide peaks.

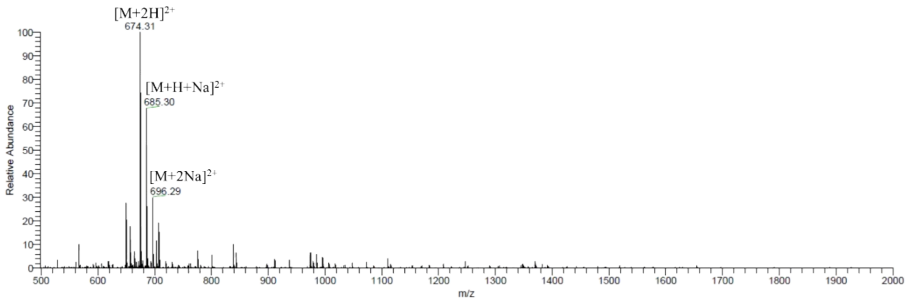

ESI Mass spectrum of purified product **7m**. Calculated Mass $[M+H]^{+}$: 1347.60; $[M+2H]^{2+}$: 674.31; Mass Found (ESI+) $[M+2H]^{2+}$: 674.31; $[M+H+Na]^{2+}$: 685.30; $[M+2Na]^{2+}$: 696.29.

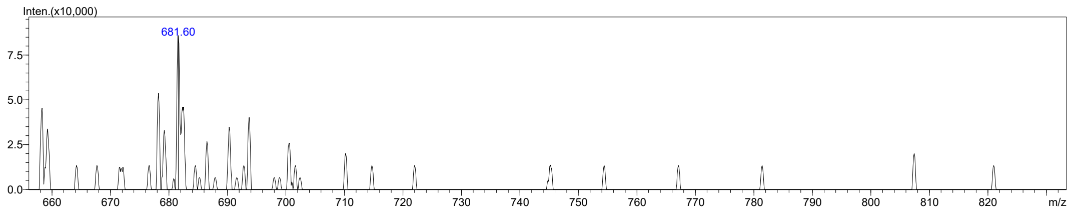

ESI Mass spectrum of Oxidized **7m** (MS+16). Calculated Mass $[M+H]^{+}$: 1363.60; $[M+2H]^{2+}$: 682.31; Mass Found (ESI+) $[M+2H]^{2+}$: 681.60.

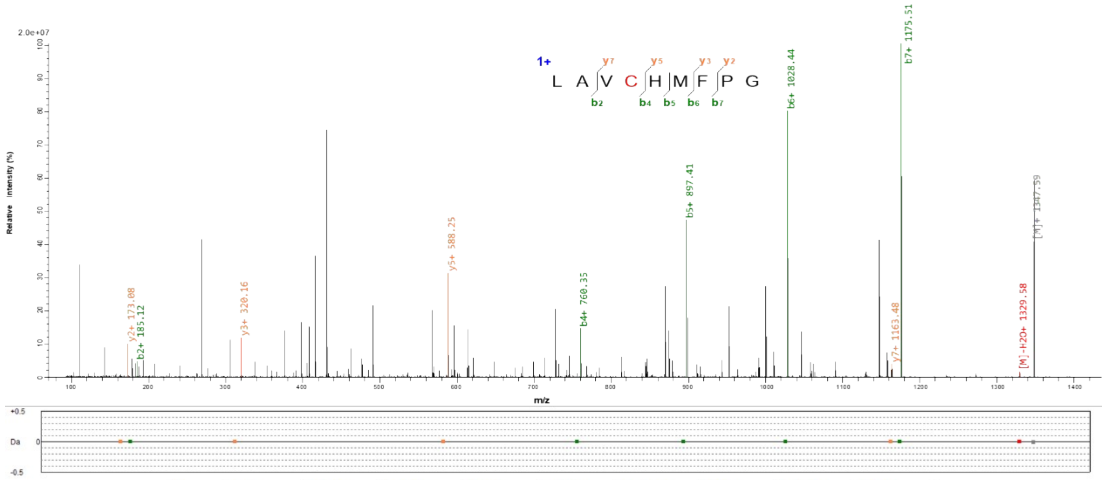

#	L(1/*)	A(2/8)	V(3/7)	C(4/6)	H(5/5)	M(6/4)	F(7/3)	P(8/2)	G(*/1)
b+	114.0913	185.1284	284.1969	760.3521	897.4110	1028.4515	1175.5199	1272.5726	*
b++	57.5493	93.0679	142.6021	380.6797	449.2091	514.7294	588.2636	636.7900	*
y+	*	1234.5206	1163.4835	1064.4151	588.2599	451.2010	320.1605	173.0921	76.0393
y++	*	617.7639	582.2454	532.7112	294.6336	226.1041	160.5839	87.0497	38.5233

De novo ms/ms analysis of purified product **7m**.

3.4. Characterization data for the reaction of peptide 8

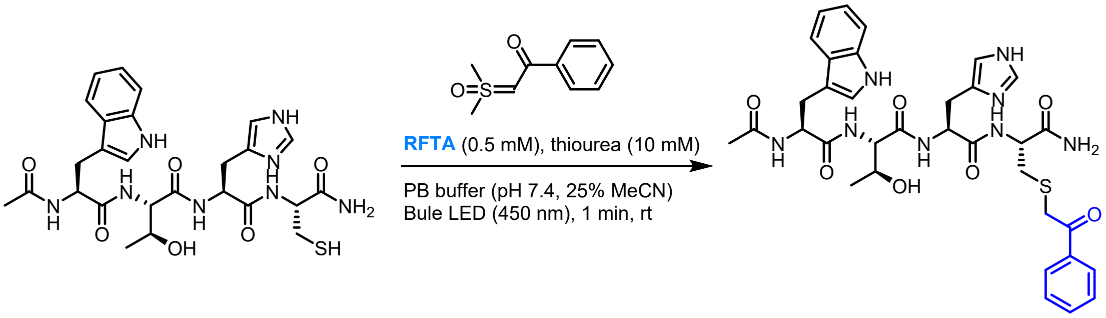

The reaction was followed **General procedure B** using peptide **8** (1 mM), sequence: Ac-WDE<u>**C**</u>-NH_2, and sulfoxonium ylide **1a**. A stock solution of peptide **8** (1 mM) was made up by 2 mL PB buffer (pH 7.4, 25% MeCN) and 1 mg peptide **8**.

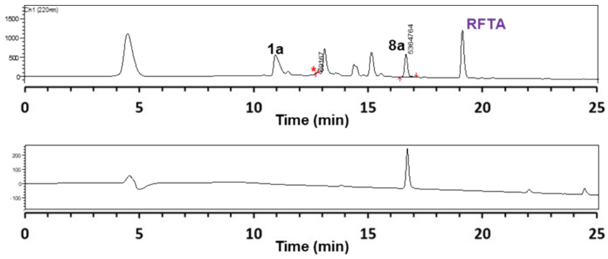

Peptide peak	Relative area
Substrate 8	0
Target product **8a**	99%
By-product (disulfide) *	1%

LC trace of the reaction of peptide **8** and **1a** and purified product **8a**. (10-80% B phase over 25 min, 1 mL·min^{-1} flow rate, 0.1% TFA, λ = 220 nm, Kromasil 100-5-C18 4.6 × 250 mm, 5 µm column). The table refer to the normalized integration of the peptide peaks.

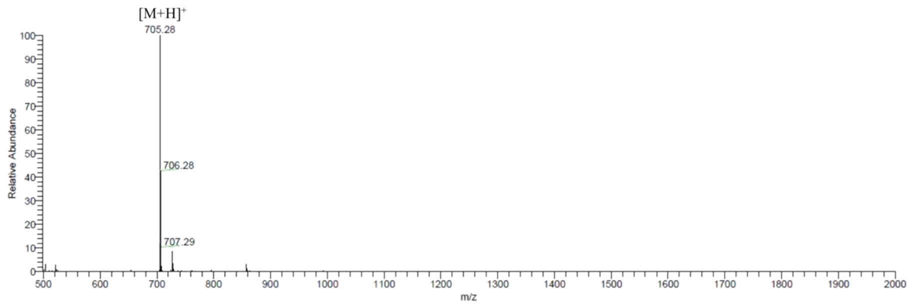

ESI Mass spectrum of purified product **8a**. Calculated Mass $[M+H]^+$: 705.26; Mass Found (ESI+) $[M+H]^+$: 705.26.

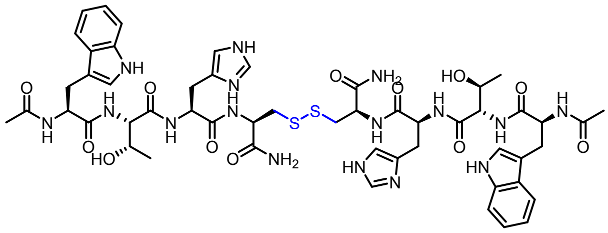

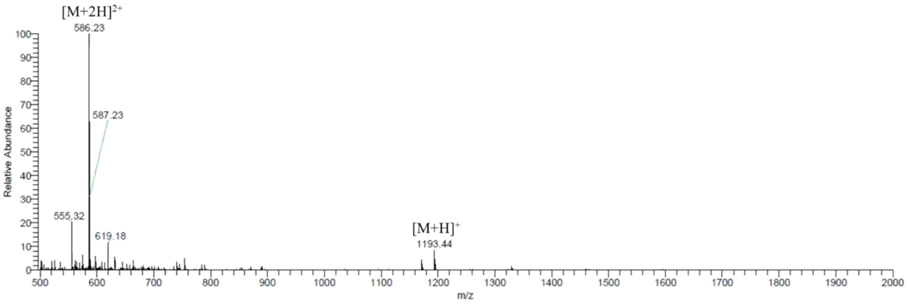

ESI Mass spectrum of oxidized by-product of **8** (disulfide). Calculated Mass [M+H]$^+$: 1193.44; [M+2H]$^{2+}$: 586.23; Mass Found (ESI+) [M+H]$^+$: 1193.44; [M+2H]$^{2+}$: 586.23.

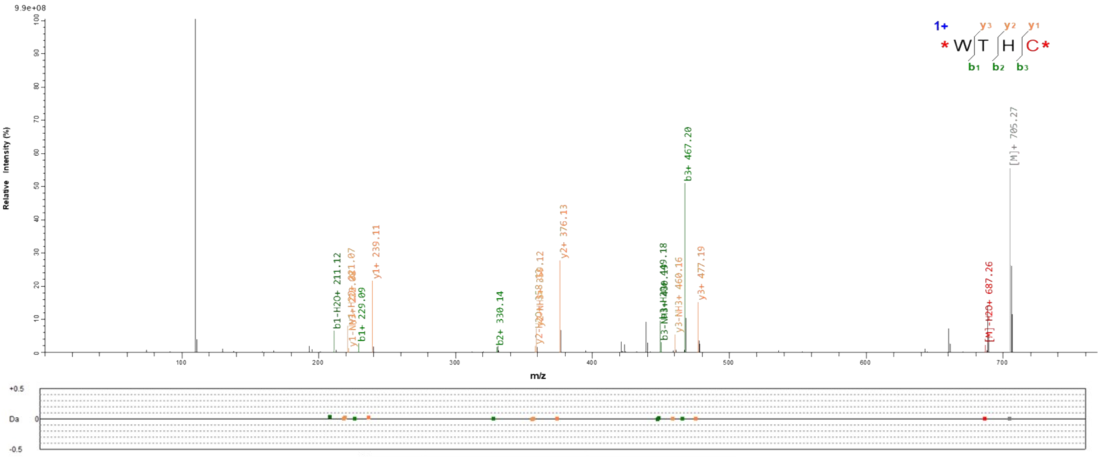

#	W(1/*)	T(2/3)	H(3/2)	C(*/1)
b+	229.0972	330.1448	467.2038	*
b++	115.0522	165.5761	234.1055	*
y+	*	477.1915	376.1438	239.0849
y++	*	239.0994	188.5755	120.0461

De novo ms/ms analysis of purified product **8a**.

3.5. Characterization data for the reaction of peptide 9

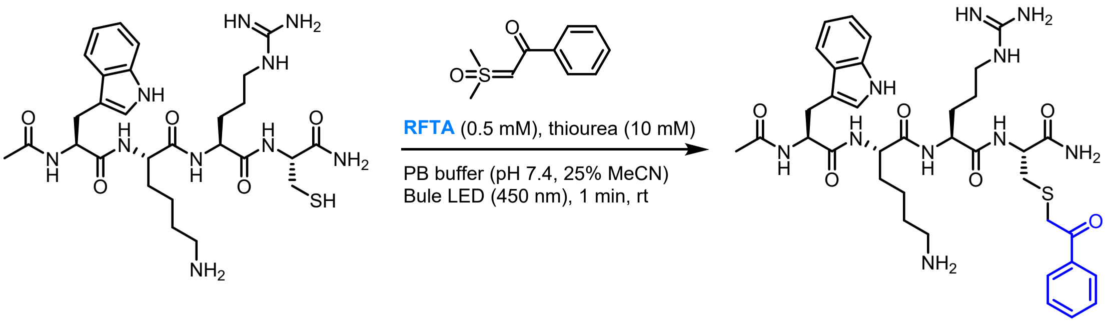

The reaction was followed **General procedure B** using peptide **9** (1 mM), sequence: Ac-WDEC-NH_2, and sulfoxonium ylide **1a**. A stock solution of peptide **9** (1 mM) was made up by 2 mL PB buffer (pH 7.4, 25% MeCN) and 1 mg peptide **9**.

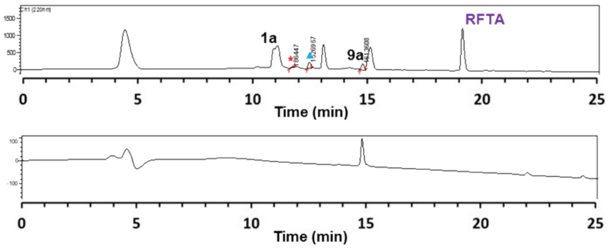

Peptide peak	Relative area
Substrate **9**	0
Target product **9a**	49%
By-product (disulfide) *	6%
By-product (**9** MS+78) ▲	45%

LC trace of the reaction of peptide **9** and **1a** and purified product **9a**. (10-80% B phase over 25 min, 1 mL·min^{-1} flow rate, 0.1% TFA, λ = 220 nm, Kromasil 100-5-C18 4.6 × 250 mm, 5 μm column). The table refer to the normalized integration of the peptide peaks.

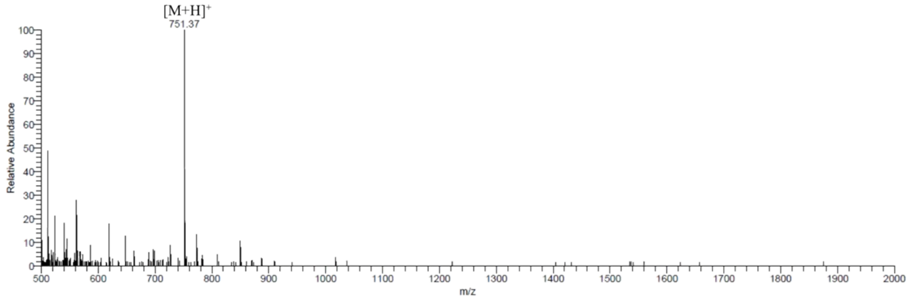

ESI Mass spectrum of purified product **9a**. Calculated Mass $[M+H]^+$: 751.37; Mass Found (ESI+) $[M+H]^+$: 751.37.

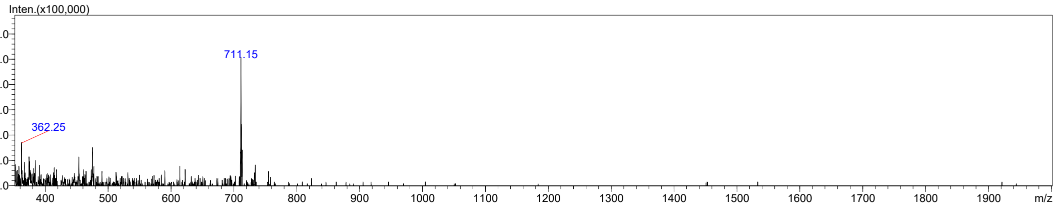

ESI Mass spectrum of by-product (**9** MS+78). Calculated Mass $[M+H]^+$: 711.32; Mass Found (ESI+) $[M+H]^+$: 711.15.

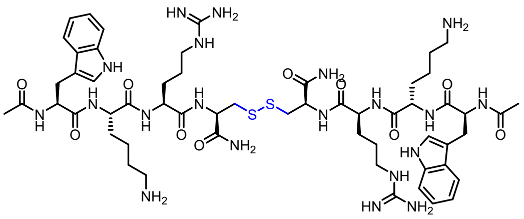

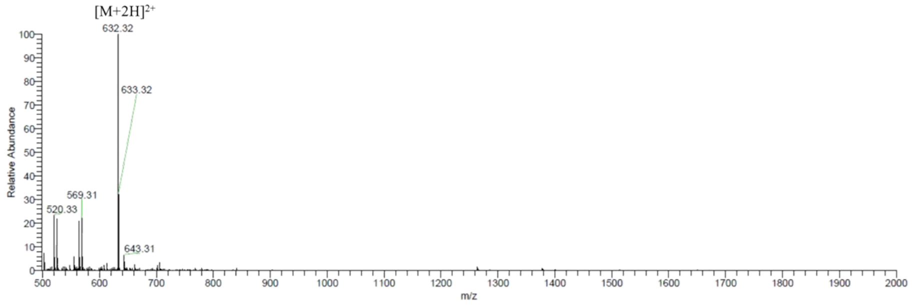

ESI Mass spectrum of oxidized by-product of **9** (disulfide). Calculated Mass $[M+H]^{+}$: 1262.63; $[M+2H]^{2+}$: 632.32; Mass Found (ESI+) $[M+2H]^{2+}$: 632.32.

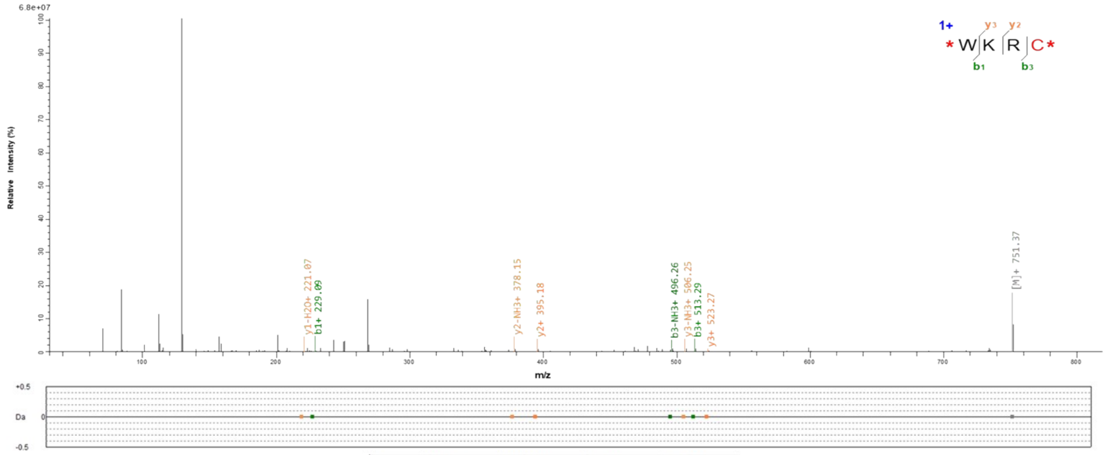

#	W(1/*)	K(2/3)	R(3/2)	C(*/1)
b+	229.0972	357.1921	513.2932	*
b++	115.0522	179.0997	257.1502	*
y+	*	523.2809	395.1860	239.0849
y++	*	262.1441	198.0966	120.0461

De novo ms/ms analysis of purified product **9a**.

3.6. Characterization data for the reaction of peptide 10

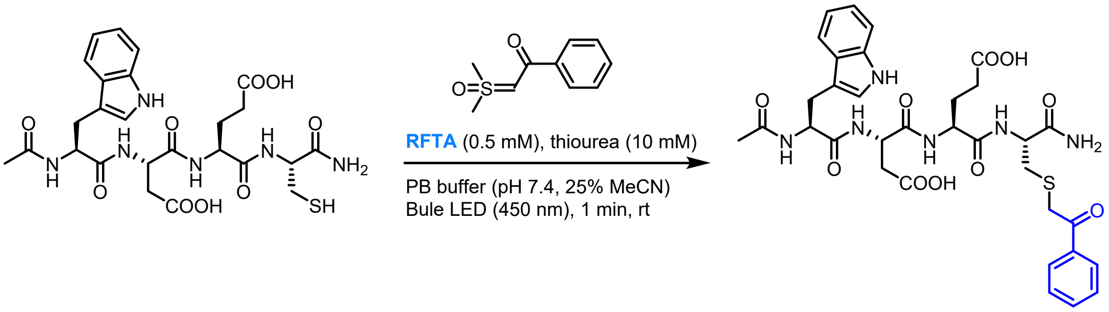

The reaction was followed **General procedure B** using peptide **10** (1 mM), sequence: Ac-WDE**C**-NH$_2$, and sulfoxonium ylide **1a**. A stock solution of peptide **10** (1 mM) was made up by 2 mL PB buffer (pH 7.4, 25% MeCN) and 1 mg peptide **10**.

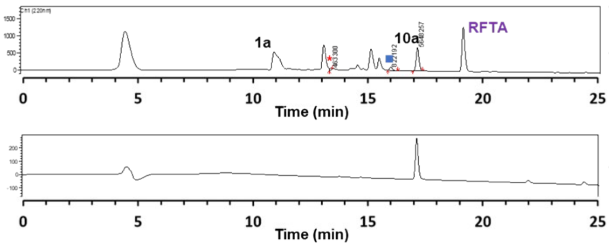

Peptide peak	Relative area
Substrate **10**	0
Target product **10a**	82%
By-product (disulfide) *	7%
Oxidized **10a** (MS+16) ■	11%

LC trace of the reaction of peptide **10** and **1a** and purified product **10a**. (10-80% B phase over 25 min, 1 mL·min^{-1} flow rate, 0.1% TFA, λ = 220 nm, Kromasil 100-5-C18 4.6 × 250 mm, 5 µm column). The table refer to the normalized integration of the peptide peaks.

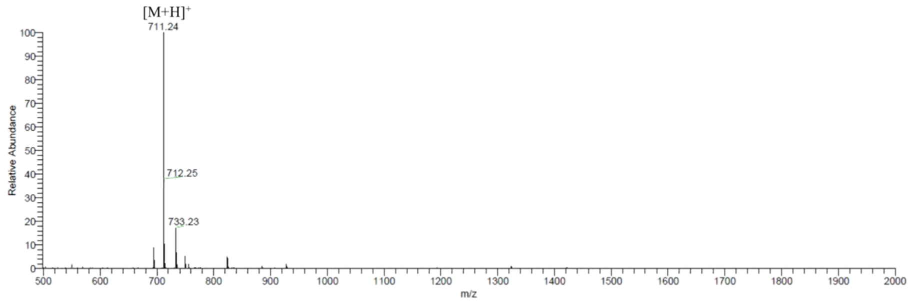

ESI Mass spectrum of purified product **10a**. Calculated Mass [M+H]$^+$: 711.24; Mass Found (ESI+) [M+H]$^+$: 711.24.

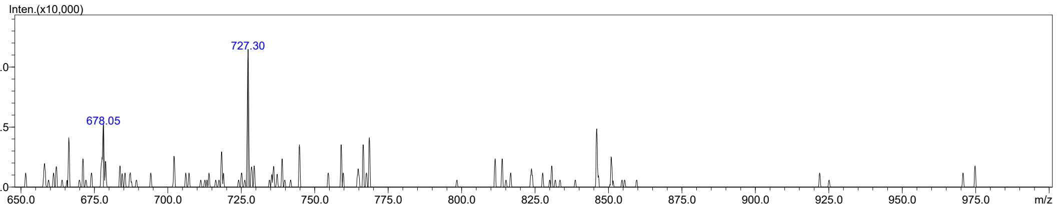

ESI Mass spectrum of oxidized **10a** (MS+16). Calculated Mass [M+H]$^+$: 727.24; Mass Found (ESI+) [M+H]$^+$: 727.30.

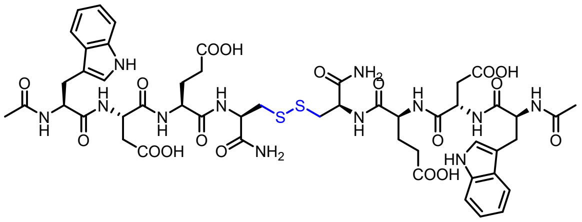

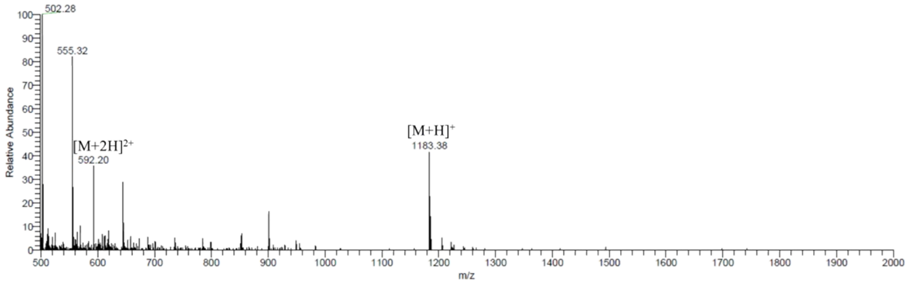

ESI Mass spectrum of oxidized by-product of **10** (disulfide). Calculated Mass $[M+H]^+$: 1183.38; $[M+2H]^{2+}$: 592.20; Mass Found (ESI+) $[M+H]^+$: 1183.38; $[M+2H]^{2+}$: 592.20.

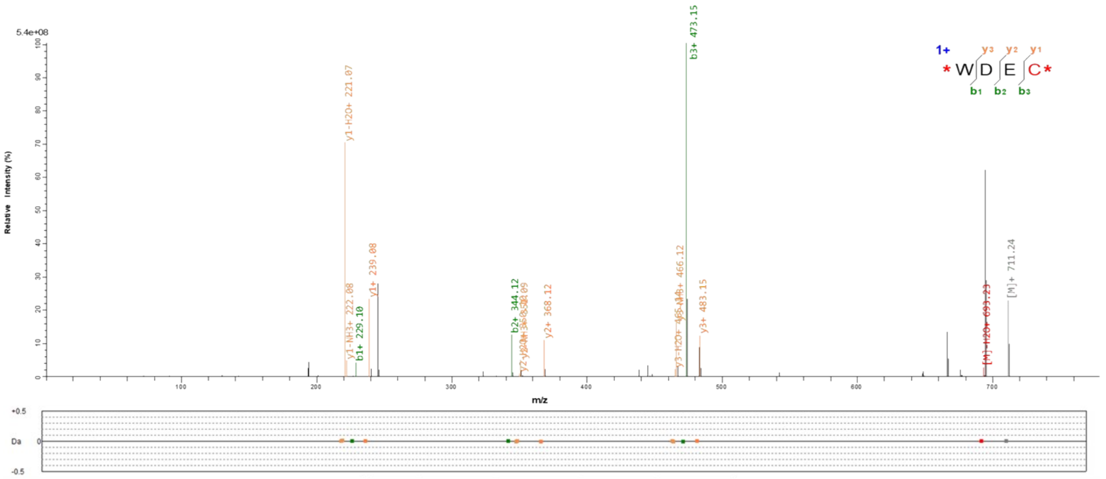

#	W(1/*)	D(2/3)	E(3/2)	C(*/1)
b+	229.0972	344.1241	473.1667	*
b++	115.0522	172.5657	237.0870	*
y+	*	483.1544	368.1275	239.0849
y++	*	242.0808	184.5674	120.0461

De novo ms/ms analysis of purified product **10a**.

3.7. Characterization data for the reaction of peptide 11

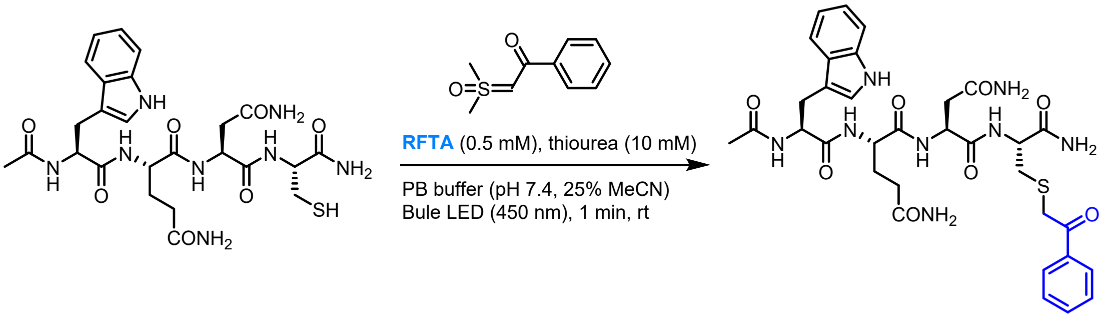

The reaction was followed **General procedure B** using peptide **11** (1 mM), sequence: Ac-WDE<u>**C**</u>-NH_2, and sulfoxonium ylide **1a**. A stock solution of peptide **11** (1 mM) was made up by 2 mL PB buffer (pH 7.4, 25% MeCN) and 1 mg peptide **11**.

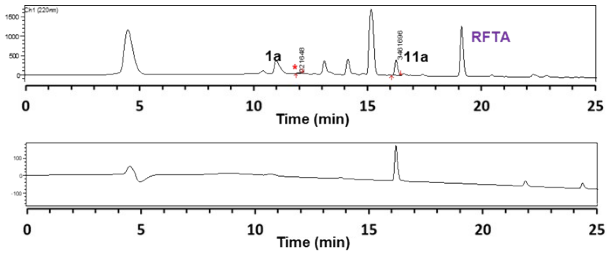

Peptide peak	Relative area
Substrate **11**	0
Target product **11a**	94%
By-product (disulfide) *	6%

LC trace of the reaction of peptide **11** and **1a** and purified product **11a**. (10-80% B phase over 25 min, 1 mL·min^{-1} flow rate, 0.1% TFA, λ = 220 nm, Kromasil 100-5-C18 4.6 × 250 mm, 5 µm column). The table refer to the normalized integration of the peptide peaks.

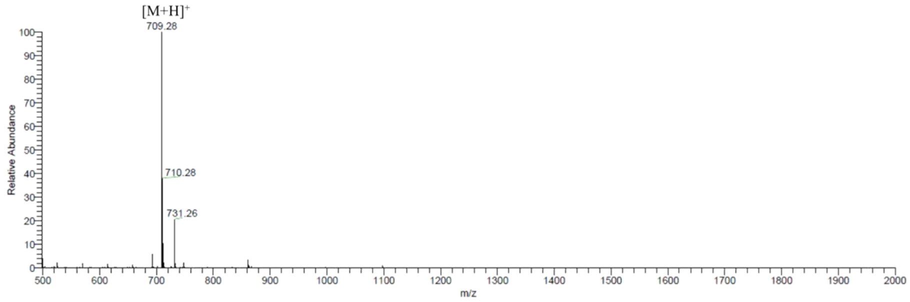

ESI Mass spectrum of purified product **11a**. Calculated Mass $[M+H]^+$: 709.28; Mass Found (ESI+) $[M+H]^+$: 709.28.

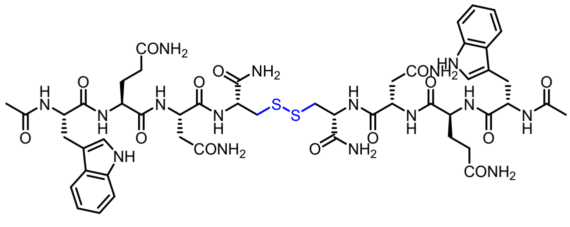

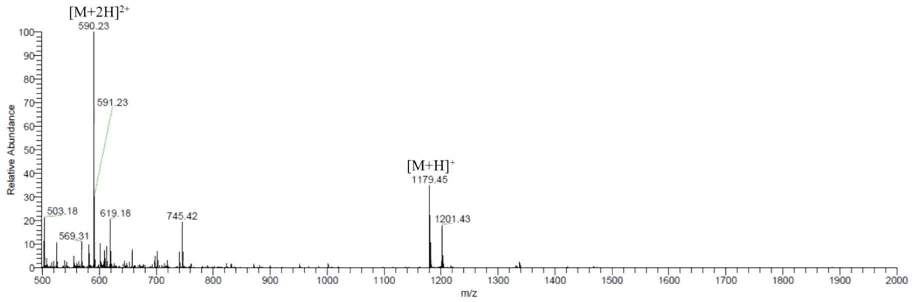

ESI Mass spectrum of oxidized by-product of **11** (disulfide). Calculated Mass $[M+H]^+$: 1179.45; $[M+2H]^{2+}$: 590.23; Mass Found (ESI+) $[M+H]^+$: 1179.45; $[M+2H]^{2+}$: 590.23.

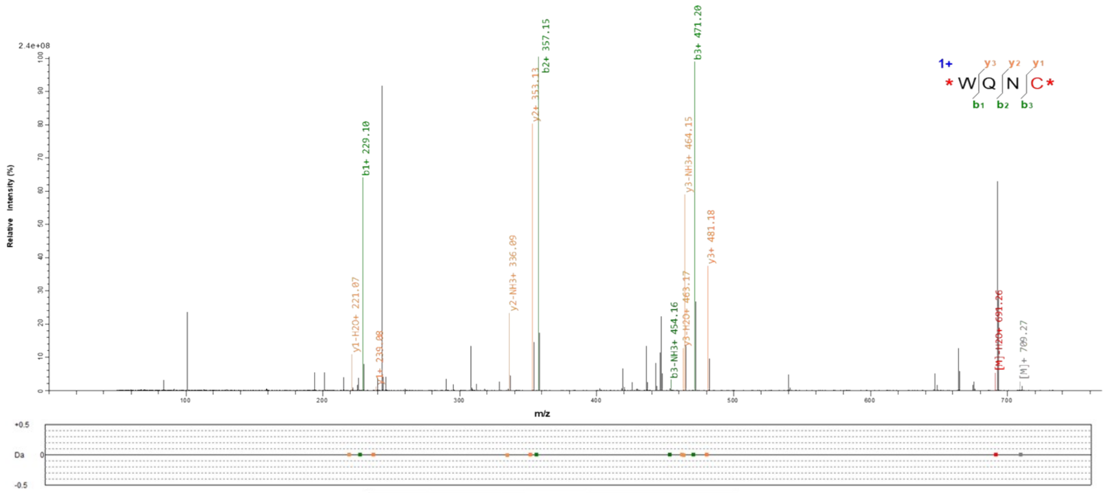

#	W(1/*)	Q(2/3)	N(3/2)	C(*/1)
b+	229.0972	357.1557	471.1987	*
b++	115.0522	179.0815	236.1030	*
y+	*	481.1864	353.1278	239.0849
y++	*	241.0968	177.0675	120.0461

De novo ms/ms analysis of purified product **11a**.

3.8. Characterization data for the reaction of peptide 12

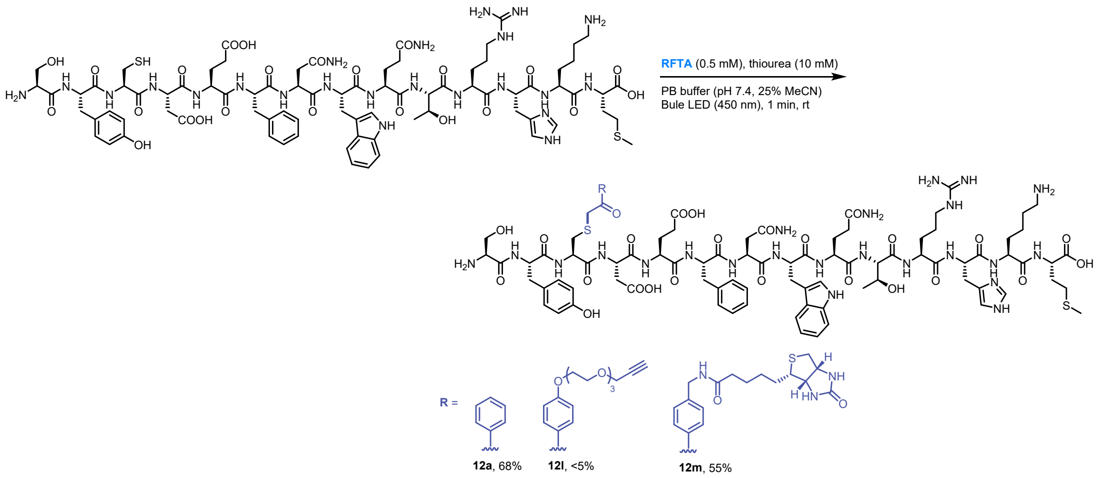

The reaction was followed **General procedure B** using peptide **12** (1 mM), sequence: NH_2-SY**C**DEFNWQTRHKM-OH, and sulfoxonium ylide **1a, 1l** and **1m**. A stock solution of peptide **12** (1 mM) was made up by 2 mL PB buffer (pH 7.4, 25% MeCN) and 3.7 mg peptide **12**.

3.8.1. Characterization of 12a

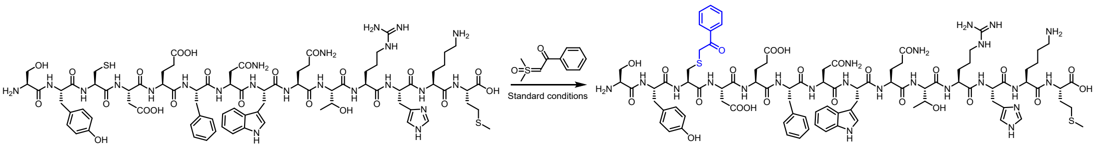

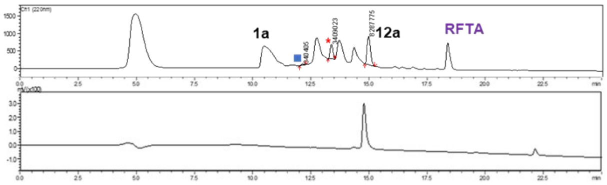

Peptide peak	Relative area
Substrate **12**	0
Target product **12a**	68%
By-product (disulfide) *	28%
Oxidized **12** (MS+16) ■	4%

LC trace of the reaction of peptide **12** and **1a** and purified product **12a**. (10-80% B phase over 25 min, 1 mL·min^{-1} flow rate, 0.1% TFA, λ = 220 nm, Kromasil 100-5-C18 4.6 × 250 mm, 5 μm column). The table refer to the normalized integration of the peptide peaks.

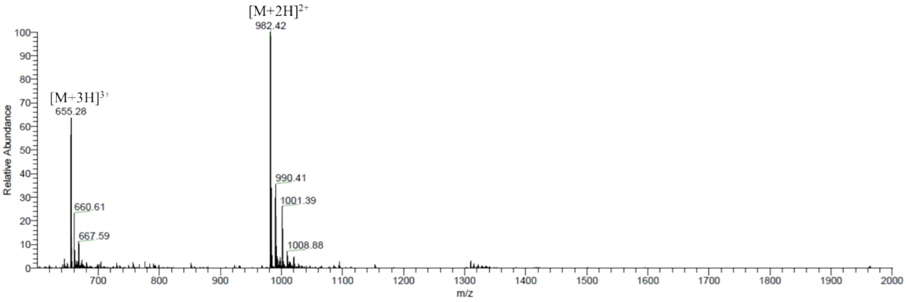

ESI Mass spectrum of purified product **12a**. Calculated Mass $[M+2H]^{2+}$: 982.42; $[M+3H]^{3+}$: 655.28; Mass Found (ESI+) $[M+2H]^{2+}$: 982.42; $[M+3H]^{3+}$: 655.28.

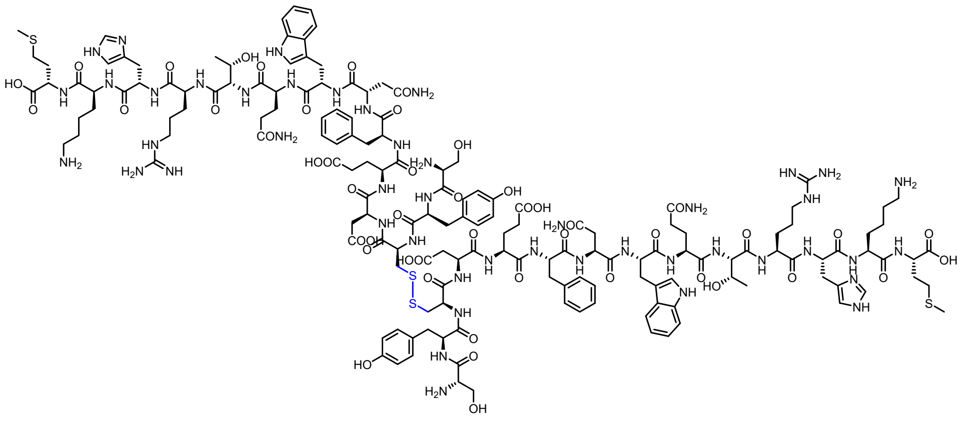

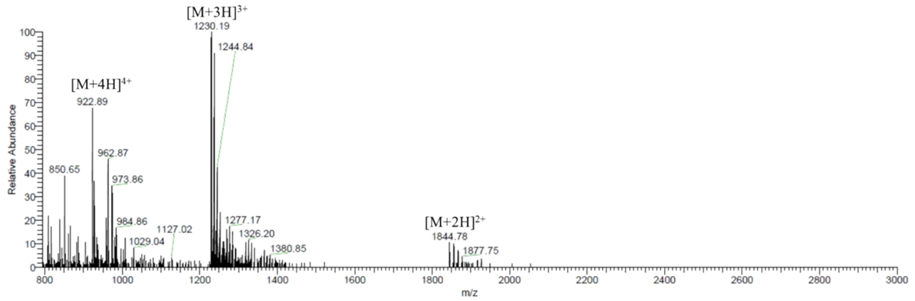

ESI Mass spectrum of oxidized by-product of **12** (disulfide). Calculated Mass [M+H]$^{+}$: 3686.54; [M+2H]$^{2+}$: 1844.78; [M+3H]$^{3+}$: 1230.19; [M+4H]$^{4+}$: 922.89; Mass Found (ESI+) Calculated Mass [M+H]$^{+}$: 3686.54; [M+2H]$^{2+}$: 1844.78; [M+3H]$^{3+}$: 1230.19; [M+4H]$^{4+}$: 922.89.

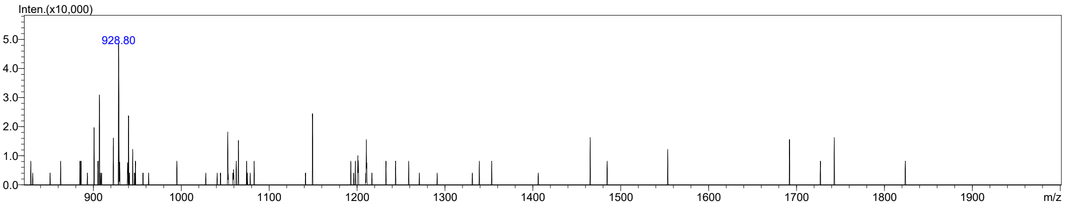

ESI Mass spectrum of oxidized **12** (MS+16). Calculated Mass [M-H]$^{-}$: 1858.78; [M-2H]$^{2-}$: 928.89; Mass Found (ESI+) Calculated Mass [M-2H]$^{2-}$: 928.80.

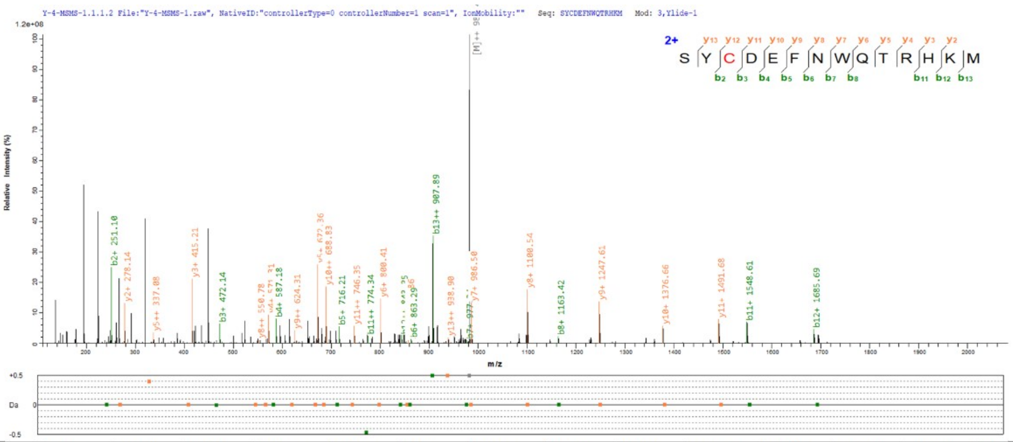

#	S(1/*)	Y(2/13)	C(3/12)	D(4/11)	E(5/10)	F(6/9)	N(7/8)	W(8/7)	Q(9/6)	T(10/5)	R(11/4)	H(12/3)	K(13/2)	M(*/1)
b+	88.0393	251.1026	472.1537	587.1806	716.2232	863.2916	977.3345	1163.4139	1291.4724	1392.5201	1548.6212	1685.6801	1813.7751	*
b++	44.5233	126.0550	236.5805	294.0939	358.6152	432.1494	489.1709	582.2106	646.2399	696.7637	774.8143	843.3437	907.3912	*
y+	*	1875.7941	1712.7308	1491.6798	1376.6528	1247.6102	1100.5418	986.4989	800.4196	672.3610	571.3133	415.2122	278.1533	150.0583
y++	*	938.4007	856.8690	746.3435	688.8300	624.3088	550.7745	493.7531	400.7134	336.6841	286.1603	208.1097	139.5803	75.5328

De novo ms/ms analysis of purified product **12a**.

The MS/MS searching on other nucleophilic residues of product 12a

The MS/MS searching on K site:

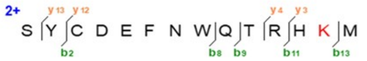

#	S(1/*)	Y(2/13)	C(3/12)	D(4/11)	E(5/10)	F(6/9)	N(7/8)	W(8/7)	Q(9/6)	T(10/5)	R(11/4)	H(12/3)	K(13/2)	M(*/1)
b+	88.0393	251.1026	354.1118	469.1388	598.1813	745.2498	859.2927	1045.3720	1173.4306	1274.4783	1430.5794	1567.6383	1813.7751	*
b++	44.5233	126.0550	177.5595	235.0730	299.5943	373.1285	430.1500	523.1896	587.2189	637.7428	715.7933	784.3228	907.3912	*
y+	*	1875.7941	1712.7308	1609.7216	1494.6947	1365.6521	1218.5837	1104.5407	918.4614	790.4029	689.3552	533.2541	396.1952	150.0583
y++	*	938.4007	856.8690	805.3644	747.8510	683.3297	609.7955	552.7740	459.7344	395.7051	345.1812	267.1307	198.6012	75.5328

The MS/MS searching on R site:

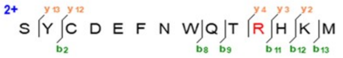

#	S(1/*)	Y(2/13)	C(3/12)	D(4/11)	E(5/10)	F(6/9)	N(7/8)	W(8/7)	Q(9/6)	T(10/5)	R(11/4)	H(12/3)	K(13/2)	M(*/1)
b+	88.0393	251.1026	354.1118	469.1388	598.1813	745.2498	859.2927	1045.3720	1173.4306	1274.4783	1548.6212	1685.6801	1813.7751	*
b++	44.5233	126.0550	177.5595	235.0730	299.5943	373.1285	430.1500	523.1896	587.2189	637.7428	774.8143	843.3437	907.3912	*
y+	*	1875.7941	1712.7308	1609.7216	1494.6947	1365.6521	1218.5837	1104.5407	918.4614	790.4029	689.3552	415.2122	278.1533	150.0583
y++	*	938.4007	856.8690	805.3644	747.8510	683.3297	609.7955	552.7740	459.7344	395.7051	345.1812	208.1097	139.5803	75.5328

The MS/MS searching on H site:

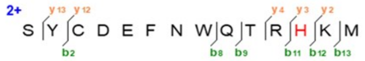

#	S(1/*)	Y(2/13)	C(3/12)	D(4/11)	E(5/10)	F(6/9)	N(7/8)	W(8/7)	Q(9/6)	T(10/5)	R(11/4)	H(12/3)	K(13/2)	M(*/1)
b+	88.0393	251.1026	354.1118	469.1388	598.1813	745.2498	859.2927	1045.3720	1173.4306	1274.4783	1430.5794	1685.6801	1813.7751	*
b++	44.5233	126.0550	177.5595	235.0730	299.5943	373.1285	430.1500	523.1896	587.2189	637.7428	715.7933	843.3437	907.3912	*
y+	*	1875.7941	1712.7308	1609.7216	1494.6947	1365.6521	1218.5837	1104.5407	918.4614	790.4029	689.3552	533.2541	278.1533	150.0583
y++	*	938.4007	856.8690	805.3644	747.8510	683.3297	609.7955	552.7740	459.7344	395.7051	345.1812	267.1307	139.5803	75.5328

The MS/MS searching on Y site:

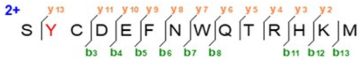

#	S(1/*)	Y(2/13)	C(3/12)	D(4/11)	E(5/10)	F(6/9)	N(7/8)	W(8/7)	Q(9/6)	T(10/5)	R(11/4)	H(12/3)	K(13/2)	M(*/1)
b+	88.0393	369.1445	472.1537	587.1806	716.2232	863.2916	977.3345	1163.4139	1291.4724	1392.5201	1548.6212	1685.6801	1813.7751	*
b++	44.5233	185.0759	236.5805	294.0939	358.6152	432.1494	489.1709	582.2106	646.2399	696.7637	774.8143	843.3437	907.3912	*
y+	*	1875.7941	1594.6889	1491.6798	1376.6528	1247.6102	1100.5418	986.4989	800.4196	672.3610	571.3133	415.2122	278.1533	150.0583
y++	*	938.4007	797.8481	746.3435	688.8300	624.3088	550.7745	493.7531	400.7134	336.6841	286.1603	208.1097	139.5803	75.5328

The MS/MS searching on S site:

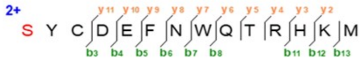

#	S(1/*)	Y(2/13)	C(3/12)	D(4/11)	E(5/10)	F(6/9)	N(7/8)	W(8/7)	Q(9/6)	T(10/5)	R(11/4)	H(12/3)	K(13/2)	M(*/1)
b+	206.0812	369.1445	472.1537	587.1806	716.2232	863.2916	977.3345	1163.4139	1291.4724	1392.5201	1548.6212	1685.6801	1813.7751	*
b++	103.5442	185.0759	236.5805	294.0939	358.6152	432.1494	489.1709	582.2106	646.2399	696.7637	774.8143	843.3437	907.3912	*
y+	*	1757.7523	1594.6889	1491.6798	1376.6528	1247.6102	1100.5418	986.4989	800.4196	672.3610	571.3133	415.2122	278.1533	150.0583
y++	*	879.3798	797.8481	746.3435	688.8300	624.3088	550.7745	493.7531	400.7134	336.6841	286.1603	208.1097	139.5803	75.5328

The MS/MS searching on T site:

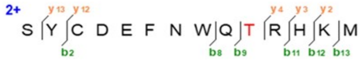

#	S(1/*)	Y(2/13)	C(3/12)	D(4/11)	E(5/10)	F(6/9)	N(7/8)	W(8/7)	Q(9/6)	T(10/5)	R(11/4)	H(12/3)	K(13/2)	M(*/1)
b+	88.0393	251.1026	354.1118	469.1388	598.1813	745.2498	859.2927	1045.3720	1173.4306	1392.5201	1548.6212	1685.6801	1813.7751	*
b++	44.5233	126.0550	177.5595	235.0730	299.5943	373.1285	430.1500	523.1896	587.2189	696.7637	774.8143	843.3437	907.3912	*
y+	*	1875.7941	1712.7308	1609.7216	1494.6947	1365.6521	1218.5837	1104.5407	918.4614	790.4029	571.3133	415.2122	278.1533	150.0583
y++	*	938.4007	856.8690	805.3644	747.8510	683.3297	609.7955	552.7740	459.7344	395.7051	286.1603	208.1097	139.5803	75.5328

The MS/MS searching on D site:

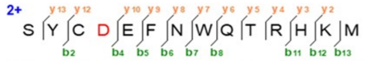

#	S(1/*)	Y(2/13)	C(3/12)	D(4/11)	E(5/10)	F(6/9)	N(7/8)	W(8/7)	Q(9/6)	T(10/5)	R(11/4)	H(12/3)	K(13/2)	M(*/1)
b+	88.0393	251.1026	354.1118	587.1806	716.2232	863.2916	977.3345	1163.4139	1291.4724	1392.5201	1548.6212	1685.6801	1813.7751	*
b++	44.5233	126.0550	177.5595	294.0939	358.6152	432.1494	489.1709	582.2106	646.2399	696.7637	774.8143	843.3437	907.3912	*
y+	*	1875.7941	1712.7308	1609.7216	1376.6528	1247.6102	1100.5418	986.4989	800.4196	672.3610	571.3133	415.2122	278.1533	150.0583
y++	*	938.4007	856.8690	805.3644	688.8300	624.3088	550.7745	493.7531	400.7134	336.6841	286.1603	208.1097	139.5803	75.5328

The MS/MS searching on E site:

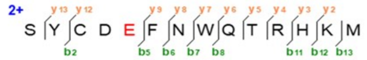

#	S(1/*)	Y(2/13)	C(3/12)	D(4/11)	E(5/10)	F(6/9)	N(7/8)	W(8/7)	Q(9/6)	T(10/5)	R(11/4)	H(12/3)	K(13/2)	M(*/1)
b+	88.0393	251.1026	354.1118	469.1388	716.2232	863.2916	977.3345	1163.4139	1291.4724	1392.5201	1548.6212	1685.6801	1813.7751	*
b++	44.5233	126.0550	177.5595	235.0730	358.6152	432.1494	489.1709	582.2106	646.2399	696.7637	774.8143	843.3437	907.3912	*
y+	*	1875.7941	1712.7308	1609.7216	1494.6947	1247.6102	1100.5418	986.4989	800.4196	672.3610	571.3133	415.2122	278.1533	150.0583
y++	*	938.4007	856.8690	805.3644	747.8510	624.3088	550.7745	493.7531	400.7134	336.6841	286.1603	208.1097	139.5803	75.5328

The MS/MS searching on Q site:

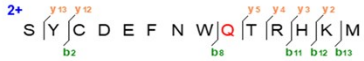

#	S(1/*)	Y(2/13)	C(3/12)	D(4/11)	E(5/10)	F(6/9)	N(7/8)	W(8/7)	Q(9/6)	T(10/5)	R(11/4)	H(12/3)	K(13/2)	M(*/1)
b+	88.0393	251.1026	354.1118	469.1388	598.1813	745.2498	859.2927	1045.3720	1291.4724	1392.5201	1548.6212	1685.6801	1813.7751	*
b++	44.5233	126.0550	177.5595	235.0730	299.5943	373.1285	430.1500	523.1896	646.2399	696.7637	774.8143	843.3437	907.3912	*
y+	*	1875.7941	1712.7308	1609.7216	1494.6947	1365.6521	1218.5837	1104.5407	918.4614	672.3610	571.3133	415.2122	278.1533	150.0583
y++	*	938.4007	856.8690	805.3644	747.8510	683.3297	609.7955	552.7740	459.7344	336.6841	286.1603	208.1097	139.5803	75.5328

The MS/MS searching on N site:

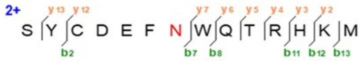

#	S(1/*)	Y(2/13)	C(3/12)	D(4/11)	E(5/10)	F(6/9)	N(7/8)	W(8/7)	Q(9/6)	T(10/5)	R(11/4)	H(12/3)	K(13/2)	M(*/1)
b+	88.0393	251.1026	354.1118	469.1388	598.1813	745.2498	977.3345	1163.4139	1291.4724	1392.5201	1548.6212	1685.6801	1813.7751	*
b++	44.5233	126.0550	177.5595	235.0730	299.5943	373.1285	489.1709	582.2106	646.2399	696.7637	774.8143	843.3437	907.3912	*
y+	*	1875.7941	1712.7308	1609.7216	1494.6947	1365.6521	1218.5837	986.4989	800.4196	672.3610	571.3133	415.2122	278.1533	150.0583
y++	*	938.4007	856.8690	805.3644	747.8510	683.3297	609.7955	493.7531	400.7134	336.6841	286.1603	208.1097	139.5803	75.5328

In order to further confirm the potential functionalization on other nucleophilic residues, we have used the pLabel (v2.4) software[12] to automatically match the b-/y- ion fragments of products under the default parameters with a TOL (0.5 Da), the matching mass tolerance of fragment ions. As shown above, the analysis on the C site exhibited abundant MS fragments and the key fragment **b3** and **y12** were found. On the contrary, the analysis on the other nucleophilic sites exhibited a small number of fragments. Especially, the reliable match for site-adjacent key fragments were absent for these residues. For example, even though a few key fragments, **y3** (689.4) and **y4** (267.1), for K and H site were coincidently found, but the fragments between C3 and W8 were completely absent in the searching. Thus, the results demonstrated that the cysteine site is the only possible adducted position in product **12a**.

3.8.2. Characterization of 12l

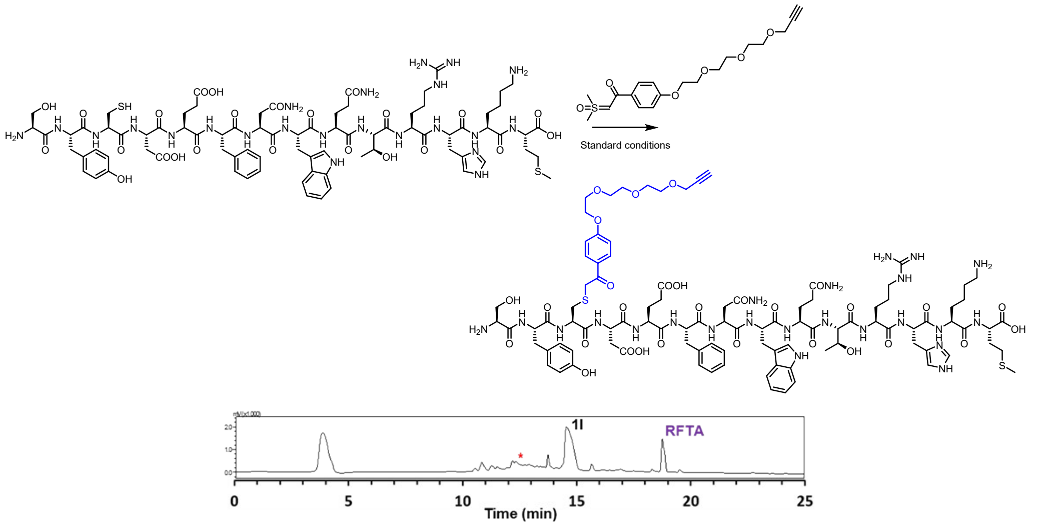

LC trace of the reaction of peptide **12** and **1l** and purified product **12l**. (10-80% B phase over 25 min, 1 mL·min^{-1} flow rate, 0.1% TFA, λ = 220 nm, Kromasil 100-5-C18 4.6 × 250 mm, 5 µm column).

3.8.3. Characterization of 12m

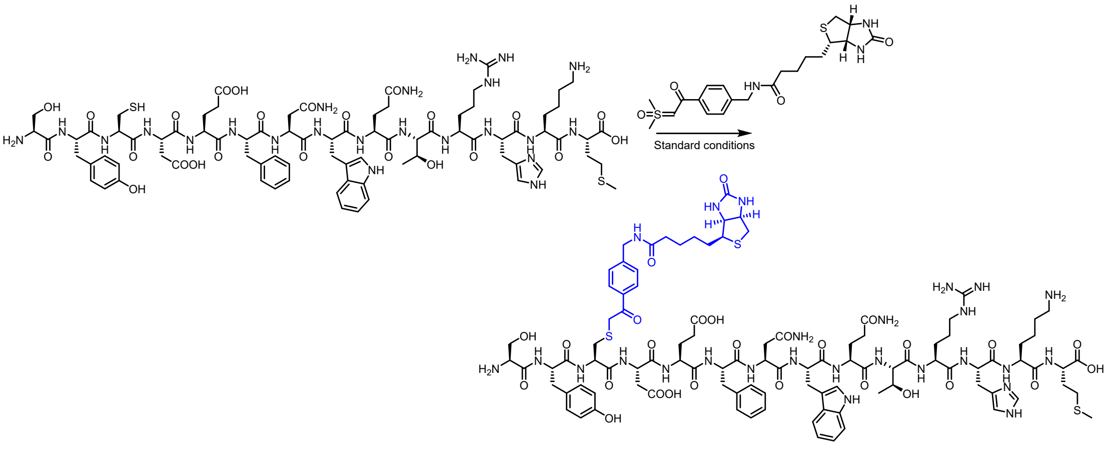

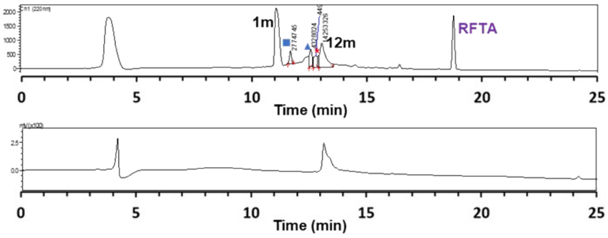

Peptide peak	Relative area
Substrate **12**	0
Target product **12m**	55%
By-product (disulfide) *	17%
Oxidized **12** (MS+16) ■	17%
Oxidized **12m** (MS+16) ▲	11%

LC trace of the reaction of peptide **12** and **1m** and purified product **12m**. (10-80% B phase over 25 min, 1 mL·min^{-1} flow rate, 0.1% TFA, λ = 220 nm, Kromasil 100-5-C18 4.6 × 250 mm, 5 µm column). The table refer to the normalized integration of the peptide peaks.

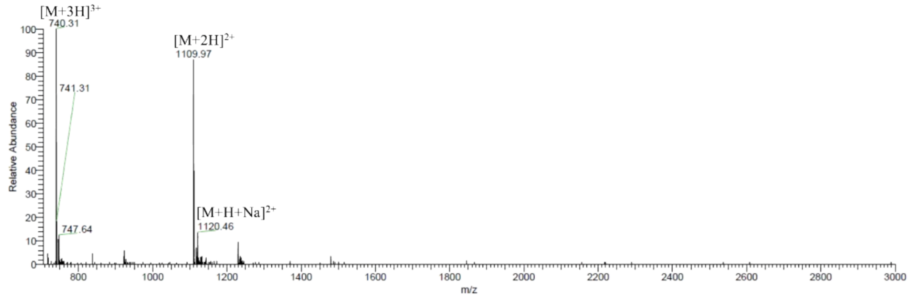

ESI Mass spectrum of purified product **12m**. Calculated Mass [M+H]$^{+}$: 2217.93; [M+2H]$^{2+}$: 1109.97; [M+3H]$^{3+}$: 740.31; Mass Found (ESI+) [M+H+Na]$^{2+}$: 1120.46; [M+2H]$^{2+}$: 1109.97; [M+3H]$^{3+}$: 740.31.

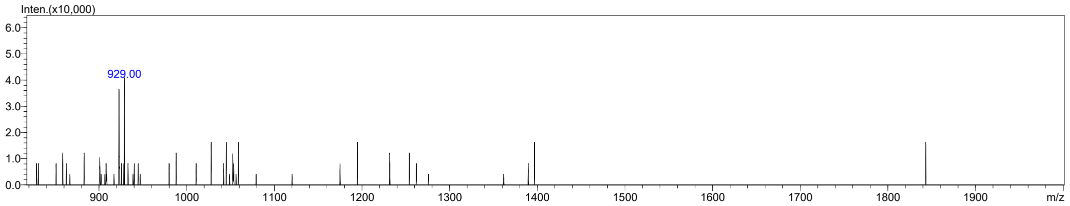

ESI Mass spectrum of oxidized **12** (MS+16). Calculated Mass [M-H]$^{-}$: 1858.78; [M-2H]$^{2-}$: 928.89; Mass Found (ESI+) Calculated Mass [M-2H]$^{2-}$: 929.00.

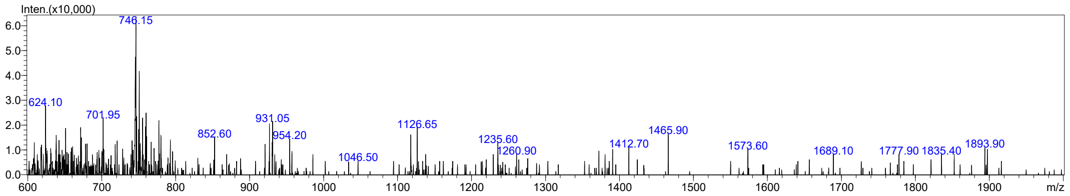

ESI Mass spectrum of oxidized **12m** (MS+16). Calculated Mass [M+H]$^{+}$: 2233.92; [M+2H]$^{2+}$: 1117.46; [M+3H]$^{3+}$: 745.31; Mass Found (ESI+) Calculated Mass [M+3H]$^{3+}$: 746.15.

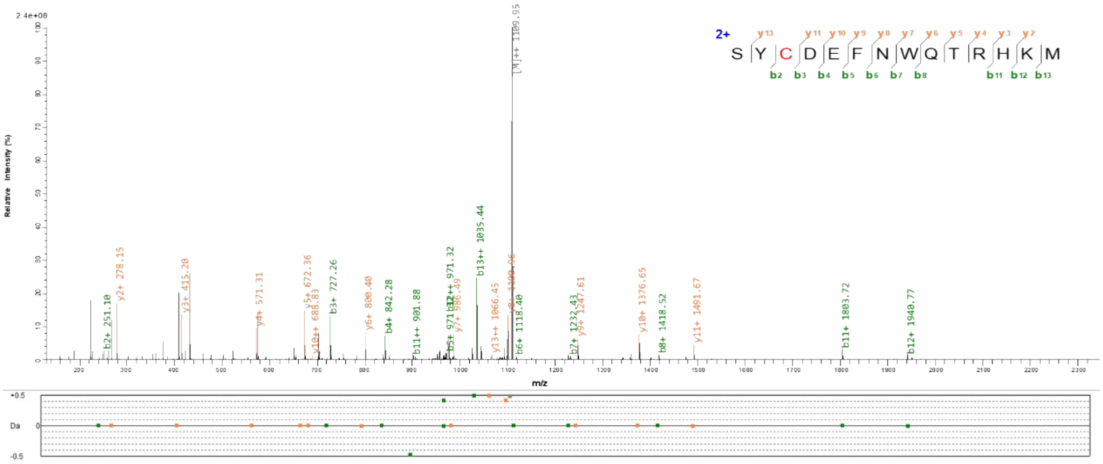

#	S(1/*)	Y(2/13)	C(3/12)	D(4/11)	E(5/10)	F(6/9)	N(7/8)	W(8/7)	Q(9/6)	T(10/5)	R(11/4)	H(12/3)	K(13/2)	M(*/1)
b+	88.0393	251.1026	727.2578	842.2848	971.3274	1118.3958	1232.4387	1418.5180	1546.5766	1647.6243	1803.7254	1940.7843	2068.8792	*
b++	44.5233	126.0550	364.1326	421.6460	486.1673	559.7015	616.7230	709.7626	773.7919	824.3158	902.3663	970.8958	1034.9433	*
y+	*	2130.8983	1967.8350	1491.6798	1376.6528	1247.6102	1100.5418	986.4989	800.4196	672.3610	571.3133	415.2122	278.1533	150.0583
y++	*	1065.9528	984.4211	746.3435	688.8300	624.3088	550.7745	493.7531	400.7134	336.6841	286.1603	208.1097	139.5803	75.5328

De novo ms/ms analysis of purified product **12m**.

3.9. Characterization data for the reaction of peptide 13

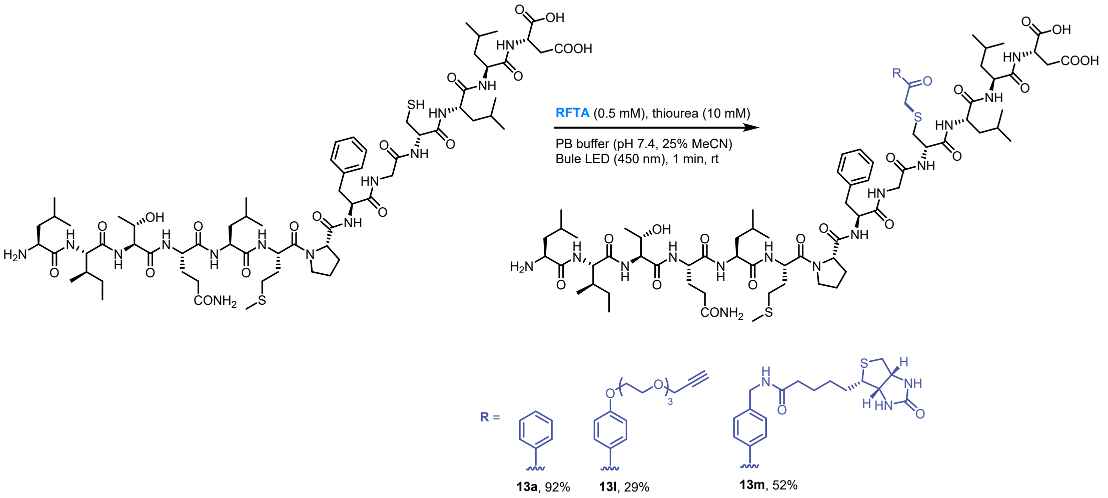

The reaction was followed **General procedure B** using peptide **13** (1 mM), sequence: NH2-LITQLMPFG<u>C</u>LLE, and sulfoxonium ylide **1a, 1l** and **1m**. A stock solution of peptide **13** (1 mM) was made up by 2 mL PB buffer (pH 7.4, 25% MeCN) and 2.9 mg peptide **13**.

3.9.1. Characterization of 13a

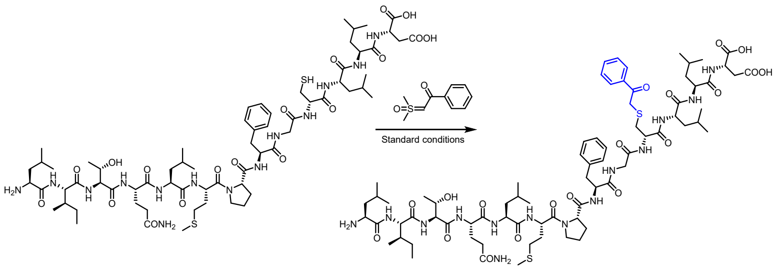

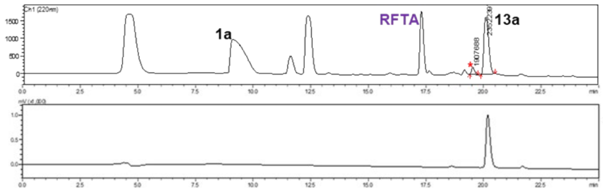

Peptide peak	Relative area
Substrate **13**	0
Target product **13a**	92%
By-product (disulfide) *	8%

LC trace of the reaction of peptide **13** and **1a** and purified product **13a**. (10-80% B phase over 25 min, 1 mL·min^{-1} flow rate, 0.1% TFA, λ = 220 nm, Kromasil 100-5-C18 4.6 × 250 mm, 5 μm column). The table refer to the normalized integration of the peptide peaks.

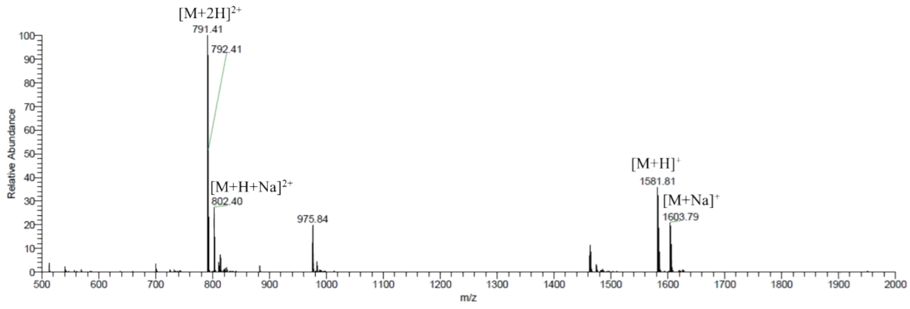

ESI Mass spectrum of purified product **13a**. Calculated Mass $[M+H]^+$: 1581.81; $[M+2H]^{2+}$: 791.41; Mass Found (ESI+) $[M+H]^+$: 1581.81; $[M+2H]^{2+}$: 791.41.

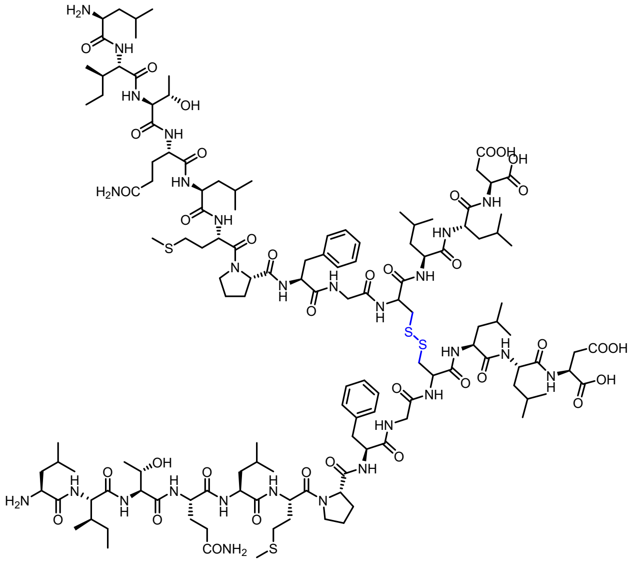

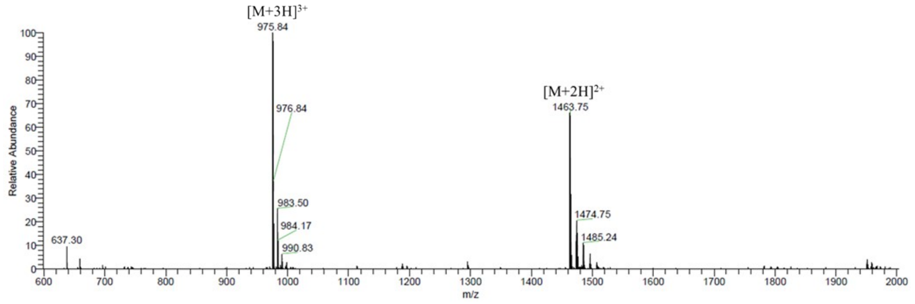

ESI Mass spectrum of oxidized by-product of **13** (disulfide). Calculated Mass $[M+H]^+$: 2924.50; $[M+2H]^{2+}$: 1463.75; $[M+3H]^{3+}$: 975.84; Mass Found (ESI+) $[M+H]^+$: 2924.50; $[M+2H]^{2+}$: 1463.75; $[M+3H]^{3+}$: 975.84.

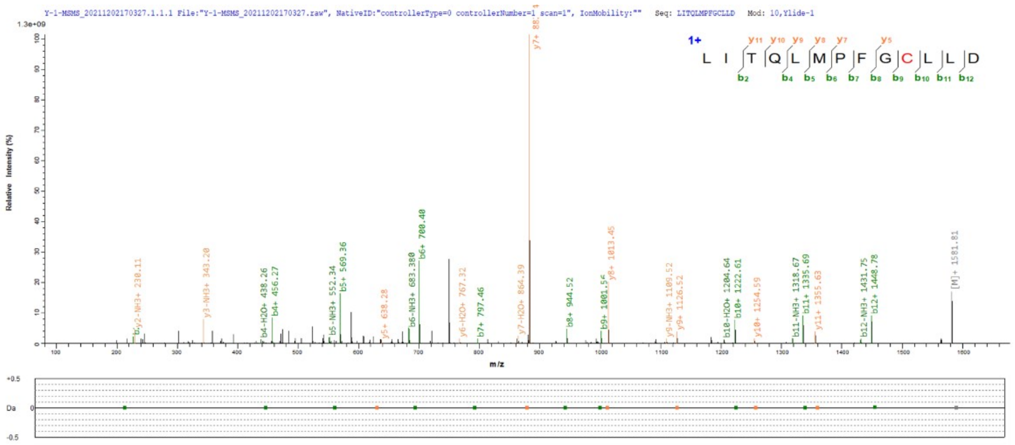

#	L(1/*)	I(2/12)	T(3/11)	Q(4/10)	L(5/9)	M(6/8)	P(7/7)	F(8/6)	G(9/5)	C(10/4)	L(11/3)	L(12/2)	D(*/1)
b+	114.0913	227.1754	328.2231	456.2817	569.3657	700.4062	797.4590	944.5274	1001.5488	1222.5999	1335.6840	1448.7680	*
b++	57.5493	114.0913	164.6152	228.6445	285.1865	350.7067	399.2331	472.7673	501.2781	611.8036	668.3456	724.8876	*
y+	*	1468.7215	1355.6374	1254.5897	1126.5311	1013.4471	882.4066	785.3538	638.2854	581.2640	360.2129	247.1288	134.0448
y++	*	734.8644	678.3223	627.7985	563.7692	507.2272	441.7069	393.1805	319.6463	291.1356	180.6101	124.0681	67.5260

De novo ms/ms analysis of purified product **13a**.

The MS/MS searching on other nucleophilic residues of product 13a

The MS/MS searching on T site:

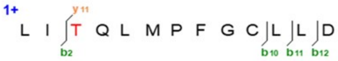

#	L(1/*)	I(2/12)	T(3/11)	Q(4/10)	L(5/9)	M(6/8)	P(7/7)	F(8/6)	G(9/5)	C(10/4)	L(11/3)	L(12/2)	D(*/1)
b+	114.0913	227.1754	446.2649	574.3235	687.4076	818.4481	915.5008	1062.5692	1119.5907	1222.5999	1335.6840	1448.7680	*
b++	57.5493	114.0913	223.6361	287.6654	344.2074	409.7277	458.2541	531.7883	560.2990	611.8036	668.3456	724.8876	*
y+	*	1468.7215	1355.6374	1136.5479	1008.4893	895.4052	764.3647	667.3120	520.2436	463.2221	360.2129	247.1288	134.0448
y++	*	734.8644	678.3223	568.7776	504.7483	448.2062	382.6860	334.1596	260.6254	232.1147	180.6101	124.0681	67.5260

The MS/MS searching on Q site:

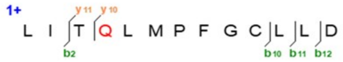

#	L(1/*)	I(2/12)	T(3/11)	Q(4/10)	L(5/9)	M(6/8)	P(7/7)	F(8/6)	G(9/5)	C(10/4)	L(11/3)	L(12/2)	D(*/1)
b+	114.0913	227.1754	328.2231	574.3235	687.4076	818.4481	915.5008	1062.5692	1119.5907	1222.5999	1335.6840	1448.7680	*
b++	57.5493	114.0913	164.6152	287.6654	344.2074	409.7277	458.2541	531.7883	560.2990	611.8036	668.3456	724.8876	*
y+	*	1468.7215	1355.6374	1254.5897	1008.4893	895.4052	764.3647	667.3120	520.2436	463.2221	360.2129	247.1288	134.0448
y++	*	734.8644	678.3223	627.7985	504.7483	448.2062	382.6860	334.1596	260.6254	232.1147	180.6101	124.0681	67.5260

The MS/MS searching on D site:

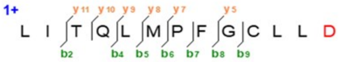

#	L(1/*)	I(2/12)	T(3/11)	Q(4/10)	L(5/9)	M(6/8)	P(7/7)	F(8/6)	G(9/5)	C(10/4)	L(11/3)	L(12/2)	D(*/1)
b+	114.0913	227.1754	328.2231	456.2817	569.3657	700.4062	797.4590	944.5274	1001.5488	1104.5580	1217.6421	1330.7262	*
b++	57.5493	114.0913	164.6152	228.6445	285.1865	350.7067	399.2331	472.7673	501.2781	552.7827	609.3247	665.8667	*
y+	*	1468.7215	1355.6374	1254.5897	1126.5311	1013.4471	882.4066	785.3538	638.2854	581.2640	478.2548	365.1707	252.0866
y++	*	734.8644	678.3223	627.7985	563.7692	507.2272	441.7069	393.1805	319.6463	291.1356	239.6310	183.0890	126.5470

In order to further confirm the potential functionalization on other nucleophilic residues, we have used the pLabel (v2.4) software[12] to automatically match the b-/y- ion fragments of products under the default parameters with a TOL (0.5 Da), the matching mass tolerance of fragment ions. As shown above, the analysis on the C site exhibited abundant MS fragments and the key fragment **b10** and **y5** were found. On the contrary, the analysis on the other nucleophilic sites exhibited a small number of fragments. Especially, the reliable match for site-adjacent key fragments were absent for these residues. Thus, the results demonstrated that the cysteine site is the only possible adducted position in product **13a**.

3.9.2. Characterization of 13l

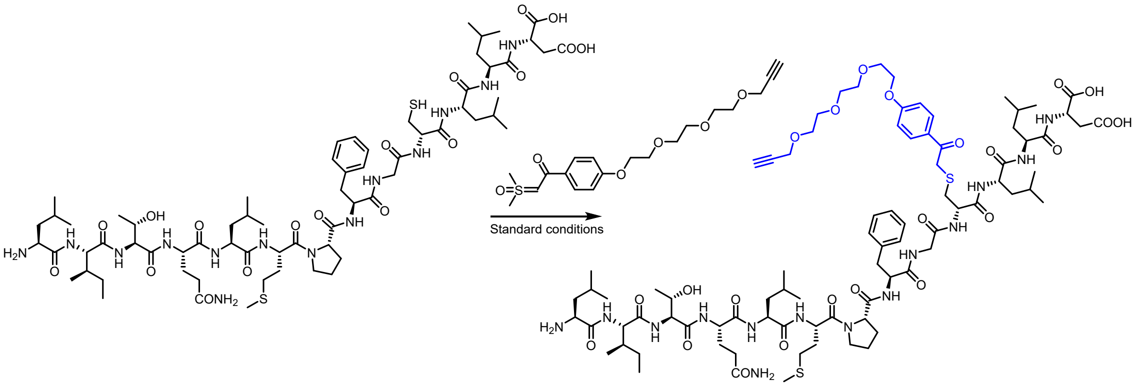

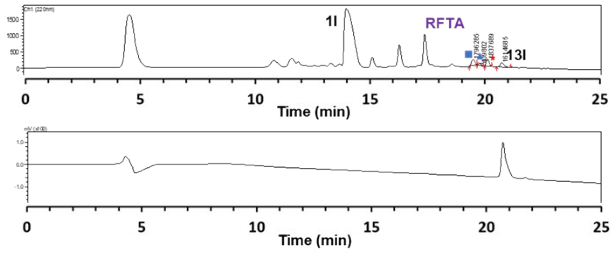

Peptide peak	Relative area
Substrate **13**	0
Target product **13l**	29%
By-product (disulfide) *	32%
By-product (**13** MS-102) ■	32%
By-product (**13** MS+190) ▲	7%

LC trace of the reaction of peptide **13** and **1l** and purified product **13l**. (10-80% B phase over 25 min, 1 mL·min^{-1} flow rate, 0.1% TFA, λ = 220 nm, Kromasil 100-5-C18 4.6 × 250 mm, 5 µm column). The table refer to the normalized integration of the peptide peaks.

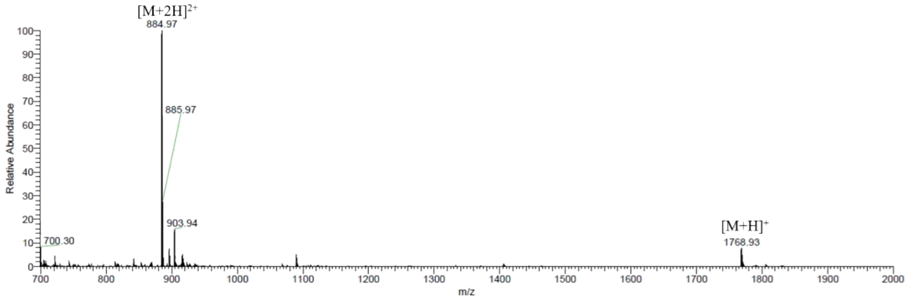

ESI Mass spectrum of purified product **13l**. Calculated Mass [M+H]$^{+}$: 1768.93; [M+2H]$^{2+}$: 884.97; Mass Found (ESI+) [M+H]$^{+}$: 1768.93; [M+2H]$^{2+}$: 884.97.

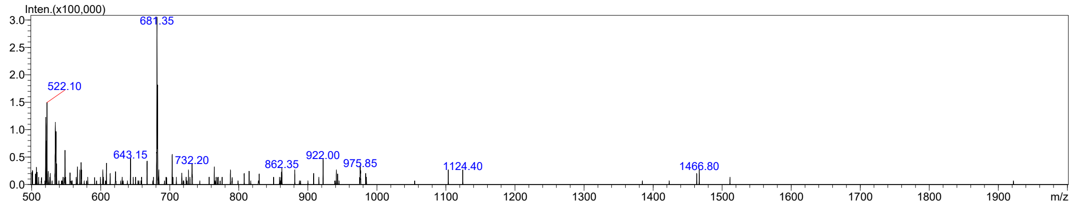

ESI Mass spectrum of by-product (**13** MS-102). Calculated Mass [M+H]$^{+}$: 1361.76; [M+2H]$^{2+}$: 681.38; Mass Found (ESI+) [M+2H]$^{2+}$: 681.35.

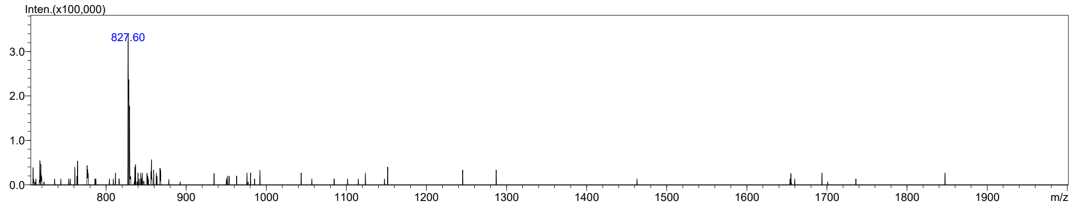

ESI Mass spectrum of by-product (**13** MS+190). Calculated Mass [M+H]$^{+}$: 1653.76; [M+2H]$^{2+}$: 827.38; Mass Found (ESI+) [M+2H]$^{2+}$: 827.60

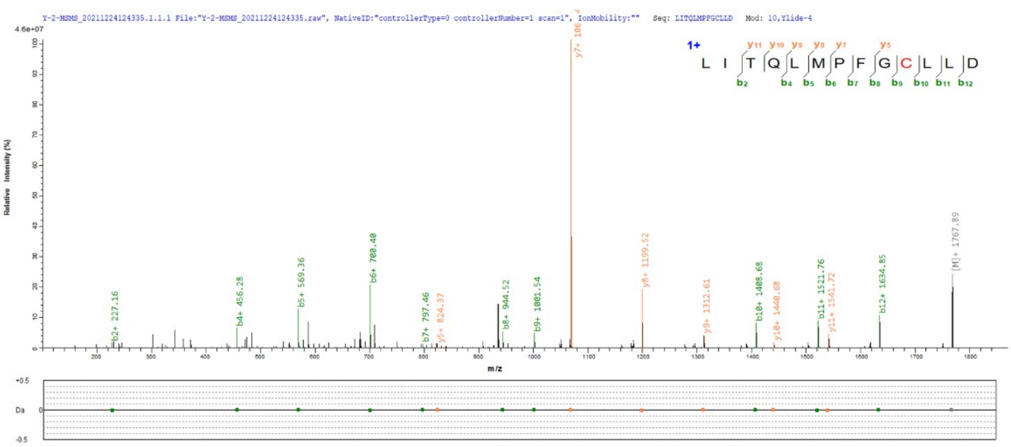

#	L(1/*)	I(2/12)	T(3/11)	Q(4/10)	L(5/9)	M(6/8)	P(7/7)	F(8/6)	G(9/5)	C(10/4)	L(11/3)	L(12/2)	D(*/1)
b+	114.0913	227.1754	328.2231	456.2817	569.3657	700.4062	797.4590	944.5274	1001.5488	1408.6891	1521.7732	1634.8572	*
b++	57.5493	114.0913	164.6152	228.6445	285.1865	350.7067	399.2331	472.7673	501.2781	704.8482	761.3902	817.9323	*
y+	*	1654.8107	1541.7266	1440.6789	1312.6203	1199.5363	1068.4958	971.4430	824.3746	767.3532	360.2129	247.1288	134.0448
y++	*	827.9090	771.3669	720.8431	656.8138	600.2718	534.7515	486.2252	412.6910	384.1802	180.6101	124.0681	67.5260

De novo ms/ms analysis of purified product **13l**.

3.9.3. Characterization of 13m

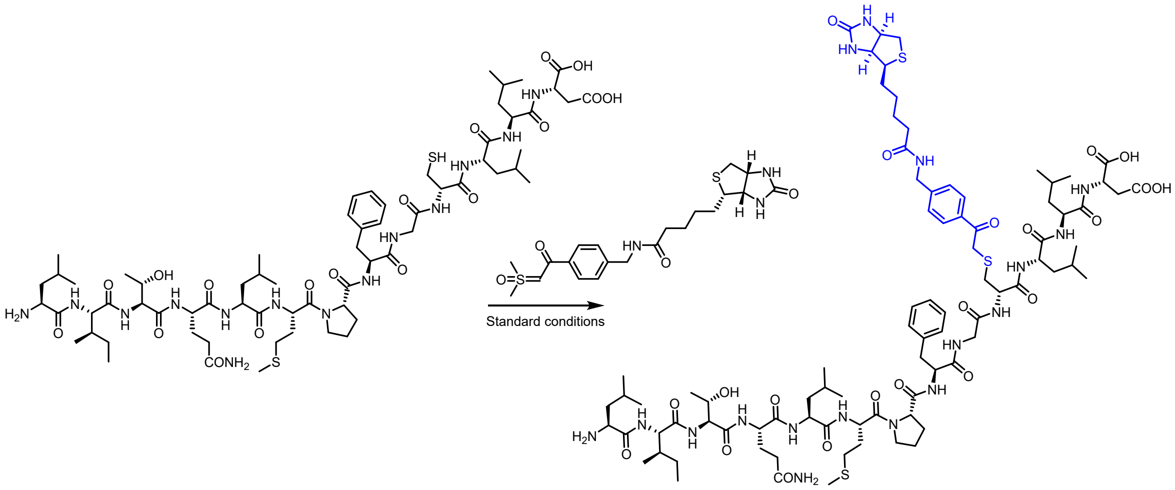

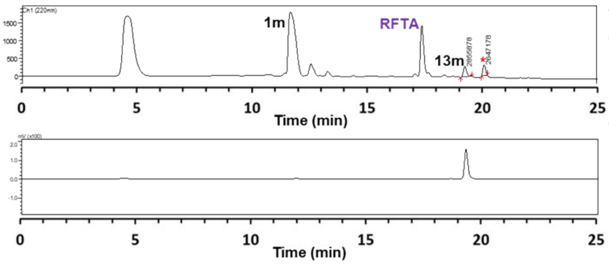

Peptide peak	Relative area
Substrate 13	0
Target product **13m**	52%
By-product (disulfide) *	48%

LC trace of the reaction of peptide **13** and **1m** and purified product **13m**. (10-80% B phase over 25 min, 1 mL·min^{-1} flow rate, 0.1% TFA, λ = 220 nm, Kromasil 100-5-C18 4.6 × 250 mm, 5 μm column). The table refer to the normalized integration of the peptide peaks.

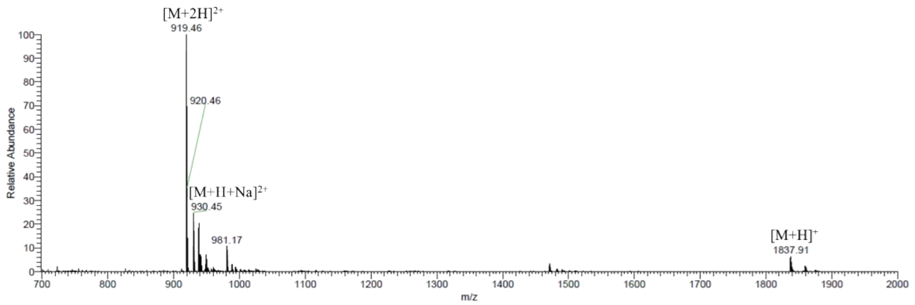

ESI Mass spectrum of purified product **13m**. Calculated Mass [M+H]$^{+}$: 1837.91; [M+2H]$^{2+}$: 919.46; Mass Found (ESI+) [M+H]$^{+}$: 1837.91; [M+2H]$^{2+}$: 919.46.

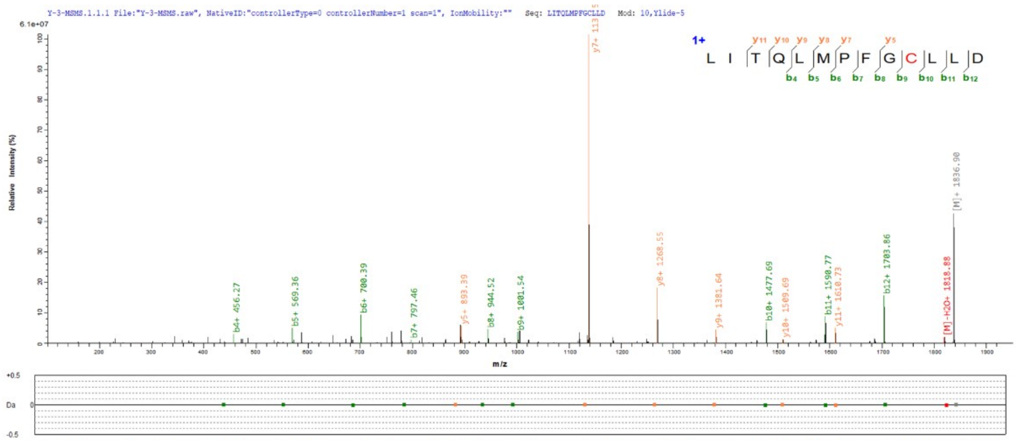

#	L(1/*)	I(2/12)	T(3/11)	Q(4/10)	L(5/9)	M(6/8)	P(7/7)	F(8/6)	G(9/5)	C(10/4)	L(11/3)	L(12/2)	D(*/1)
b+	114.0913	227.1754	328.2231	456.2817	569.3657	700.4062	797.4590	944.5274	1001.5488	1477.7040	1590.7881	1703.8722	*
b++	57.5493	114.0913	164.6152	228.6445	285.1865	350.7067	399.2331	472.7673	501.2781	739.3557	795.8977	852.4397	*
y+	*	1723.8256	1610.7415	1509.6939	1381.6353	1268.5512	1137.5107	1040.4580	893.3896	836.3681	360.2129	247.1288	134.0448
y++	*	862.4164	805.8744	755.3506	691.3213	634.7793	569.2590	520.7326	447.1984	418.6877	180.6101	124.0681	67.5260

De novo ms/ms analysis of purified product **13m**.

3.10. Characterization data for the reaction of peptide 14

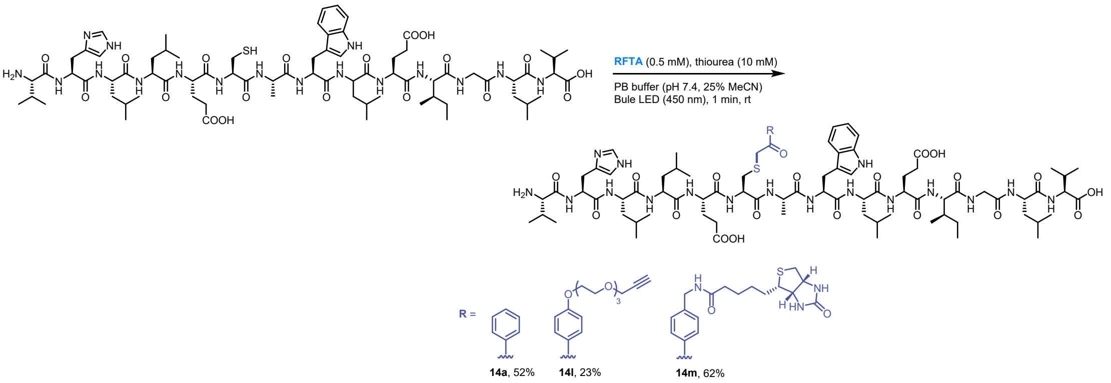

The reaction was followed **General procedure B** using peptide **14** (1 mM), sequence: NH2-VHLLECAWLEIGLV-OH, and sulfoxonium ylide **1a, 1l** and **1m**. A stock solution of peptide **14** (1 mM) was made up by 2 mL PB buffer (pH 7.4, 25% MeCN) and 3.2 mg peptide **14**.

3.10.1. Characterization of 14a

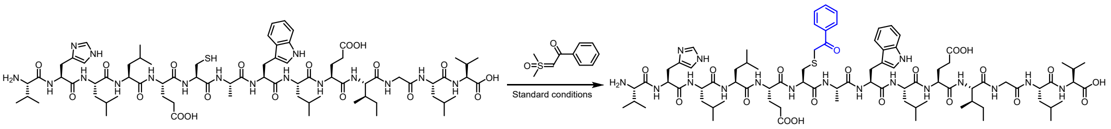

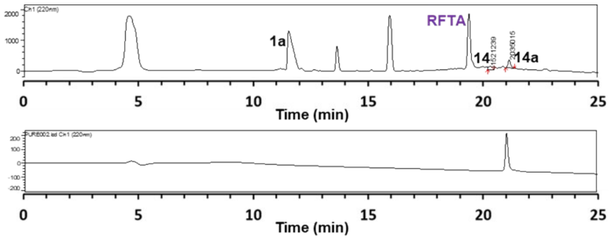

Peptide peak	Relative area
Substrate **14**	48%
Target product **14a**	52%
By-product (disulfide) *	0

LC trace of the reaction of peptide **14** and **1a** and purified product **14a**. (10-80% B phase over 25 min, 1 mL·min^{-1} flow rate, 0.1% TFA, λ = 220 nm, Kromasil 100-5-C18 4.6 × 250 mm, 5 μm column). The table refer to the normalized integration of the peptide peaks.

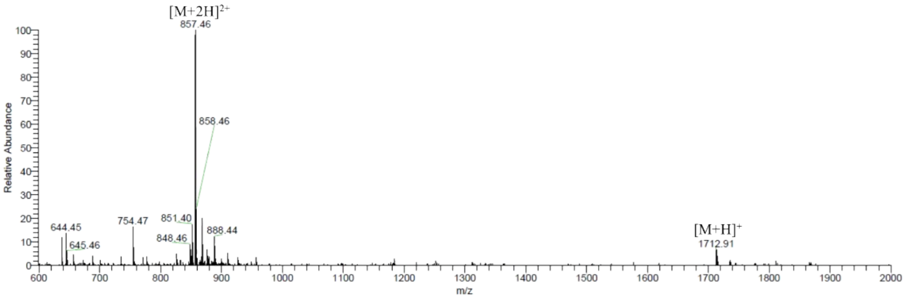

ESI Mass spectrum of purified product **14a**. Calculated Mass $[M+H]^+$: 1712.91; $[M+2H]^{2+}$: 857.46; Mass Found (ESI+) $[M+H]^+$: 1712.91; $[M+2H]^{2+}$: 857.46.

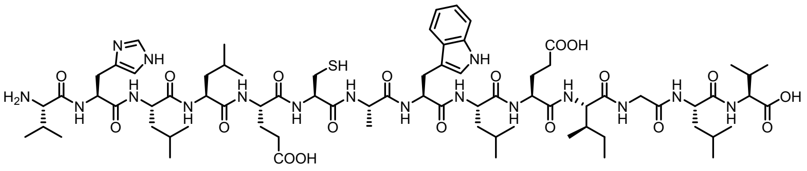

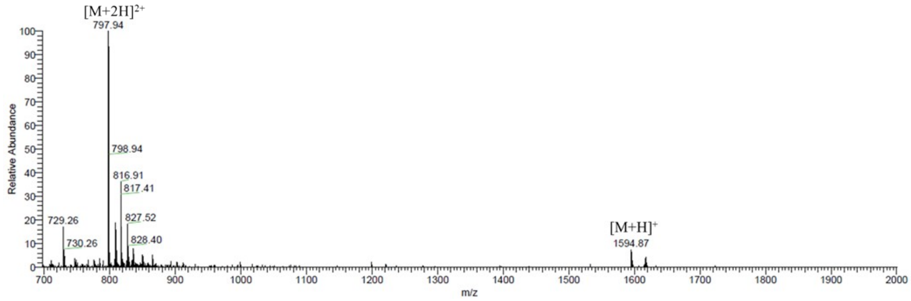

ESI Mass spectrum of the remaining **14**. Calculated Mass $[M+H]^{+}$: 1594.87; $[M+2H]^{2+}$: 797.94; Mass Found (ESI+) $[M+H]^{+}$: 1594.87; $[M+2H]^{2+}$: 797.94.

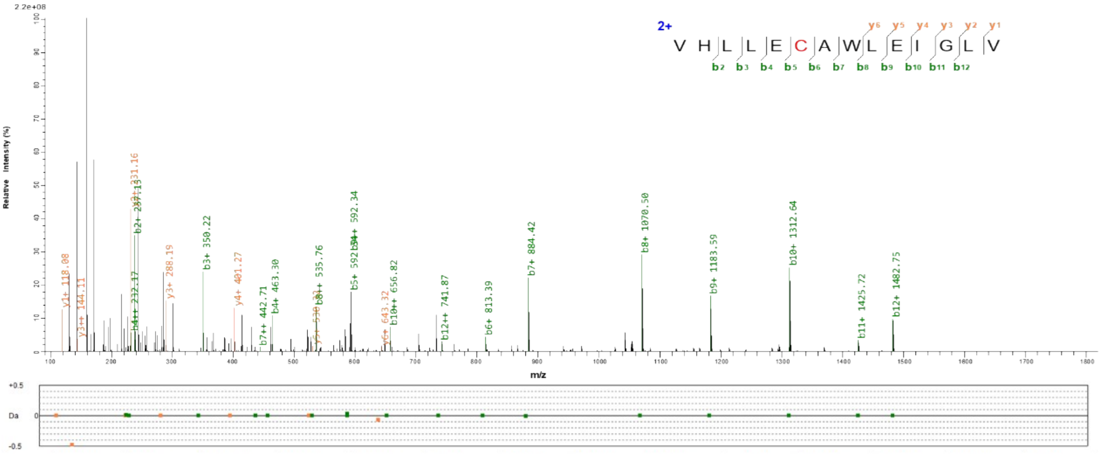

#	V(1/*)	H(2/13)	L(3/12)	L(4/11)	E(5/10)	C(6/9)	A(7/8)	W(8/7)	L(9/6)	E(10/5)	I(11/4)	G(12/3)	L(13/2)	V(*/1)
b+	100.0757	237.1346	350.2187	463.3027	592.3453	813.3964	884.4335	1070.5128	1183.5969	1312.6394	1425.7235	1482.7450	1595.8290	*
b++	50.5415	119.0709	175.6130	232.1550	296.6763	407.2018	442.7204	535.7600	592.3021	656.8234	713.3654	741.8761	798.4181	*
y+	*	1613.8396	1476.7807	1363.6966	1250.6125	1121.5700	900.5189	829.4818	643.4025	530.3184	401.2758	288.1918	231.1703	118.0863
y++	*	807.4234	738.8940	682.3519	625.8099	561.2886	450.7631	415.2445	322.2049	265.6629	201.1416	144.5995	116.0888	59.5468

De novo ms/ms analysis of purified product **14a**.

The MS/MS searching on other nucleophilic residues of product 14a

The MS/MS searching on T site:

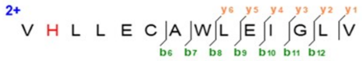

#	V(1/*)	H(2/13)	L(3/12)	L(4/11)	E(5/10)	C(6/9)	A(7/8)	W(8/7)	L(9/6)	E(10/5)	I(11/4)	G(12/3)	L(13/2)	V(*/1)
b+	100.0757	355.1765	468.2605	581.3446	710.3872	813.3964	884.4335	1070.5128	1183.5969	1312.6394	1425.7235	1482.7450	1595.8290	*
b++	50.5415	178.0919	234.6339	291.1759	355.6972	407.2018	442.7204	535.7600	592.3021	656.8234	713.3654	741.8761	798.4181	*
y+	*	1613.8396	1358.7388	1245.6548	1132.5707	1003.5281	900.5189	829.4818	643.4025	530.3184	401.2758	288.1918	231.1703	118.0863
y++	*	807.4234	679.8730	623.3310	566.7890	502.2677	450.7631	415.2445	322.2049	265.6629	201.1416	144.5995	116.0888	59.5468

The MS/MS searching on E5 site:

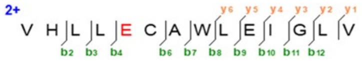

#	V(1/*)	H(2/13)	L(3/12)	L(4/11)	E(5/10)	C(6/9)	A(7/8)	W(8/7)	L(9/6)	E(10/5)	I(11/4)	G(12/3)	L(13/2)	V(*/1)
b+	100.0757	237.1346	350.2187	463.3027	710.3872	813.3964	884.4335	1070.5128	1183.5969	1312.6394	1425.7235	1482.7450	1595.8290	*
b++	50.5415	119.0709	175.6130	232.1550	355.6972	407.2018	442.7204	535.7600	592.3021	656.8234	713.3654	741.8761	798.4181	*
y+	*	1613.8396	1476.7807	1363.6966	1250.6125	1003.5281	900.5189	829.4818	643.4025	530.3184	401.2758	288.1918	231.1703	118.0863
y++	*	807.4234	738.8940	682.3519	625.8099	502.2677	450.7631	415.2445	322.2049	265.6629	201.1416	144.5995	116.0888	59.5468

The MS/MS searching on E10 site:

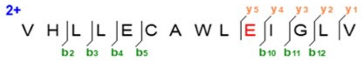

#	V(1/*)	H(2/13)	L(3/12)	L(4/11)	E(5/10)	C(6/9)	A(7/8)	W(8/7)	L(9/6)	E(10/5)	I(11/4)	G(12/3)	L(13/2)	V(*/1)
b+	100.0757	237.1346	350.2187	463.3027	592.3453	695.3545	766.3916	952.4709	1065.5550	1312.6394	1425.7235	1482.7450	1595.8290	*
b++	50.5415	119.0709	175.6130	232.1550	296.6763	348.1809	383.6994	476.7391	533.2811	656.8234	713.3654	741.8761	798.4181	*
y+	*	1613.8396	1476.7807	1363.6966	1250.6125	1121.5700	1018.5608	947.5237	761.4443	648.3603	401.2758	288.1918	231.1703	118.0863
y++	*	807.4234	738.8940	682.3519	625.8099	561.2886	509.7840	474.2655	381.2258	324.6838	201.1416	144.5995	116.0888	59.5468

In order to further confirm the potential functionalization on other nucleophilic residues, we have used the pLabel (v2.4) software[12] to automatically match the b-/y- ion fragments of products under the default parameters with a TOL (0.5 Da), the matching mass tolerance of fragment ions. As shown above, the analysis on the C site exhibited abundant MS fragments and the key fragment **b6** were found. On the contrary, the analysis on the other nucleophilic sites exhibited a small number of fragments. Especially, the reliable match for site-adjacent key fragments were absent for these residues. For example, even though the key fragments, **y5** (648.4), for E site were coincidently found, but the fragments between C6 and E10 were completely absent in the searching. Thus, the results demonstrated that the cysteine site is the only possible adducted position in product **14a**.

3.10.2. Characterization of 14l

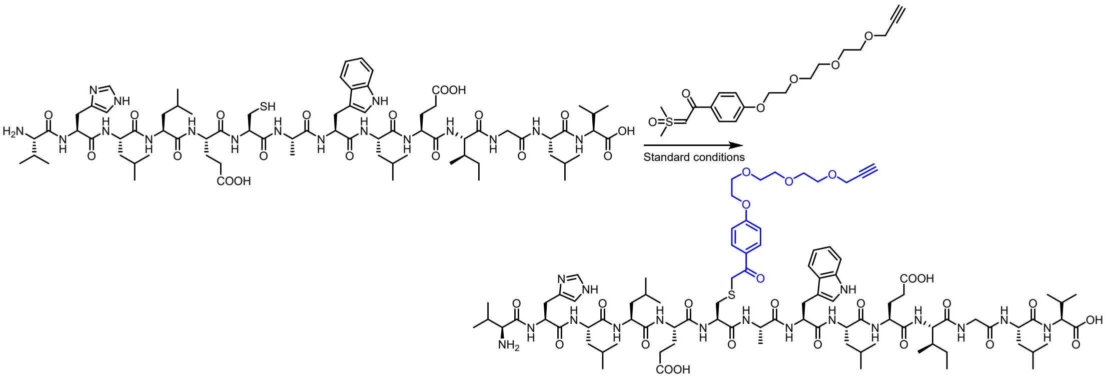

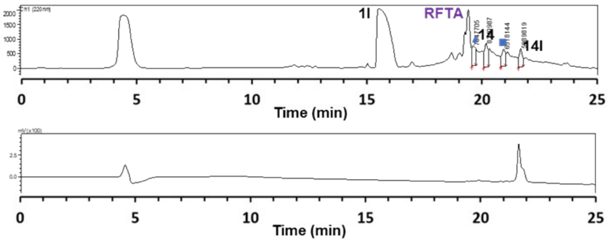

Peptide peak	Relative area
Substrate **14**	30%
Target product **14l**	23%
By-product (disulfide) *	0
By-product (**14** MS+21) ▲	26%
By-product (**14** MS+107) ■	21%

LC trace of the reaction of peptide **14** and **1l** and purified product **14l**. (10-80% B phase over 25 min, 1 mL·min^{-1} flow rate, 0.1% TFA, λ = 220 nm, Kromasil 100-5-C18 4.6 × 250 mm, 5 μm column). The table refer to the normalized integration of the peptide peaks.

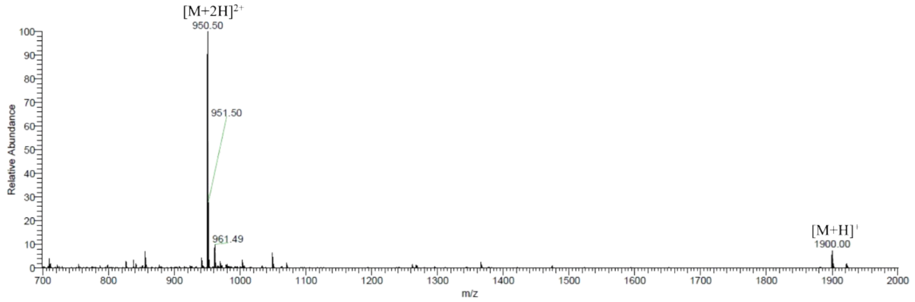

ESI Mass spectrum of purified product **14l**. Calculated Mass [M+H]$^{+}$: 1899.00; [M+2H]$^{2+}$: 950.50; Mass Found (ESI+) [M+H]$^{+}$: 1899.00; [M+2H]$^{2+}$: 950.50.

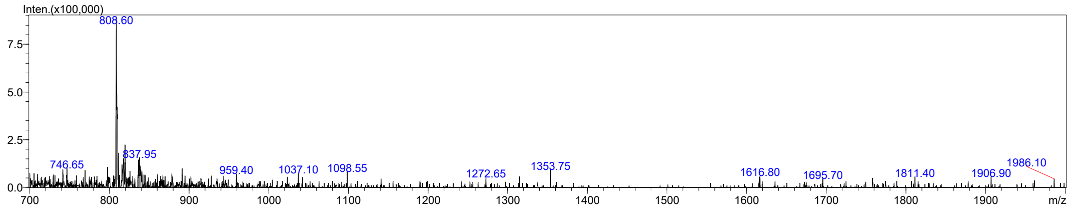

ESI Mass spectrum of by-product (**14** MS+21). Calculated Mass [M+H]$^{+}$: 1615.86; [M+2H]$^{2+}$: 808.43; Mass Found (ESI+) [M+2H]$^{2+}$: 808.60.

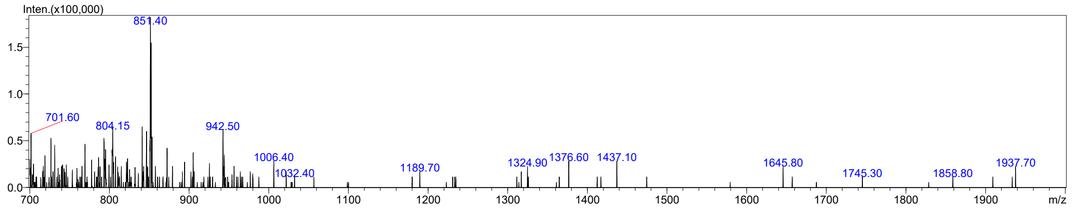

ESI Mass spectrum of by-product (**14** MS+107). Calculated Mass [M+H]$^{+}$: 1701.86; [M+2H]$^{2+}$: 851.43; Mass Found (ESI+) [M+2H]$^{2+}$: 851.40.

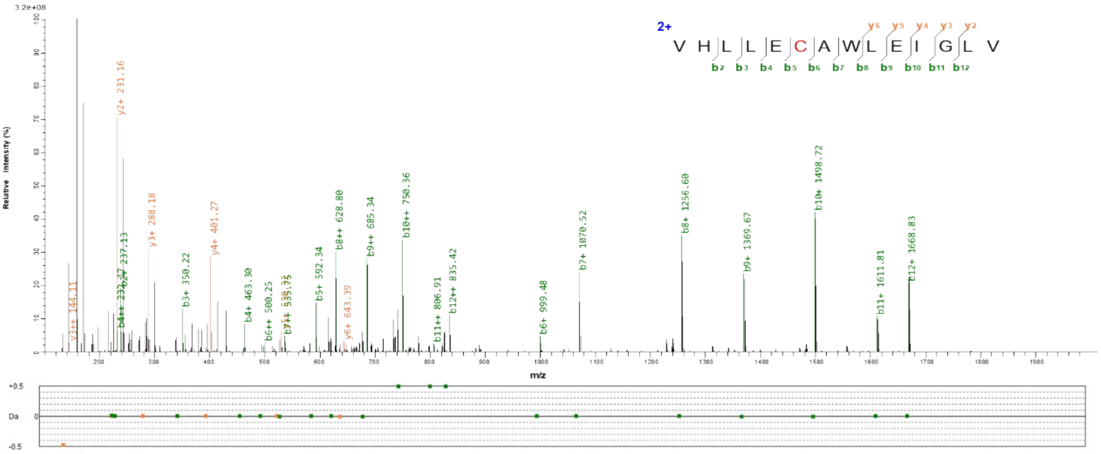

#	V(1/*)	H(2/13)	L(3/12)	L(4/11)	E(5/10)	C(6/9)	A(7/8)	W(8/7)	L(9/6)	E(10/5)	I(11/4)	G(12/3)	L(13/2)	V(*/1)
b+	100.0757	237.1346	350.2187	463.3027	592.3453	999.4856	1070.5227	1256.6020	1369.6861	1498.7286	1611.8127	1668.8342	1781.9182	*
b++	50.5415	119.0709	175.6130	232.1550	296.6763	500.2464	535.7650	628.8046	685.3467	749.8680	806.4100	834.9207	891.4628	*
y+	*	1799.9288	1662.8699	1549.7858	1436.7018	1307.6592	900.5189	829.4818	643.4025	530.3184	401.2758	288.1918	231.1703	118.0863
y++	*	900.4680	831.9386	775.3965	718.8545	654.3332	450.7631	415.2445	322.2049	265.6629	201.1416	144.5995	116.0888	59.5468

De novo ms/ms analysis of purified product **14l**.

3.10.3. Characterization of 14m

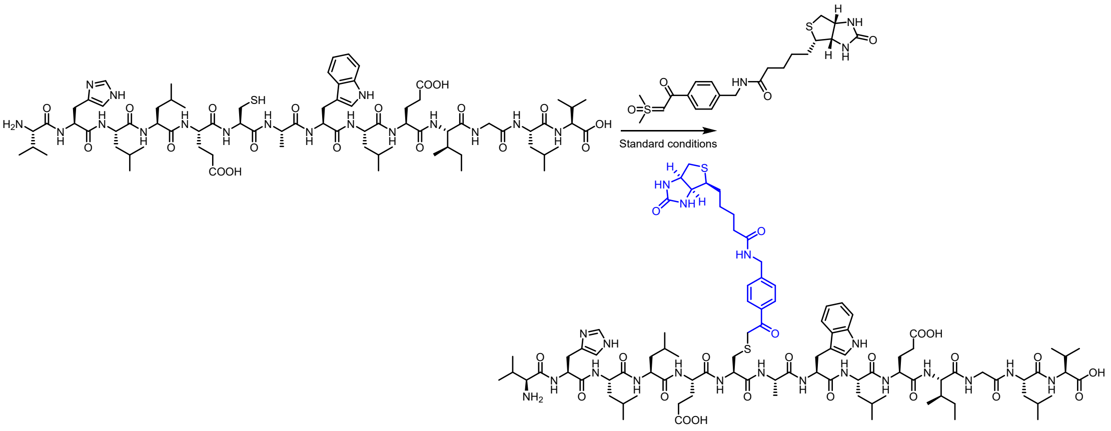

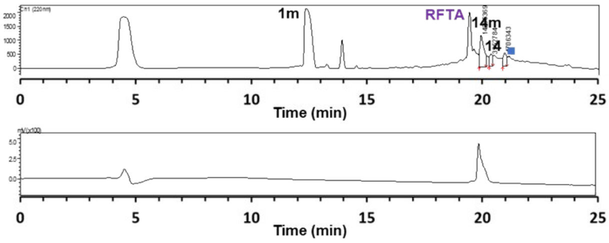

Peptide peak	Relative area
Substrate **14**	21%
Target product **14m**	62%
By-product (disulfide) *	0
By-product (**14** MS+107) ■	17%

LC trace of the reaction of peptide **14** and **1m** and purified product **14m**. (10-80% B phase over 25 min, 1 mL·min^{-1} flow rate, 0.1% TFA, λ = 220 nm, Kromasil 100-5-C18 4.6 × 250 mm, 5 µm column). The table refer to the normalized integration of the peptide peaks.

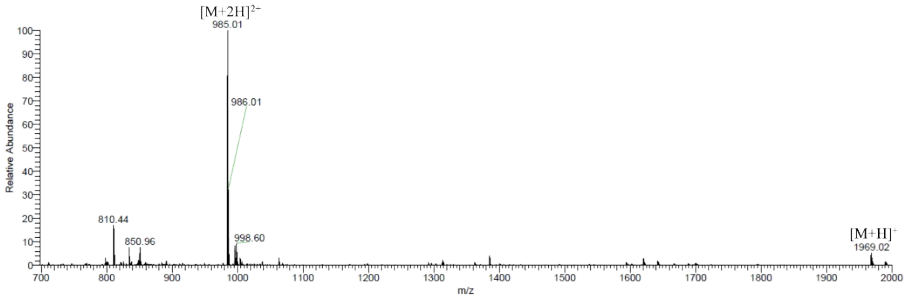

ESI Mass spectrum of purified product **14m**. Calculated Mass [M+H]$^{+}$: 1968.01; [M+2H]$^{2+}$: 985.01; Mass Found (ESI+) [M+H]$^{+}$: 1968.01; [M+2H]$^{2+}$: 985.01.

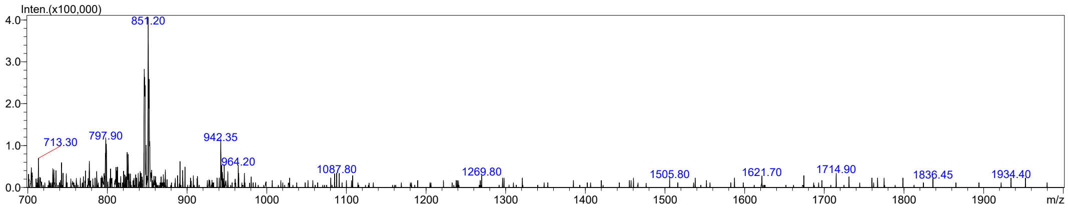

ESI Mass spectrum of by-product (**14** MS+107). Calculated Mass [M+H]$^{+}$: 1701.86; [M+2H]$^{2+}$: 851.43; Mass Found (ESI+) [M+2H]$^{2+}$: 851.20.

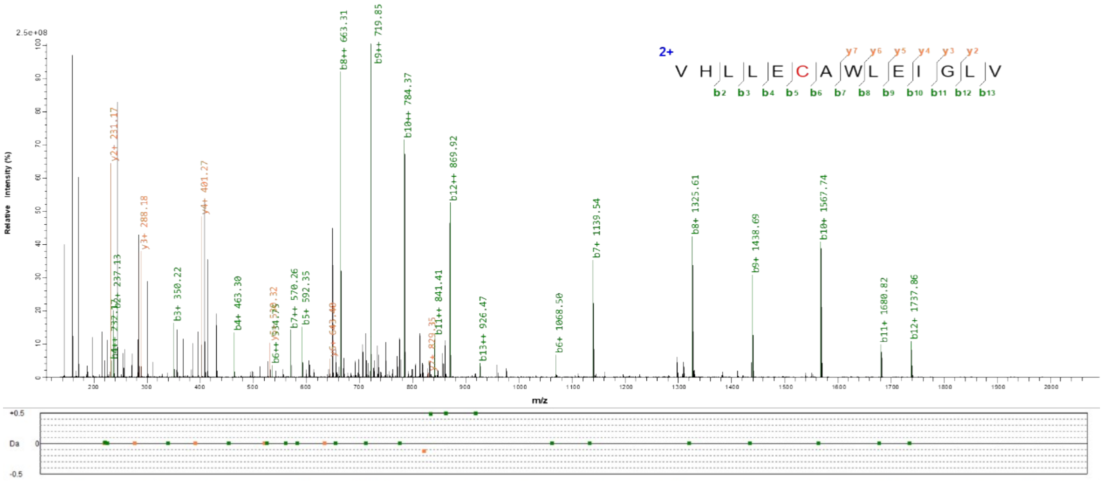

#	V(1/*)	H(2/13)	L(3/12)	L(4/11)	E(5/10)	C(6/9)	A(7/8)	W(8/7)	L(9/6)	E(10/5)	I(11/4)	G(12/3)	L(13/2)	V(*/1)
b+	100.0757	237.1346	350.2187	463.3027	592.3453	1068.5005	1139.5376	1325.6169	1438.7010	1567.7436	1680.8276	1737.8491	1850.9332	*
b++	50.5415	119.0709	175.6130	232.1550	296.6763	534.7539	570.2725	663.3121	719.8541	784.3754	840.9175	869.4282	925.9702	*
y+	*	1868.9437	1731.8848	1618.8008	1505.7167	1376.6741	900.5189	829.4818	643.4025	530.3184	401.2758	288.1918	231.1703	118.0863
y++	*	934.9755	866.4460	809.9040	753.3620	688.8407	450.7631	415.2445	322.2049	265.6629	201.1416	144.5995	116.0888	59.5468

De novo ms/ms analysis of purified product **14m**.

3.11. Characterization data for the reaction of peptide I-III

3.11.1. The reaction peptide I

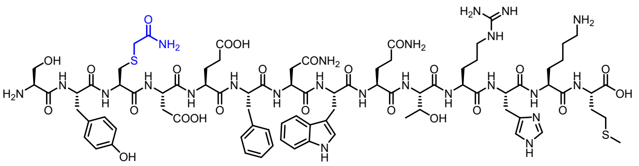

The reaction was followed **General procedure B** using peptide **I** (1 mM), sequence: NH_2-SYC(IAM)DEFNWQTRHKM-OH, and sulfoxonium ylide **1a**. A stock solution of peptide **I** (1 mM) was made up by 1 mL PB buffer (pH 7.4, 25% MeCN) and 1.9 mg peptide **I**.

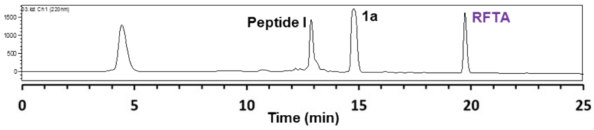

LC trace of the reaction of peptide **I** and **1a**. (10-70% B phase over 25 min, 1 mL·min^{-1} flow rate, 0.1% TFA, λ = 220 nm, Inertsil ODS-SP 4.6 × 250 mm, 5 µm column).

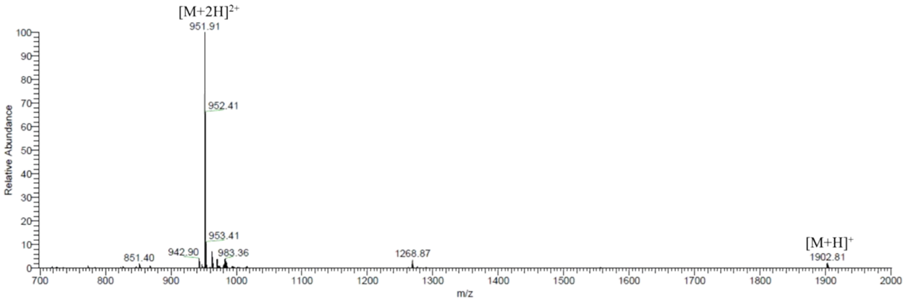

ESI Mass spectrum of remaining peptide I. Calculated Mass $[M+H]^+$: 1901.80; $[M+2H]^{2+}$: 951.41; Mass Found (ESI+) $[M+H]^+$: 1902.81; $[M+2H]^{2+}$: 951.91.

3.11.2. The reaction peptide II

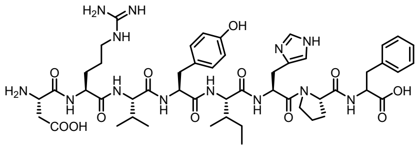

The reaction was followed **General procedure B** using peptide **II** (1 mM), sequence: NH_2-DRVYIHPF-OH, and sulfoxonium ylide **1a**. A stock solution of peptide **II** (1 mM) was made up by 1 mL PB buffer (pH 7.4, 25% MeCN) and 1.1 mg peptide **II**.

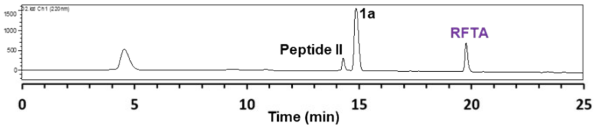

LC trace of the reaction of peptide **I** and **1a**. (10-70% B phase over 25 min, 1 mL·min^{-1} flow rate, 0.1% TFA, λ = 220 nm, Inertsil ODS-SP 4.6 × 250 mm, 5 µm column).

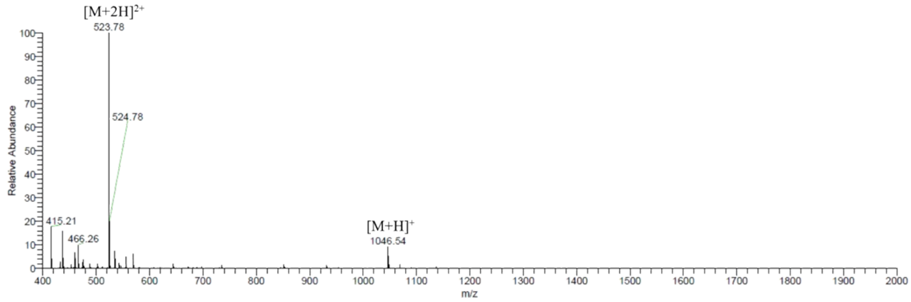

ESI Mass spectrum of remaining peptide I. Calculated Mass [M+H]$^+$: 1901.80; [M+2H]$^{2+}$: 951.41; Mass Found (ESI+) [M+H]$^+$: 1902.81; [M+2H]$^{2+}$: 951.91.

3.11.3. The reaction peptide III

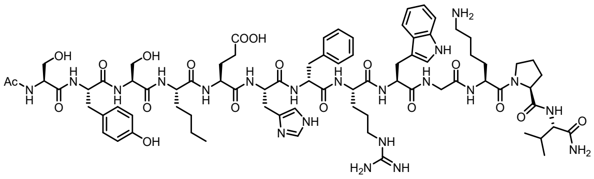

The reaction was followed **General procedure B** using peptide **III** (1 mM), sequence: Ac-SYSNleEHfRWKPV-NH_2, and sulfoxonium ylide **1a**. A stock solution of peptide **III** (1 mM) was made up by 1 mL PB buffer (pH 7.4, 25% MeCN) and 1.6 mg peptide **III**.

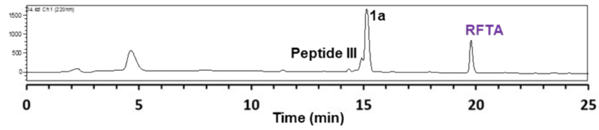

LC trace of the reaction of peptide **I** and **1a**. (10-70% B phase over 25 min, 1 mL·min^{-1} flow rate, 0.1% TFA, λ = 220 nm, Inertsil ODS-SP 4.6 × 250 mm, 5 µm column).

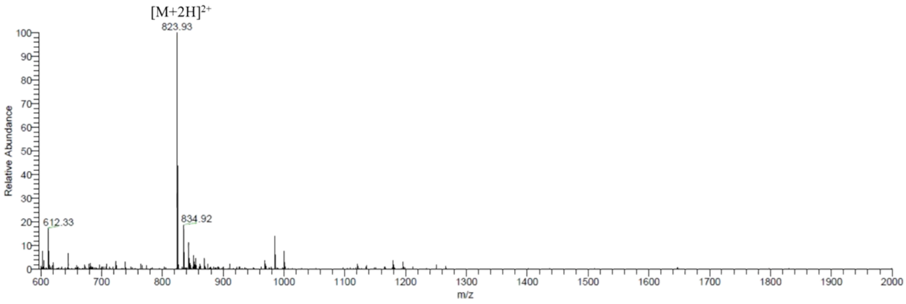

ESI Mass spectrum of remaining peptide I. Calculated Mass [M+H]$^+$: 1901.80; [M+2H]$^{2+}$: 951.41; Mass Found (ESI+) [M+H]$^+$: 1902.81; [M+2H]$^{2+}$: 951.91.

4. Mechanistic experiments

4.1. Luminescence screening

The luminescence screening was performed by following a similar procedure of previous report[13]. In order to consistent with the reaction conditions, PB buffer (pH 7.4, 25% MeCN) was used as the solvent for luminescence experiments. A photocatalyst concentration of 20 μM was used throughout the experiments along with substrate concentrations of 20 mM, which equates to 1000 equivalents of each potential quencher relative to the photocatalyst. The luminescence emission spectrum of each sample excited at 420 nm was measured twice and an average was taken. The emission intensity (I) at a pre-defined wavelength was noted and compared with that of the photocatalyst in isolation (I_0). The amount of decrease in the emission intensity was then quantified as a "quenching percentage" (F) defined by the following formula:

$$F(\%) = 100\left(1 - \frac{I}{I_0}\right)\% \qquad \text{Equation 1}$$

The structure of the photocatalysts employed in this study are shown in **Figure S2**. The results were listed in Table **S1** and Figure **S10** to compare with the reaction conversions of **1a** and peptide **7**. The luminescence spectra were summarized in **Figure S3**.

4.2. Stern-Volmer luminescence quenching studies

Stern-Volmer luminescence quenching studies were carried out using a 20 μM solution of RFTA and variable concentrations of sulfoxonium ylide **1a** and Ac-Cys-OH **2** in PB buffer (pH 7.4, 25% MeCN). Two independent duplications were performed for two substrates. The solutions were irradiated at 420 nm and the luminescence was measured at 530 nm (I_0 = emission intensity of the photocatalyst in isolation at the specified wavelength; I = observed intensity as a function of the quencher concentration). The luminescence spectra and were summarized in **Figure S7**.

4.3. UV/vis absorption studies

UV/vis absorption spectra of the starting materials were observed in PB buffer (pH 7.4, 25% MeCN): RFTA (20 uM), sulfoxonium ylide **1a** (20 mM) and Ac-Cys-OH **2** (20 mM). The photocatalyst RFTA presented a maximum absorption at 450 nm in visible light region, and the substrates **1a** and **2** didn't show any absorption in visible light region as shown in **Figure S8**. Therefore, the photocatalysts were the only absorbing species within the reaction mixture.

4.4. Cyclic Voltammogram

Cycle voltammetry (CV) measurements were carried out using an electrochemical workstation (CHI600E).
Insets show the reference of Fc/Fc^{+}

1a HOMO
Init E (V) = 0
High E (V) = 1.7
Low E (V) = 0
Init P/N = P
Scan Rate (V/s) = 0.1
Segment = 2
Sample Interval (V) = 0.001
Quiet Time (sec) = 2

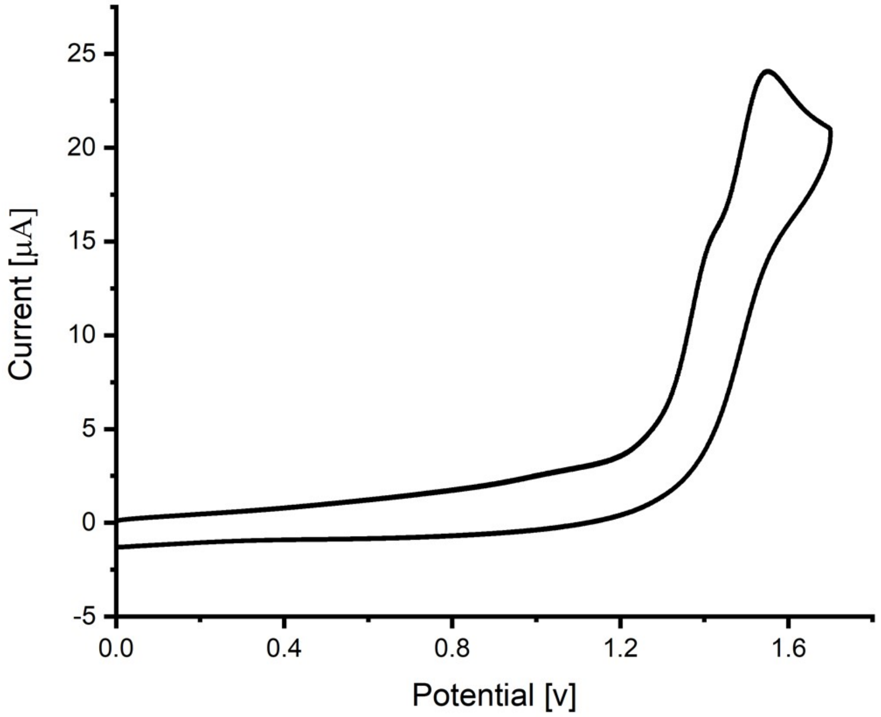

RFTA HOMO
Init E (V) = 0
High E (V) = 2.6
Low E (V) = 0
Init P/N = P
Scan Rate (V/s) = 0.1
Segment = 2
Sample Interval (V) = 0.001
Quiet Time (sec) = 2

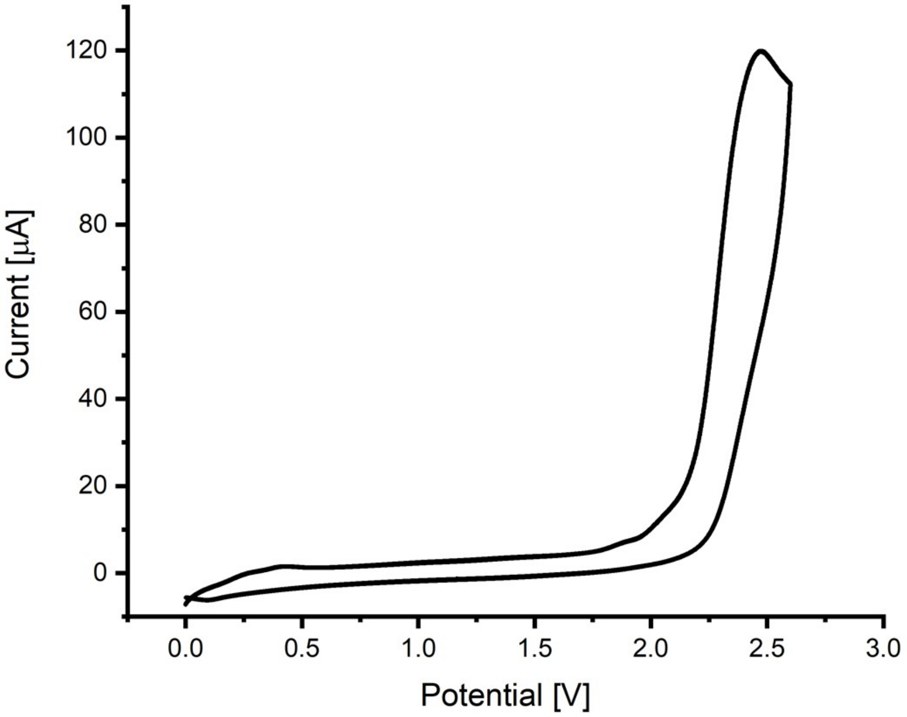

5. Supplementary material and information for protein modification

5.1. General information

All chemical reagents are commercially available from *Energy Chemical* without purification. **High-Resolution Mass Spectrometry (HRMS)** and **MS/MS** analysis were measured on a Q_Exactive_Focus.

5.2. General procedure C for the reaction of protein and proteome

A 20 mM MeCN stock solution of RFTA was made up of 5.7 mg RFTA (0.01 mmol) and 0.5 mL MeCN, a 1 M water stock solution of thiourea was made up of 76 mg thiourea (1 mmol) and 1 mL deionized water, and a 100 mM water stock solution of sulfoxonium ylide was made up of specific sulfoxonium ylide (0.1 mmol) and 1 mL deionized water. These stock solutions were stored at room temperature away from light. To a 2 mL vial was added 200 uL specific protein (50 μM) or cell lysate (2 mg/mL) PB buffer (pH 7.4) solution and 0.4 μL RFTA (20 mM), 10 μL specific sulfoxonium ylide (100 mM) and 2 μL thiourea (1 M) stock solution. The vial was then caped and equipped with magnetic bar. The sealed vial was degassed and refilled with nitrogen. The reaction was stirred and irradiated with 450 nm LED light (40 W) for 10 s, as shown in the following figure. The resulting solution was then subjected for the analyzation of ESI-TOF MS, MS/MS as well as Western-blotting.

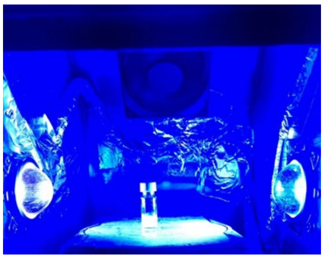

5.3. LC-MS analysis

Ultrafiltration centrifuge tubes are wetting before use by MilliQ water. The 50 μL resulting solution for the reaction of BSA was added to ultrafiltration centrifuge tubes for the analyzation of ESI-TOF MS, respectively. The tubes were washing three times with 200 μL MilliQ water to remove residue small molecule weight substrates by centrifugation with 5000 rpm. The protein concentration was determined by Nanodrop and waiting for ESI-TOF MS analysis.

5.4. LC-MS/MS analysis

The 50 μL resulting solution for BSA was added to 1.5 ml tube and 5 μL 5× loading buffer was added to this mixture following by in-gel SDS-PAGE scanning. The gel was stained by coomassie blue and destained by decolorant. The protein lane was cut in to pieces, washed by ddH_2O and decolorized. Cuticle was subjected to reduction and alkylation with 10 mM DTT at 37 ℃ for 30

min and 10 mM IAA in the dark at room temperature for 30 min. The mixture was washed with 100μL ddH_2O, dehydrated by 50% acetonitrile, 100% acetonitrile and immersed with NH_4HCO_3 solution containing 1 μg trypsin overnight 17 hours. The supernatant was collected by centrifugation (1400 g, 3 min) and combined with 2 × 50 μL extraction liquid after incubating for 30 min, add 5% (v/v) formic acid to stop digest, stored at -80 ℃ and desalted for LC-MS/MS analysis.

6. Supplementary material and information for chemoproteomics analysis

6.1. Western blot

The probe **1m** incubated with BSA, Hela and MCF7 cell lysate under the above modified reaction condition, respectively, samples without **1m** as control. Loading buffer was added to these samples following by in-gel SDS-PAGE (10% acrylamide, Marker: MIKX^{TM} Tris-Glycine 4-20% DB245 10-245 kD) and electrophoresis transfer. The nitrocellulose filter membrane was blocked by 5% milk for 1 hour and incubated with anti-biotin antibody (rabbit) for 3 hours at room temperature. The nitrocellulose filter membrane was scanned with e-BLOT by chemiluminescence.

6.2. MS-based proteomics

The harvested cells were lysed by sonication (1s work, 1s break,10 pulses, 50% duty cycle, output setting = 3). The soluble (supernatant) and membrane (pellet) was separated by centrifugation (12000rpm, 10 min × 2 times, 4 ˚C). Protein concentration was determined by BCA assay. Each samples contained 2 mg proteomes and was treated with biotinylated sulfoxonium ylide (5 mM) in vitro for 10 seconds 450 nm light irradiation, then cold methanol (volume ratio = 1:3) was added to the above solution and appeared precipitation in a short time. The precipitation protein was separated by centrifugation at 4 ˚C, 14,000 rpm for 10 min and wash twice by 300 μL methanol. The precipitation followed by solubilized in 300 μL PBS containing 1.2% SDS by sonication then dilute to 0.2% SDS in PBS. A volume of 50 μL of streptavidin-agarose beads slurry was added to the solution and incubated for 3 hours at 29 ˚C. After incubation, the beads were pelleted by centrifugation and washed (3 × 1 mL cold PBS and 3 × 1 mL cold water) and resuspended in PBS containing 6 M urea. Subsequently, 10 mM DTT was added to the beads containing solution and incubated at 37 ˚C for 30 min and 20 mM IAM was added and reacted at 37 ˚C for 30 min in dark. The bead mixture was diluted with PBS containing 2 M urea and incubated with 2 μg trypsin overnight. The system was acidified to a final concentration of 2% (v/v) formic acid and the beads were further washed (3 × 0.6 mL cold PBS and 3 × 1 mL cold water) to remove the unmodified peptides. The beads were then incubated with 60 μL washing buffer (80% acetonitrile, 0.1% formic acid in water) for 10min at 25 ˚C and for 10min at 72 ˚C, respectively, to release probe-modified peptides. Combined the washing buffer dried for LC-MS/MS analysis.

7. Reference

1. Mangion, I. K.; Nwamba, I. K.; Shevlin, M.; Huffman, M. A., Iridium-catalyzed X-H insertions of sulfoxonium ylides. *Org. Lett.* **2009,** *11* (16), 3566-3569.
2. Mangion, I. K.; Weisel, M., Gold (I) catalysis of X–H bond insertions. *Tetrahedron Lett.* **2010,** *51* (41), 5490-5492.
3. Vaitla, J.; Hopmann, K. H.; Bayer, A., Rhodium-Catalyzed Synthesis of Sulfur Ylides via in Situ Generated Iodonium Ylides. *Org. Lett.* **2017,** *19* (24), 6688-6691.
4. Li, J.; He, H.; Huang, M.; Chen, Y.; Luo, Y.; Yan, K.; Wang, Q.; Wu, Y., Iridium-Catalyzed B-H Bond Insertion Reactions Using Sulfoxonium Ylides as Carbene Precursors toward alpha-Boryl Carbonyls. *Org. Lett.* **2019,** *21* (22), 9005-9008.
5. Furniel, L. G.; Burtoloso, A. C. B., Copper-catalyzed N–H insertion reactions from sulfoxonium ylides. *Tetrahedron* **2020,** *76* (51), 131313.
6. Mu, Y.; Chen, Y.; Gao, Y.; Sun, J.; Iqbal, Z.; Wan, Y.; Yang, M.; Yang, Z.; Tang, D., Ru-Catalyzed O-H Insertion of Sulfoxonium Ylide and Carboxylic Acid to Synthesize α-Acyloxy Ketones. *ChemistrySelect* **2020,** *5* (5), 1705-1708.
7. Dias, R. M.; Burtoloso, A. C., Catalyst-Free Insertion of Sulfoxonium Ylides into Aryl Thiols. A Direct Preparation of beta-Keto Thioethers. *Org. Lett.* **2016,** *18* (12), 3034-3037.
8. Abberley, J. P.; Killah, R.; Walker, R.; Storey, J. M. D.; Imrie, C. T.; Salamonczyk, M.; Zhu, C.; Gorecka, E.; Pociecha, D., Heliconical smectic phases formed by achiral molecules. *Nat. Commun.* **2018,** *9* (1), 228.
9. Barday, M.; Janot, C.; Halcovitch, N. R.; Muir, J.; Aissa, C., Cross-Coupling of alpha-Carbonyl Sulfoxonium Ylides with C-H Bonds. *Angew. Chem. Int. Ed. Engl.* **2017,** *56* (42), 13117-13121.
10. Truce, W. E.; Madding, G. D., Sulfone-stabilized oxosulfonuim ylides. *Tetrahedron Lett.* **1966,** *7* (31), 3681-3687.
11. Sachse, F.; Gebauer, K.; Schneider, C., Continuous Flow Synthesis of 2H-Thiopyrans via thia-Diels–Alder Reactions of Photochemically Generated Thioaldehydes. *Eur. J. Org. Chem.* **2020,** *2021* (1), 64-71.
12. Wang, L. H.; Li, D. Q.; Fu, Y.; Wang, H. P.; Zhang, J. F.; Yuan, Z. F.; Sun, R. X.; Zeng, R.; He, S. M.; Gao, W., pFind 2.0: a software package for peptide and protein identification via tandem mass spectrometry. *Rapid Commun Mass Spectrom* **2007,** *21* (18), 2985-91.
13. Teders, M.; Henkel, C.; Anhauser, L.; Strieth-Kalthoff, F.; Gomez-Suarez, A.; Kleinmans, R.; Kahnt, A.; Rentmeister, A.; Guldi, D.; Glorius, F., The energy-transfer-enabled biocompatible disulfide-ene reaction. *Nat. Chem.* **2018,** *10* (9), 981-988.

8. NMR spectra

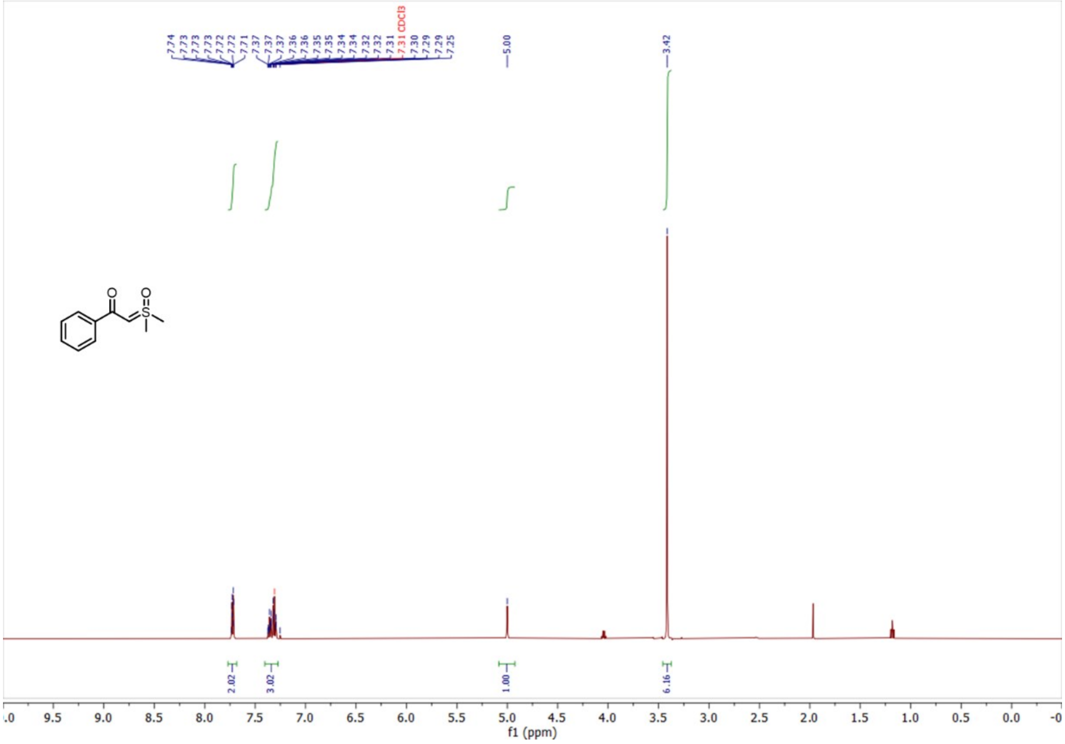

^{1}H NMR spectrum of **1a**

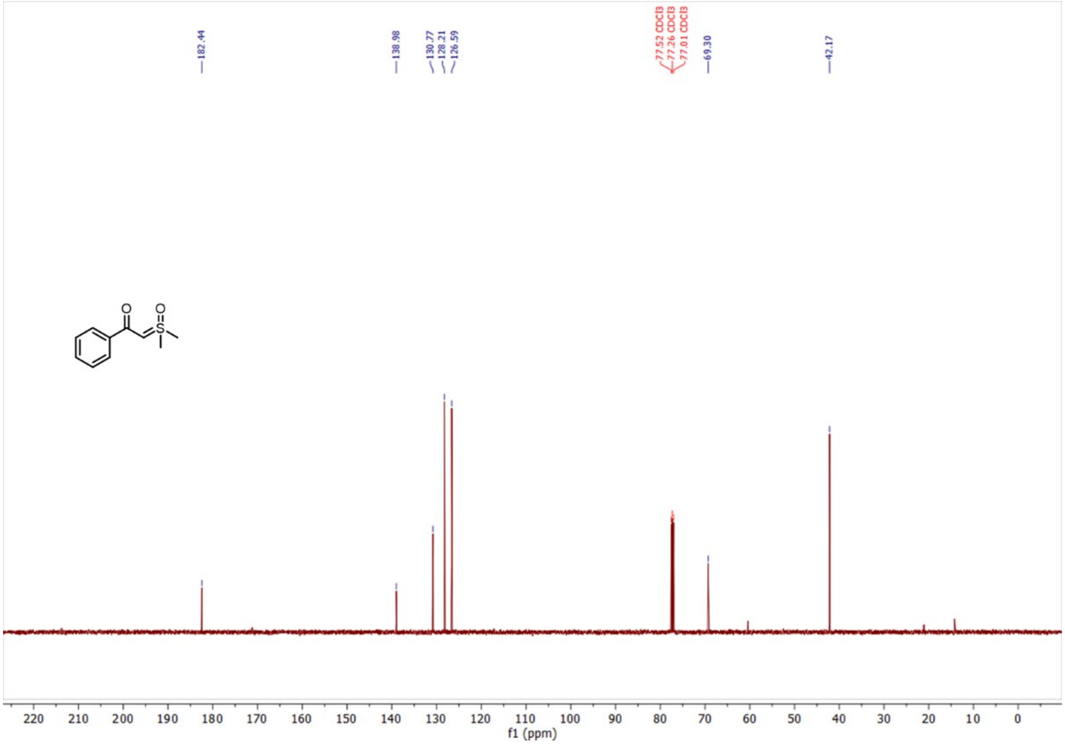

^{13}C NMR spectrum of **1a**

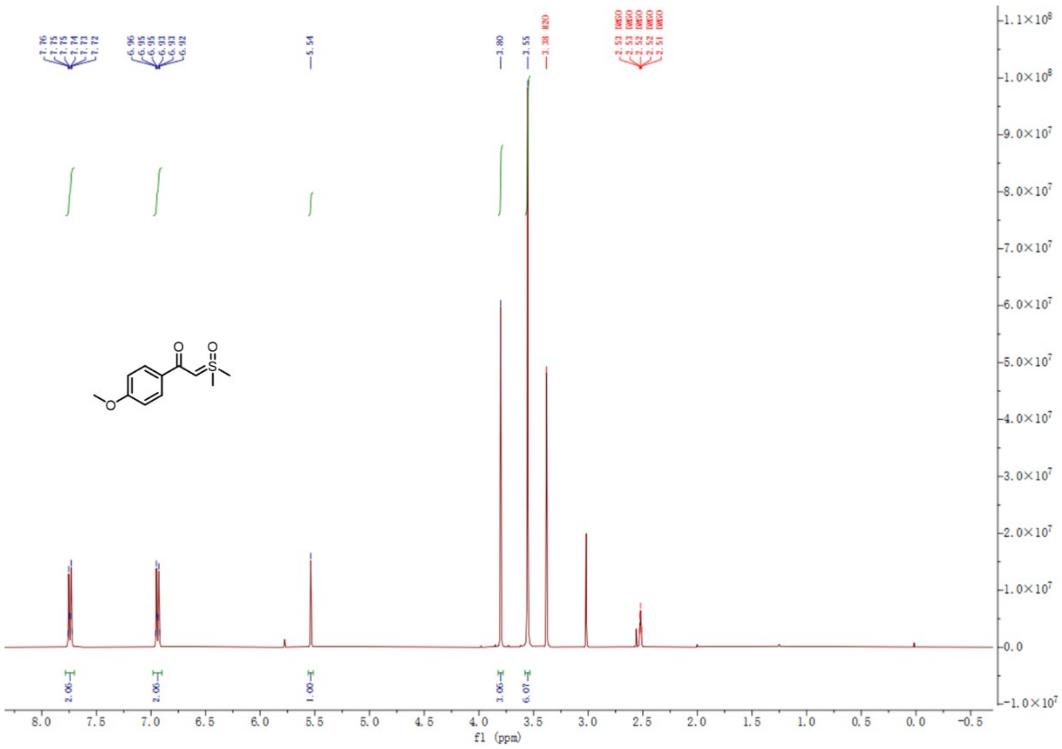

^{1}H NMR spectrum of **1b**

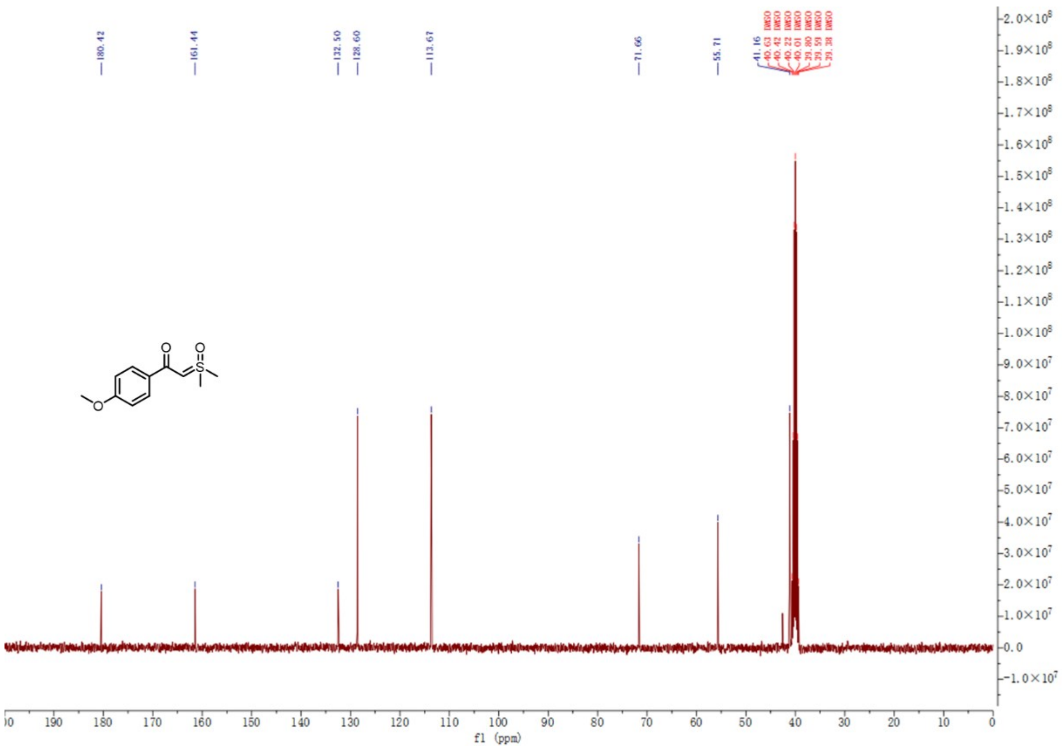

^{13}C NMR spectrum of **1b**

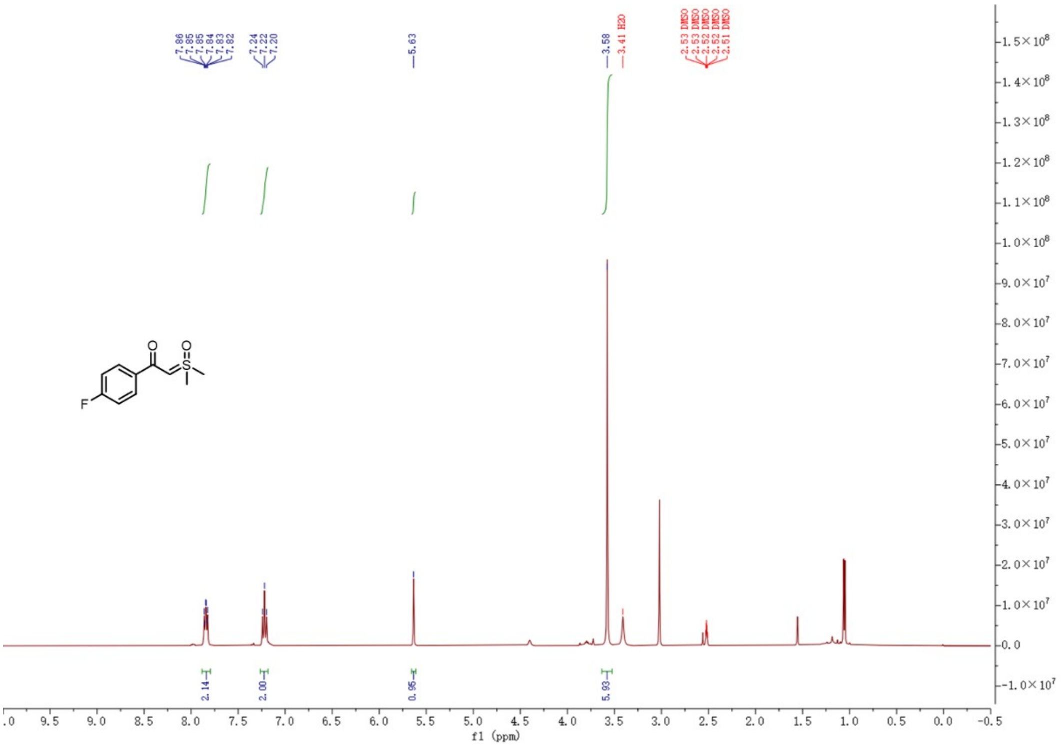

^{1}H NMR spectrum of **1c**

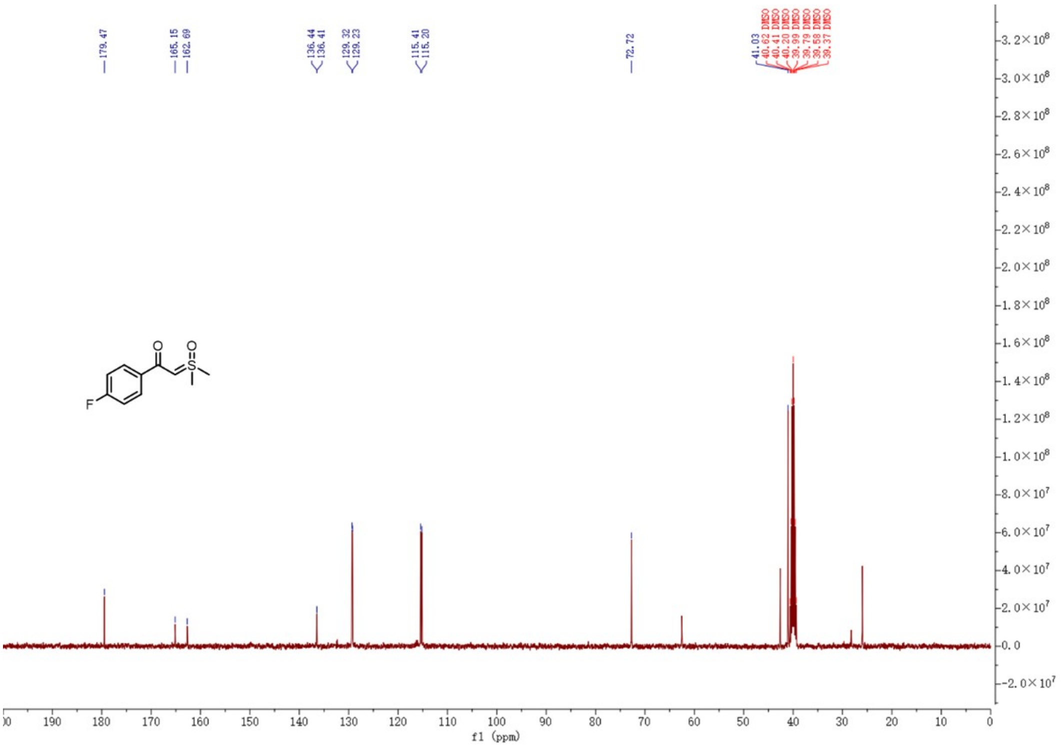

^{13}C NMR spectrum of **1c**

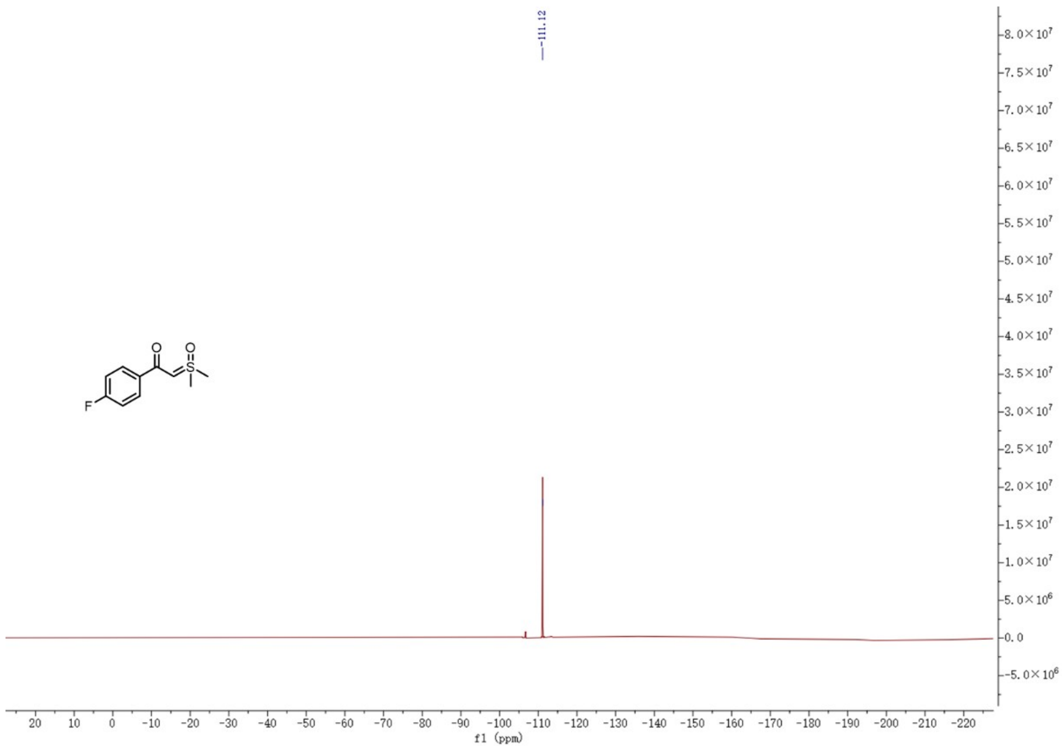

^{19}F NMR spectrum of **1c**

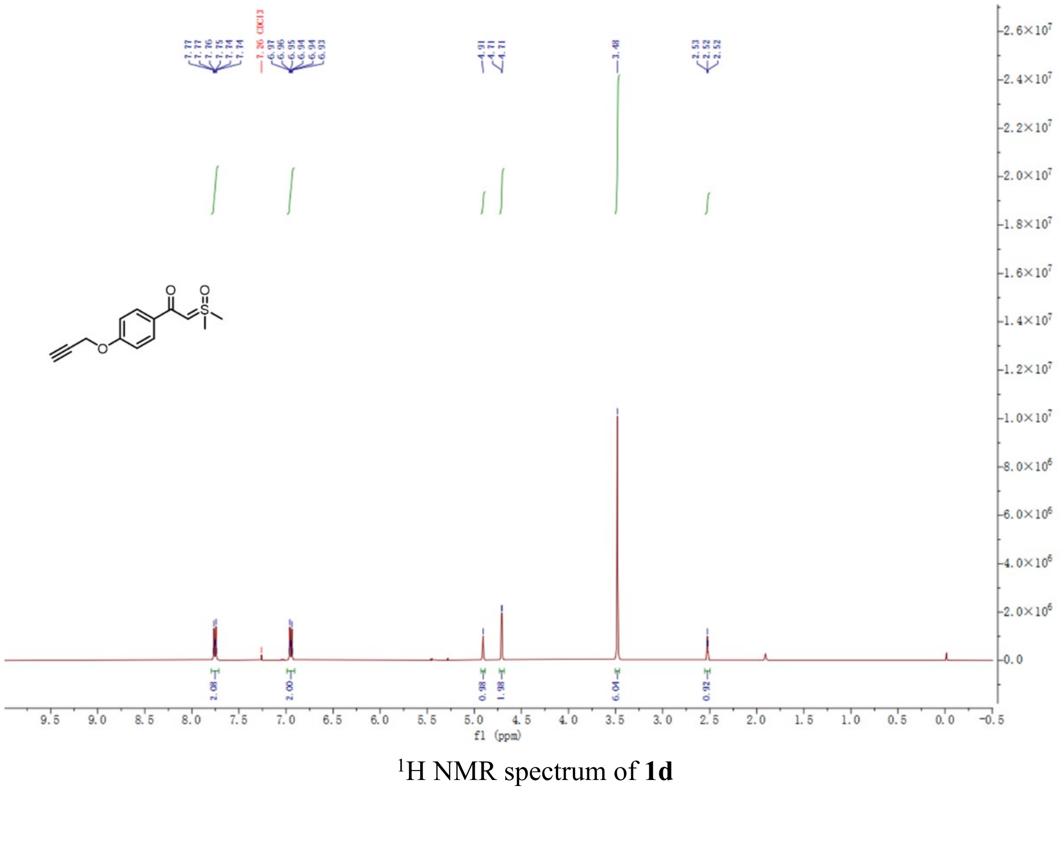

^{1}H NMR spectrum of **1d**

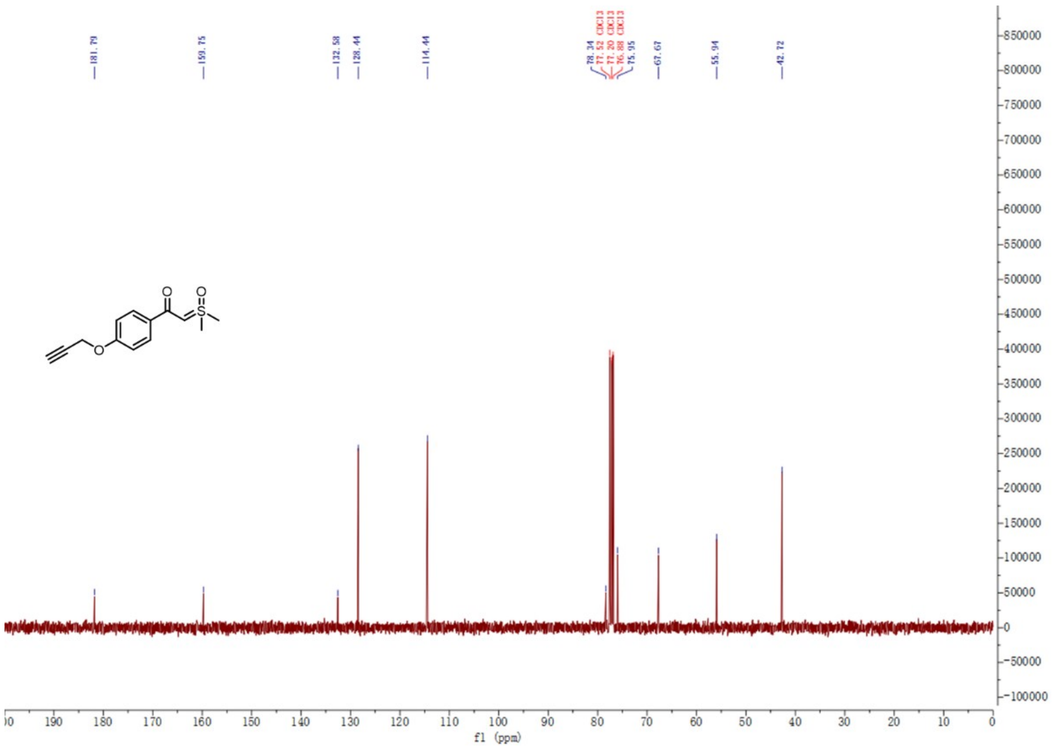

^{13}C NMR spectrum of **1d**

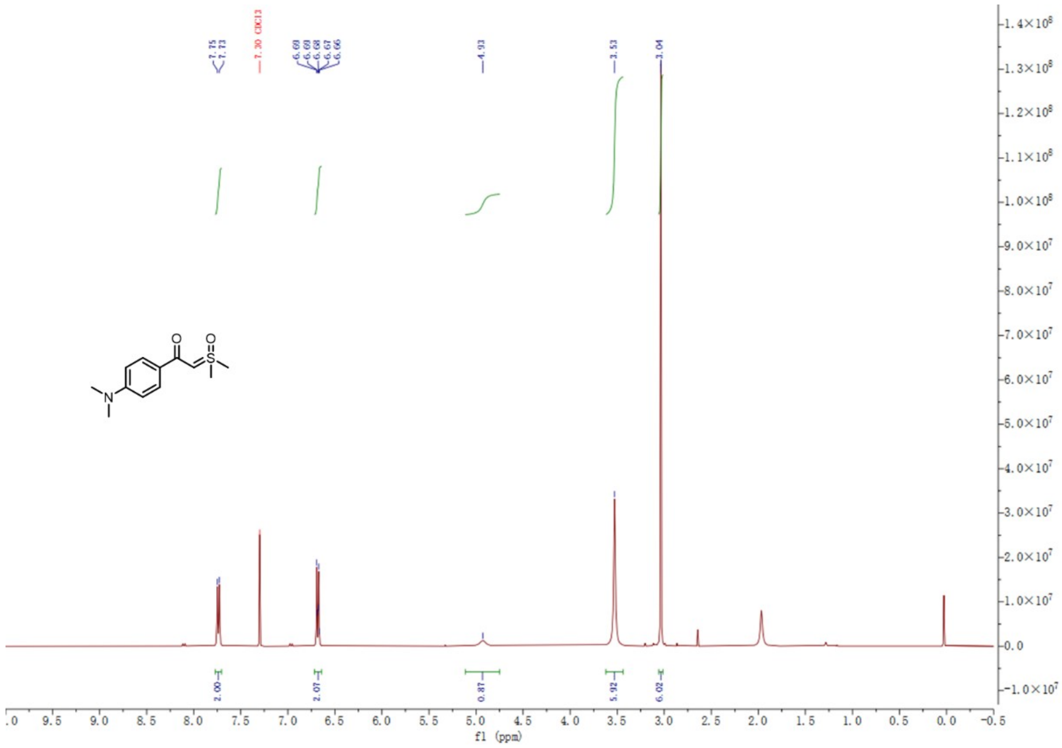

^{1}H NMR spectrum of **1e**

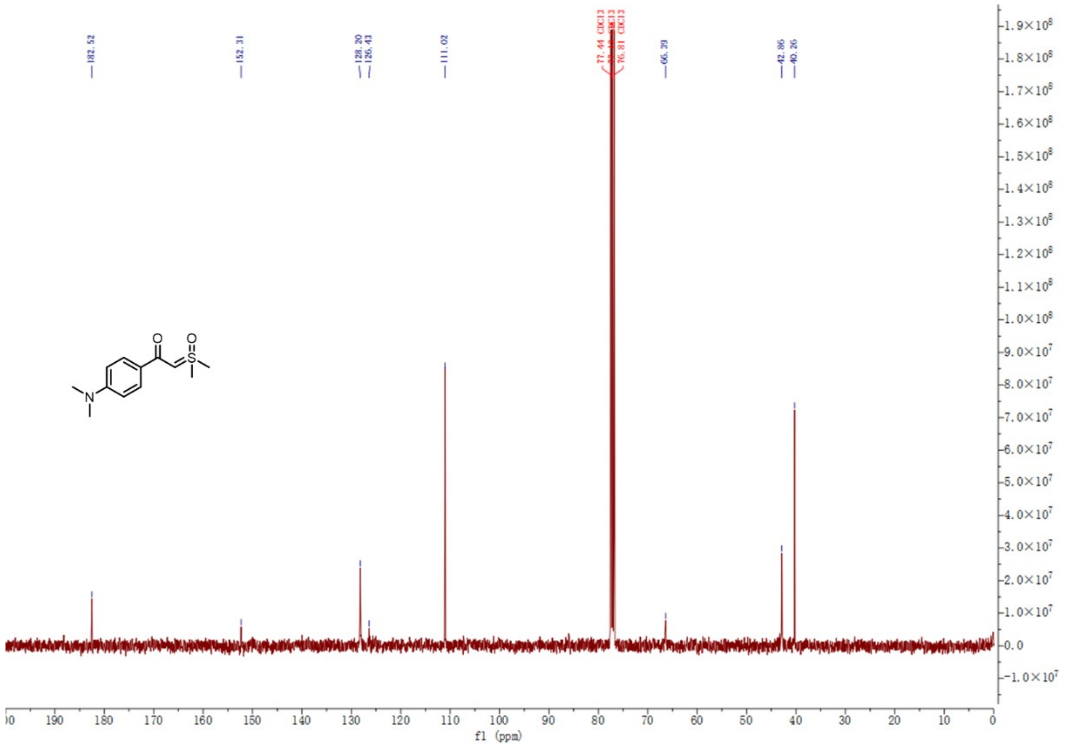

^{13}C NMR spectrum of **1e**

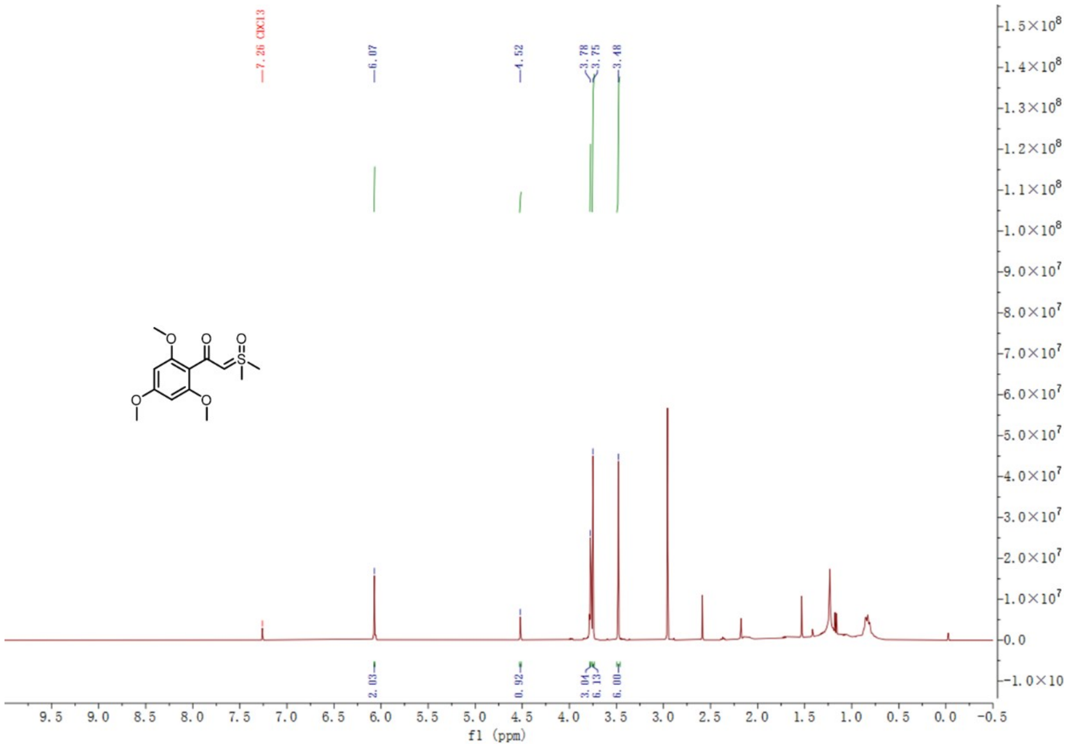

^{1}H NMR spectrum of **1f**

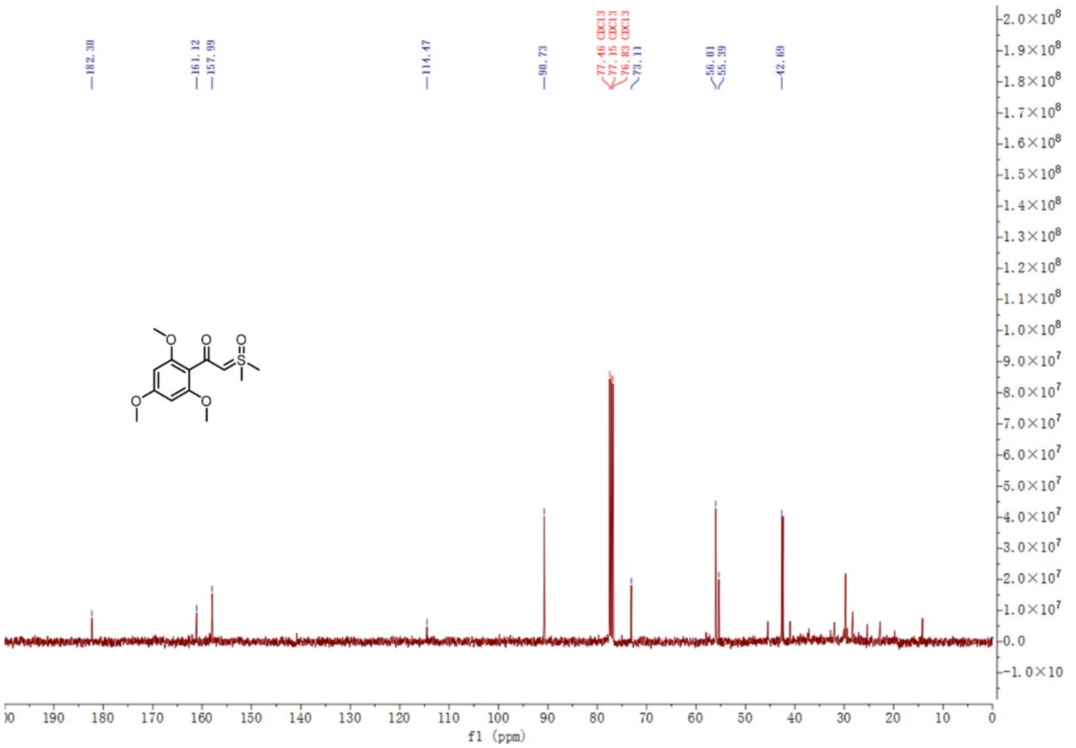

^{13}C NMR spectrum of **1f**

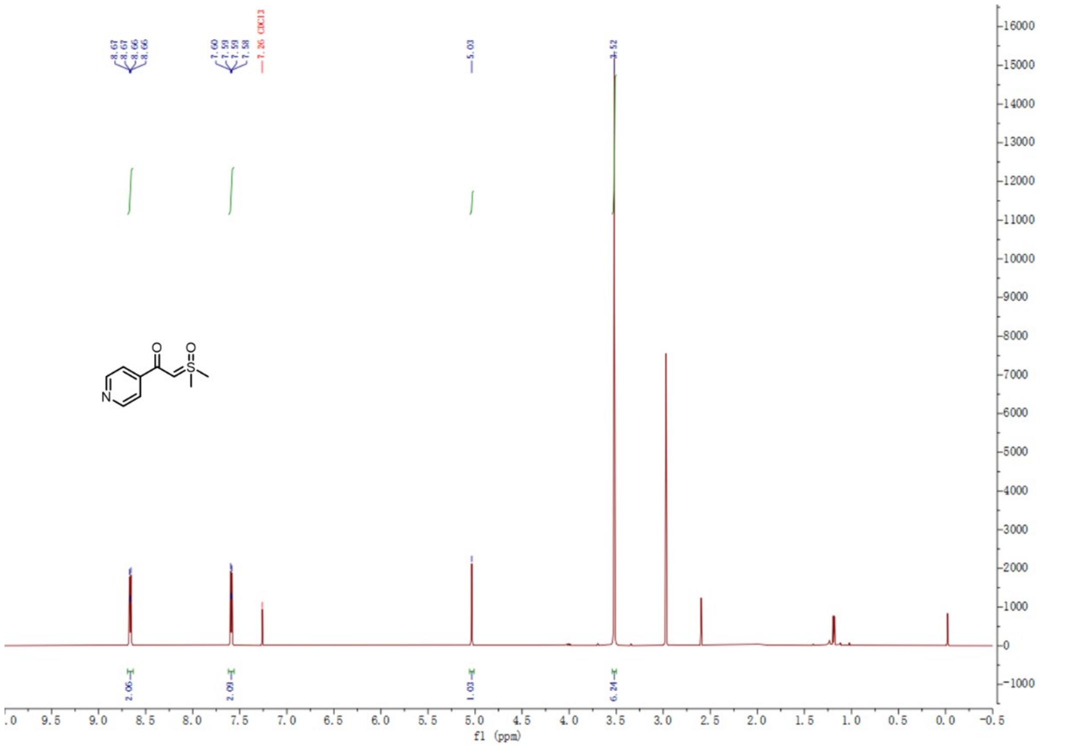

^{1}H NMR spectrum of **1g**

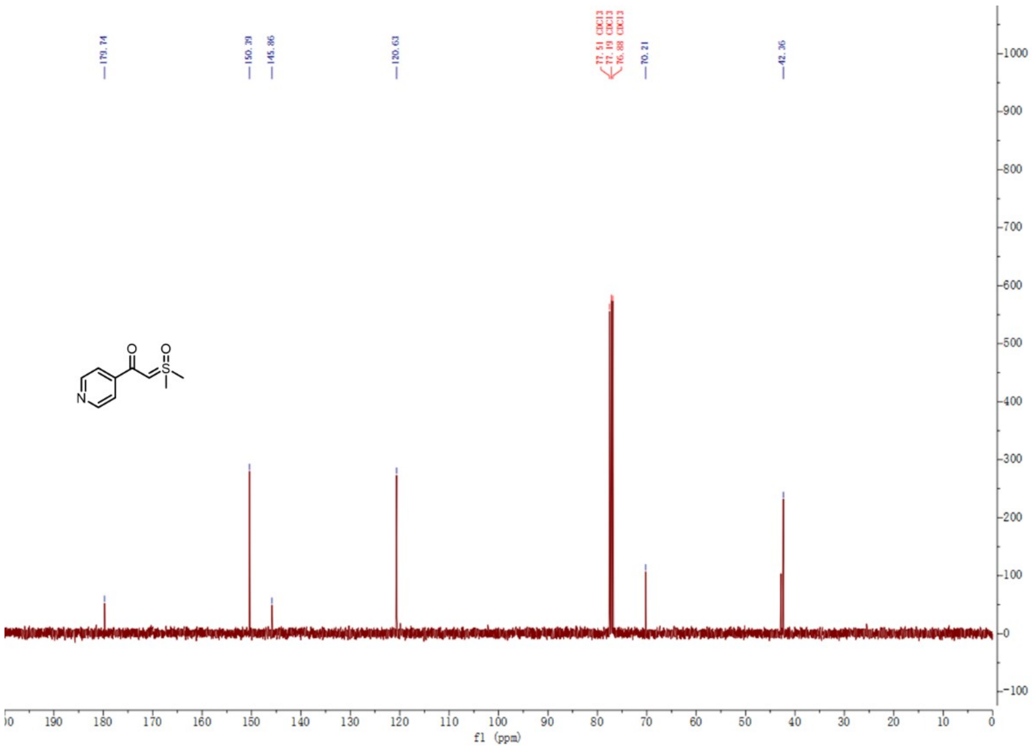

^{13}C NMR spectrum of **1g**

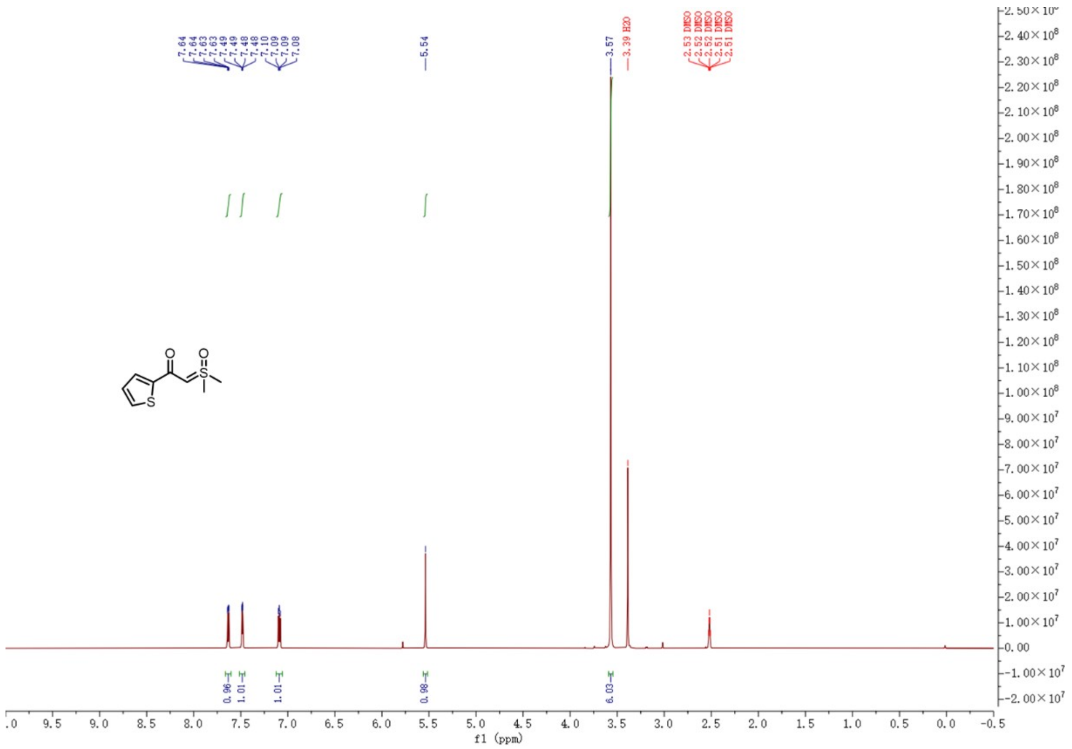

^{1}H NMR spectrum of **1h**

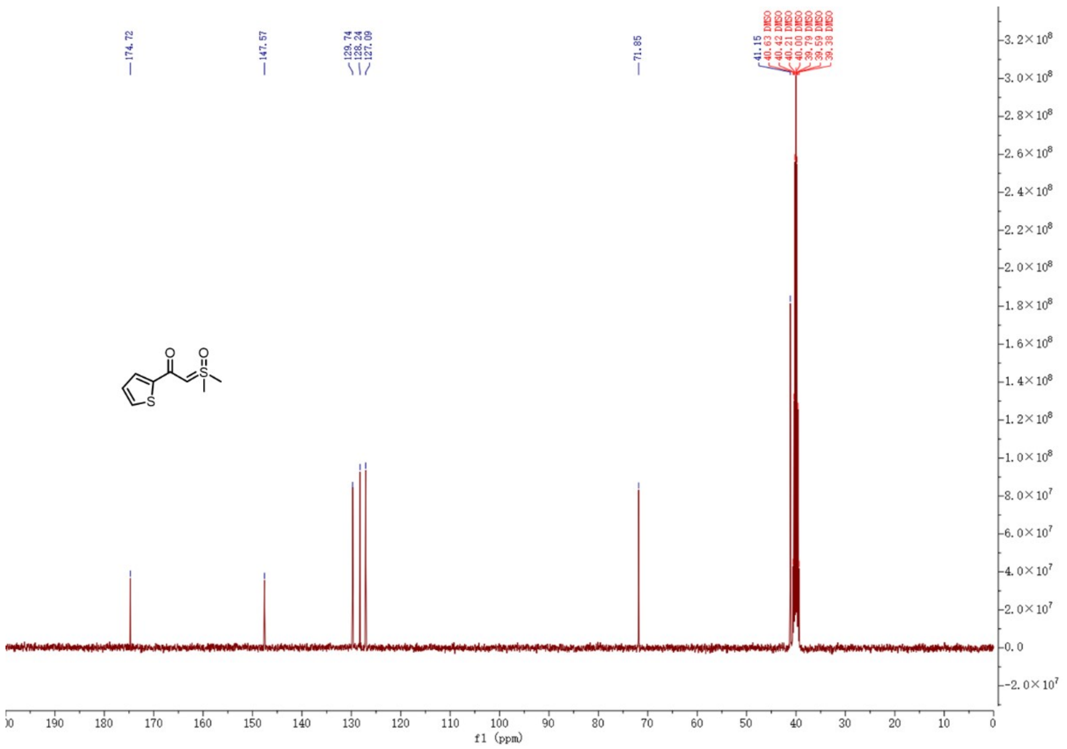

^{13}C NMR spectrum of **1h**

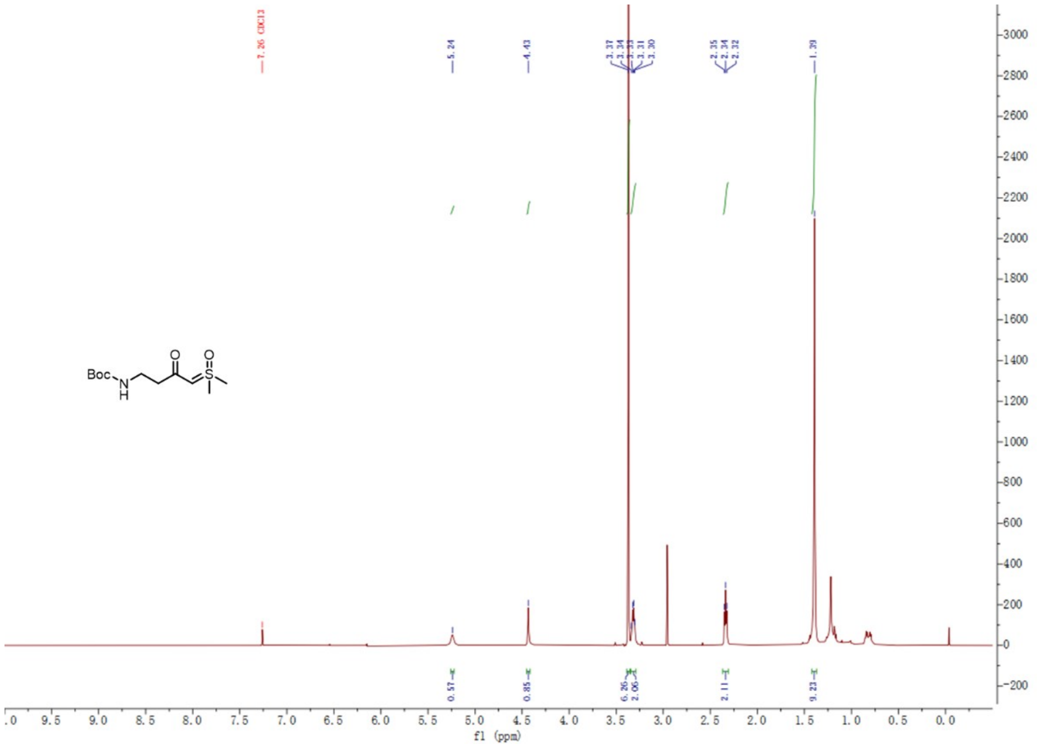

^{1}H NMR spectrum of **1i**

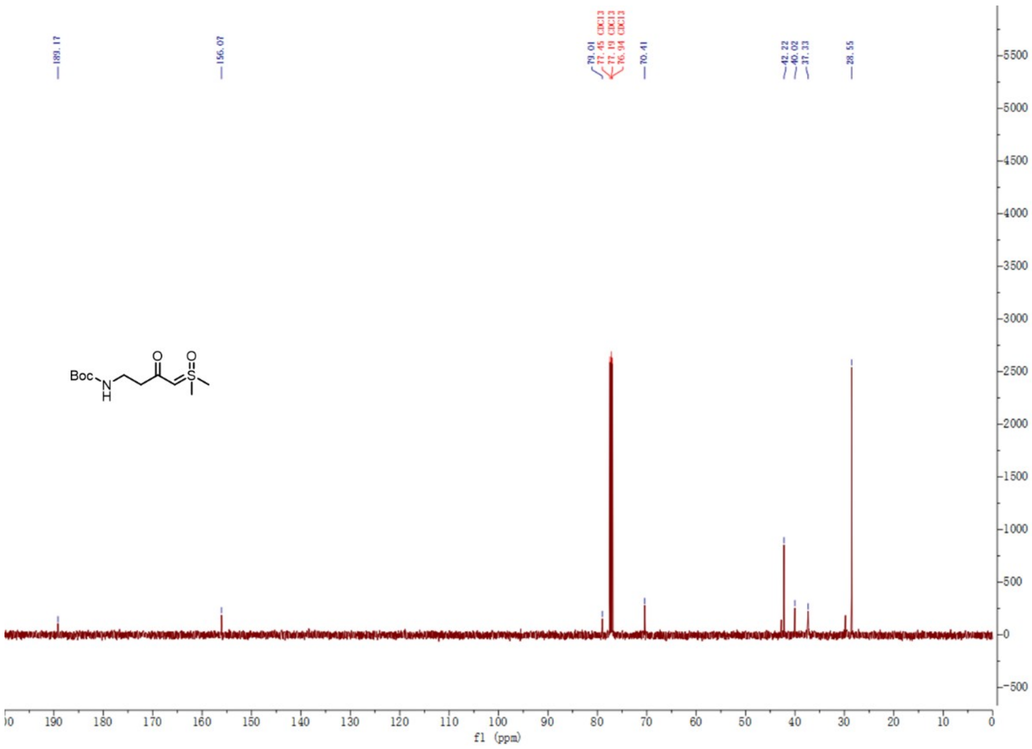

^{13}C NMR spectrum of **1i**

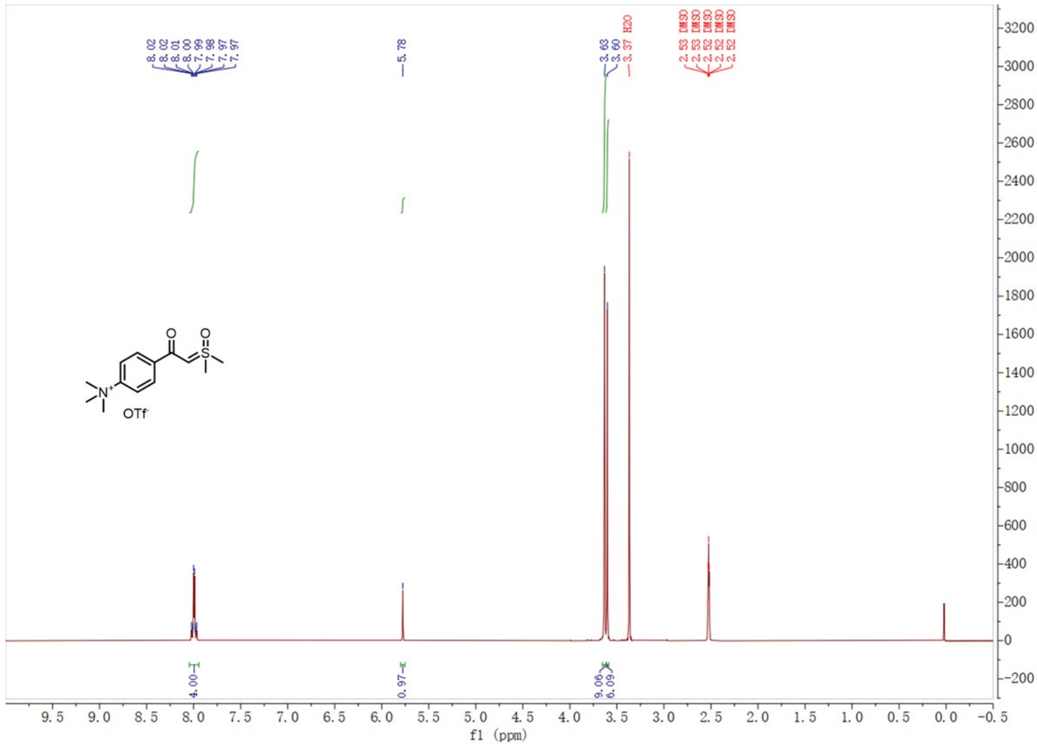

^{1}H NMR spectrum of **1j**

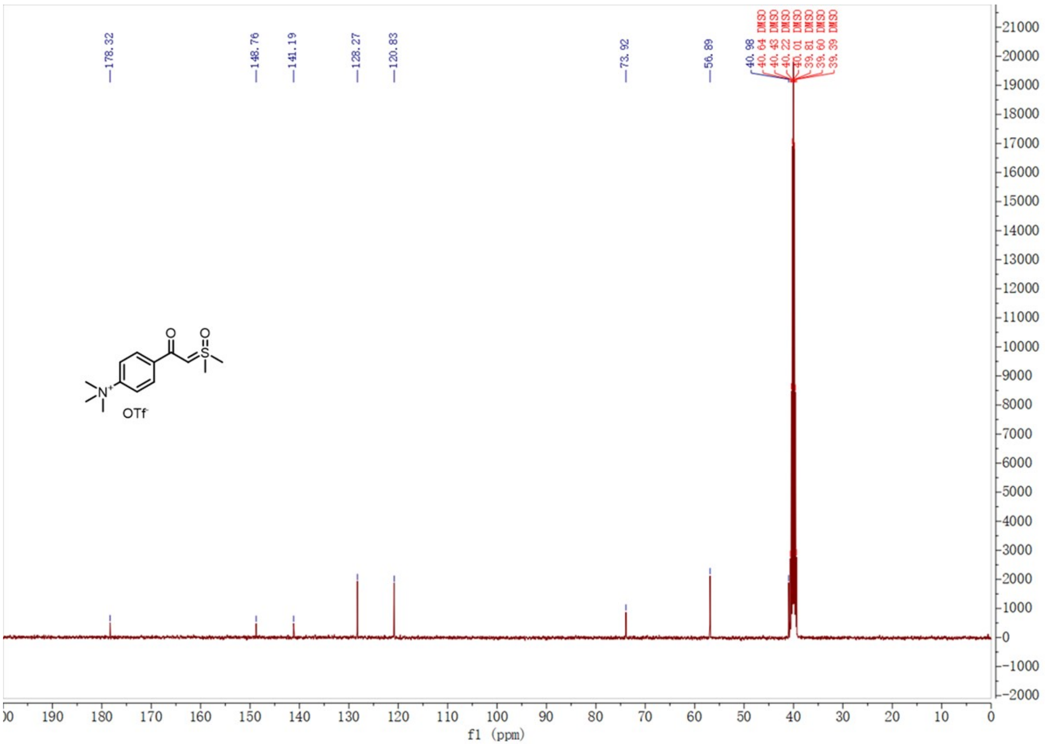

^{13}C NMR spectrum of **1j**

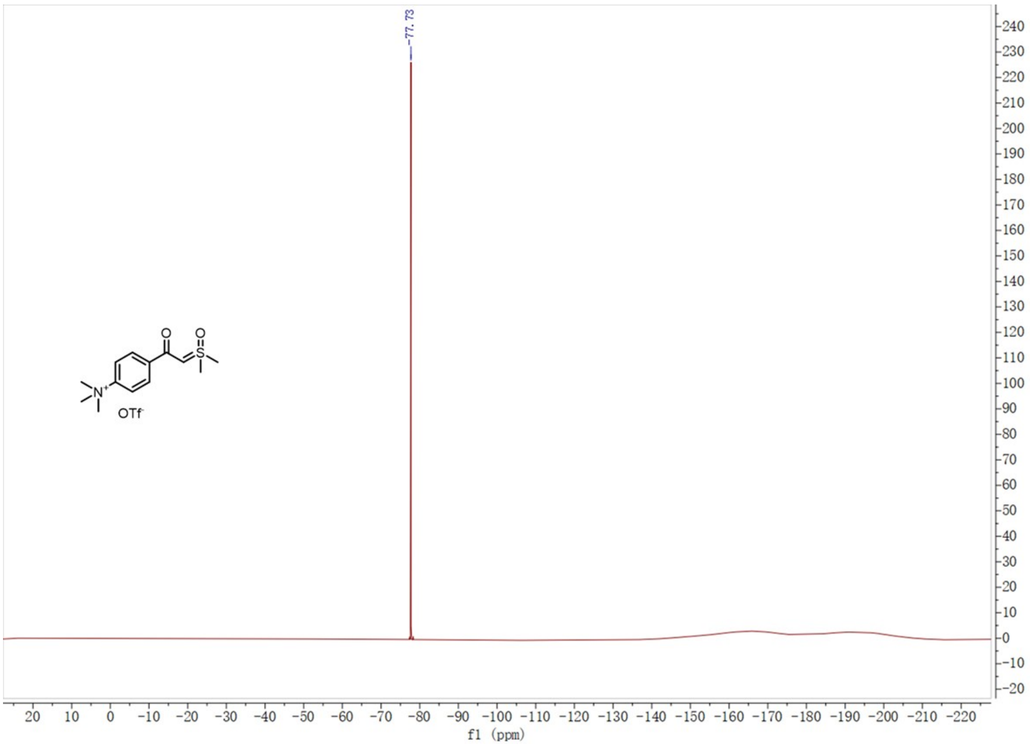

^{19}F NMR spectrum of **1j**

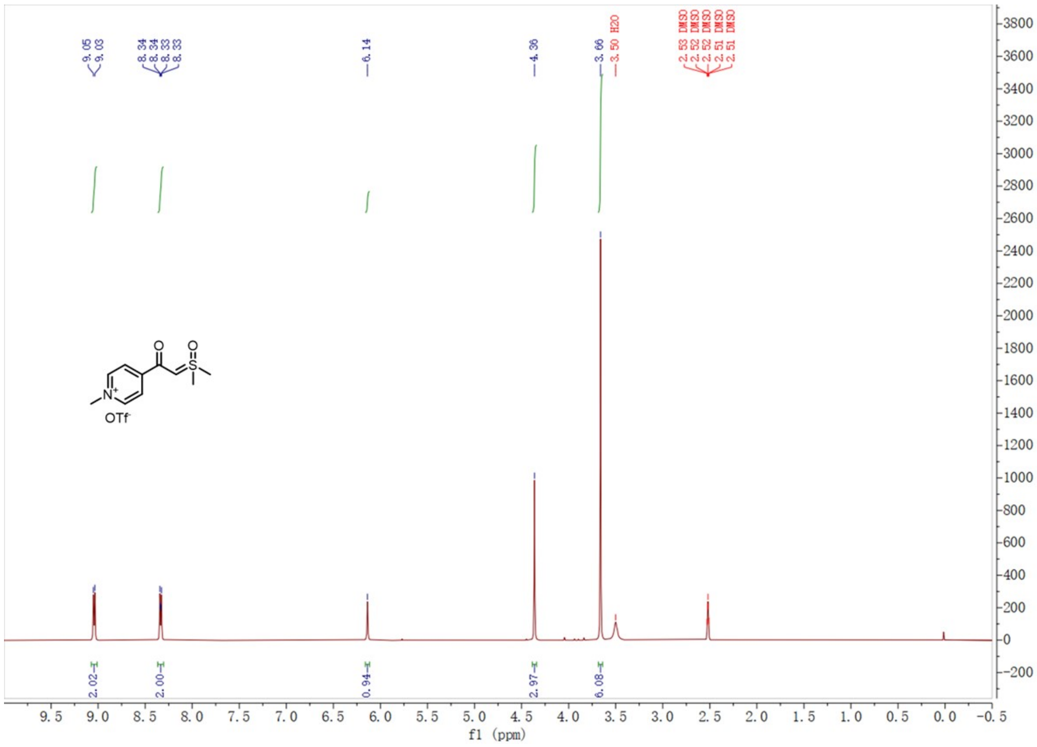

^{1}H NMR spectrum of **1k**

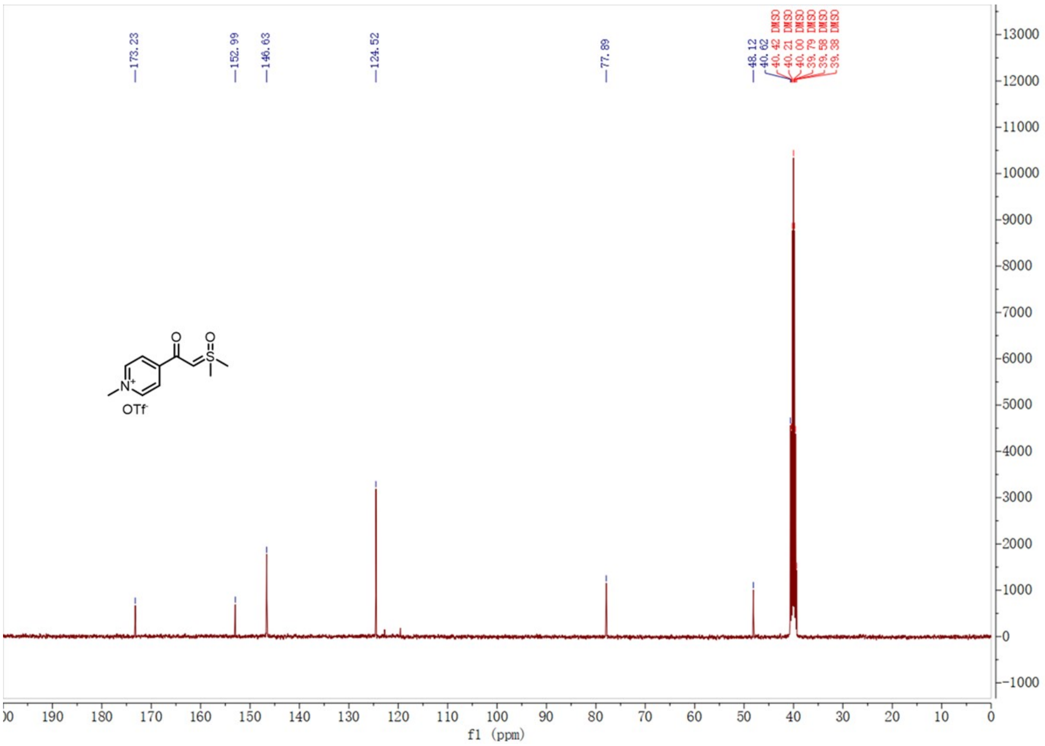

^{13}C NMR spectrum of **1k**

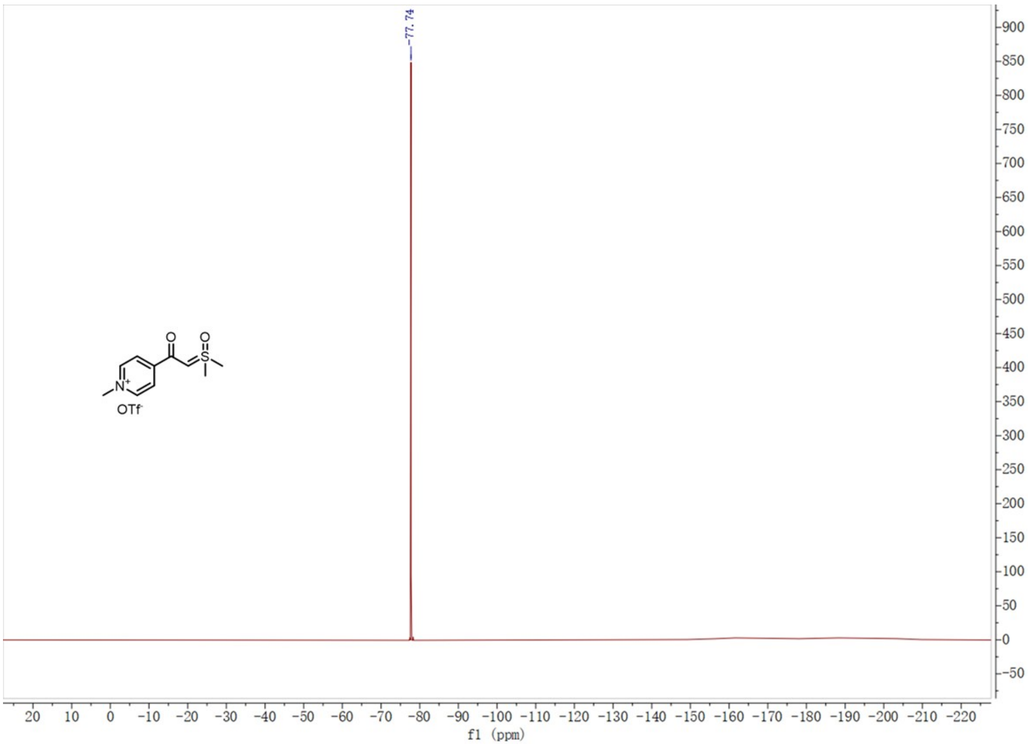

^{19}F NMR spectrum of **1k**

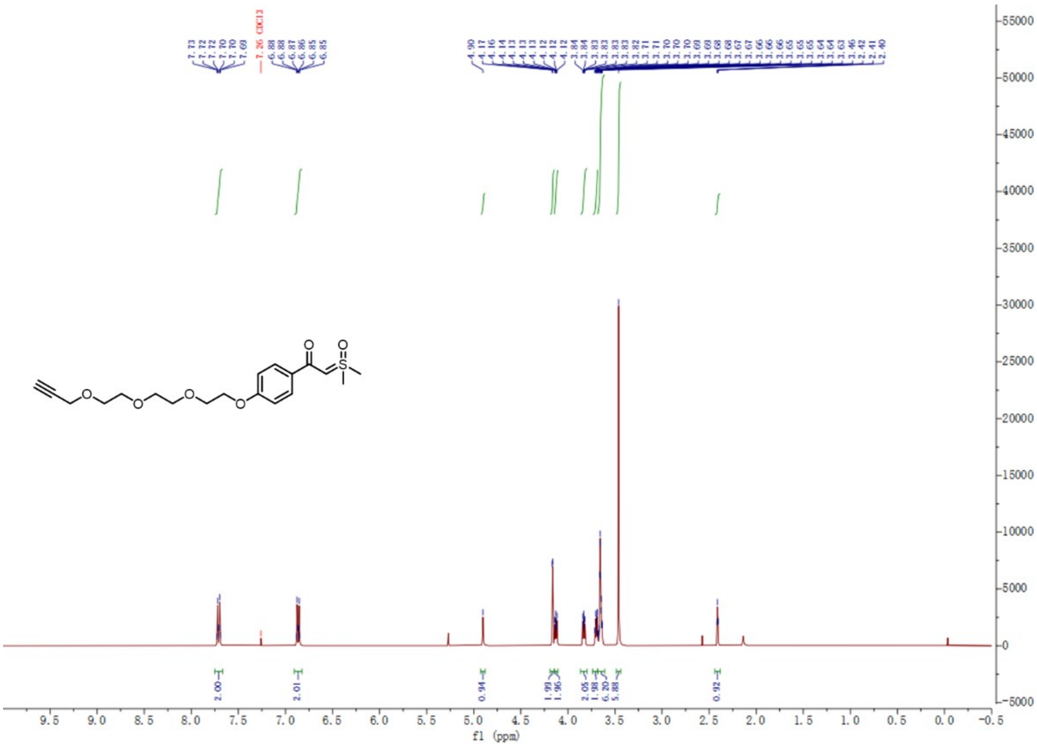

^{1}H NMR spectrum of **1l**

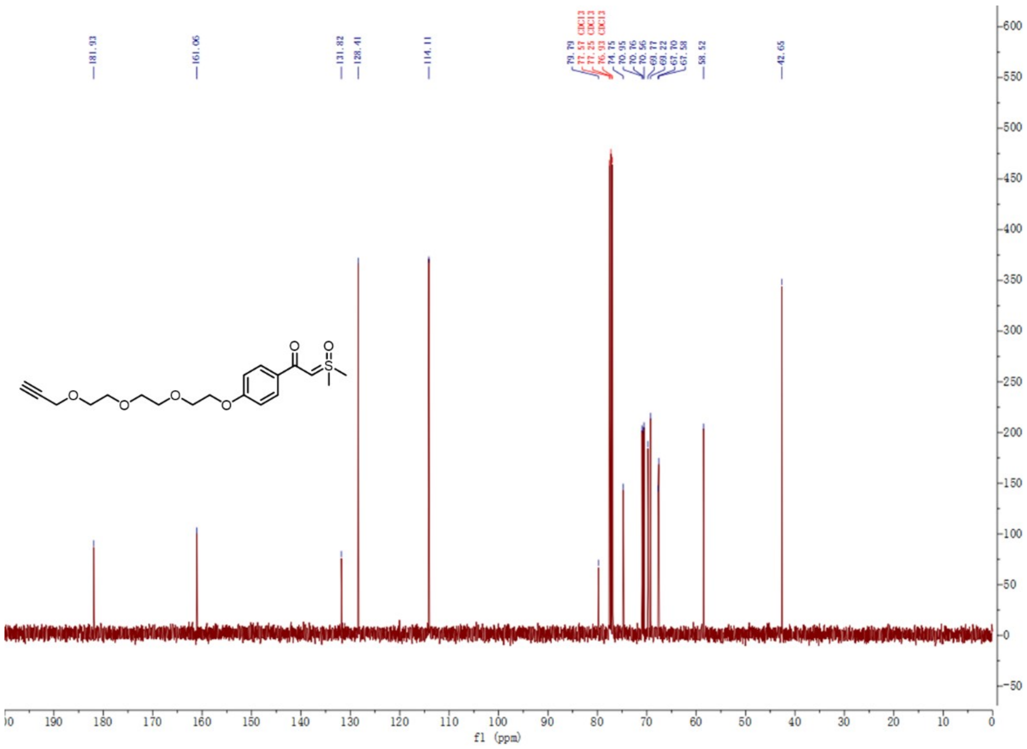

^{13}C NMR spectrum of **1l**

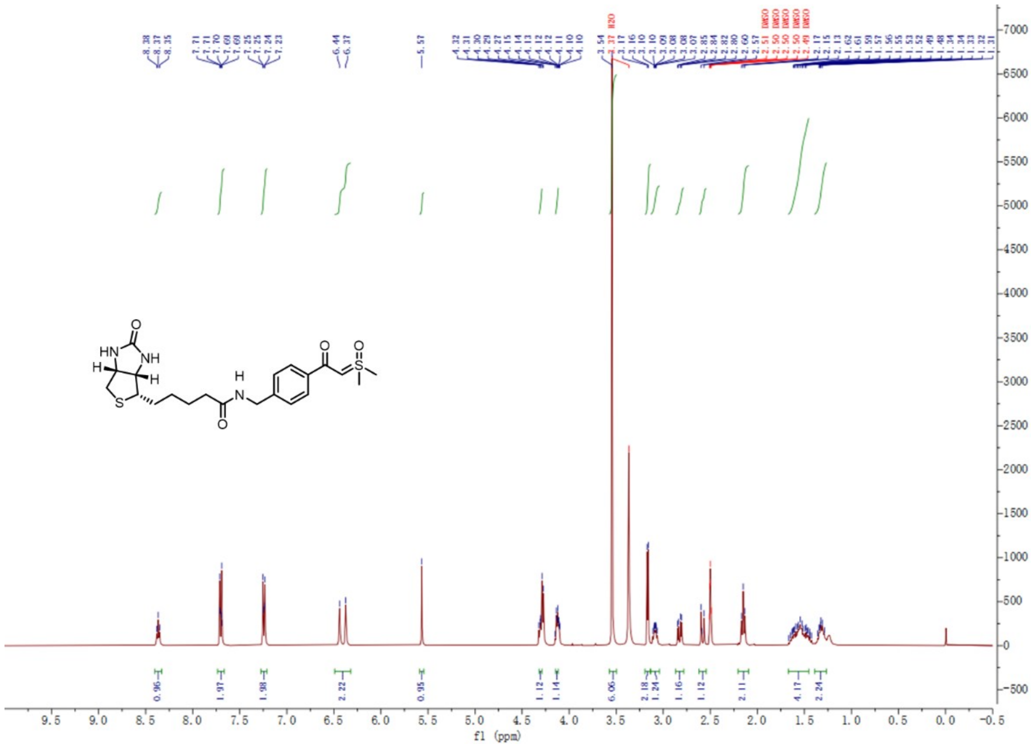

^{1}H NMR spectrum of **1m**

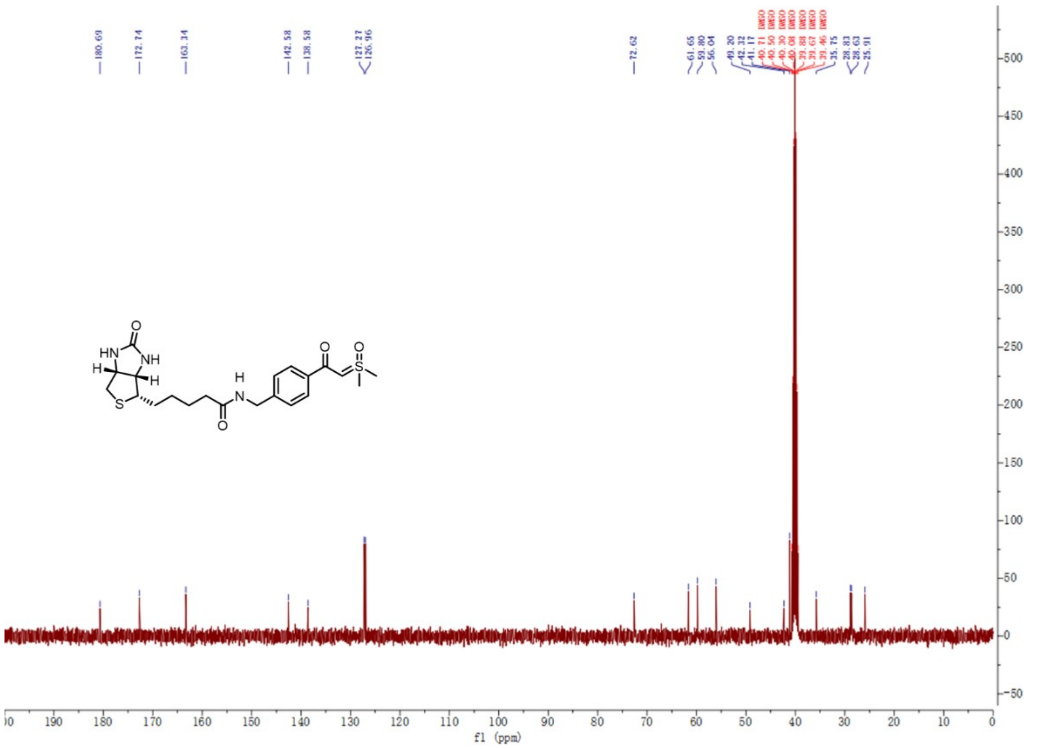

^{13}C NMR spectrum of **1m**

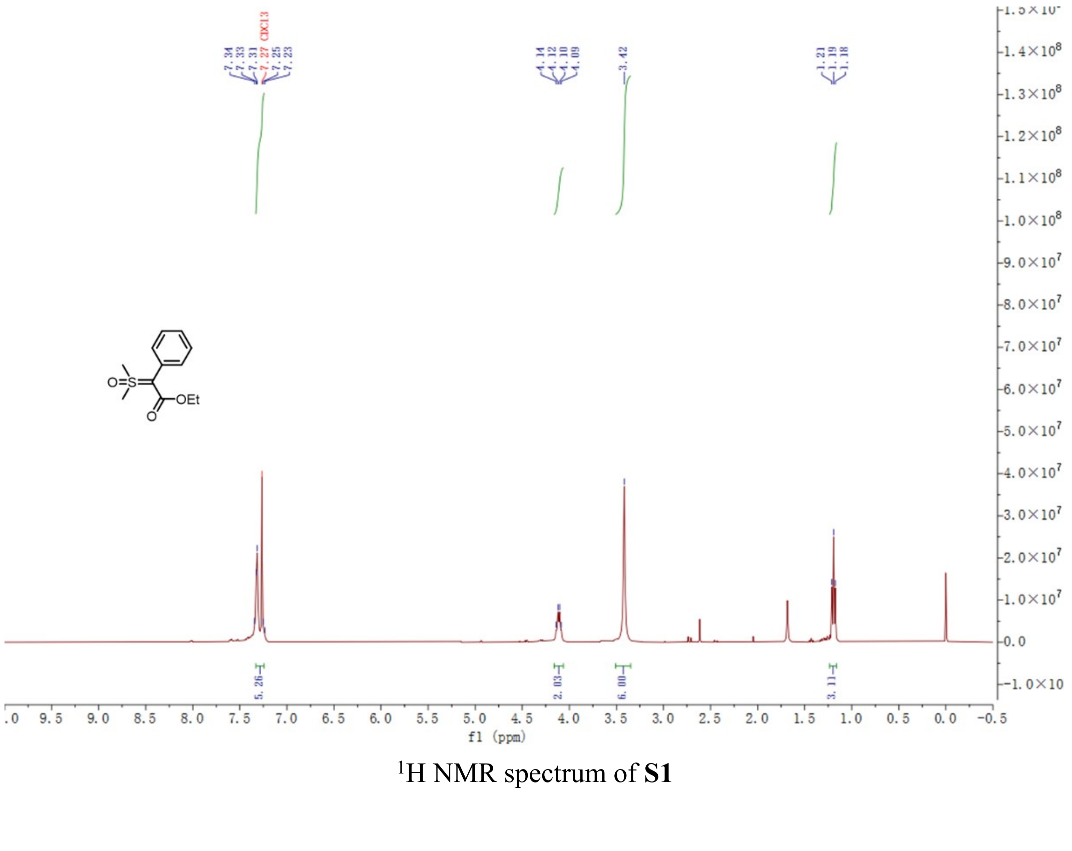

^{1}H NMR spectrum of **S1**

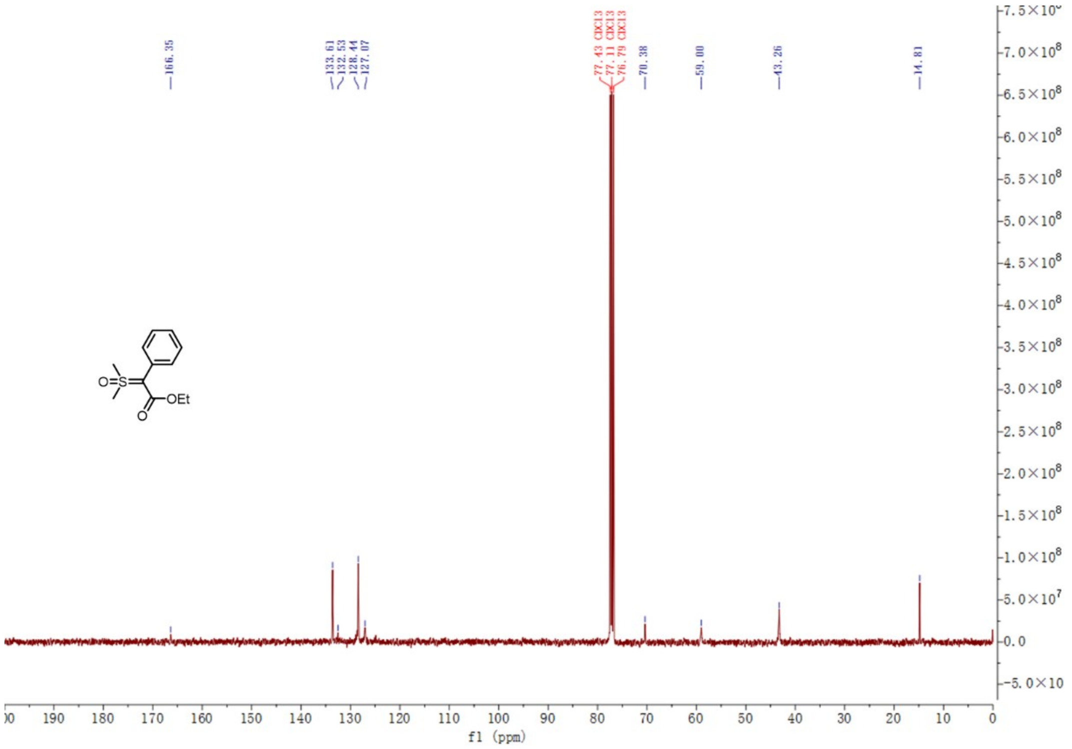

^{13}C NMR spectrum of **S1**

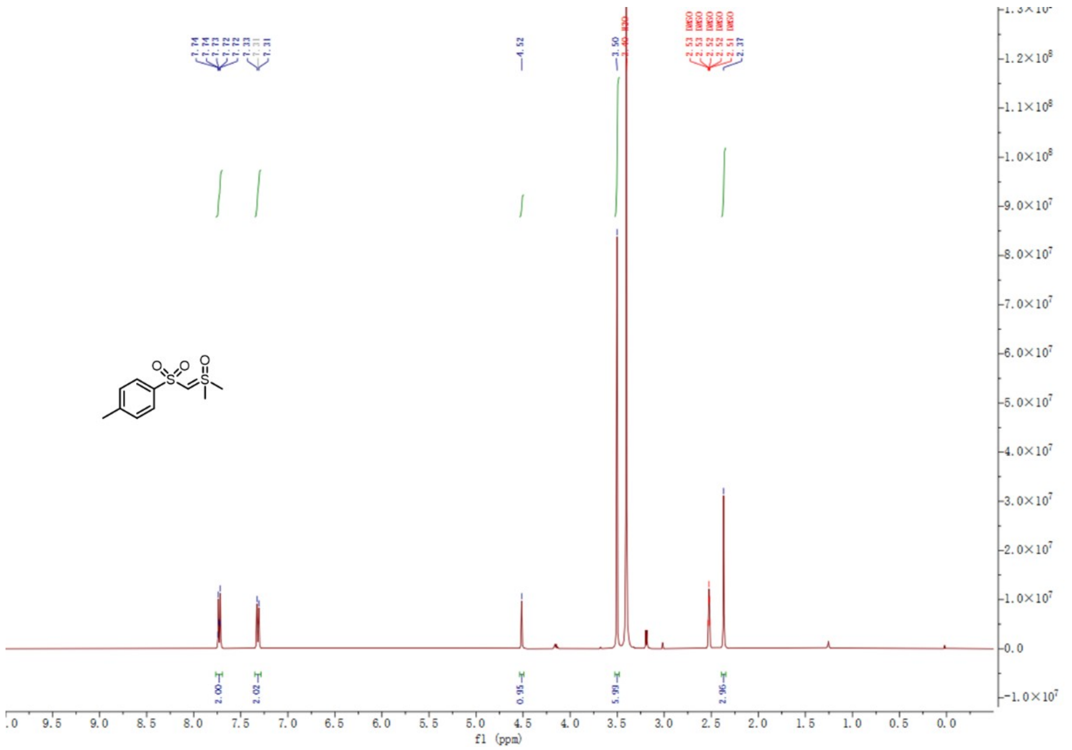

^{1}H NMR spectrum of **S2**

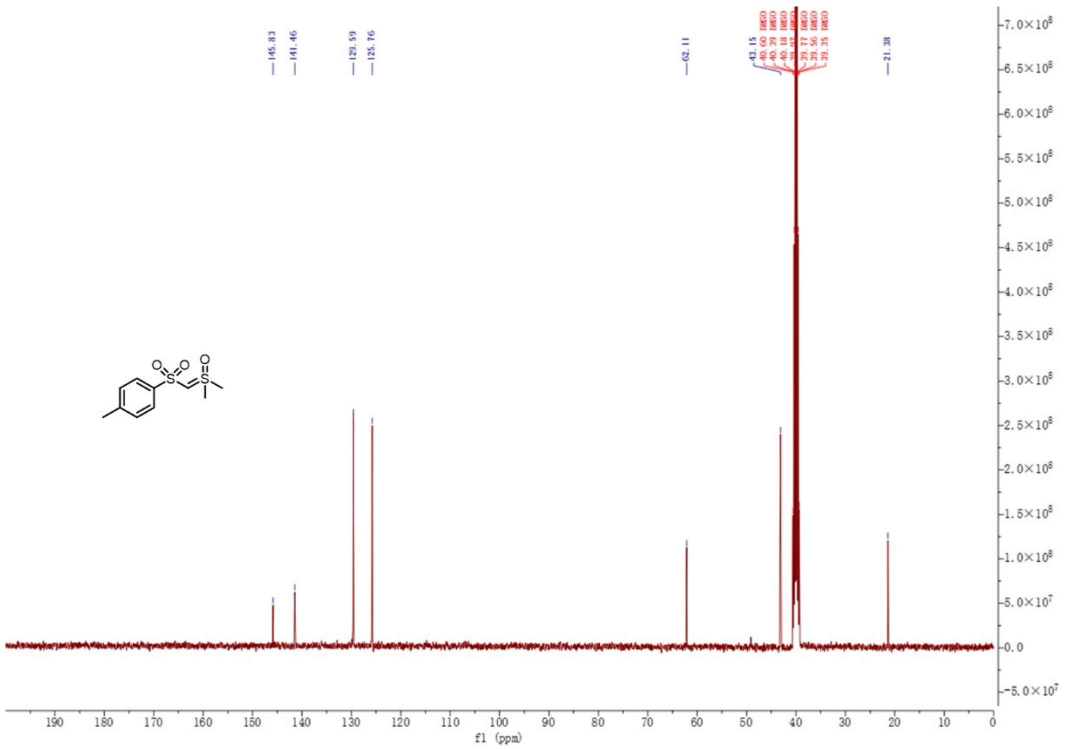

^{13}C NMR spectrum of **S2**

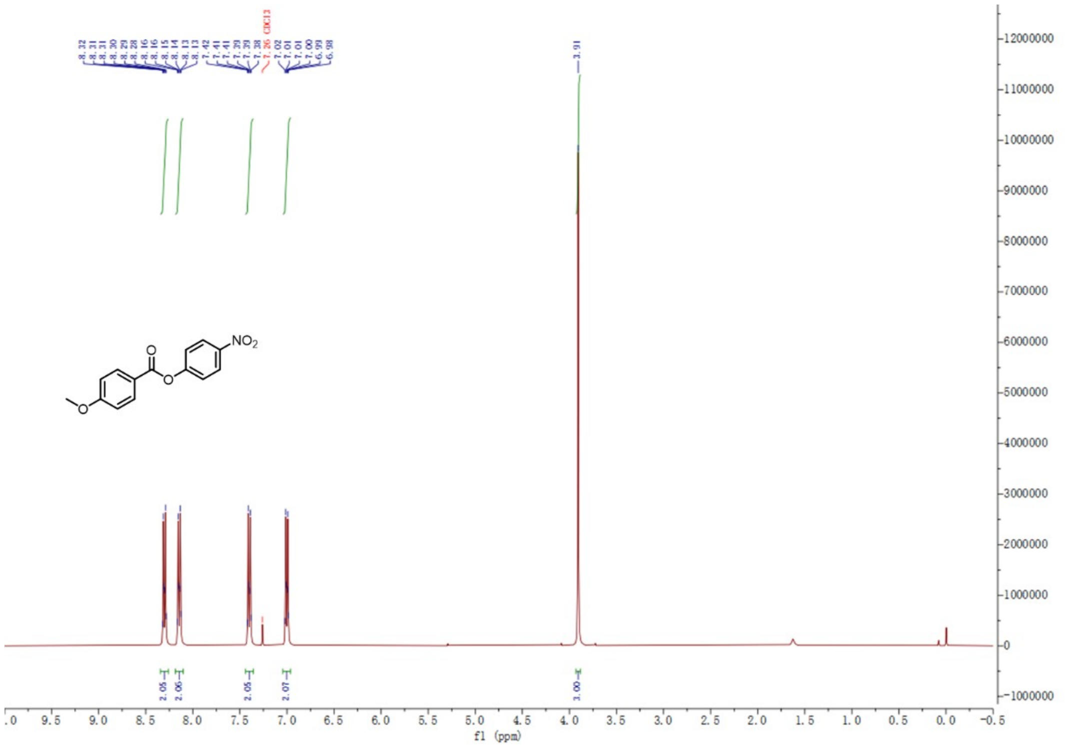

^{1}H NMR spectrum of **S3**

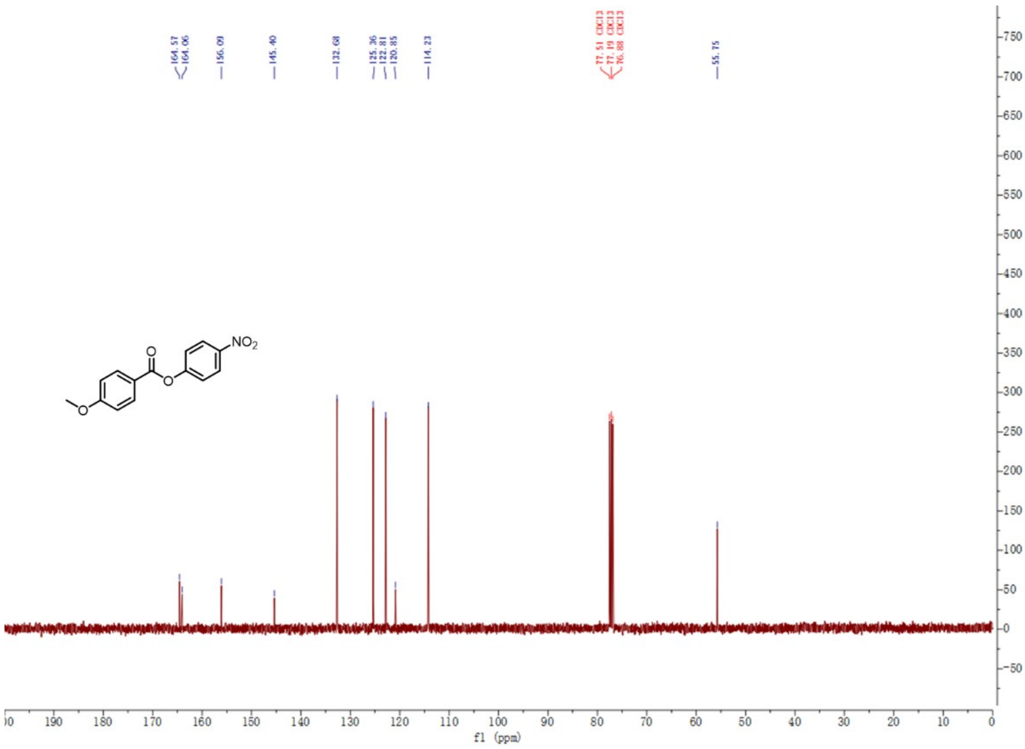

^{13}C NMR spectrum of **S3**

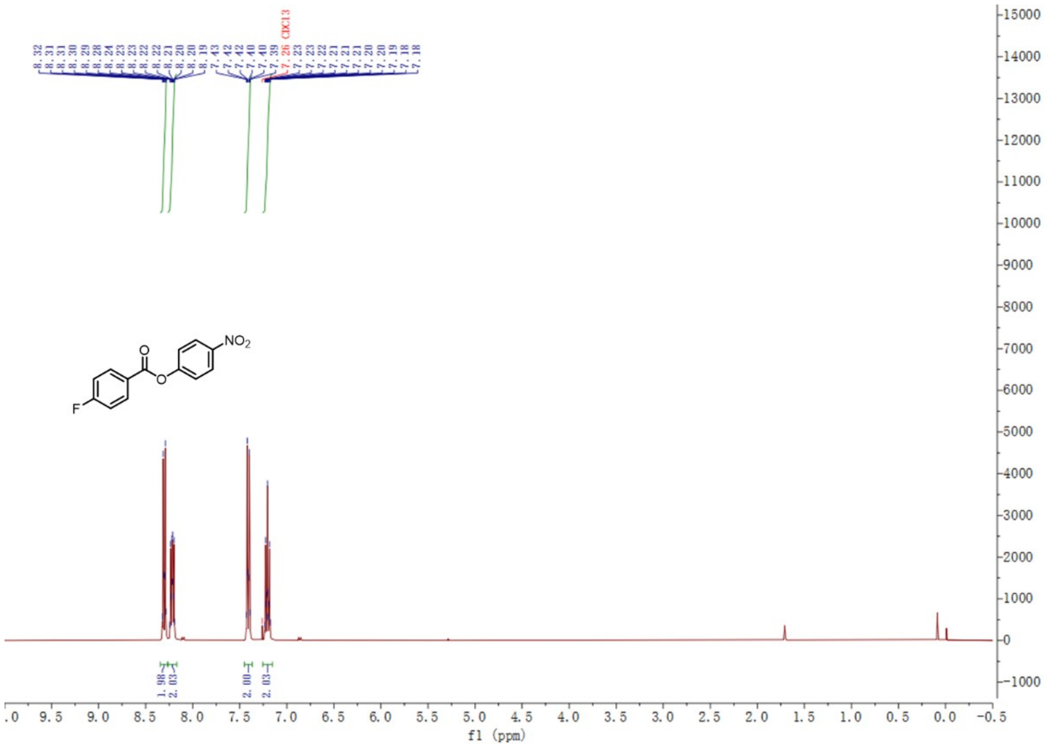

^{1}H NMR spectrum of **S4**

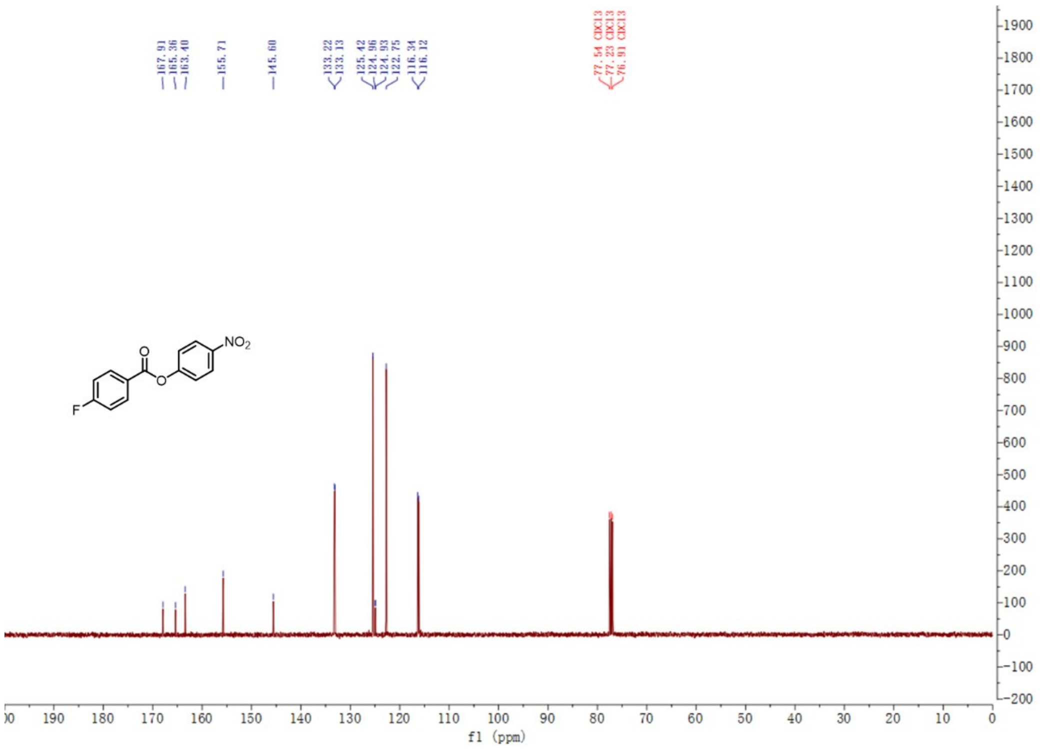

^{13}C NMR spectrum of **S4**

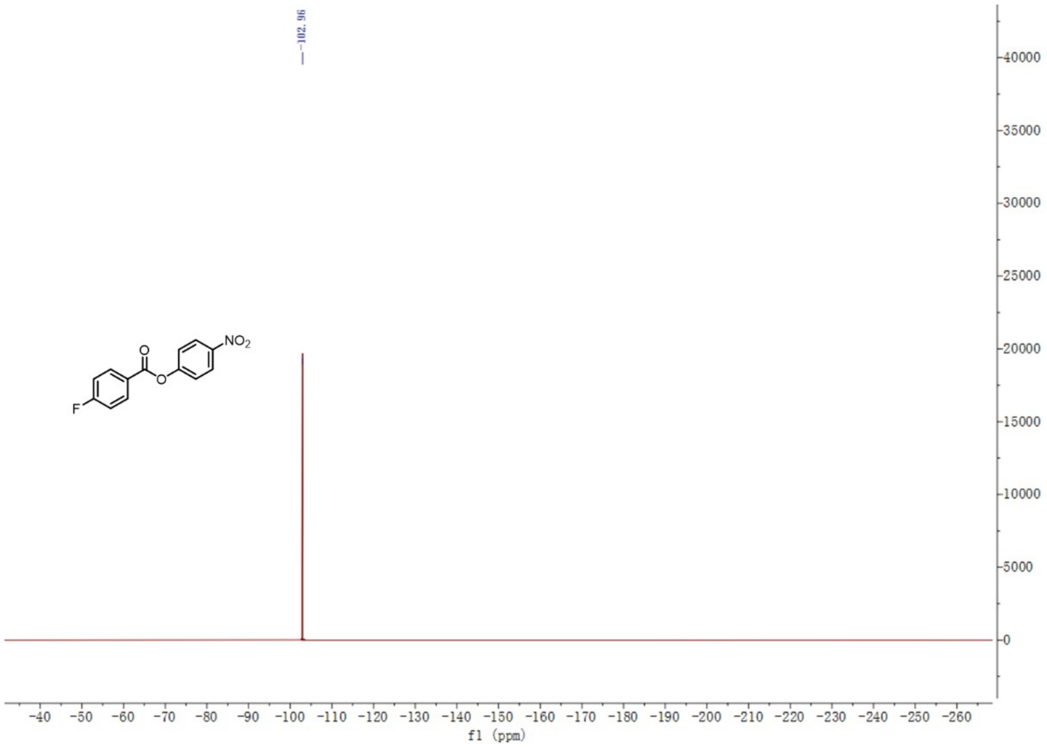

^{19}F NMR spectrum of **S4**

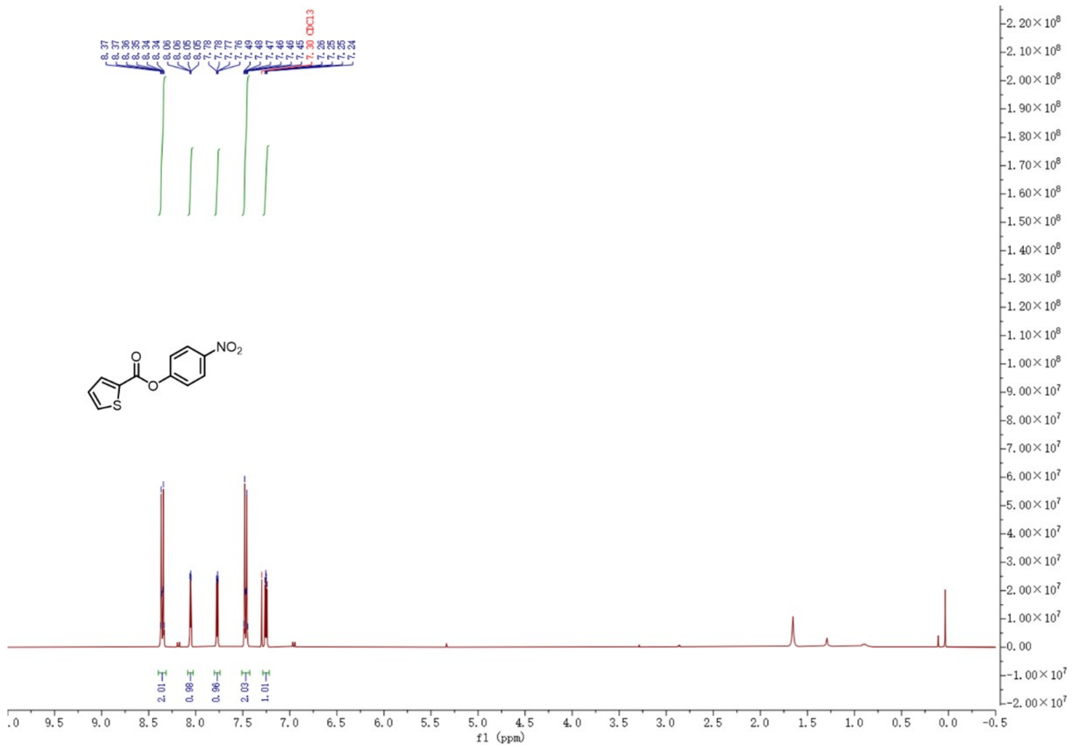

^{1}H NMR spectrum of **S5**

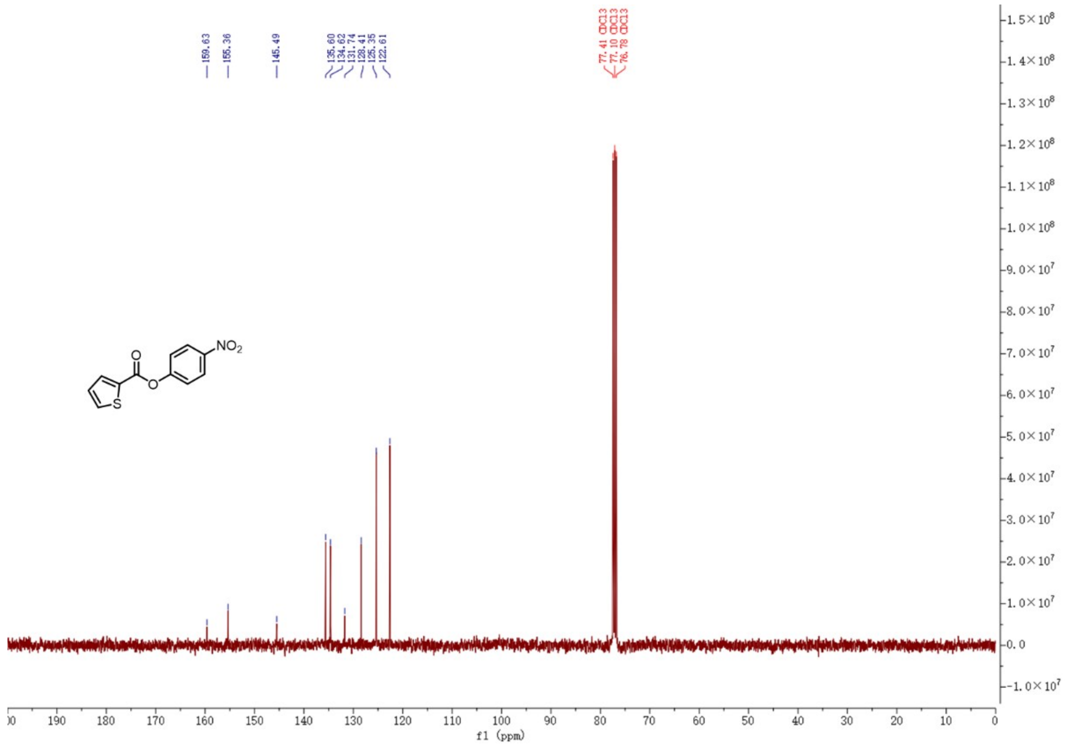

^{13}C NMR spectrum of **S5**

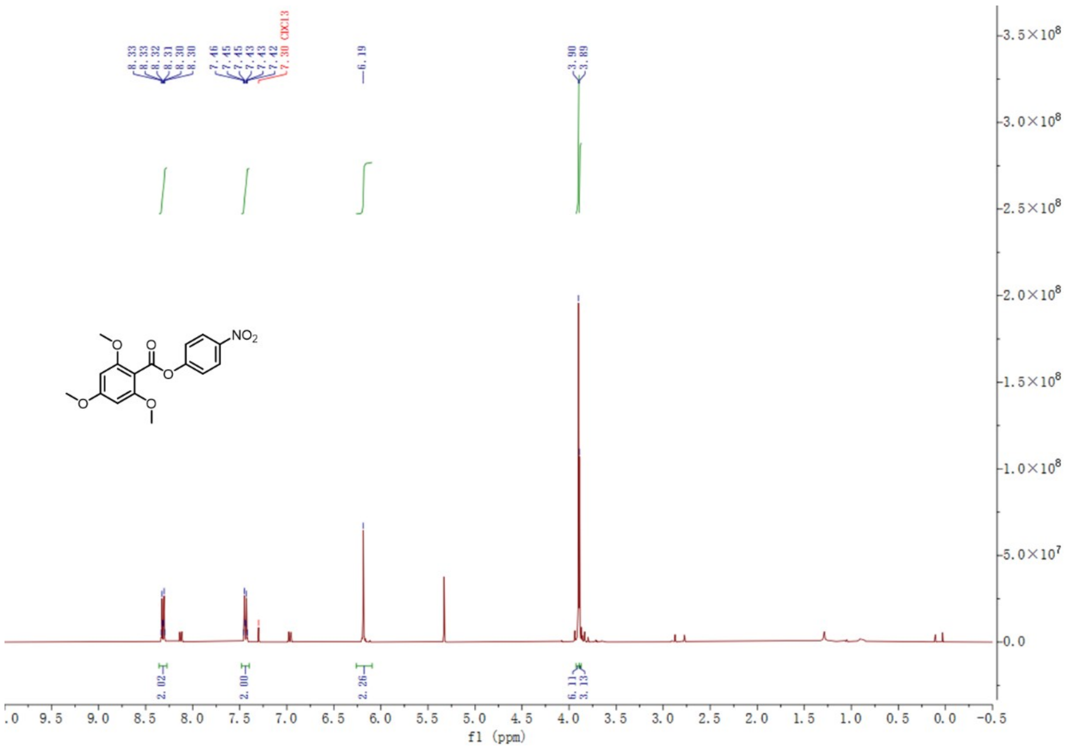

^{1}H NMR spectrum of **S6**

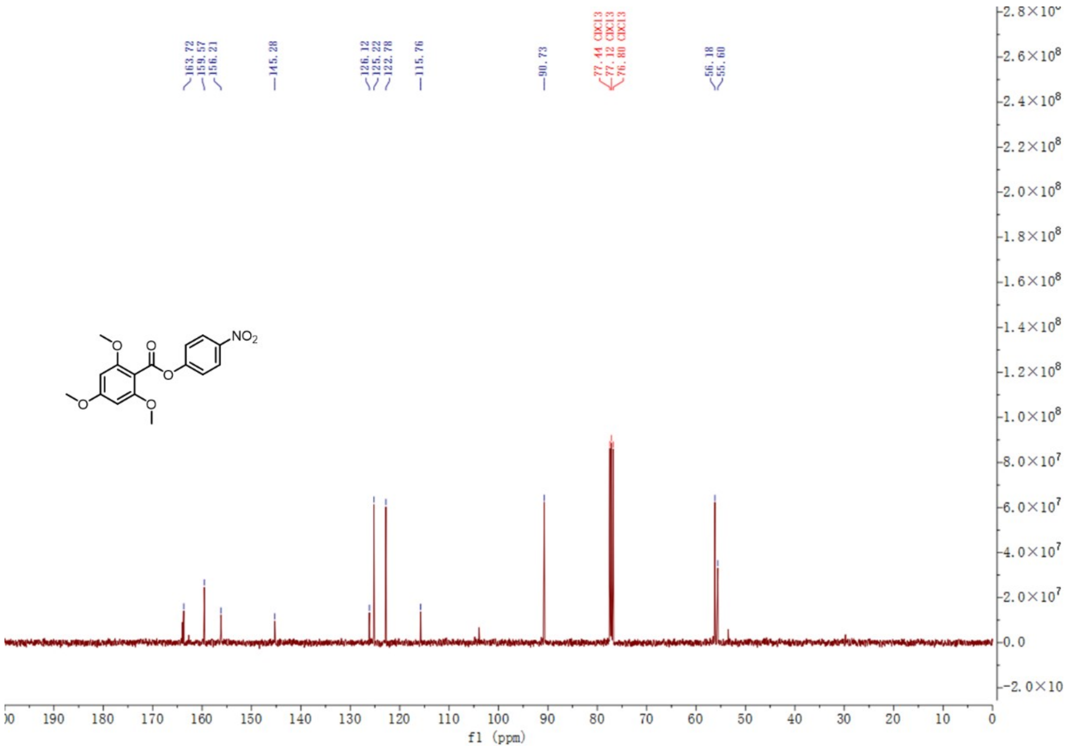

^{13}C NMR spectrum of **S6**

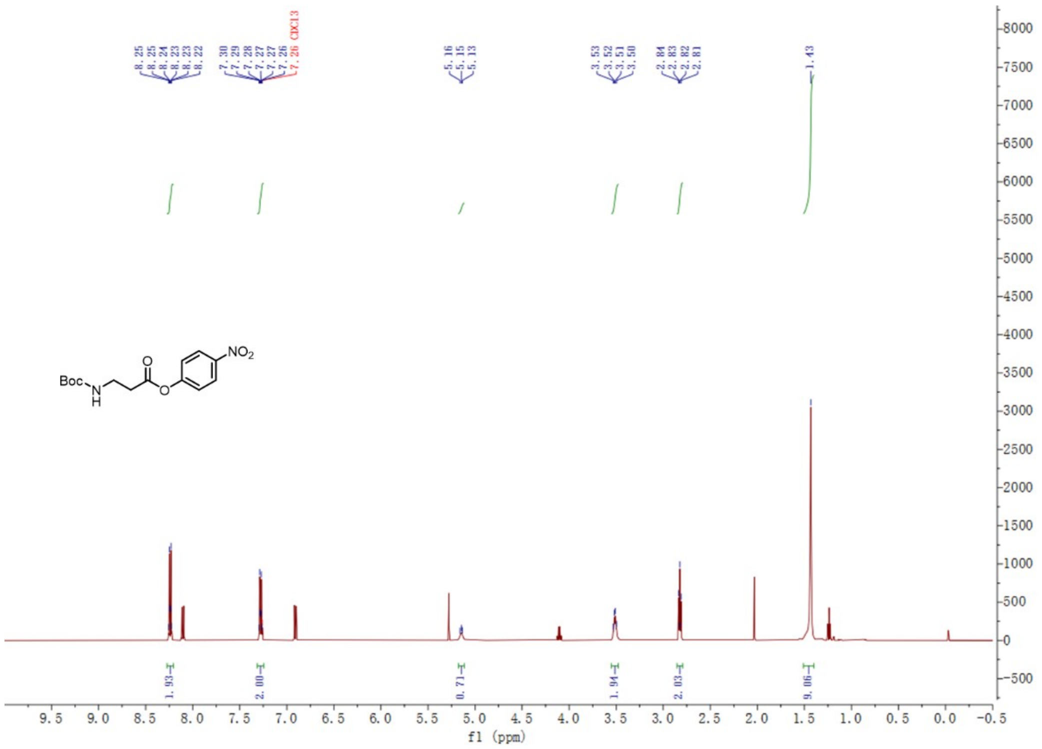

^{1}H NMR spectrum of **S7**

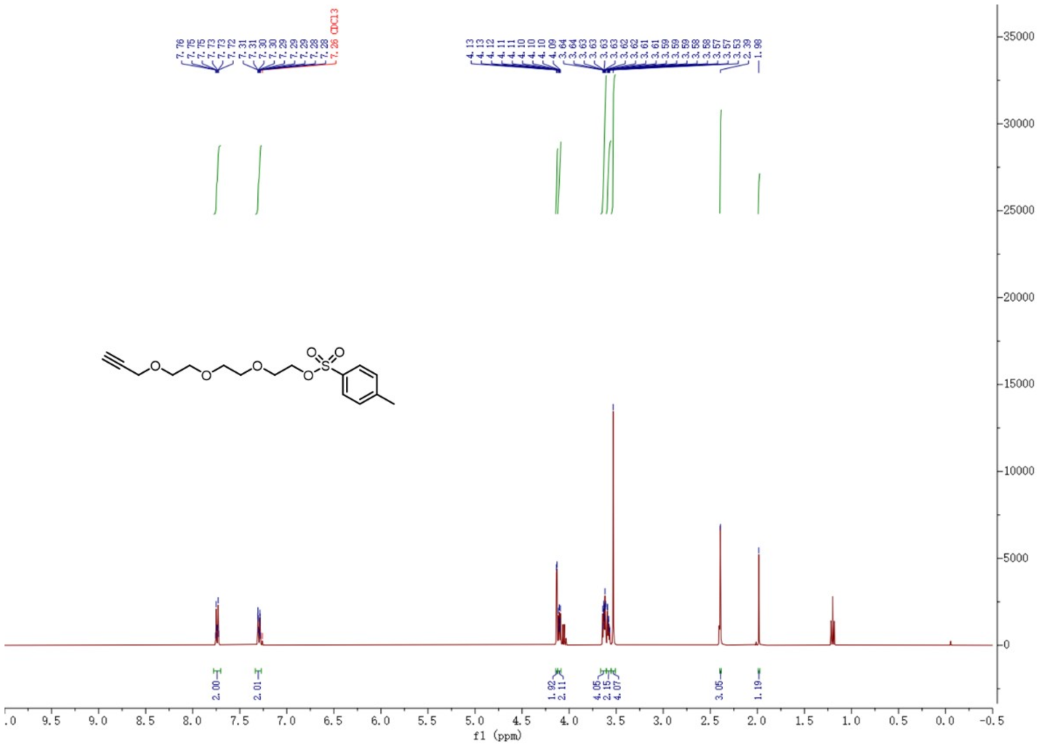

^{1}H NMR spectrum of **S8**

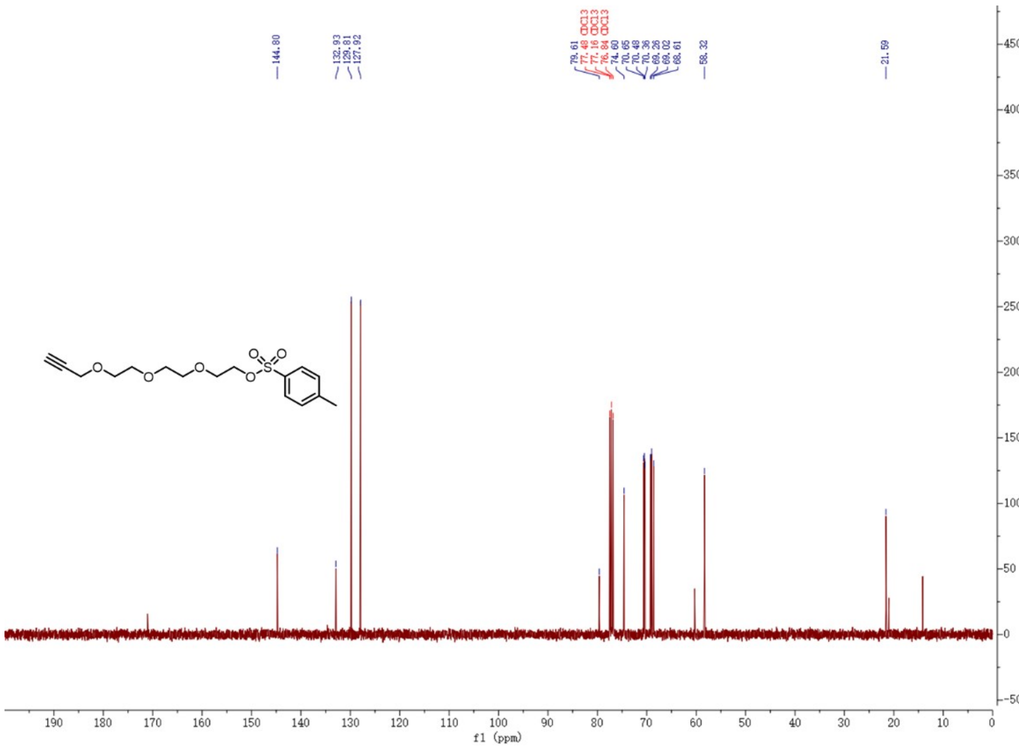

^{13}C NMR spectrum of **S8**

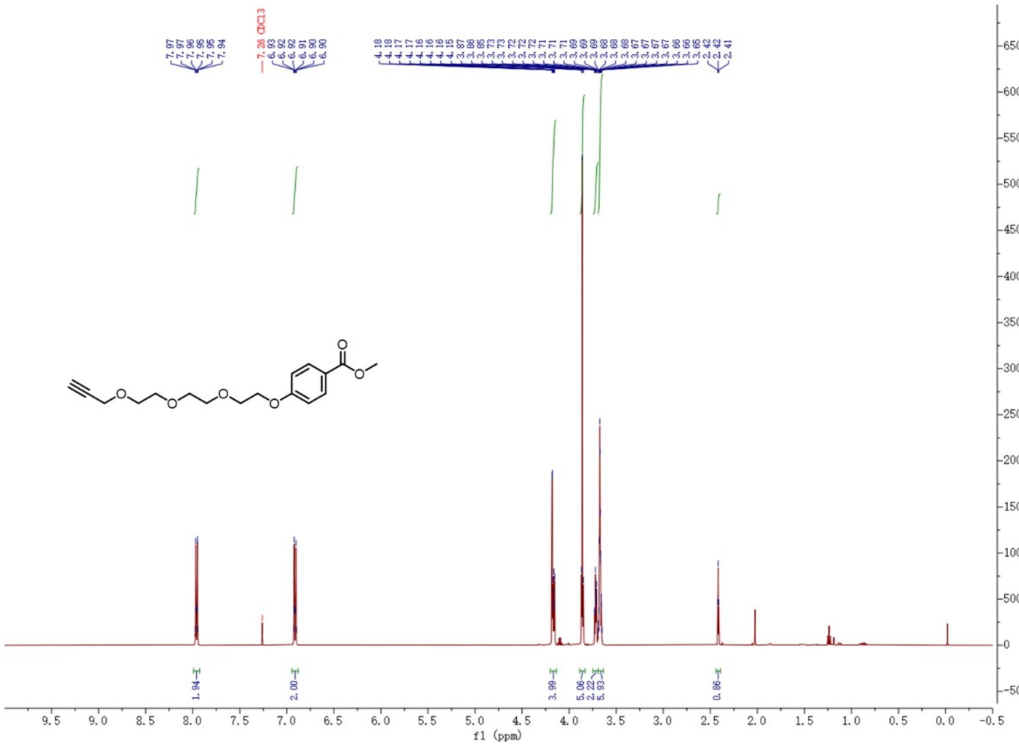

^{1}H NMR spectrum of **S9**

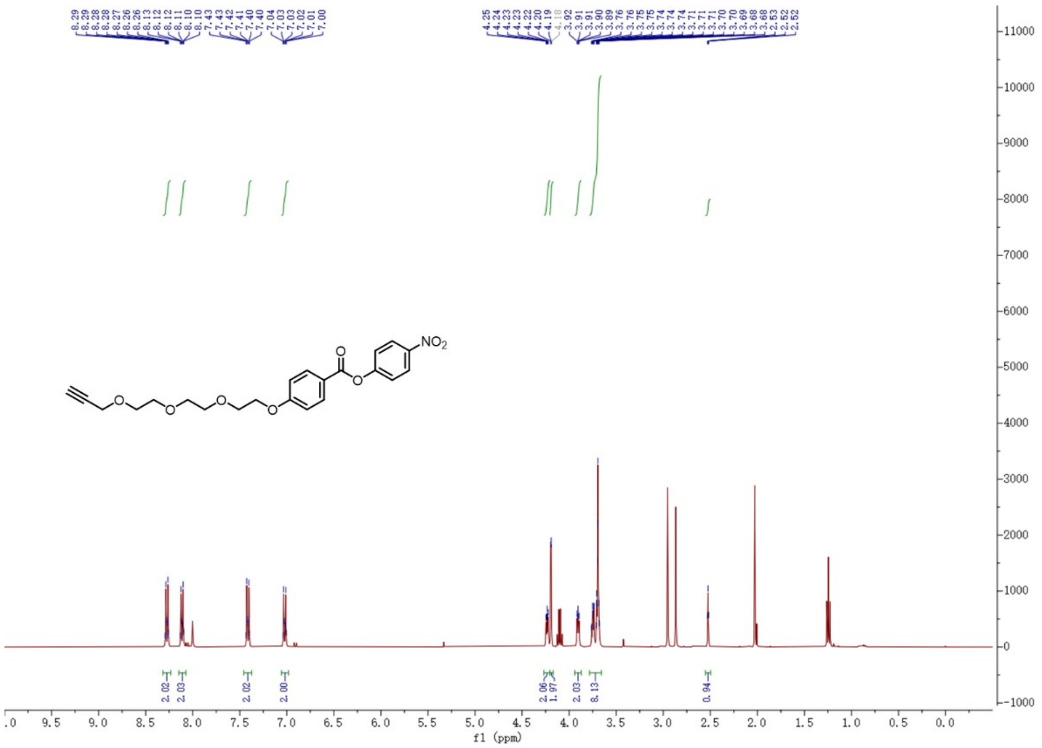

^{1}H NMR spectrum of **S10**

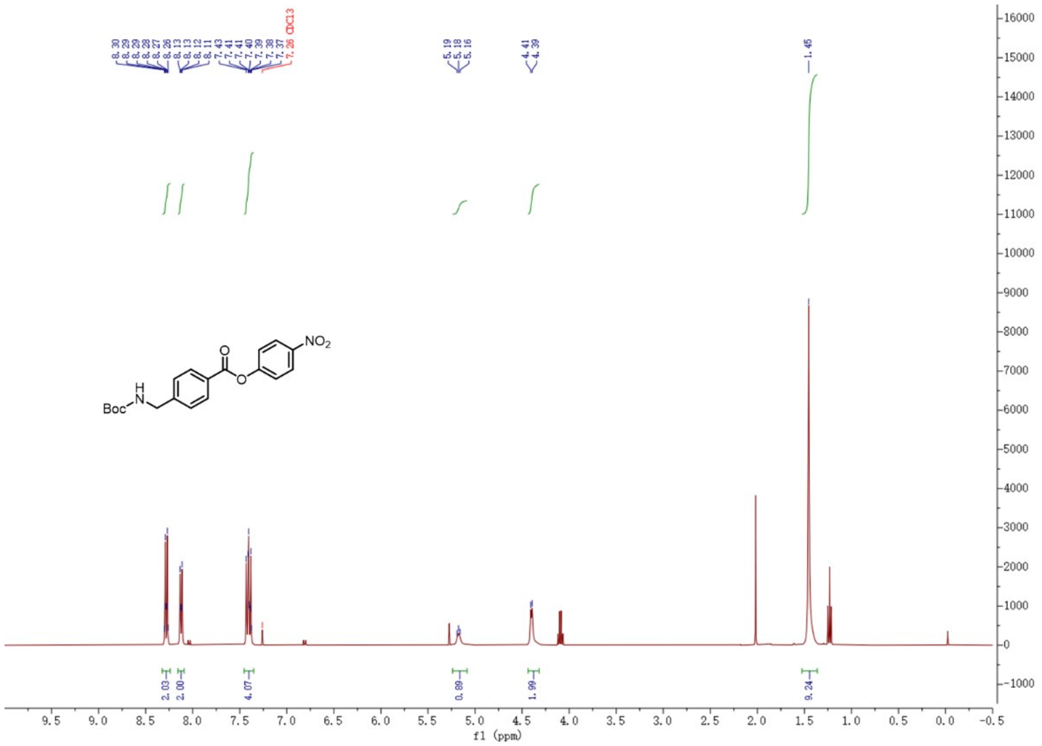

^{1}H NMR spectrum of **S11**

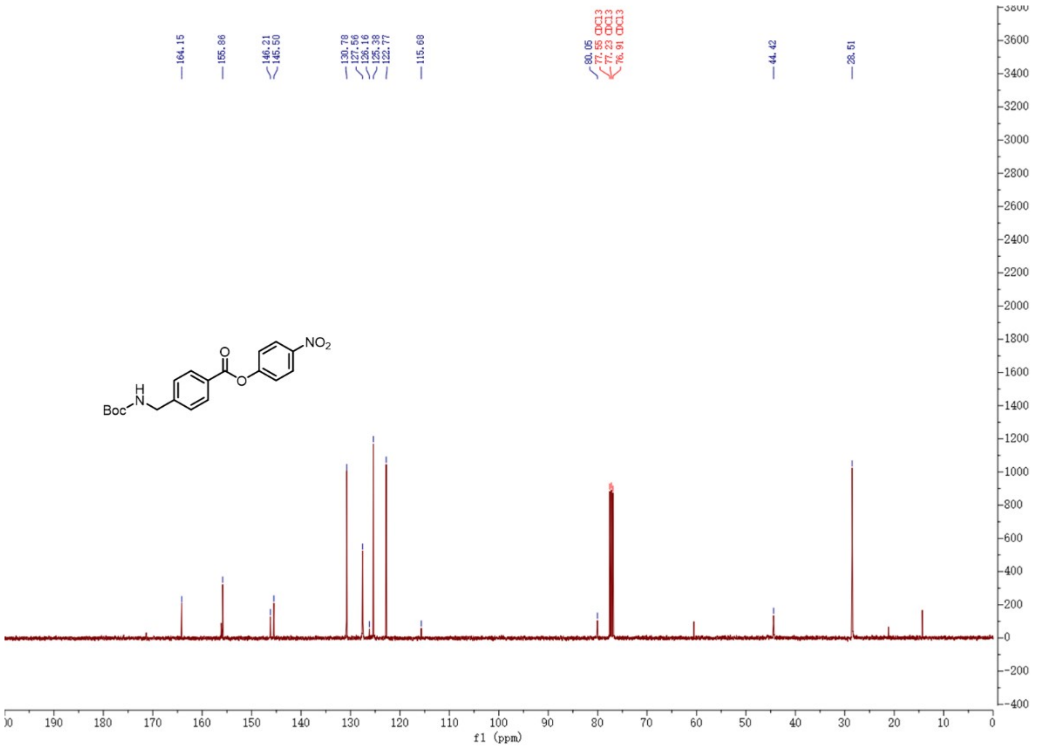

^{13}C NMR spectrum of **S11**

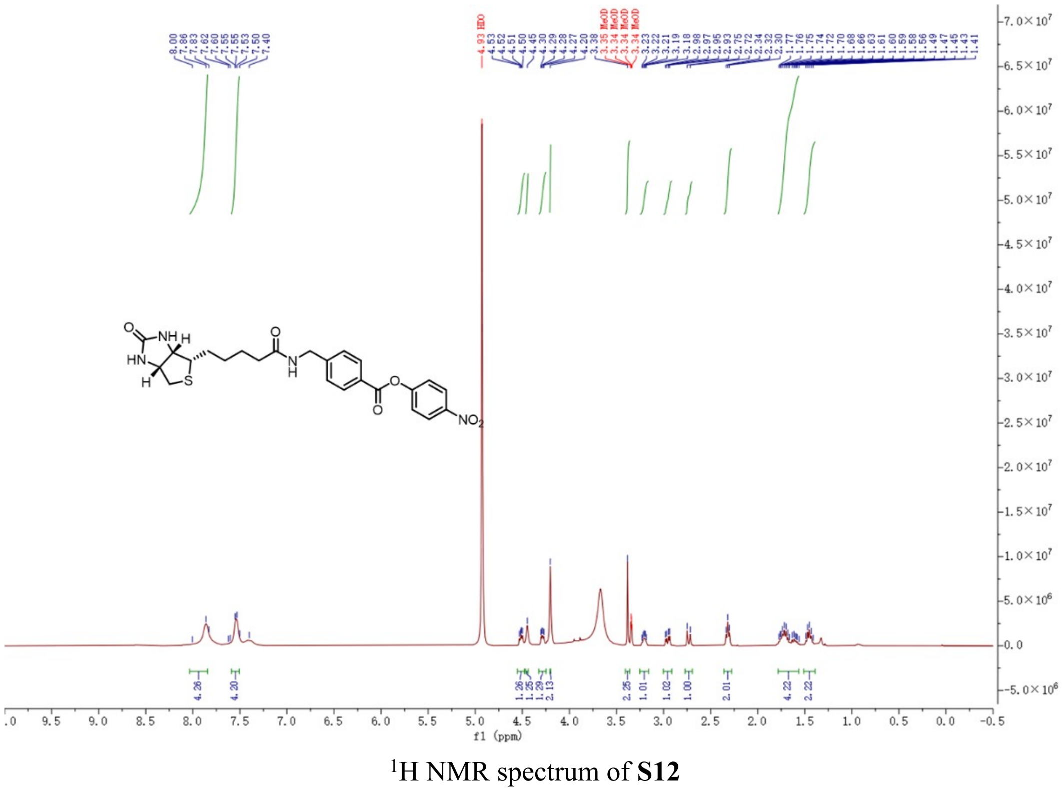

^{1}H NMR spectrum of **S12**

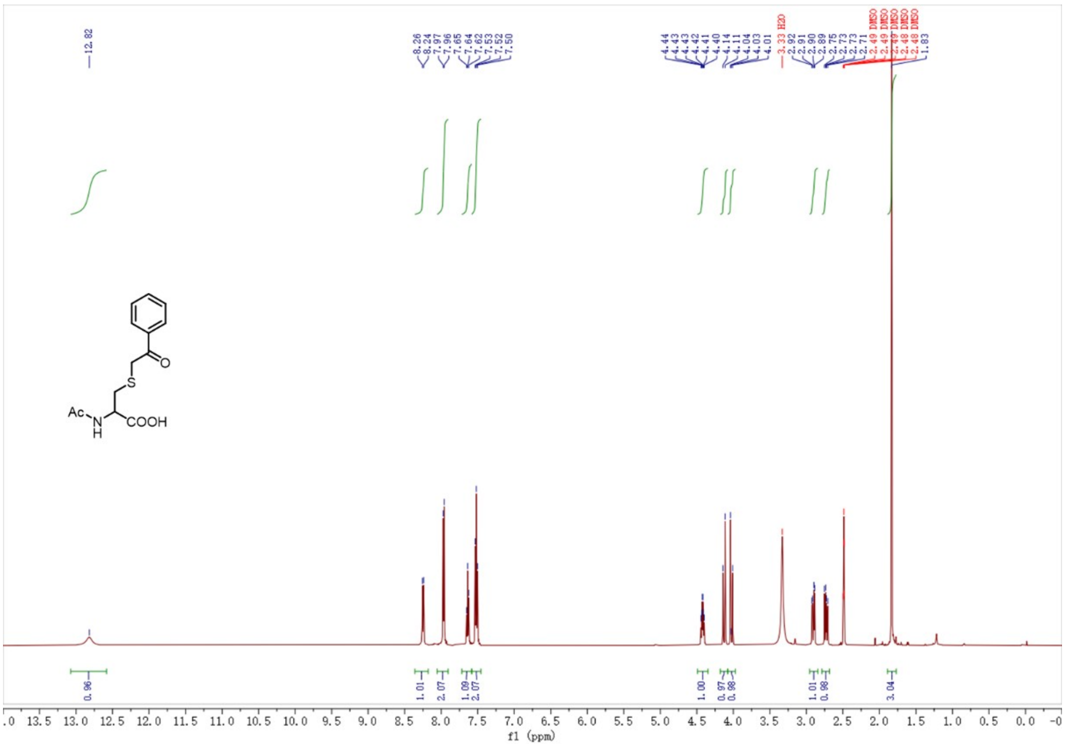

^{1}H NMR spectrum of **2a**

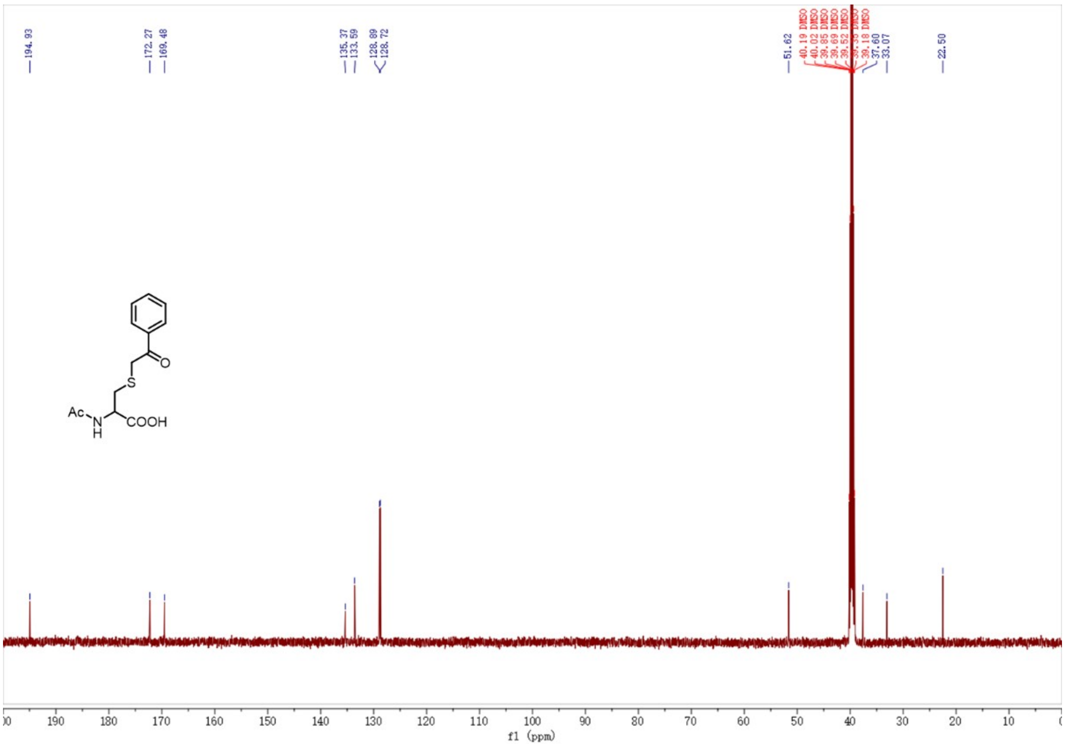

^{13}C NMR spectrum of **2a**

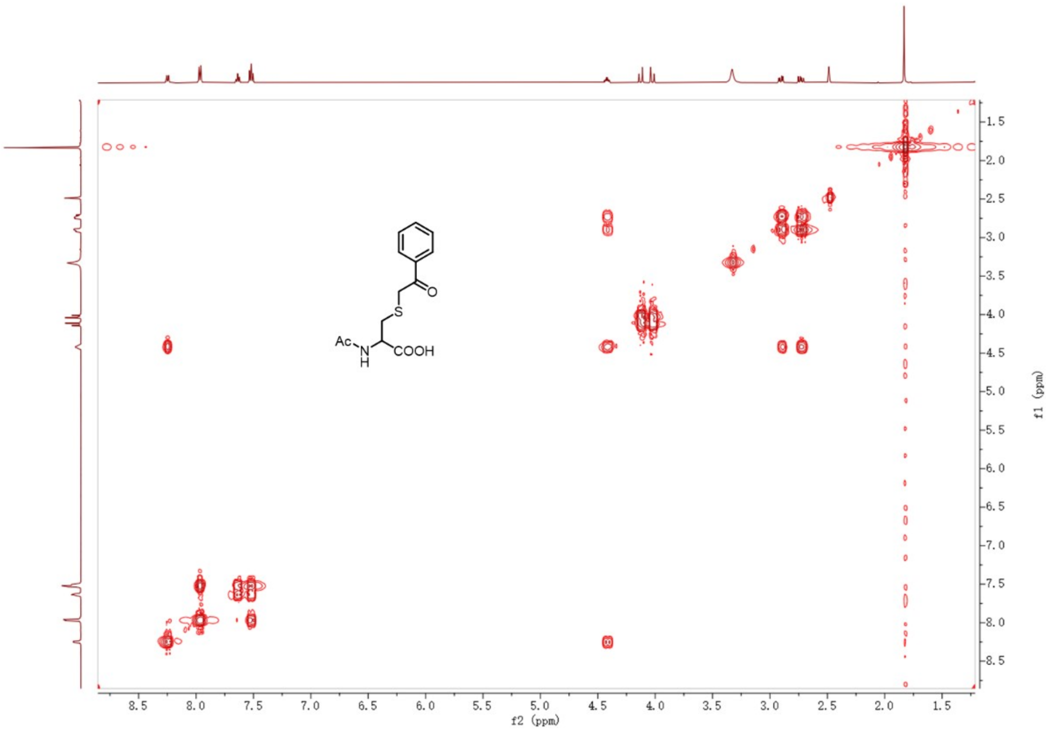

^{1}H-^{1}H COSY NMR spectrum of **2a**

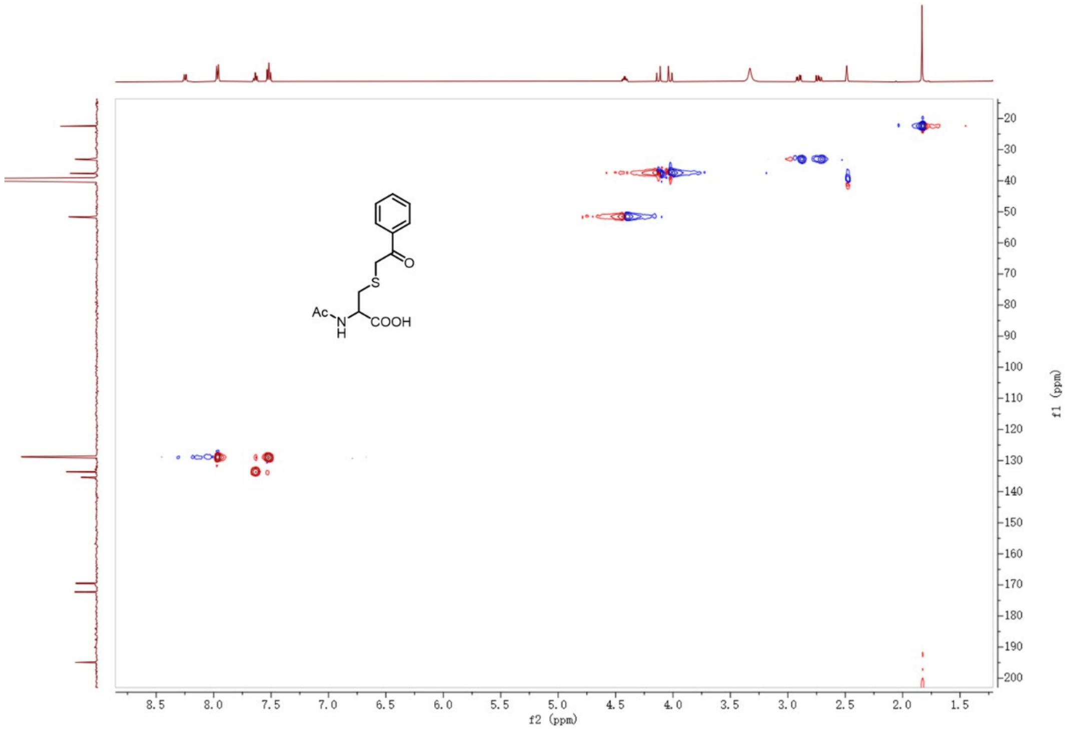

^{1}H-^{13}C HSQC NMR spectrum of **2a**

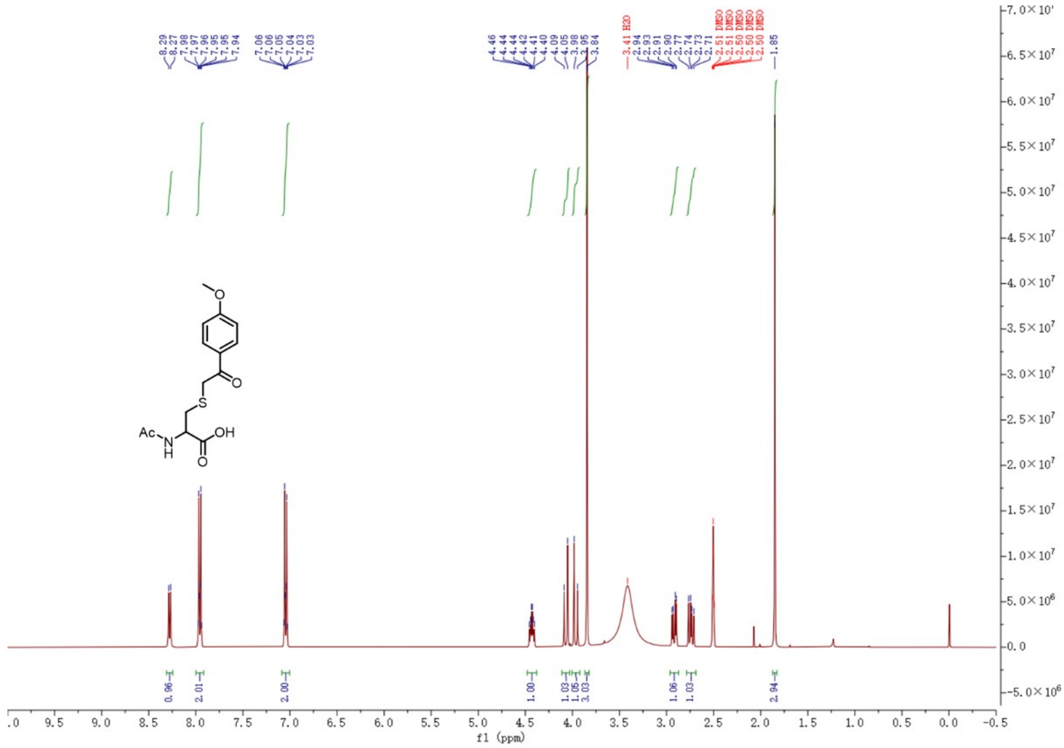

^{1}H NMR spectrum of **2b**

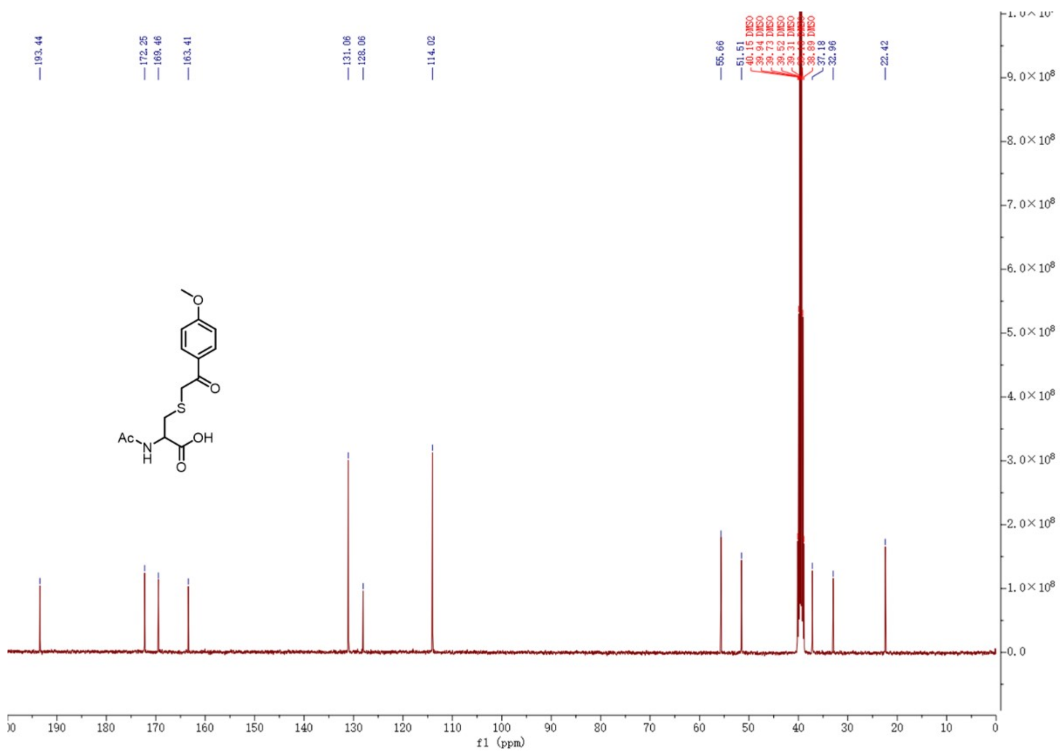

^{13}C NMR spectrum of **2b**

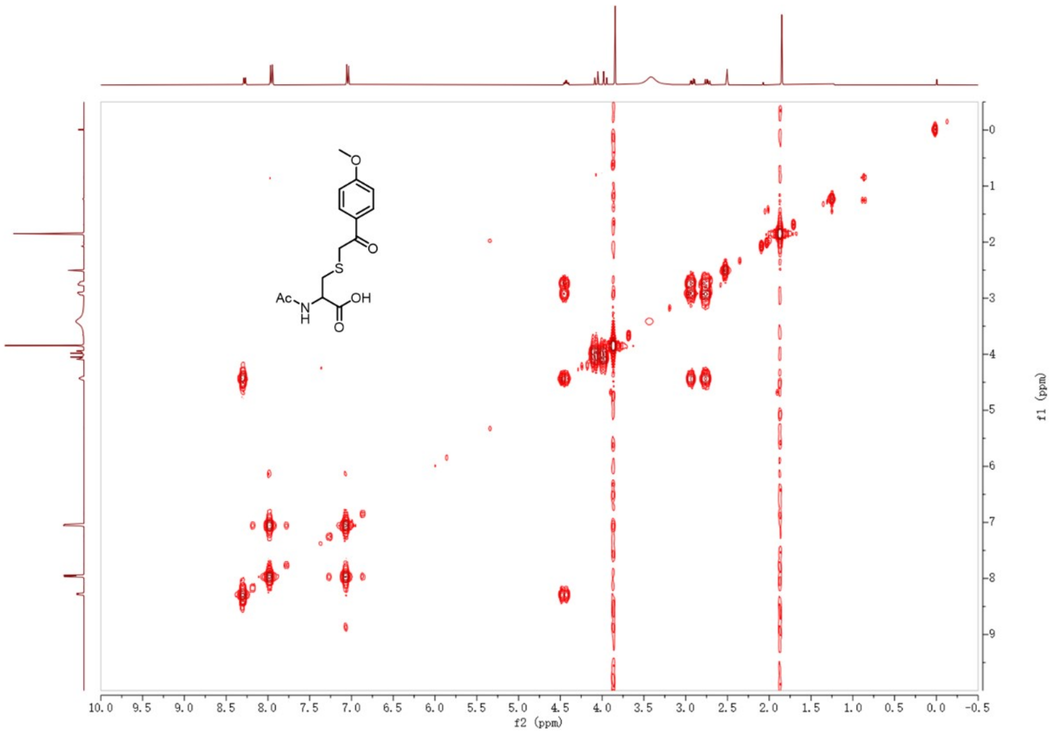

^{1}H-^{1}H COSY NMR spectrum of **2b**

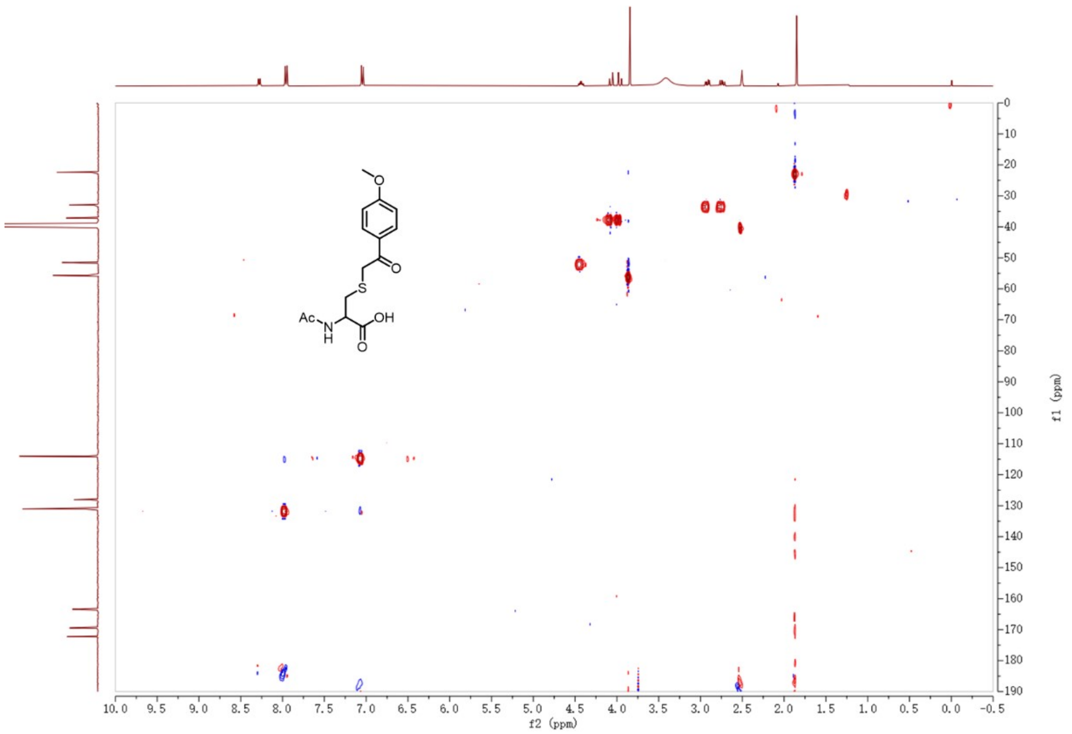

^{1}H-^{13}C HSQC NMR spectrum of **2b**

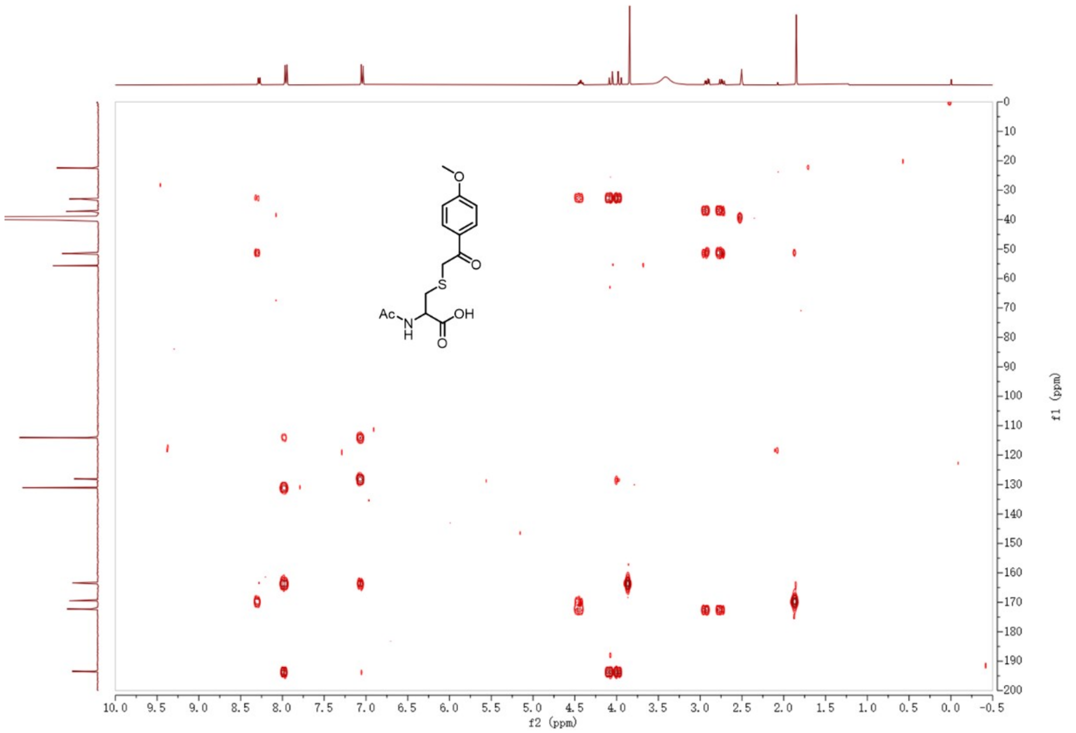

^{1}H-^{13}C HMBC NMR spectrum of **2b**

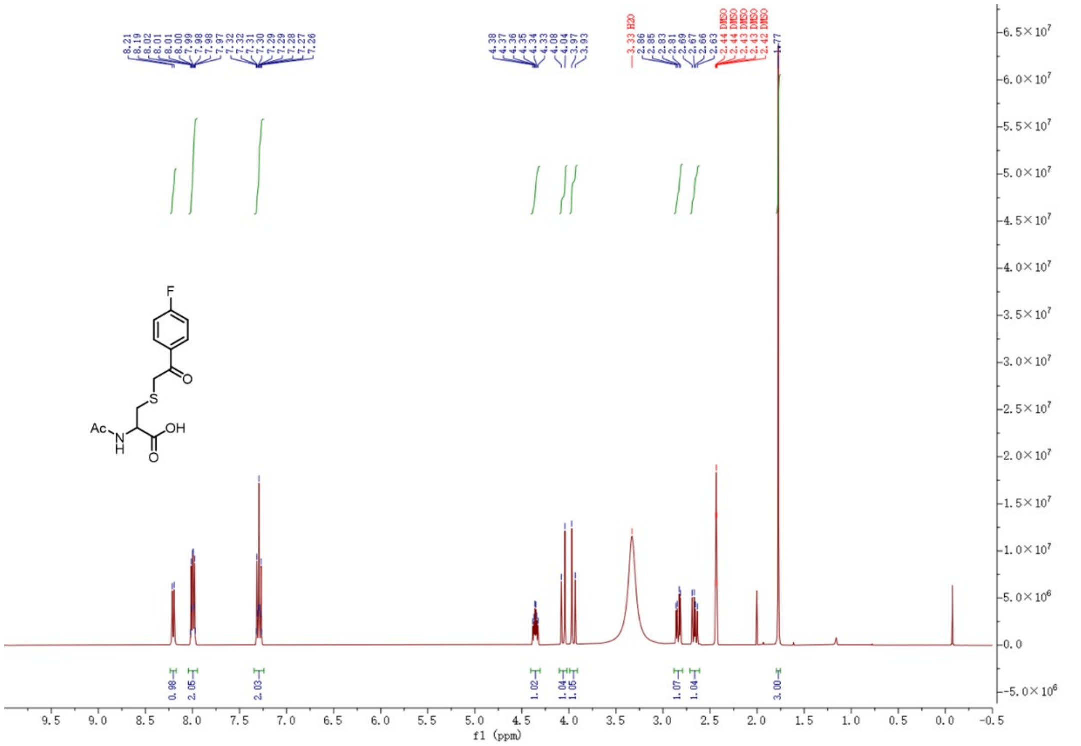

^{1}H NMR spectrum of **2c**

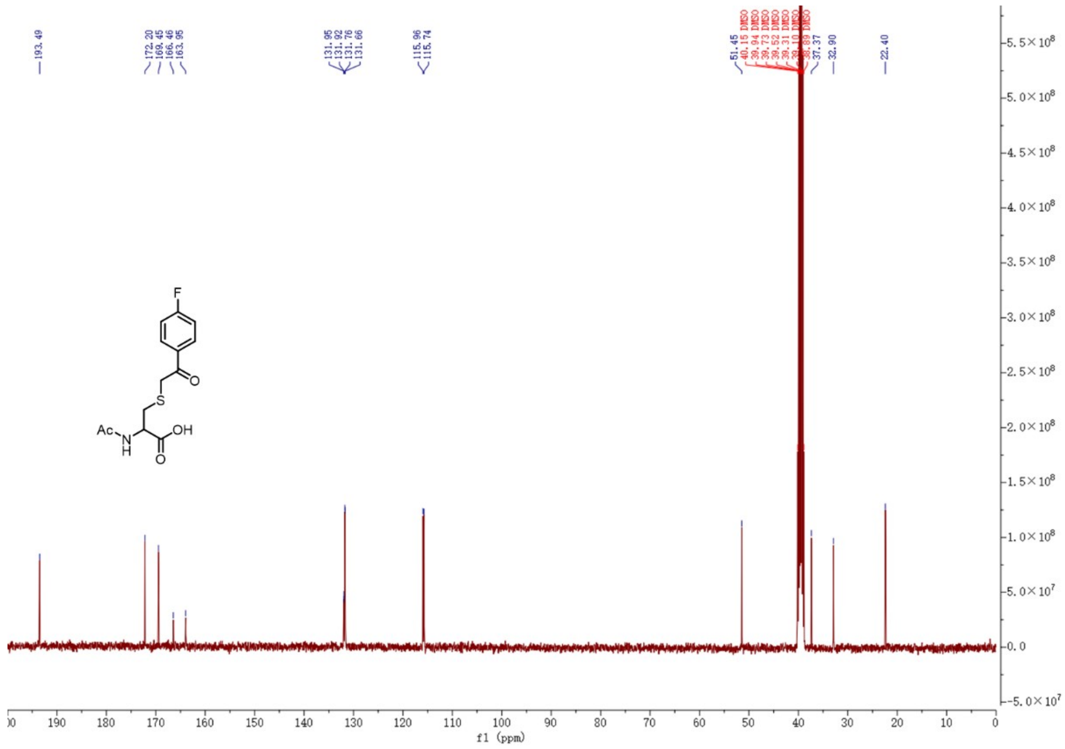

^{13}C NMR spectrum of **2c**

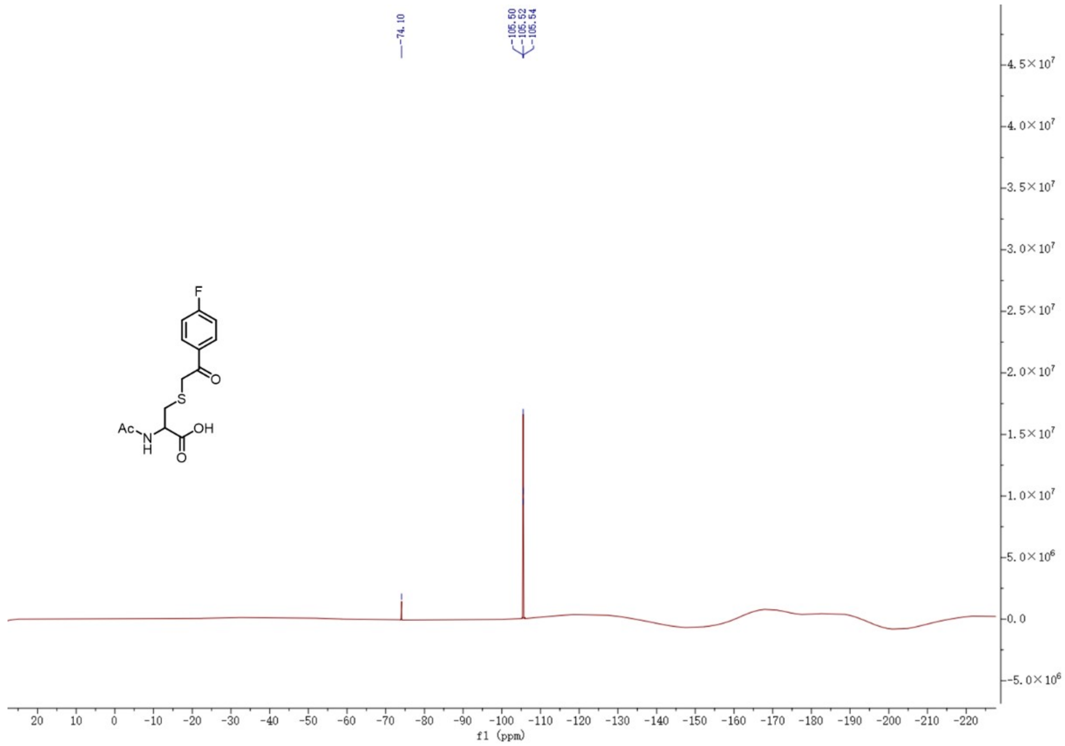

^{19}F NMR spectrum of **2c**

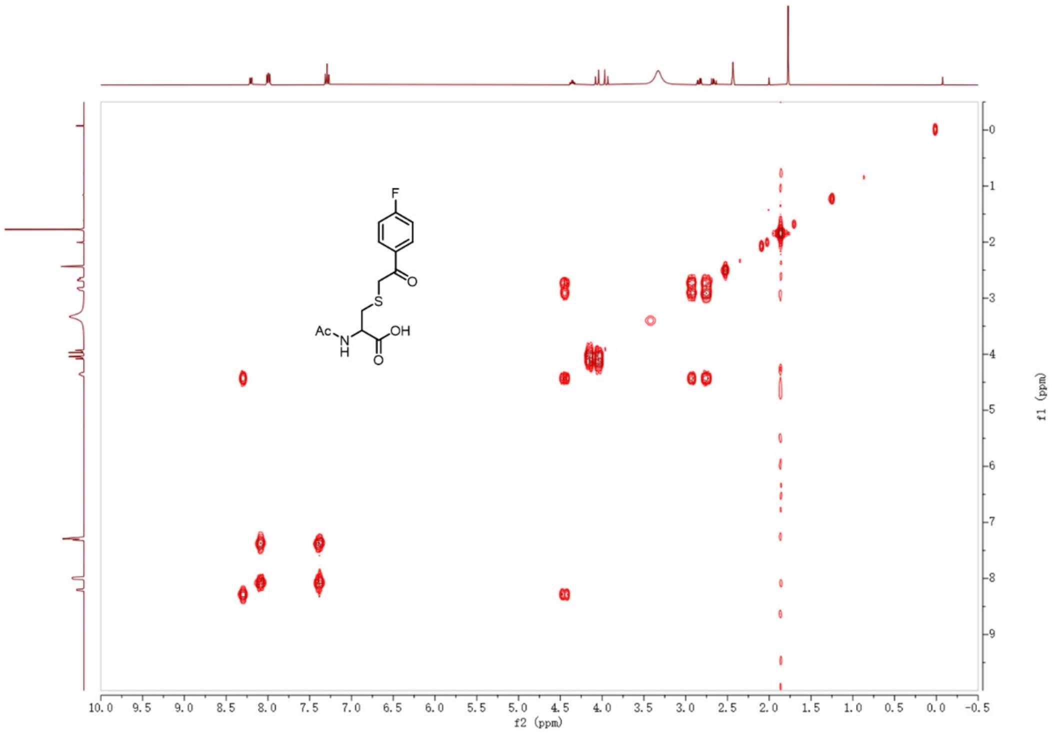

^{1}H-^{1}H COSY NMR spectrum of **2c**

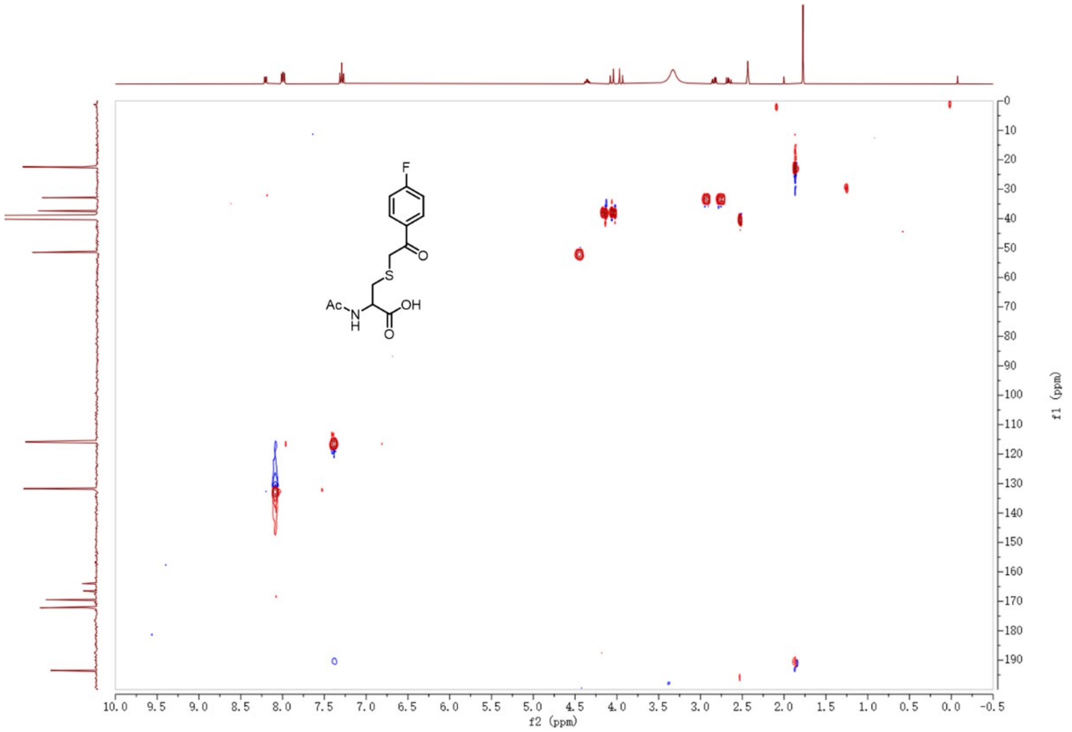

^{1}H-^{13}C HSQC NMR spectrum of **2c**

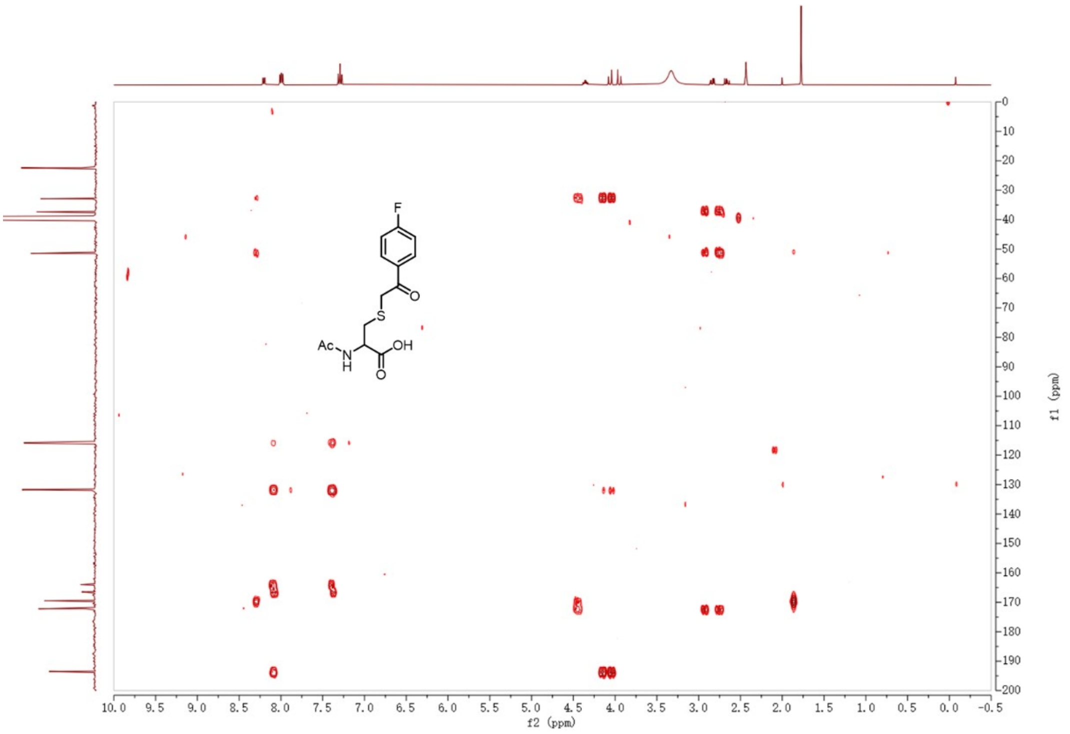

^{1}H-^{13}C HMBC NMR spectrum of **2c**

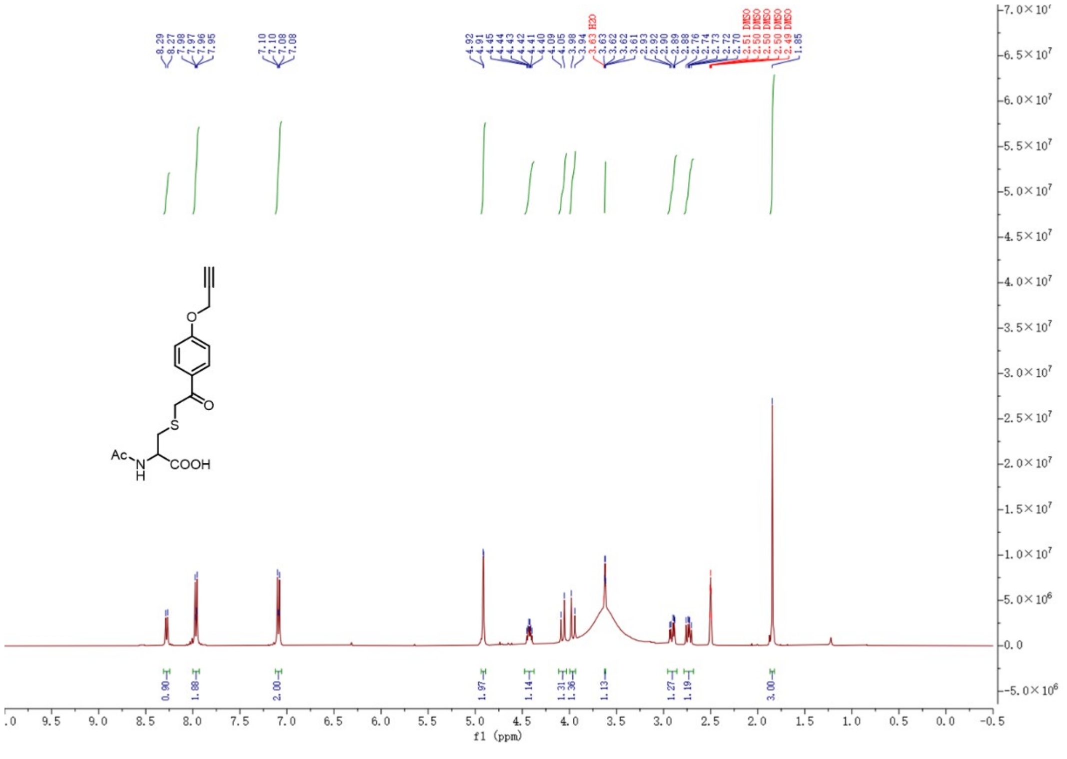

^{1}H NMR spectrum of **2d**

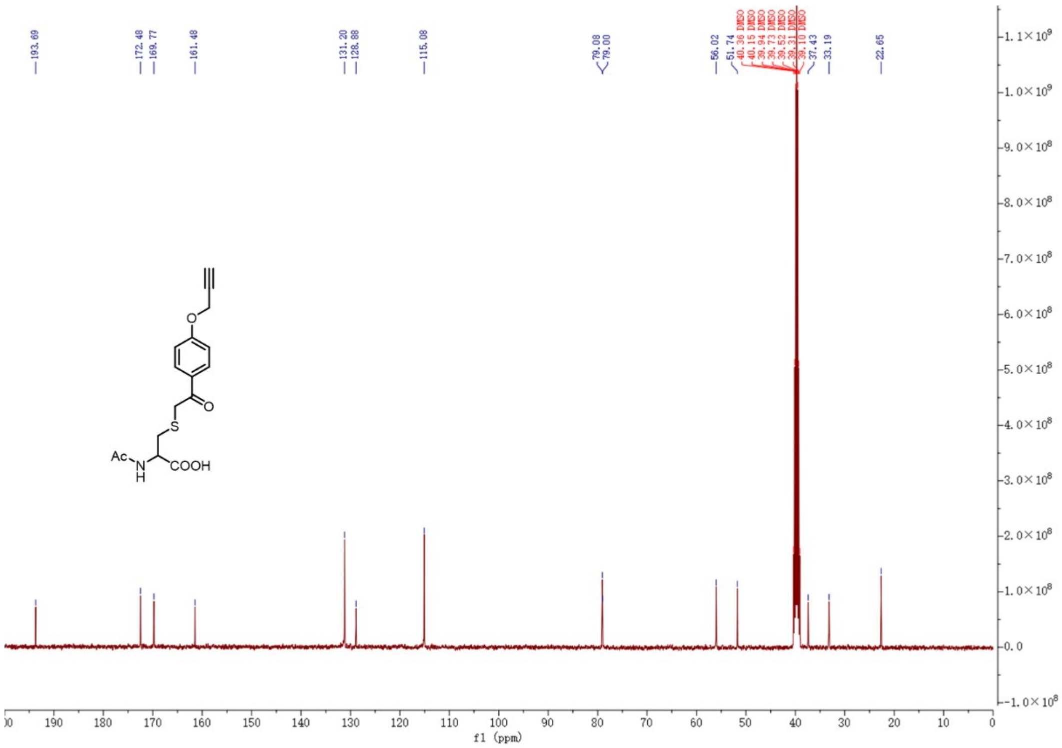

^{13}C NMR spectrum of **2d**

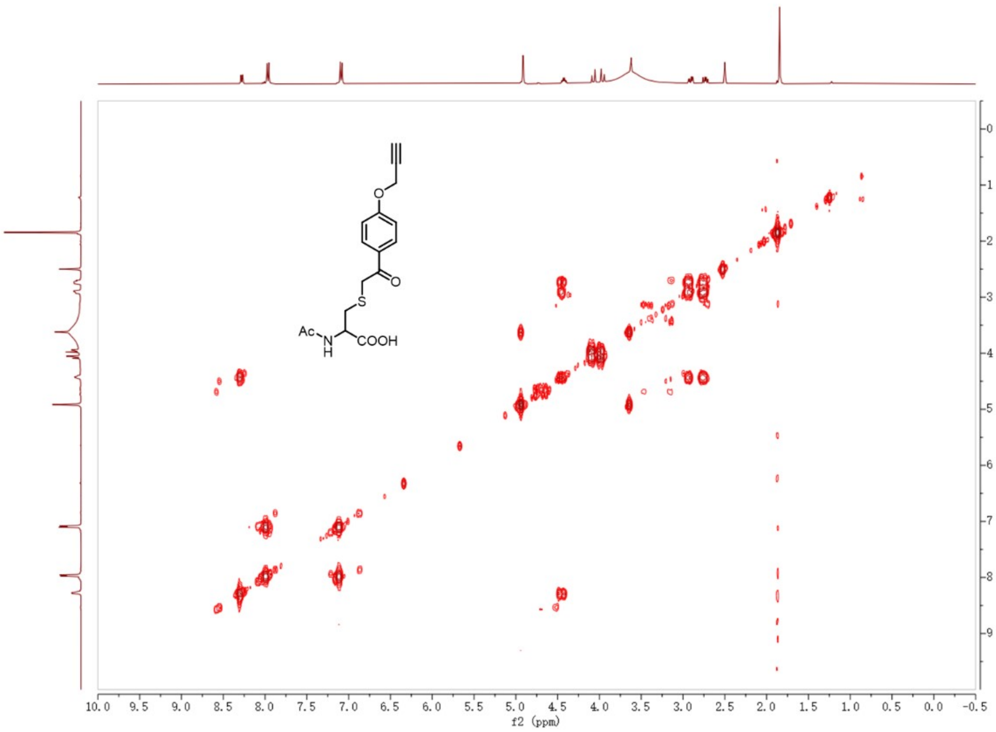

^{1}H-^{1}H COSY NMR spectrum of **2d**

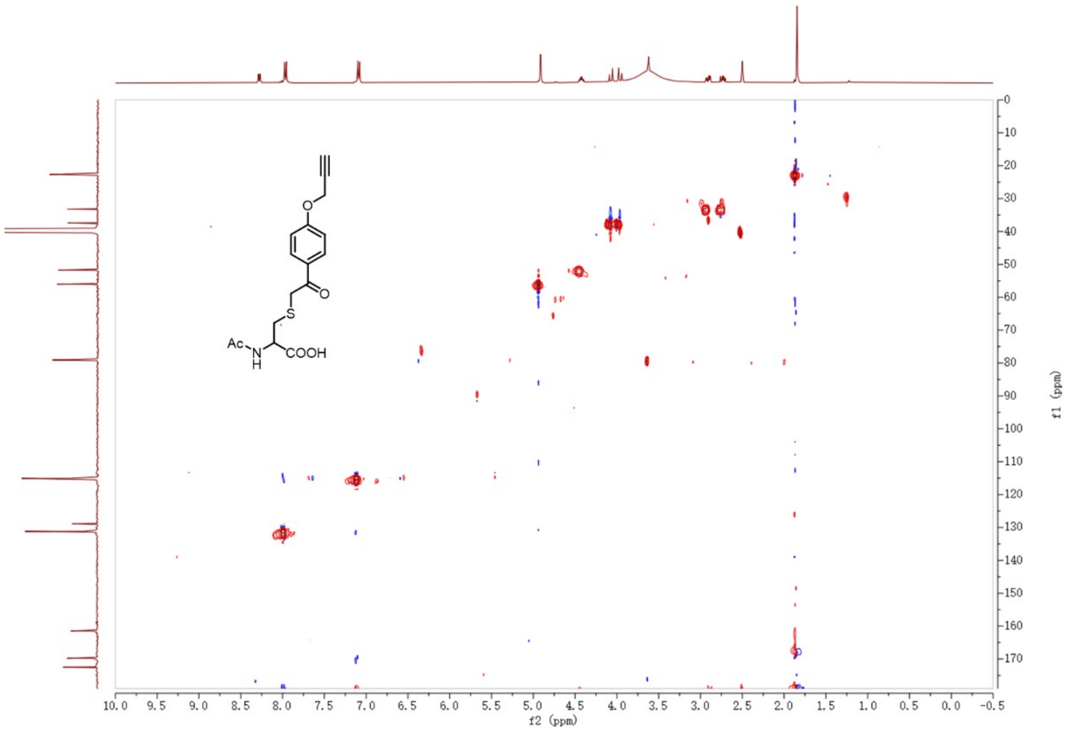

^{1}H-^{13}C HSQC NMR spectrum of **2d**

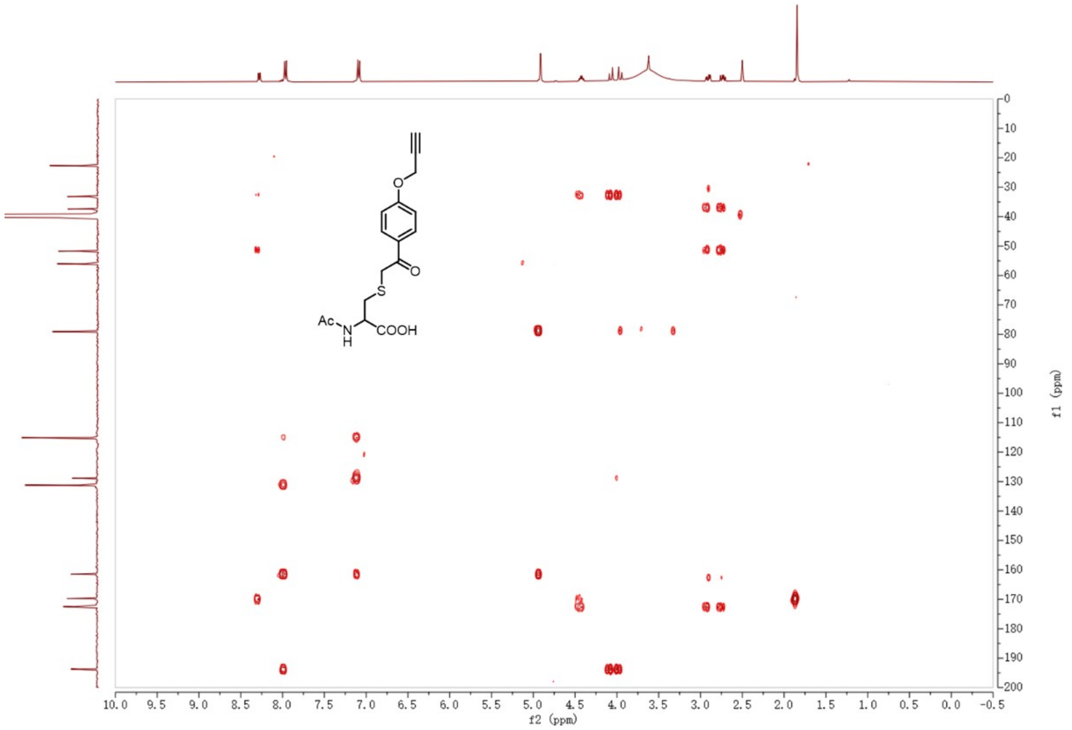

^{1}H-^{13}C HMBC NMR spectrum of **2d**

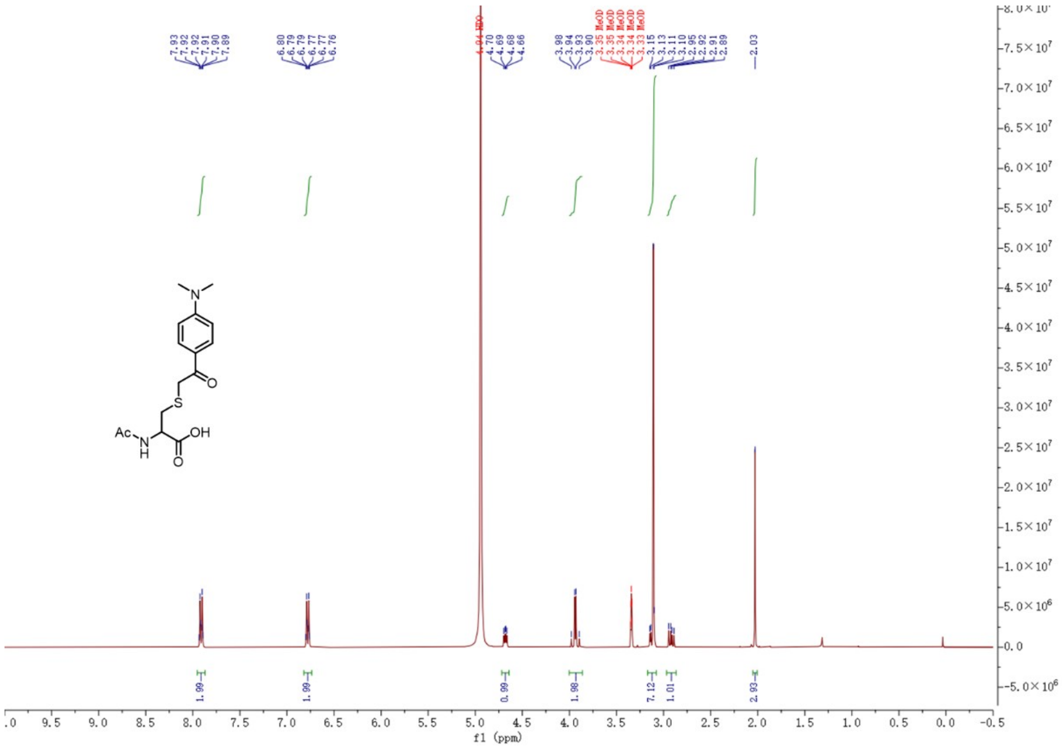

^{1}H NMR spectrum of **2e**

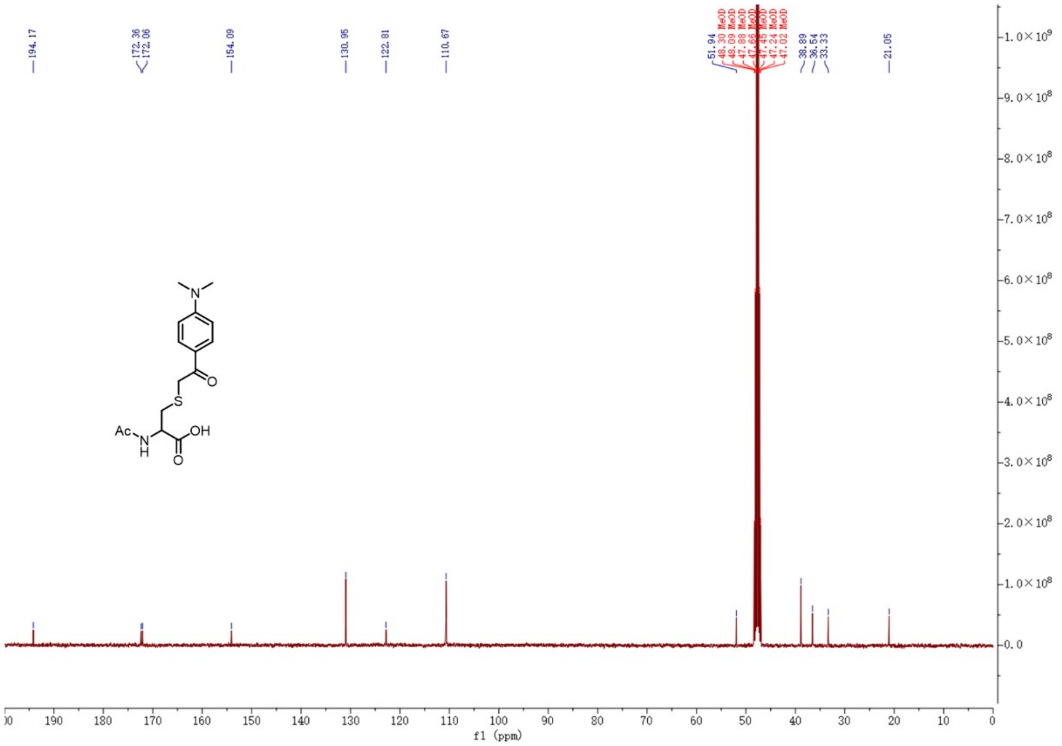

^{13}C NMR spectrum of **2e**

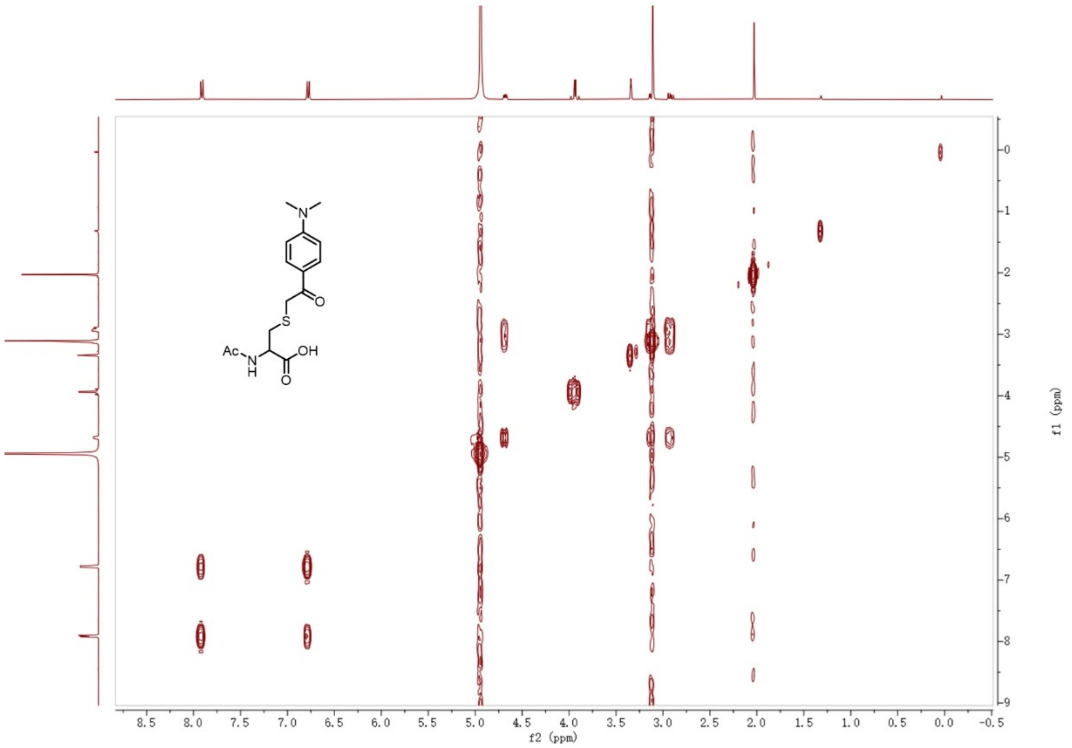

^{1}H-^{1}H COSY NMR spectrum of **2e**

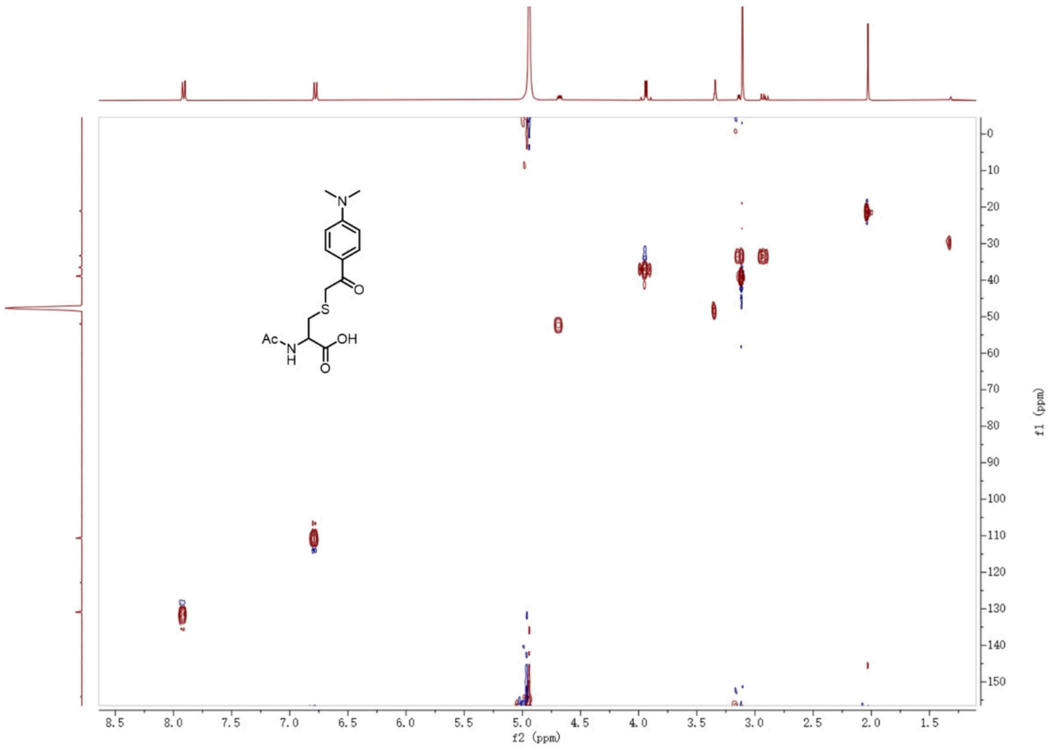

^{1}H-^{13}C HSQC NMR spectrum of **2e**

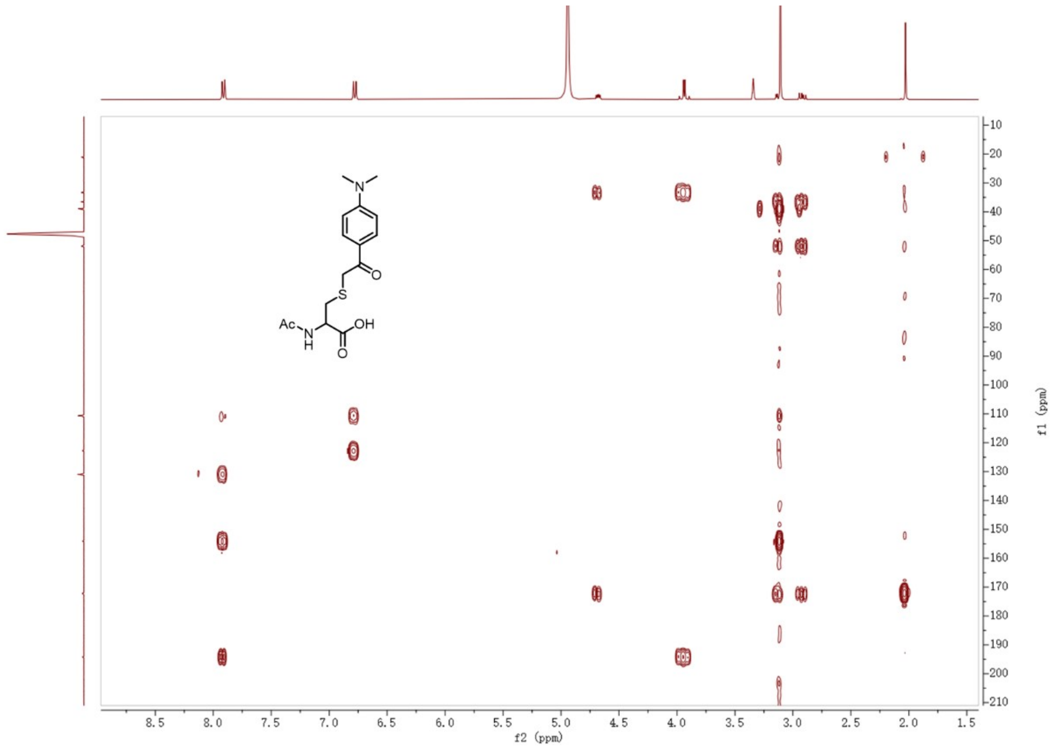

^{1}H-^{13}C HMBC NMR spectrum of **2e**

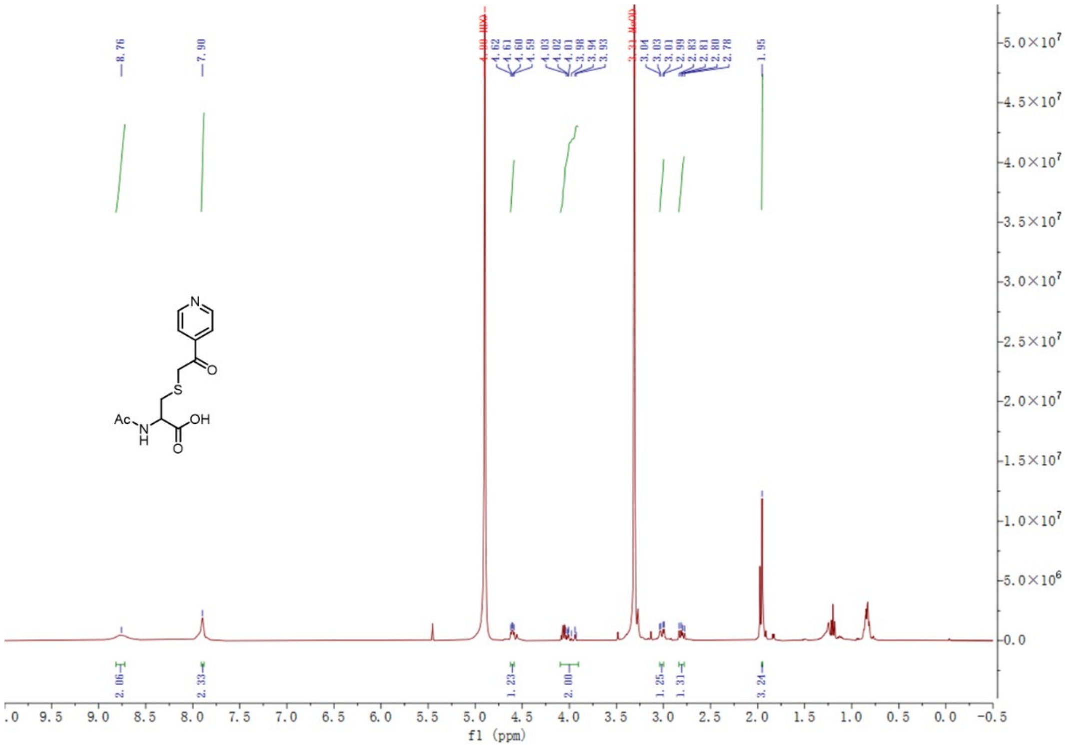

^{1}H NMR spectrum of **2g**

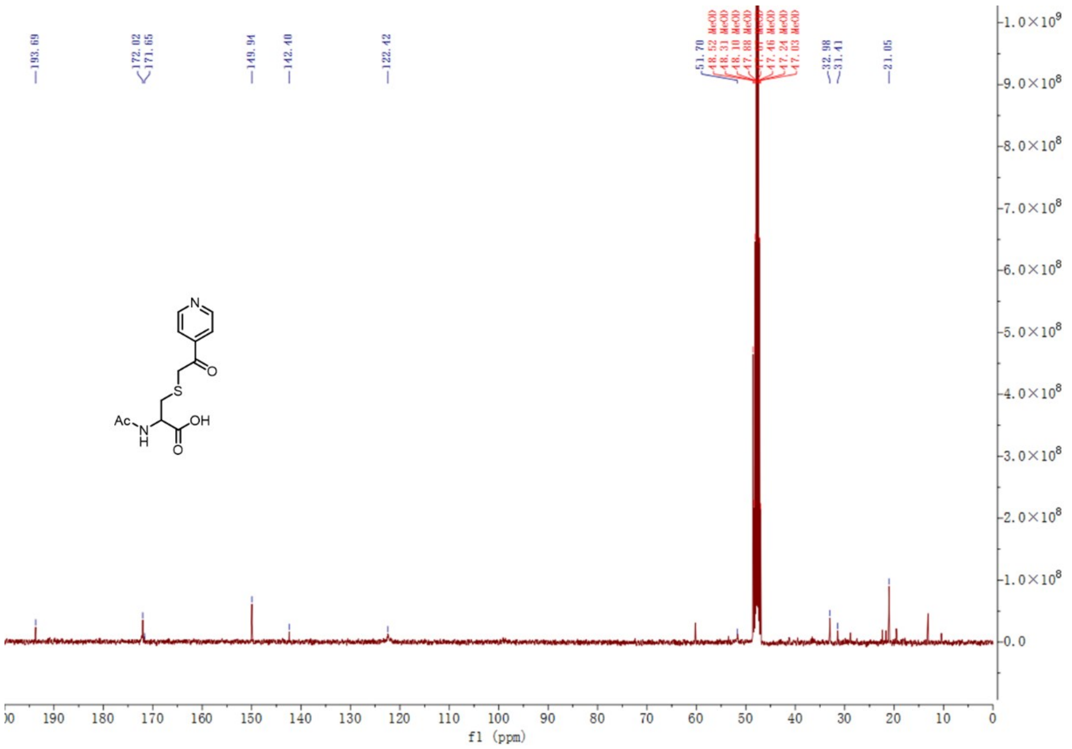

^{13}C NMR spectrum of **2g**

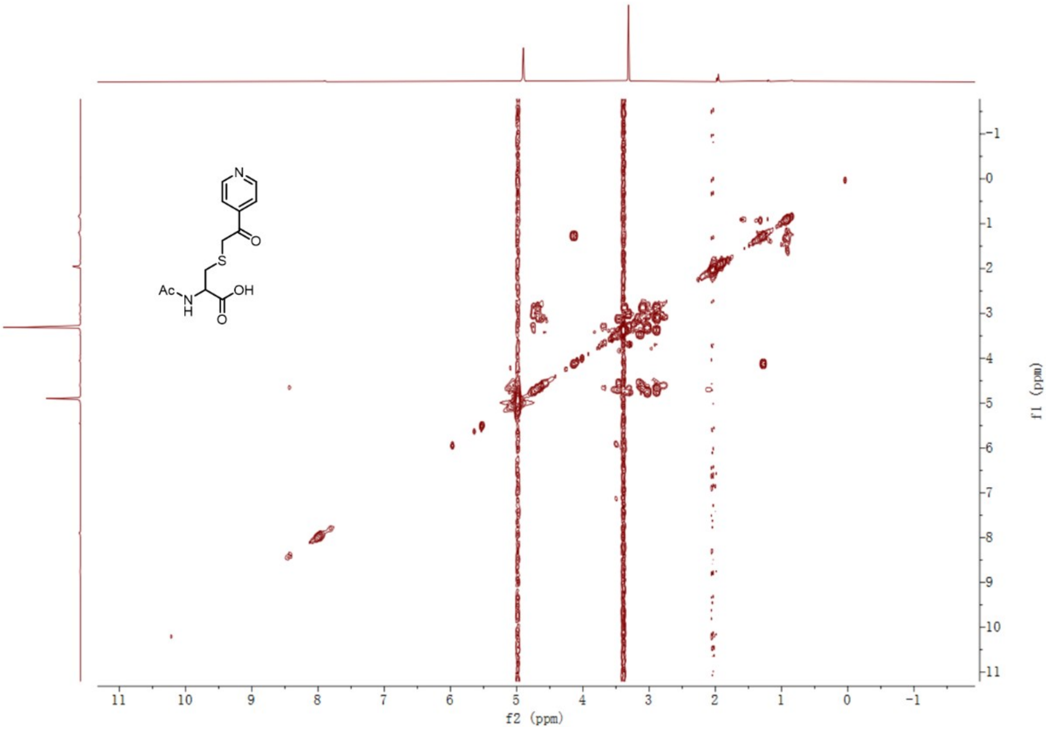

^{1}H-^{1}H COSY NMR spectrum of **2g**

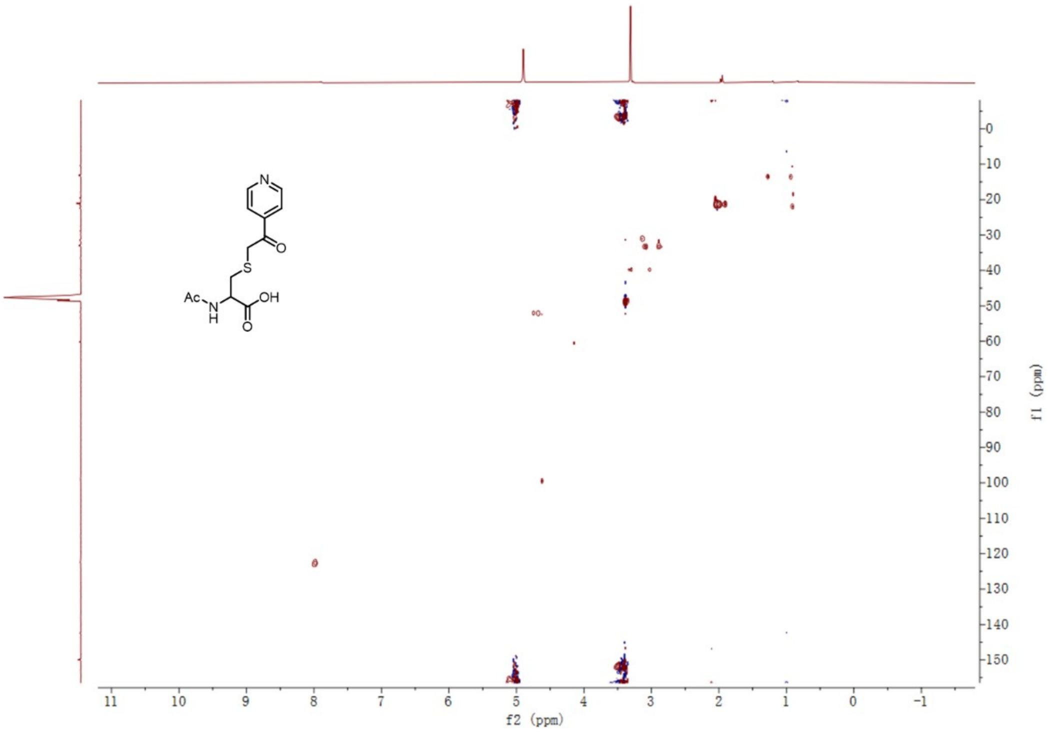

^{1}H-^{13}C HSQC NMR spectrum of **2g**

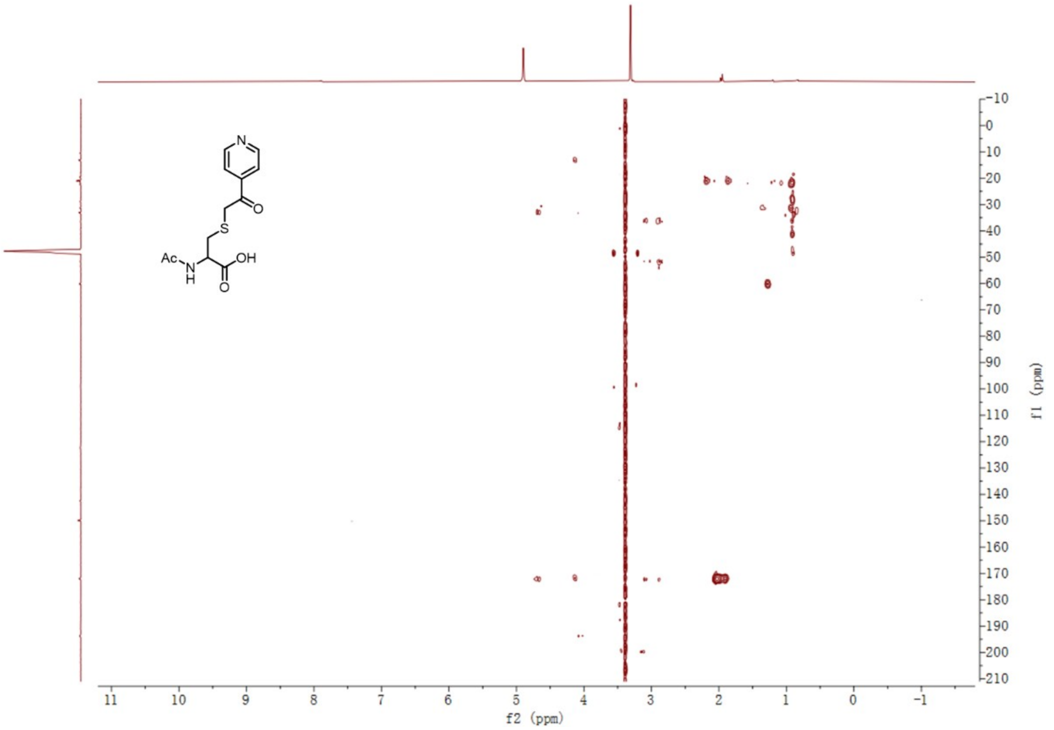

^{1}H-^{13}C HMBC NMR spectrum of **2g**

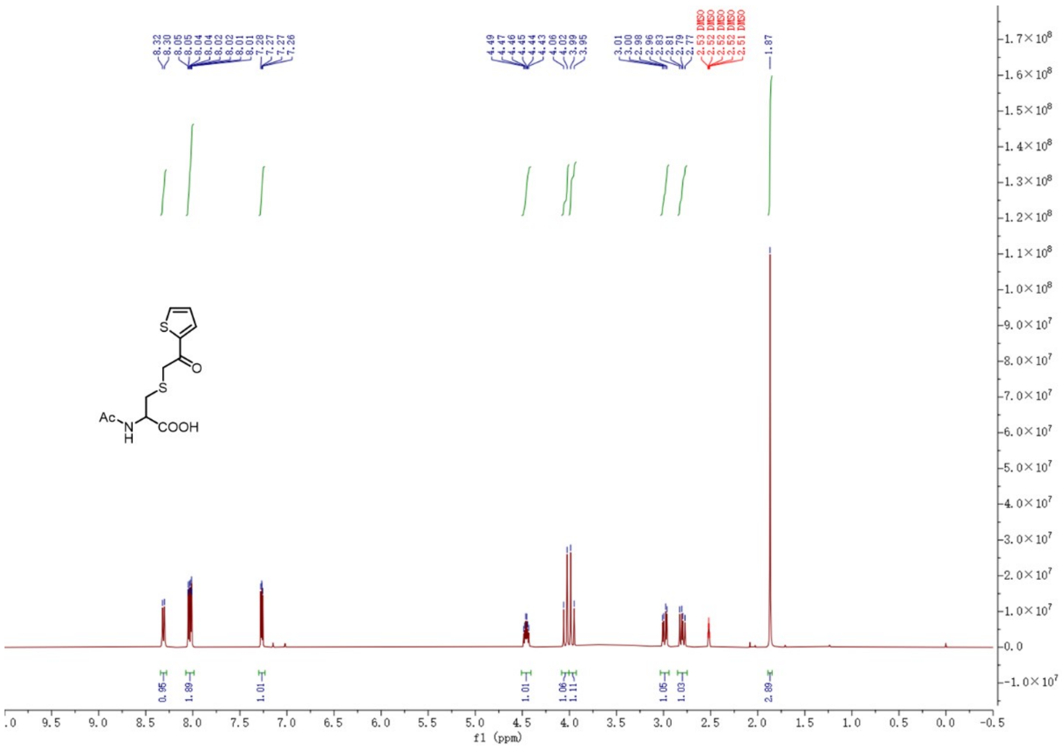

^{1}H NMR spectrum of **2h**

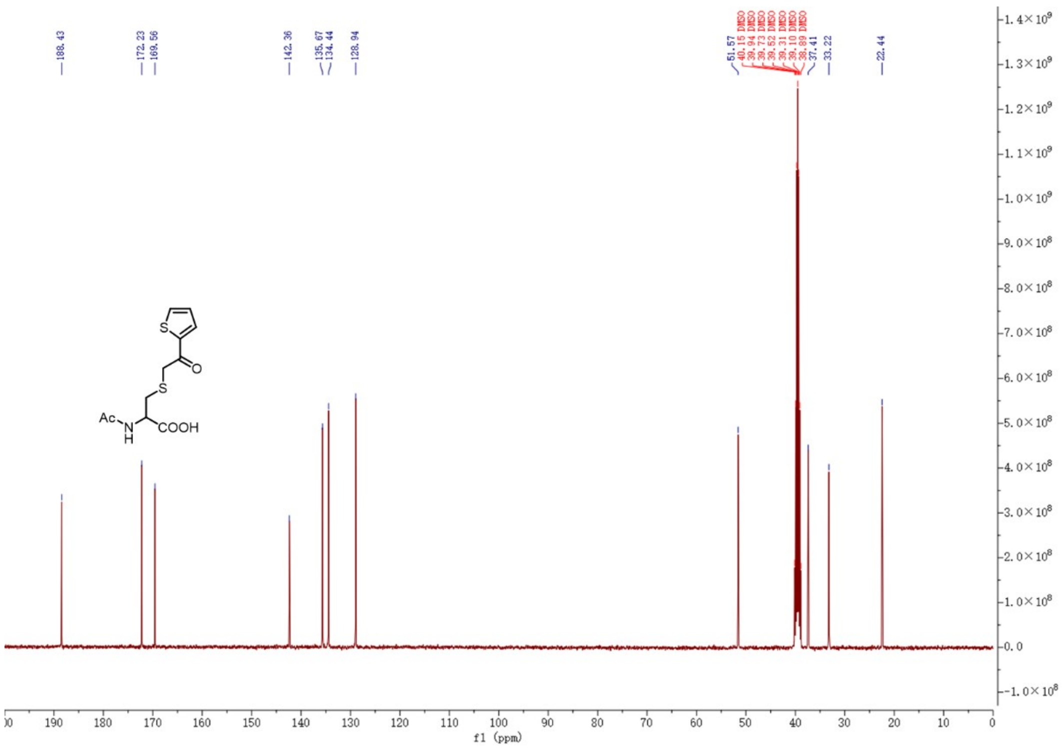

^{13}C NMR spectrum of **1h**

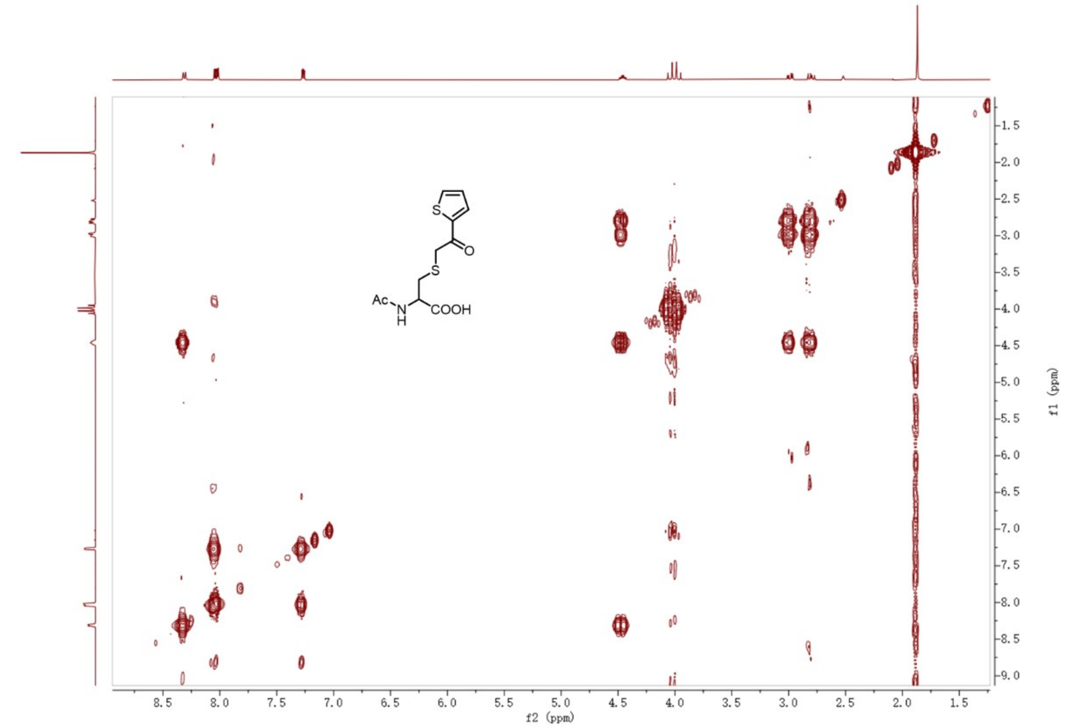

^{1}H-^{1}H COSY NMR spectrum of **2h**

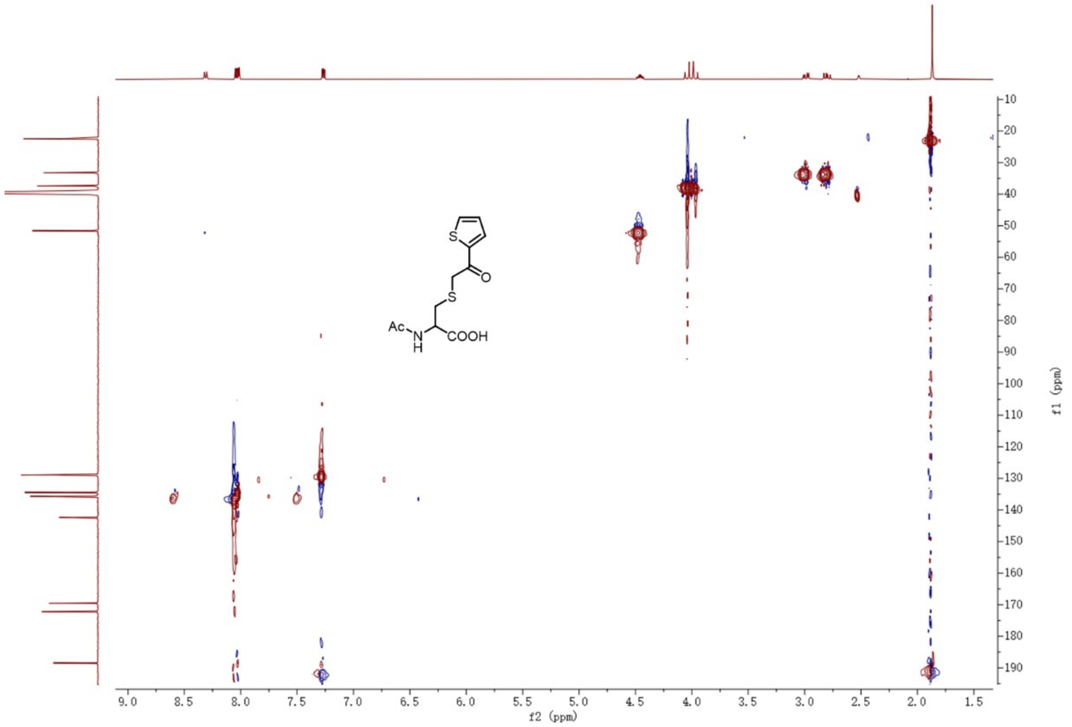

^{1}H-^{13}C HSQC NMR spectrum of **2h**

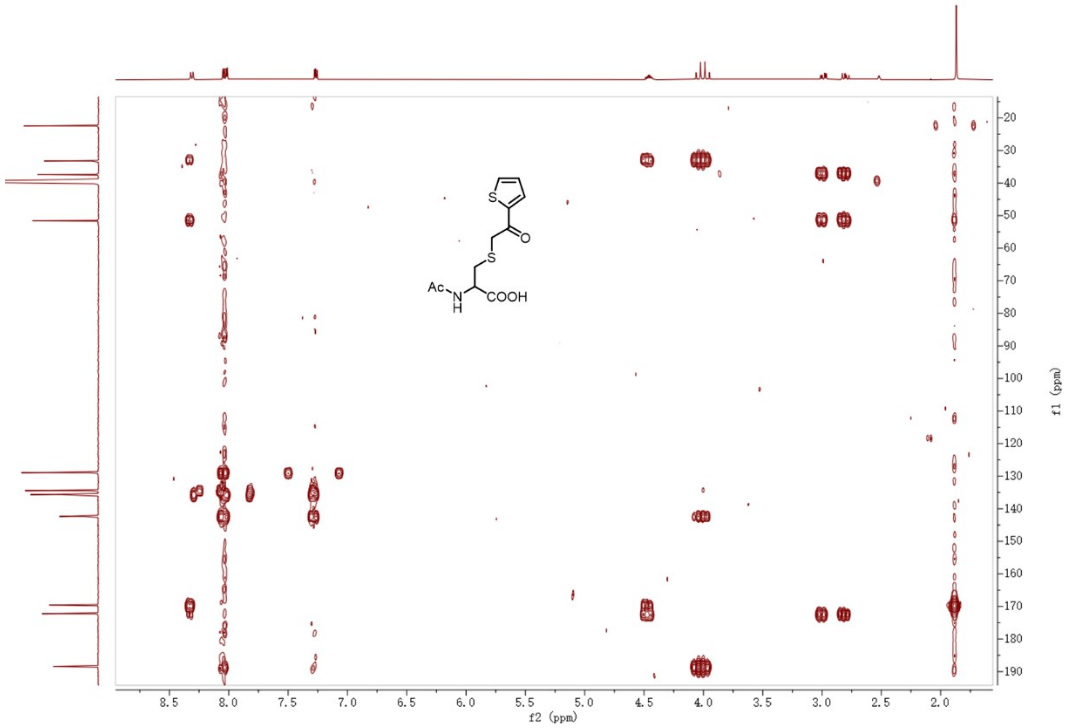

^{1}H-^{13}C HMBC NMR spectrum of **2h**

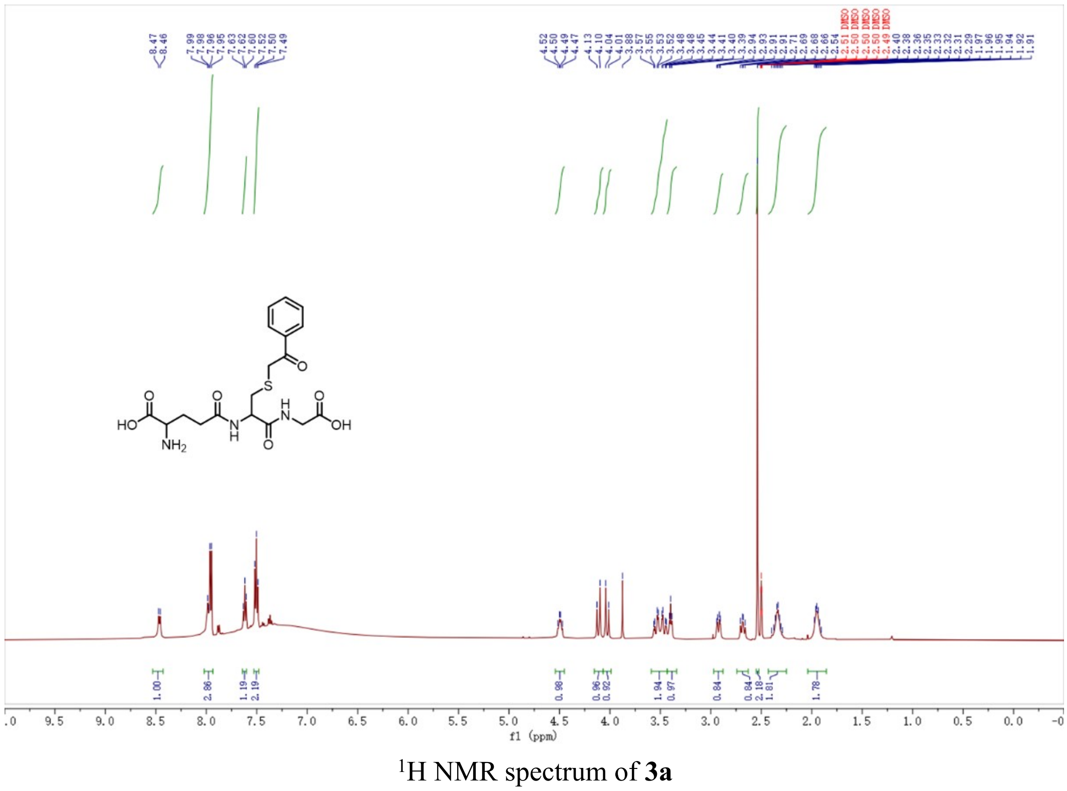

^{1}H NMR spectrum of **3a**

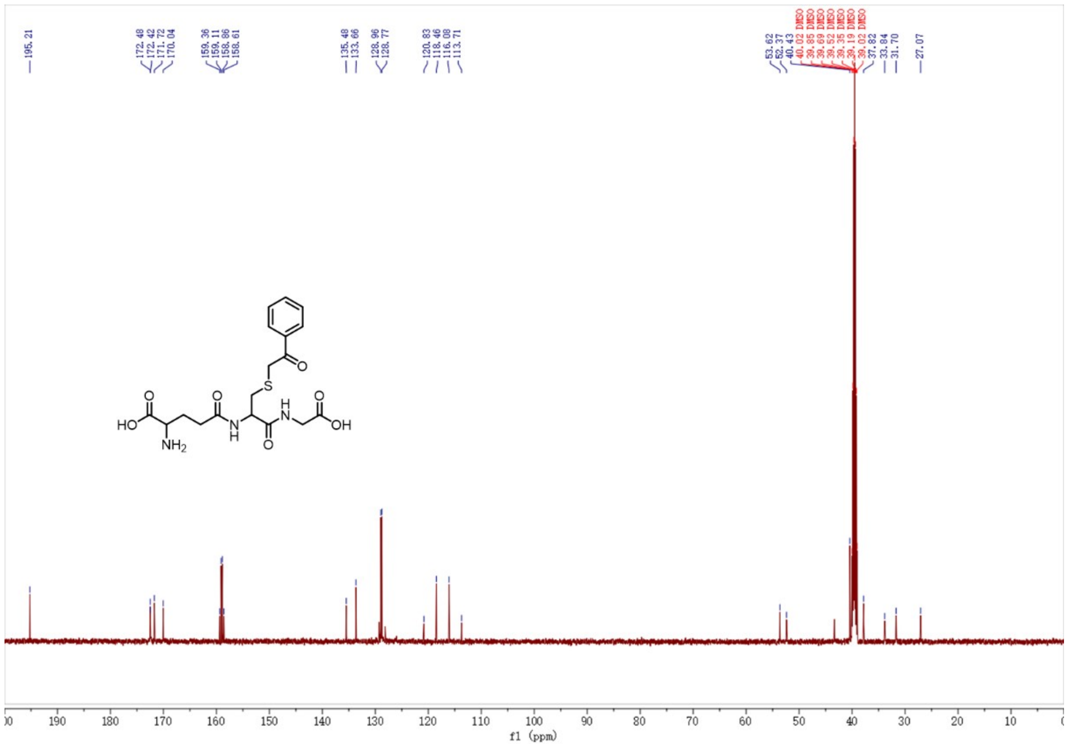

^{13}C NMR spectrum of **3a**

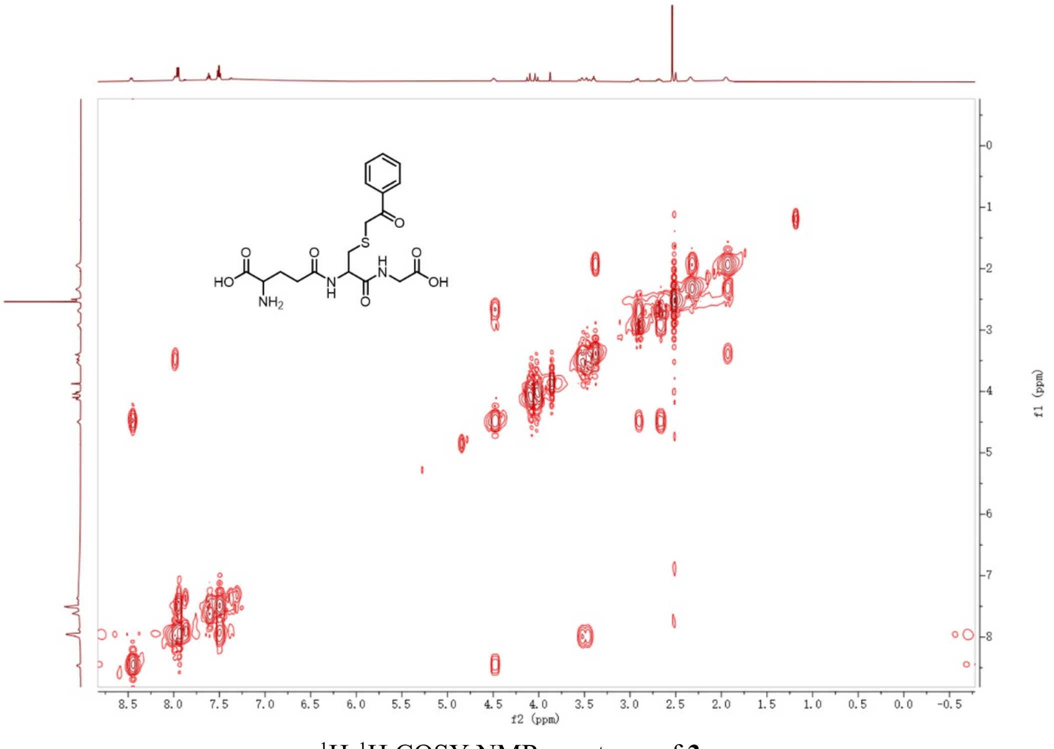

^{1}H-^{1}H COSY NMR spectrum of **3a**

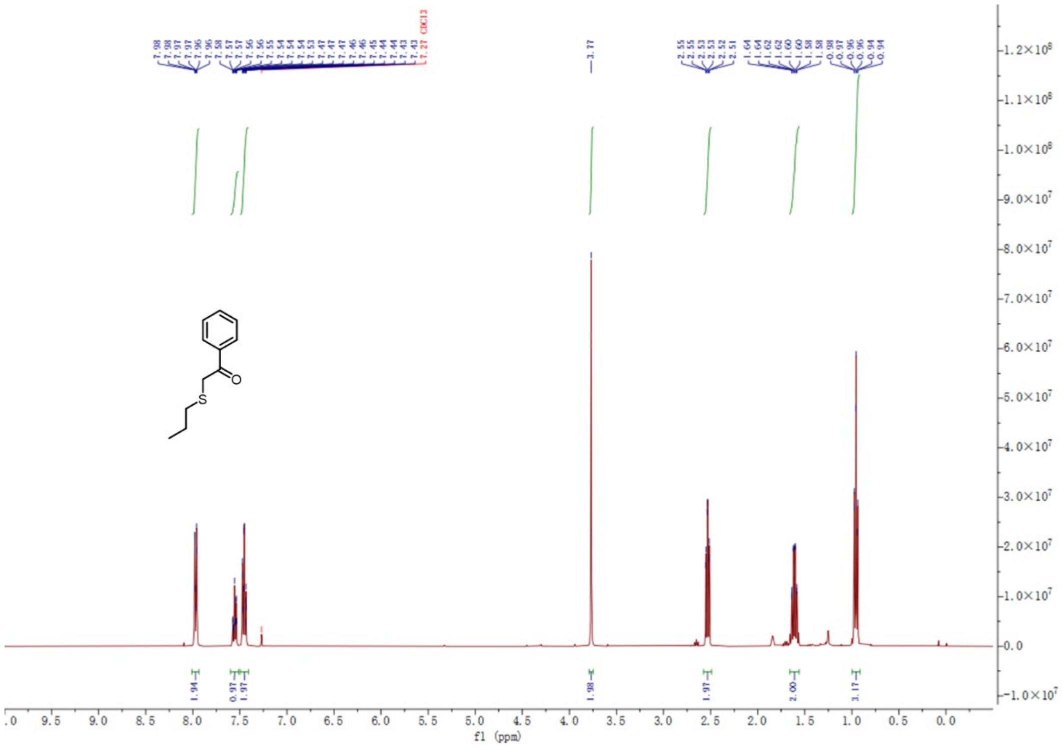

^{1}H NMR spectrum of **4a**

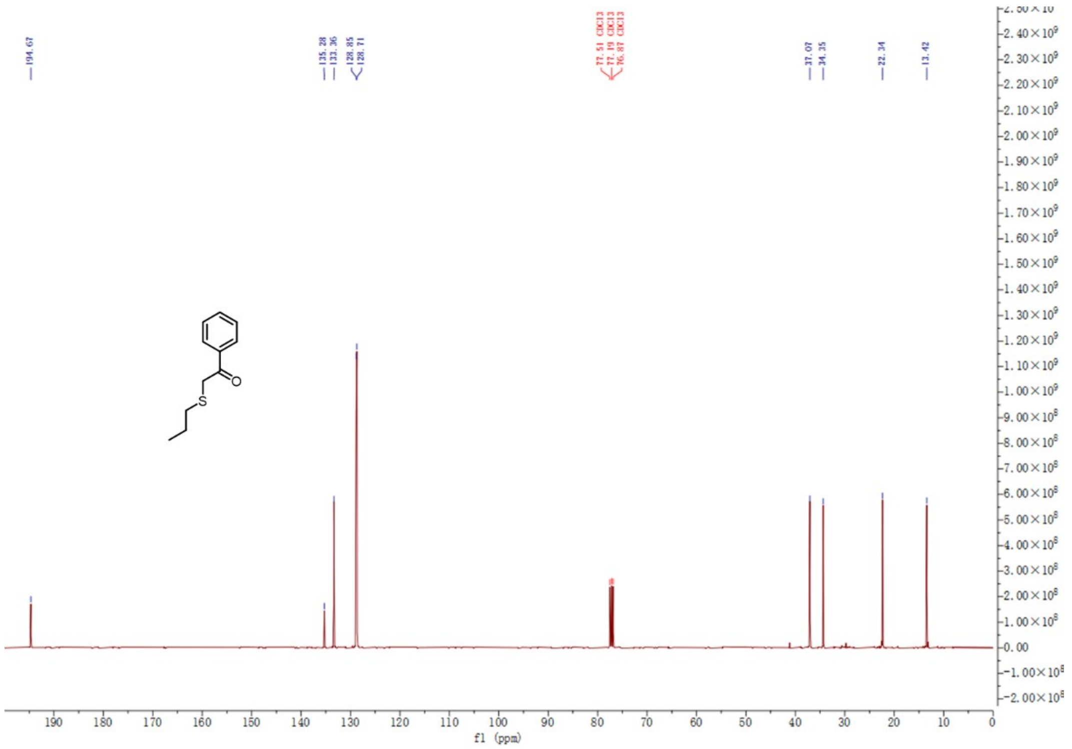

^{13}C NMR spectrum of **4a**

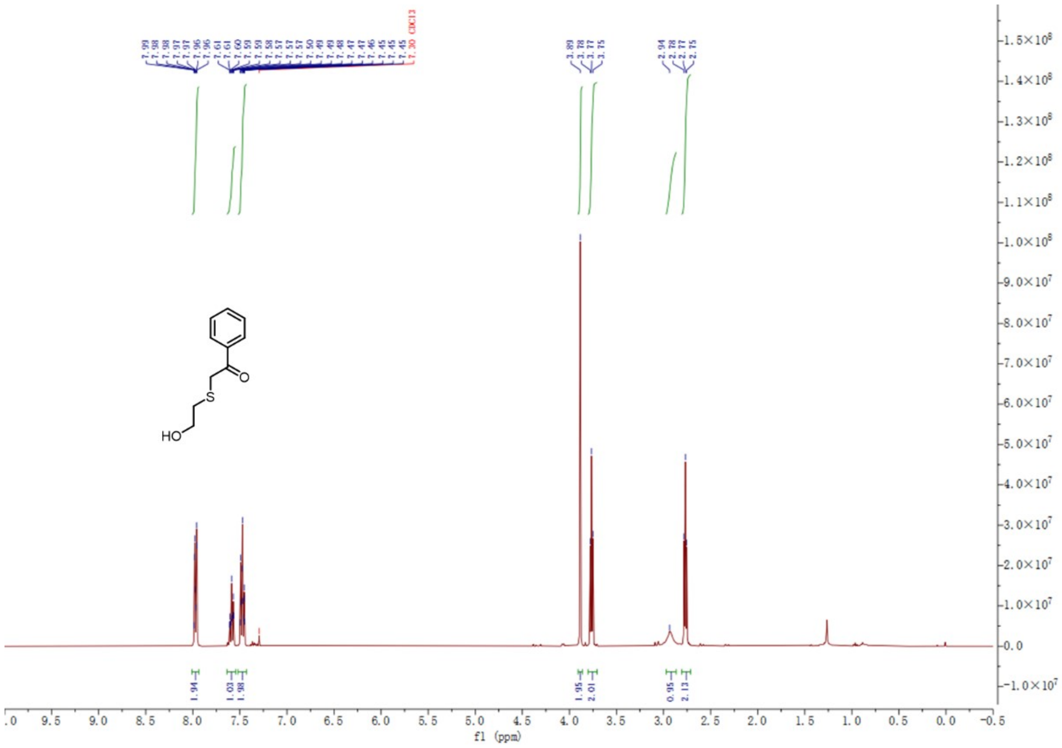

^{1}H NMR spectrum of **5a**

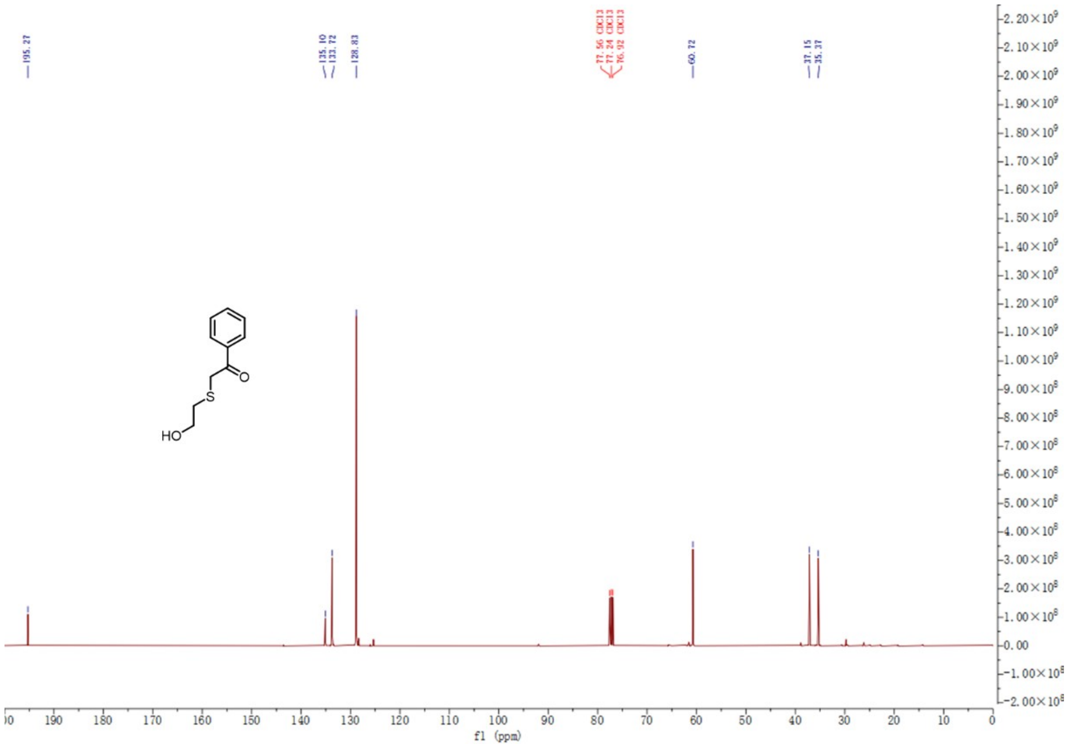

^{13}C NMR spectrum of **5a**

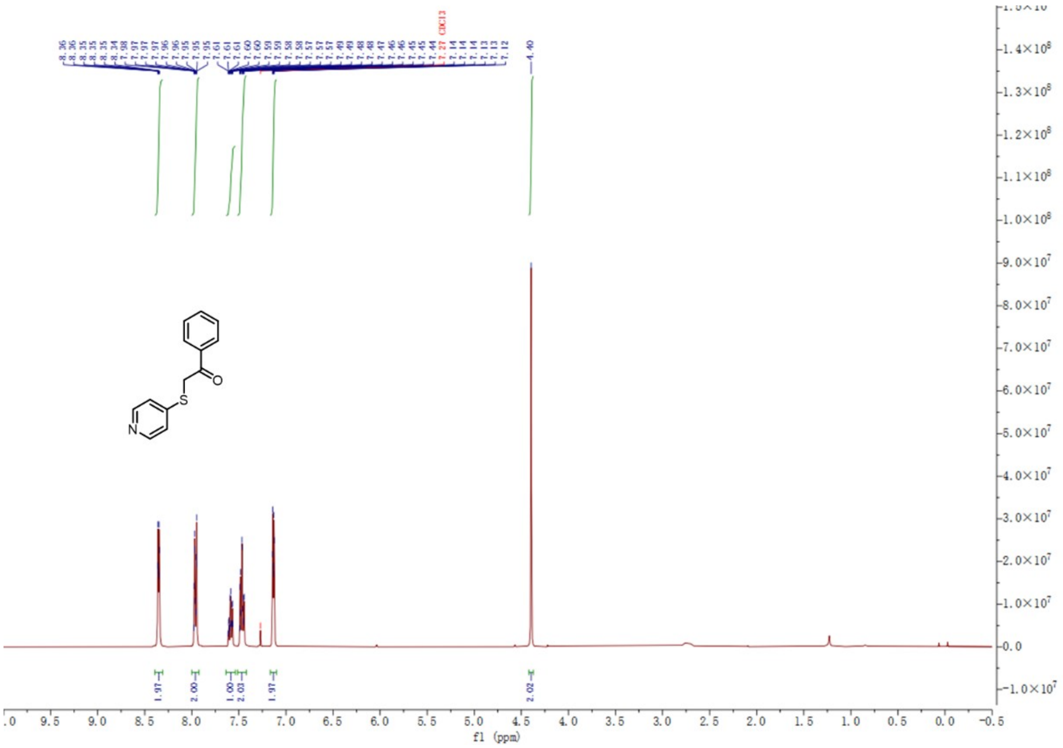

^{1}H NMR spectrum of **6a**

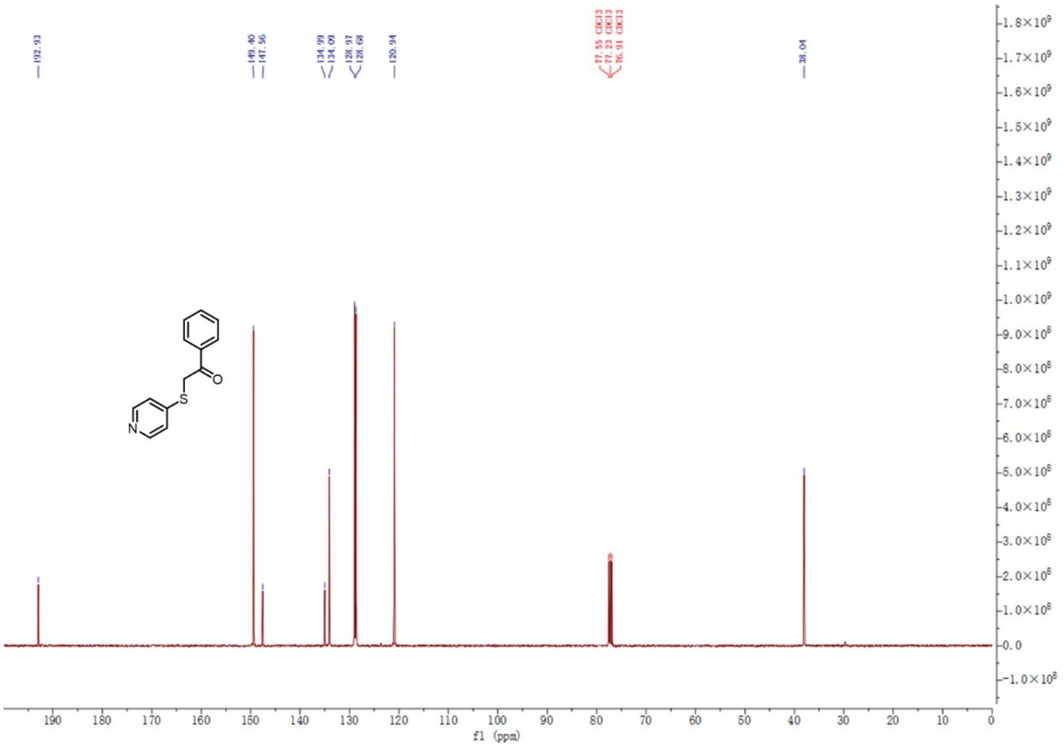

^{13}C NMR spectrum of **6a**
